# Higher versus lower positive end-expiratory pressure in patients without acute respiratory distress syndrome: a meta-analysis of randomized controlled trials

**DOI:** 10.1186/s13054-021-03669-4

**Published:** 2021-07-15

**Authors:** Tommaso Pettenuzzo, Annalisa Boscolo, Alessandro De Cassai, Nicolò Sella, Francesco Zarantonello, Paolo Persona, Laura Pasin, Giovanni Landoni, Paolo Navalesi

**Affiliations:** 1 Institute of Anesthesia and Intensive Care, University Hospital of Padua, 13 Via Gallucci, 35121 Padua, Italy; 2 Department of Medicine, Institute of Anesthesia and Intensive Care, University of Padua, Padua, Italy; 3 Anesthesia and Intensive Care Department, IRCCS San Raffaele Scientific Institute, Milan, Italy; 4 School of Medicine, Vita-Salute San Raffaele University, Milan, Italy

**Keywords:** Mechanical ventilation, Positive end-expiratory pressure, Mortality, Meta-analysis

## Abstract

**Background:**

We conducted a systematic review and meta-analysis of randomized controlled trials (RCTs) to assess the association of higher positive end-expiratory pressure (PEEP), as opposed to lower PEEP, with hospital mortality in adult intensive care unit (ICU) patients undergoing invasive mechanical ventilation for reasons other than acute respiratory distress syndrome (ARDS).

**Methods:**

We performed an electronic search of MEDLINE, EMBASE, Scopus, Cochrane Central Register of Controlled Trials, CINAHL, and Web of Science from inception until June 16, 2021 with no language restrictions. In addition, a research-in-progress database and grey literature were searched.

**Results:**

We identified 22 RCTs (2225 patients) comparing higher PEEP (1007 patients) with lower PEEP (991 patients). No statistically significant association between higher PEEP and hospital mortality was observed (risk ratio 1.02, 95% confidence interval 0.89–1.16; *I*^2^ = 0%, *p* = 0.62; low certainty of evidence). Among secondary outcomes, higher PEEP was associated with better oxygenation, higher respiratory system compliance, and lower risk of hypoxemia and ARDS occurrence. Furthermore, barotrauma, hypotension, duration of ventilation, lengths of stay, and ICU mortality were similar between the two groups.

**Conclusions:**

In our meta-analysis of RCTs, higher PEEP, compared with lower PEEP, was not associated with mortality in patients without ARDS receiving invasive mechanical ventilation. Further large high-quality RCTs are required to confirm these findings.

**Supplementary Information:**

The online version contains supplementary material available at 10.1186/s13054-021-03669-4.

## Background

Mechanical ventilation (MV) is a potentially life-saving strategy for critically ill patients; however, it can exacerbate or promote lung injury through several mechanisms, overall named ventilator-induced lung injury (VILI) [1]. These include alveolar overdistension (volutrauma and barotrauma) and repetitive opening and closing of the alveoli and small airways (atelectrauma) [2]. Positive end-expiratory pressure (PEEP) has been employed to prevent end-expiratory alveolar collapse and counteract mechanical strain, thus minimizing atelectrauma and alveolar overdistension, respectively [3]. Nonetheless, excessive PEEP may worsen mechanical stress [4, 5] and lead to hemodynamic impairment [6].

Patients suffering from the acute respiratory distress syndrome (ARDS) are more at risk of VILI owing to the small size of the ventilated lung regions and the high number of parenchymal heterogeneities, where mechanical injury may be amplified [7]. The use of higher PEEP, compared to lower PEEP, is associated with reduced mortality among patients with moderate-severe ARDS [8], who better respond to the application of PEEP in terms of increased size of ventilated lung [9], reduced atelectrauma [10], and improved oxygenation [11] than patients with mild ARDS.

Similar to patients with less severe ARDS, patients without ARDS may have a low potential for lung recruitment and be exposed to the increased risk of PEEP-related lung overdistension and hemodynamic compromise [5, 6, 12]. Recently, the restricted versus liberal positive end-expiratory pressure in patients without ARDS (RELAx) study provided evidence that a MV strategy using lower PEEP is non-inferior to a strategy based on higher PEEP in terms of the number of ventilator-free days at day 28 among intensive care unit (ICU) patients without ARDS [13]. A meta-analysis, overall including 1393 individuals from 21 randomized controlled trials (RCTs), showed that higher levels of PEEP were not associated with lower in-hospital mortality or shorter duration of ventilation, but with improved oxygenation and lower risk of hypoxemia and development of ARDS [14]. However, this meta-analysis did not include the RELAx trial, which is by far the largest RCT evaluating the role of PEEP in ICU patients without ARDS. We conducted this updated meta-analysis of RCTs to assess the association of higher PEEP, as opposed to lower PEEP, with hospital mortality in adult ICU patients intubated and mechanically ventilated for reasons other than ARDS.

## Methods

### Inclusion and exclusion criteria

The following participants, interventions, comparisons, outcomes, and study design (PICOS) question was employed: participants were adult patients undergoing invasive MV in the ICU for reasons other than ARDS; the intervention and comparison were higher PEEP and lower PEEP, respectively; the primary outcome was hospital mortality at the longest follow-up (secondary outcomes are listed in Additional File 1: Online Resource 1); study design was RCT. Studies not comparing different levels of PEEP at the same ventilatory settings were excluded.

### Literature search

We performed an electronic search of MEDLINE, EMBASE, Scopus, Cochrane Central Register of Controlled Trials, CINAHL, and Web of Science from inception until June 16, 2021 with no language restrictions. In addition, a research-in-progress database (ClinicalTrials.gov) and grey literature (OpenGrey) were searched. All references of included articles and related reviews were hand searched. Conference proceedings were excluded.

Controlled vocabulary terms (when available), text words, and keywords were variably combined with blocks of terms per concept: (“positive end-expiratory pressure” OR “PEEP”) AND (“randomized controlled trial” OR “RCT”). MEDLINE and Scopus search strategies were adapted for searches in other databases and are reported in Additional File 1: Online Resource 1.

### Data collection

The search results were merged and duplicate records of the same report were removed. The remaining studies were stored using Microsoft Excel software (Microsoft Corporation, Redmond, WA). Titles and abstracts were independently screened by two authors (TP, AB) according to the inclusion criteria, and the full texts of potentially relevant reports were retrieved. Reasons for exclusions were detailed and studies that were uncertain for inclusion were listed (Additional File 1: Online Resource 2). The full-text reports were independently examined by four authors (TP, AB, ADC, and NS). Data from included studies were recorded using a Microsoft Excel specific report form. TP and AB independently verified all extracted data for accuracy. All disagreements were resolved by referral to a third author (LP) if necessary. When data about the primary outcome were missing, we planned to contact the corresponding author of the original study with a triple e-mail reminder.

### Risk of bias assessment

The risk of bias (ROB) of the included studies was independently assessed by three authors (TP, PP, and FZ) according to the revised Cochrane ROB tool for randomized trials (RoB 2) [15]. RoB2 examines 5 domains of bias: (1) randomization process; (2) deviations from intended interventions; (3) missing outcome data; (4) measurement of the outcome; and (5) selection of the reported results. The overall RoB judgment at domain and study level was attributed according to the criteria specified in the RoB 2 tool [15]. The risk of bias of individual studies was examined at the study level. All disagreements were resolved by discussion or referral to a third author (LP) if necessary.

### Data synthesis and analysis

Meta-analyses were performed using a random-effects model to account for between-study heterogeneity [16]. The treatment effect for continuous outcomes was analyzed with the inverse variance method and expressed as mean difference (MD) or standardized mean difference (SMD) with 95% confidence interval (CI), as appropriate. The treatment effect for dichotomous outcomes was analyzed with the Mantel–Haenszel method and expressed as risk ratio (RR) with 95% CI. The 95% prediction interval (PI) was reported alongside the 95% CI as an estimate of the dispersion of the true effect size across different study conditions. While CI indicates the effect size, PI represents the interval the mean effect of a new study will fall in when included.

Statistical heterogeneity for the outcomes among studies was assessed using the *I*^2^ test. We considered *I*^2^ ≥ 75% and *p* < 0.1 as high heterogeneity [16]. Publication bias was assessed by visually inspecting a funnel plot for potential asymmetry and Egger’s test was applied when the number of studies was adequate (*n* > 10). For the primary outcome and main secondary outcomes, we assessed the certainty of evidence using the grades of recommendation, assessment, development, and evaluation (GRADE) approach [17]. Where necessary, we converted the reported median and interquartile range to estimated mean and standard deviation using a standard approach [18].

We conducted several pre-specified sensitivity analyses to assess the impact of (1) different timing of measurement of the variables; (2) use of odds ratio versus RR as effect estimate in dichotomous variables; (3) exclusion of the studies at high risk of bias. Furthermore, the following pre-planned subgroup analyses were carried out: (1) medical versus surgical patients, which may represent different patient populations with different outcomes; (2) zero end-expiratory pressure (ZEEP) versus PEEP as lower PEEP, which may differently affect the outcome; (3) tidal volumes (TVs) greater versus lower than 8 mL/kg, which is the threshold for protective TVs that is commonly considered in non-ARDS patients [19]; (4) studies published after 2000 versus before 2000, when the landmark ARDSnet trial demonstrating a survival benefit associated with TV reduction in ARDS patients was published [20].

To investigate the influence of TVs on the effect size for the primary outcome and main secondary outcomes, we performed a post hoc meta-regression. Finally, we conducted a trial sequential analysis (TSA) considering a type I error of 5%, a power of 90%, and a hospital mortality proportion in the lower PEEP arm of 33% (median event proportion in this group among the included studies). The two-sided α-spending boundaries and futility area were computed with the O’Brien-Fleming function. An 11% relative risk reduction was calculated based on the lower limit of the 95% CI for the RR of the primary outcome in the meta-analysis, and the required information size was calculated accordingly.

All analyses were performed with Review Manager (version 5.3, Nordic Cochrane Centre, Cochrane Collaboration), R 4.0.3 with Rcmdr plugin (R Foundation for Statistical Computing, Vienna, Austria; https://www.R-project.org), and the Trial Sequential Analysis software (version 0.9.5.10, Copenhagen Trial Unit, Centre for Clinical Intervention Research, Copenhagen). For all analyses, two-sided *p* values < 0.05 were considered significant.

Reporting of this systematic review and meta-analysis conformed to preferred reporting items for systematic reviews and meta-analyses (PRISMA) standards (Additional File 1: Online Resource 3). This systematic review was registered in PROSPERO, an international prospective register of systematic reviews (www.crd.york.ac.uk/PROSPERO, CRD42021237164).

## Results

**Fig. 1:**
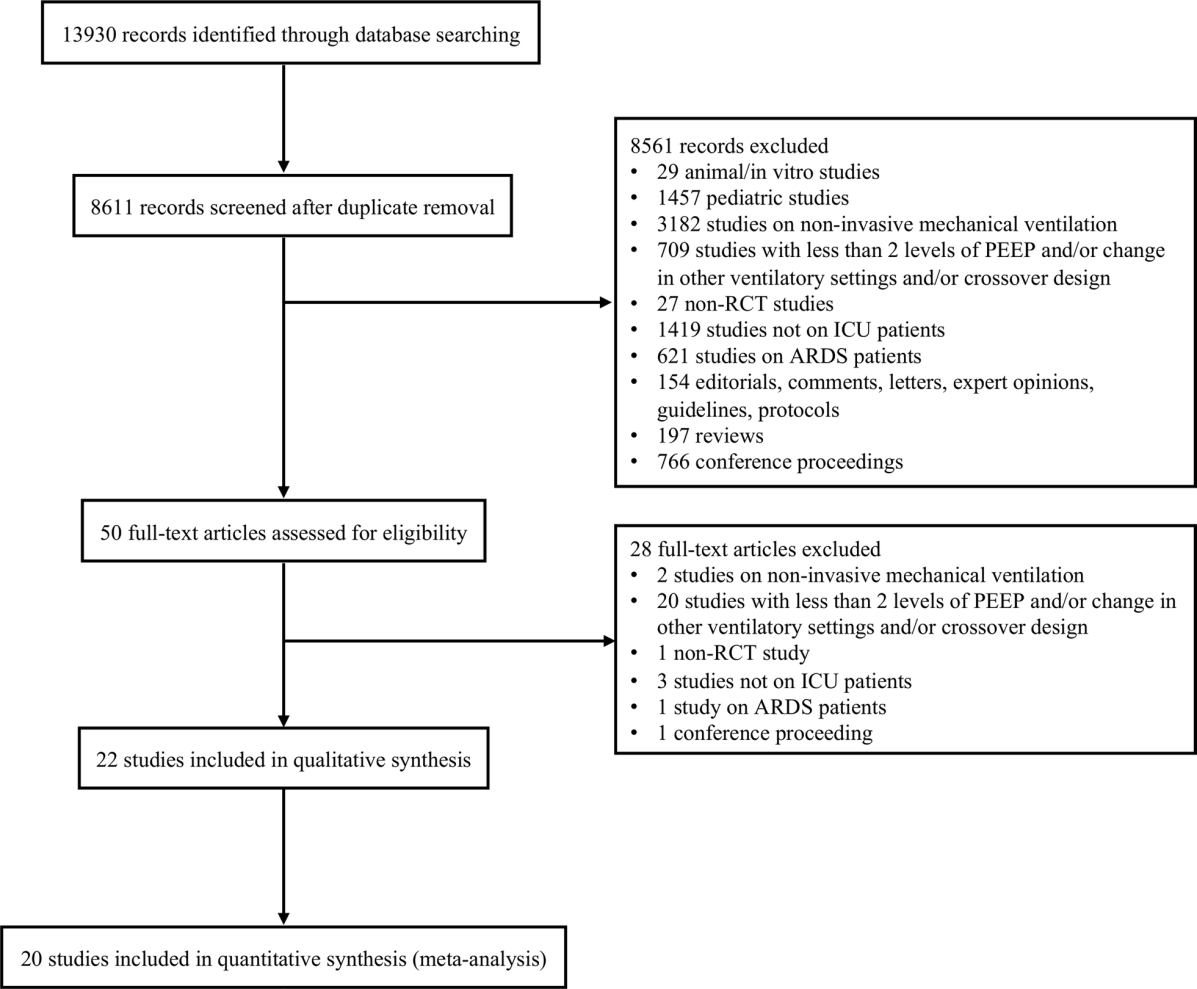
Study flow diagram. *PEEP* positive end-expiratory pressure, *RCT* randomized controlled trials, *ICU* intensive care unit, *ARDS* acute respiratory distress syndrome

We identified 8611 unique citations and assessed the full text of 50 articles for eligibility. Of these, 22 RCTs (2225 patients) [13, 21–41] comparing higher PEEP (1007 patients) with lower PEEP (991 patients) were included in the systematic review (Fig. 1). One study [40] did not report the number of randomized patients and the intermediate PEEP groups of four studies [23, 24, 31, 32] were excluded, hence the mismatch between the total number of included patients and the sum of the patients in the two groups. Data on the primary outcome were complete in the published reports of the studies.

The characteristics of the included RCTs are reported in Additional File 1: Online Resource 4. The studies were published between 1975 and 2020. Patient populations ranged from 15 to 969 patients. Ten studies (45%) enrolled post-cardiac surgery patients, six studies (27%) included patients with acute respiratory failure, four studies (18%) recruited patients with brain injury, and two studies (9%) non-hypoxemic patients. Higher PEEP ranged from 5 to 30 cmH_2_O (median 9 cmH_2_O, interquartile range [IQR] 8–10 cmH_2_O), whereas lower PEEP varied between ZEEP and 10 cmH_2_O (median 0 cmH_2_O, IQR 0–5 cmH_2_O). ZEEP was employed as lower PEEP in 13 studies (59%). PEEP was titrated arbitrarily in 17 studies (77%). TVs ranged between 6 and 15 mL/kg (median 8 mL/kg, IQR 7–11 mL/kg).

The overall risk of bias was judged to be high or moderate because of concerns related to the randomization process and the measurement of the outcome (Additional File 1: Online Resource 5).

**Fig. 2:**
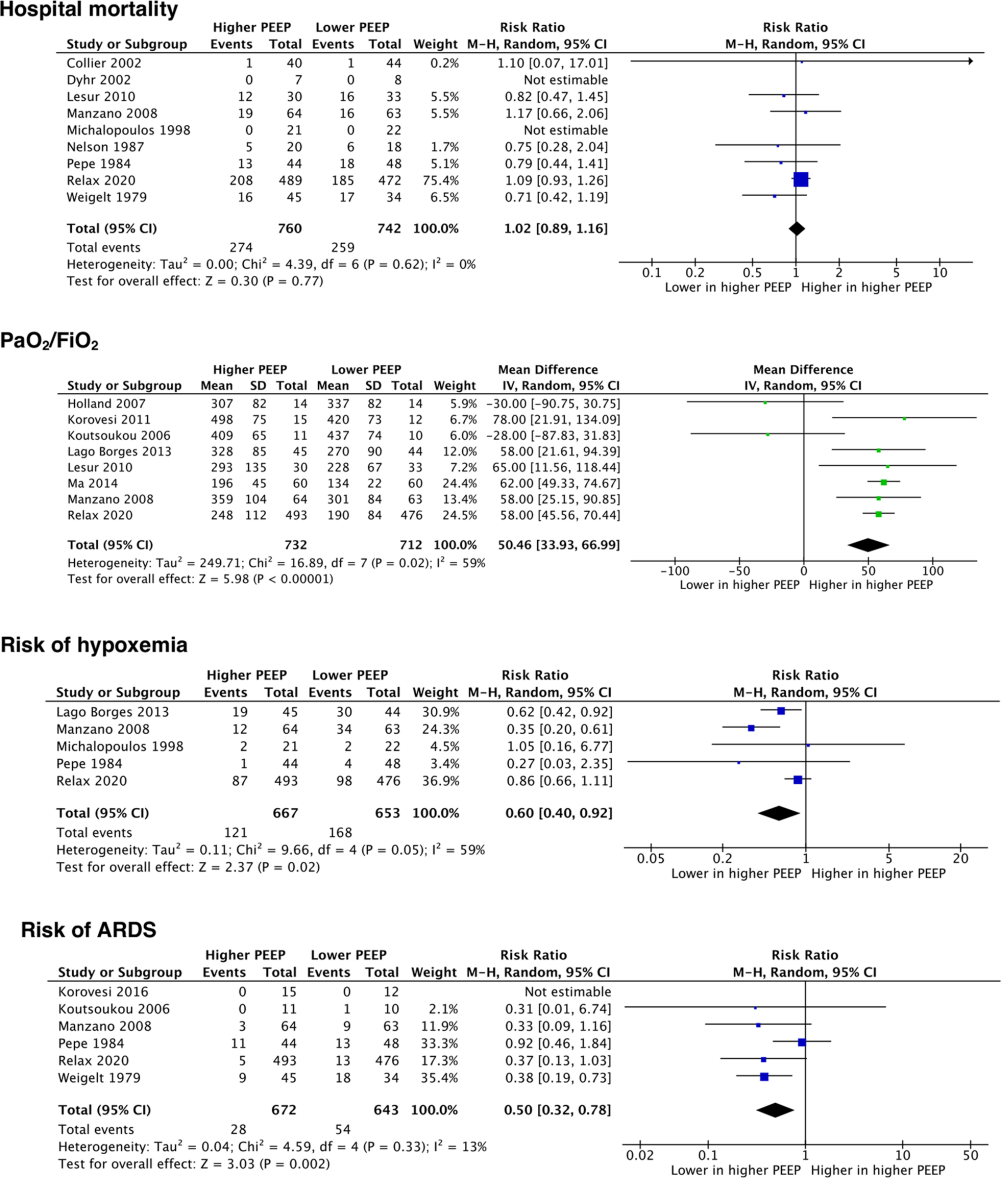
Forest plots of primary outcome and main secondary outcomes. *PEEP* positive end-expiratory pressure, *M–H* Mantel–Haenszel, *CI* confidence interval, *PaO*_*2*_*/FiO*_*2*_ arterial partial pressure of oxygen to fraction of inspired oxygen ratio, *SD* standard deviation, *IV* inverse variance, *ARDS* acute respiratory distress syndrome

The primary outcome was reported in 9 studies. Two hundred seventy-four patients out of 760 patients (36%) assigned to the higher PEEP group and 259 out of 742 patients (35%) assigned to the lower PEEP group died in the hospital (RR 1.02, 95% CI 0.89–1.16, *p* = 0.77; *I*^2^ = 0%, *p* = 0.62) (Fig. 2). The difference was not statistically significant. As shown in Additional File 1: Online Resource 6, higher PEEP was associated with improved arterial partial pressure of oxygen to fraction of inspired oxygen ratio (PaO_2_/FiO_2_), alveolar-arterial oxygen pressure difference (A-aDO_2_), and respiratory system compliance. Furthermore, we observed lower risk of hypoxemia and ARDS occurrence in the higher PEEP group. The risk of atelectasis, barotrauma, ventilator-associated pneumonia, and hypotension was similar in the 2 groups. Cardiac index, postoperative bleeding, and packed red blood cell transfusion were also no different, whereas central venous pressure was significantly higher in the higher PEEP group. Duration of ventilation, ICU and hospital length of stay, and ICU and 28-day mortality were similar between groups (Additional File 1: Online Resource 6).

The presence of publication bias was strongly suspected from the visual inspection of the funnel plot for the duration of ventilation outcome (Additional File 1: Online Resource 7). Egger’s test was not applied because of the insufficient number of included studies. According to the GRADE approach, the certainty of evidence for hospital mortality was low (Table 1). We rated down the quality of evidence because of concerns related to the risk of bias.

**Table 1:** Grades of recommendation, assessment, development, and evaluation summary of findings table

Certainty assessment								Summary of findings			
Participants (studies)	Risk of bias		Inconsistency	Indirectness	Imprecision	Other considerations	Overall certainty of evidence	Higher PEEP (N/total or N)		Lower PEEP (N/total or N)	Relative effect (RR, MD or SMD, 95% CI) of higher PEEP
*Hospital mortality*											
1502 (9 RCTs)	Very serious^a^	Not serious		Not serious	Not serious	None	⨁⨁◯◯ LOW	274/760 (36.1%)	259/742 (34.9%)		1.02 (0.89 to 1.16)
PaO_2_/FiO_2_											
1444 (8 RCTs)	Serious^b^		Serious^c^	Not serious	Not serious	Strong association	⨁⨁⨁◯ MODERATE	732		712	50.46 mmHg (33.93 to 66.99)
Compliance											
189 (3 RCTs)	Very serious^d^		Not serious	Not serious	Not serious	None	⨁⨁◯◯ LOW	101		88	8.46 mL/cmH_2_O (3.11 to 13.82)
Hypoxemia											
1320 (5 RCTs)	Very serious^e^		not Serious	Not serious	Not serious	None	⨁⨁◯◯ LOW	121/667 (18.4%)		168/653 (25.7%)	0.60 (0.40 to 0.92)
Barotrauma											
1372 (7 RCTs)	Very serious^f^		Not serious	Not serious	Not serious	None	⨁⨁◯◯ LOW	38/697 (5.5%)		52/675 (7.7%)	0.78 (0.55 to 1.11)
ARDS											
1315 (6 RCTs)	Very serious^g^		Not serious	Not serious	Not serious	None	⨁⨁◯◯ LOW	28/672 (4.2%)		54/643 (8.4%)	0.50 (0.32 to 0.78)
Duration of ventilation											
1510 (10 RCTs)	Very serious^h^		Serious^i^	Not serious	Not serious	Publication bias strongly suspected^j^	⨁◯◯◯ VERY LOW	771		739	− 0.03 (− 0.27 to 0.21)
Hospital stay											
1245 (5 RCTs)	Serious^k^		Not serious	Not serious	Not serious	None	⨁⨁⨁◯ MODERATE	629		616	− 0.02 (− 0.69 to 0.66)

PEEP, positive end-expiratory pressure; N, number of patients; RR: risk ratio; MD, mean difference; SMD: standardized mean difference; CI, confidence interval; RCT, randomized controlled trials; PaO_2_/FiO_2_, arterial partial pressure of oxygen to fraction of inspired oxygen ratio; ARDS, acute respiratory distress syndrome. “Other considerations” include publication bias, large effect, plausible confounding, and dose response gradient

^a^Five studies were at high risk of bias; four studies were at moderate risk of bias

^b^Six studies were at moderate risk of bias; two studies were at high risk of bias

^c^Moderate heterogeneity (*I*^2^ = 59%) present across studies

^d^Three studies were at high risk of bias

^e^Three studies were at high risk of bias; two studies were at moderate risk of bias

^f^Five studies were at high risk of bias; two studies were at moderate risk of bias

^g^Four studies were at high risk of bias; two studies were at moderate risk of bias

^h^Six studies were at high risk of bias; four studies were at moderate risk of bias

^i^Moderate heterogeneity (*I*^2^ = 65%) present across studies

^j^Visual inspection of the funnel plot suggests that publication bias cannot be excluded

^k^Three studies were at moderate risk of bias; two studies were at high risk of bias

**Fig. 3:**
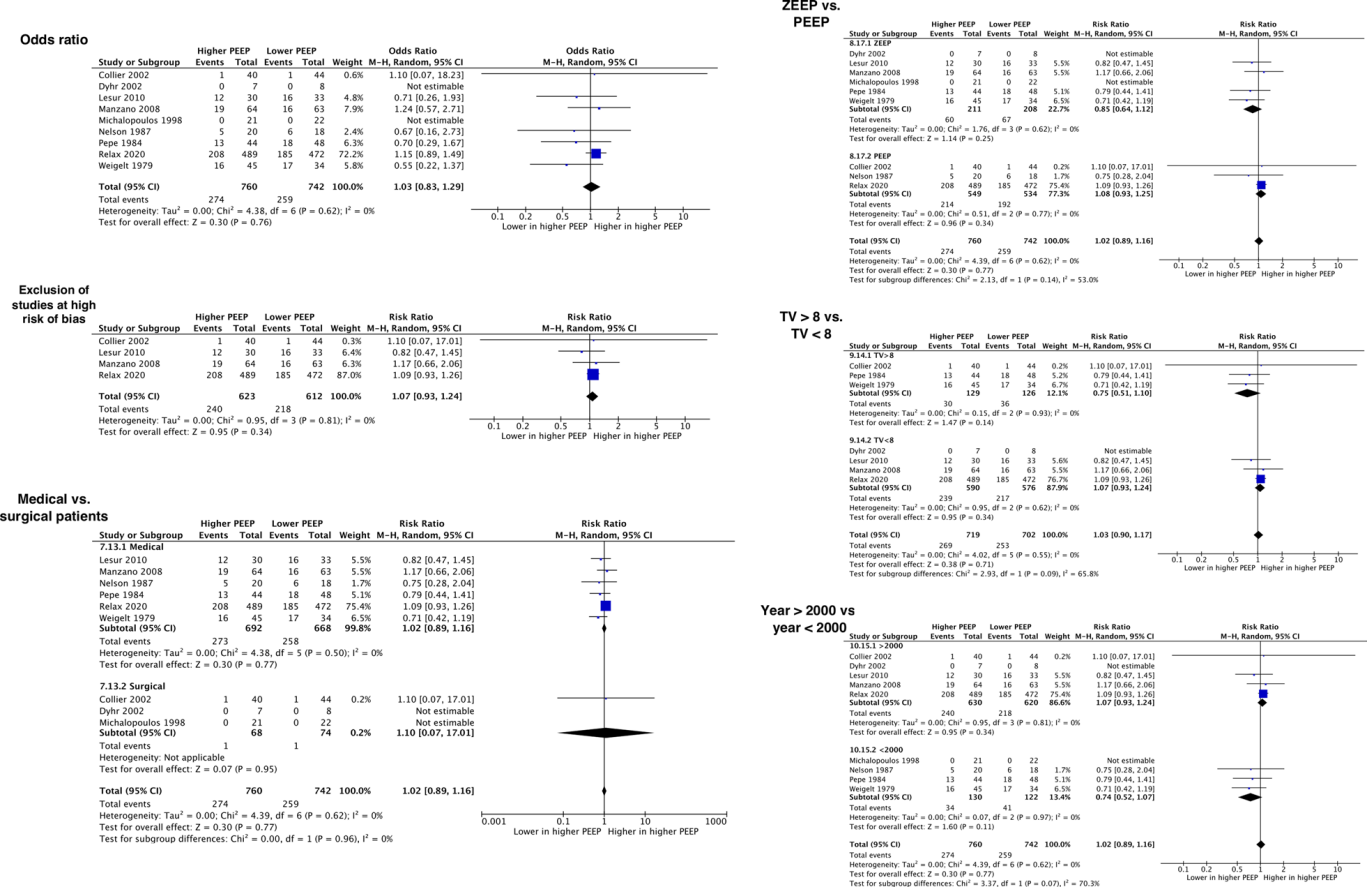
Forest plots of sensitivity and subgroup analyses for hospital mortality. Sensitivity analyses to assess the impact of the use of odds ratio versus risk ratio as effect estimate (top left) and the exclusion of the studies at high risk of bias (middle left) are reported. Subgroup analyses comparing medical versus surgical patients (bottom left), zero end-expiratory pressure versus positive end-expiratory pressure (PEEP) as lower PEEP (top right), tidal volumes greater versus lower than 8 mL/kg (middle right), and publication year after 2000 versus before 2000 (bottom right) are depicted. *PEEP* positive end-expiratory pressure, *M–H* Mantel–Haenszel, *CI* confidence interval, *ZEEP* zero end-expiratory pressure, *TV* tidal volume

The forest plots for sensitivity and subgroup analyses for hospital mortality are depicted in Fig. 3. None of the sensitivity or subgroup analyses for the primary and secondary outcomes significantly affected our results (Additional File 1: Online Resource 8, 9). We observed a significantly lower incidence of hypoxemia with higher PEEP in studies comparing higher PEEP with ZEEP versus studies comparing higher PEEP with lower PEEP (3 studies with ZEEP, 2 studies with PEEP; test for subgroup differences *p* = 0.02) (Additional File 1: Online Resource 9). A post hoc meta-regression revealed a significant negative association between TVs and the risk of hypoxemia (coefficient − 0.41, 95% CI − 0.77 to − 0.06, *p* = 0.02) (Additional File 1: Online Resource 10). The trial sequential analysis showed that the required information size of 6845 patients was not reached and the cumulative Z-score did not cross the monitoring boundaries (Additional File 1: Online Resource 11).

## Discussion

Our systematic review identified 22 RCTs, overall including 2225 patients, that evaluated the association of higher PEEP, compared to lower PEEP, with hospital mortality in unselected adult ICU patients intubated and mechanically ventilated for reasons other than ARDS. We concluded that hospital mortality was similar between the two groups with low certainty of evidence.

With respect to the secondary outcomes, higher PEEP was associated with higher PaO_2_/FiO_2_ ratio and lower A-aDO_2_, higher respiratory system compliance, and lower risk of hypoxemia and ARDS occurrence. Other secondary outcomes (e.g., occurrence of atelectasis, barotrauma, ventilation-associated pneumonia, hypotension, duration of ventilation, ICU and hospital stay, and ICU mortality) were similar between the two groups. The overall certainty of evidence was judged to be low or moderate for most secondary outcomes.

The role of PEEP during MV in patients with ARDS has been a matter of intense research. Based primarily on the results of an individual patient data meta-analysis reporting lower hospital mortality with higher PEEP among patients with moderate-severe ARDS [8], higher PEEP was suggested with a conditional recommendation in the most recent guidelines on MV in patients with ARDS [42].

The net benefit or harm of PEEP may depend on lung recruitability, i.e., the reaeration of non-aerated and/or poorly aerated lung tissue [5]. Nevertheless, the importance of lung recruitability and patient’s response to recruitment maneuvers are currently not fully understood. Patients without ARDS, like those with ARDS, may exhibit low lung recruitability and develop end-inspiratory alveolar overdistention, increased intrapulmonary shunt and dead space, and higher right ventricular afterload with higher PEEP [12]. These patients may benefit from a MV strategy including lower TVs, which were associated with improved clinical outcomes [43–45]. However, the effect of PEEP in patients without ARDS is still not well understood. To our knowledge, this is the second systematic review and meta-analysis to compare different levels of PEEP in ICU patients without ARDS. Similar to our findings, a previous work concluded that ventilation with higher PEEP is not associated with lower hospital mortality or shorter duration of ventilation, but with higher PaO_2_/FiO_2_ and lower occurrence of ARDS and hypoxemia [14]. We believe our work has some strengths compared to the previously published meta-analysis. Three studies were not confirmed for inclusion in our work because of noninvasive application of PEEP [46], crossover design [47], and differential utilization of recruitment maneuvers in the two groups [48]. On the other hand, five additional studies were selected [13, 25, 27, 29, 30]. Among these, the recent RELAx trial found that the use of lower PEEP was non-inferior to higher PEEP in terms of 28-day ventilator-free days [13]. Additionally, no difference between the groups in the occurrence of pulmonary complications, lengths of stay, and mortality was reported [13]. This trial is by far the largest RCT on this topic, accounting for more than 40% of patients included in our systematic review.

Despite the higher number of included patients and stricter selection criteria than the previous meta-analysis [14], we did not identify any association of higher PEEP with clinical outcomes, except for ARDS occurrence, but confirmed its association with physiological outcomes such as oxygenation. Several reasons may account for this.

First, PEEP may not be the most important target for preventing VILI in patients without ARDS. Driving pressure (DP), i.e., the difference between plateau pressure and total PEEP [49], and mechanical power (MP), i.e., the total inflation energy transferred from the mechanical ventilator to the lungs [50], have been recently proposed as reliable mediators of the injurious effects of MV in patients with ARDS [51–54]. Notably, patients without ARDS may benefit from lower DP [55–57] and MP [58], although the evidence is still controversial [59, 60]. Moreover, TVs may be a better surrogate of the risk of VILI in these patients with near-normal respiratory system compliance [61]. Therefore, titrating MV settings on DP and MP rather than PEEP or monitoring DP and MP during PEEP titration may be more effective in minimizing the risk of VILI in non-ARDS patients.

Second, the low certainty of evidence for hospital mortality, related to the high risk of bias of the studies contributing to this outcome, limits the strength of our conclusions. Rigorous RCTs with more selected patient populations would contribute to clarifying whether patients without ARDS may benefit from higher PEEP. Moreover, despite the overall low-moderate statistical heterogeneity, clinical heterogeneity among patient populations and outcome definitions (e.g., ARDS, barotrauma, pneumonia) was relevant in the studies included in our meta-analysis. This may have confounded the association between the level of PEEP and clinical outcomes.

Third, the existence of specific subgroups of patients without ARDS that may mostly benefit from higher PEEP might be hypothesized. However, although we performed several sensitivity and subgroup analyses, including a meta-regression, we could not identify any of such subgroups. Worth mentioning, the choice of PEEP in most studies was made arbitrarily and not individualized according to patients’ response to PEEP or to lung recruitability. Although the improved oxygenation with higher PEEP could depend on alveolar recruitment and reduced intrapulmonary shunt, which may protect against VILI, the use of excessive PEEP could lead to alveolar overdistension, cardiac dysfunction, and reduced oxygen delivery in patients with low lung recruitability regardless of arterial oxygenation [62]. Indeed, a strategy based on titration of the ventilator settings, i.e., tidal volume, PEEP, and recruitment maneuvers, on lung morphology, i.e., focal or non-focal injury distribution, may reduce mortality in ARDS patients [63]. Oxygenation may not be the best physiologic endpoint as regards the effect of PEEP. In fact, respiratory system compliance or DP may be better indices of the balance between recruitment and overdistension associated with variations of PEEP. Unfortunately, no studies provided data on DP and only 3 studies (189 patients) included compliance among the outcomes, thus limiting our confidence in our results. Furthermore, occurrence of hypotension and barotrauma, and measurements of cardiac index and dead space were only reported in a minority of studies. The trial sequential analysis showed that, although further studies are needed, these are unlikely to show any association of PEEP with mortality; this may suggest that patient selection criteria need to be modified in future studies, for example, taking lung recruitability into account.

Finally, physiological benefits may be most likely to translate into clinical advantages when baseline physiological derangements are severe enough. However, baseline oxygenation and compliance, when reported, were near-normal for most patients [13, 24, 28–32, 34, 37–39].

Our study has several limitations. First, we restricted our research to 6 databases and decided not to include conference proceedings. Therefore, we cannot exclude that other studies could have been identified by exploring other databases and that publication bias may have ensued from our search strategy. In addition, we did not perform an individual patient data meta-analysis, thus potentially missing patient subgroups benefiting from higher or lower PEEP. However, we used a reproducible and comprehensive literature search strategy, including clinical trials and grey literature, and we employed clearly defined inclusion criteria and duplicate independent citation review, data extraction, and quality assessment. Second, as the included studies were published over a 45-year period, the heterogeneous clinical protocols and outcome definitions may limit the reliability of our findings. Importantly, very different levels of PEEP and TV were applied in the studies. However, we excluded studies changing MV settings other than PEEP to avoid potential confounding factors. Additionally, our subgroup analyses on the use of ZEEP versus PEEP, TV greater versus lower than 8 mL/kg, and publication year after versus before 2000 did not identify any clinically significant variation in the intervention effect. Finally, statistical heterogeneity was relevant for some secondary outcomes. Notwithstanding, we performed several subgroup analyses and a post hoc meta-regression, which partially explained the reported heterogeneity.

## Conclusions

In our meta-analysis of RCTs including a recent well-powered high-quality RCT, higher PEEP, compared with lower PEEP, was not associated with mortality in patients without ARDS receiving invasive MV. Further large high-quality RCTs are required to confirm these findings.

## Supplementary Information

**Additional File 1: Online Resource 1.** Methods. **Online Resource 2.** Included studies and major exclusions. **Online Resource 3.** Preferred reporting items for systematic reviews and meta-analyses checklist. **Online Resource 4.** Characteristics of included studies. **Online Resource 5.** Risk of bias assessment. **Online Resource 6.** Primary and secondary outcomes. **Online Resource 7.** Funnel plots. **Online Resource 8.** Sensitivity analyses. **Online Resource 9.** Subgroup analyses. **Online Resource 10.** Meta-regression. **Online Resource 11.** Trial sequential analysis.

## Data Availability

The datasets used and/or analyzed during the current study are available from the corresponding author on reasonable request.

## Abbreviations

MV: Mechanical ventilation
VILI: Ventilator-induced lung injury
PEEP: Positive end-expiratory pressure
ARDS: Acute respiratory distress syndrome
RELAx: Restricted versus liberal positive end-expiratory pressure in patients without ARDS
ICU: Intensive care unit
RCT: Randomized controlled trial
PICOS: Participants, interventions, comparisons, outcomes, and study design
ROB: Risk of bias
MD: Mean difference
SMD: Standardized mean difference
CI: Confidence interval
RR: Risk ratio
PI: Prediction interval
GRADE: Grades of recommendation, assessment, development, and evaluation
ZEEP: Zero end-expiratory pressure
TV: Tidal volume
TSA: Trial sequential analysis
PRISMA: Preferred reporting items for systematic reviews and meta-analyses
PaO_2_/FiO_2_: Arterial partial pressure of oxygen to fraction of inspired oxygen ratio
A-aDO_2_: Alveolar-arterial oxygen pressure difference
DP: Driving pressure
MP: Mechanical power
