# Supplementary material for: Higher versus lower positive end-expiratory pressure in patients without acute respiratory distress syndrome: a meta-analysis of randomized controlled trials
Source: Crit Care. 2021 Jul 15;25:247. doi: 10.1186/s13054-021-03669-4 (PMC8280384; doi:10.1186/s13054-021-03669-4)
Supplement: Supplementary file 1 — Additional File 1: Online Resource 1. Methods. Online Resource 2. Included studies and major exclusions. Online Resource 3. Preferred reporting items for systematic reviews and meta-analyses checklist. Online Resource 4. Characteristics of included studies. Online Resource 5. Risk of bias assessment. Online Resource 6. Primary and secondary outcomes. Online Resource 7. Funnel plots. Online Resource 8. Sensitivity analyses. Online Resource 9. Subgroup analyses. Online Resource 10. Meta-regression. Online Resource 11. Trial sequential analysis. [file 13054_2021_3669_MOESM1_ESM.pdf]

Higher versus lower positive end-expiratory pressure in patients without acute respiratory distress syndrome: a meta-analysis of randomized controlled trials

Tommaso Pettenuzzo, Annalisa Boscolo, Alessandro De Cassai, Nicolò Sella, Francesco Zarantonello, Paolo Persona, Laura Pasin, Giovanni Landoni, Paolo Navalesi

Corresponding author:

Tommaso Pettenuzzo, MD

Institute of Anesthesia and Intensive Care, University Hospital of Padua, Padua, Italy, 13 Via Gallucci, 35121 Padua, Italy

Phone: +390498213090; email: tommasopettenuzzo@gmail.com

ORCID iD: 0000-0002-6745-8919

Online Resource Contents

Online Resource 1. Methods……………………………………………………………………………………………………..4

Participants, Interventions, Comparisons, Outcomes, and Study design question……………………………………….4

Database search strategies……………………………………………………………………………………………4

Risk of bias assessment………………………………………………………………………………………………5

Online Resource 2. Included studies and major exclusions…………………………………………………………………7

Studies included in both the qualitative and quantitative review………………………………………………………7

Studies included in the qualitative review only………………………………………………………………………8

Studies on non-invasive mechanical ventilation………………………………………………………………………9

Studies with less than 2 levels of positive end-expiratory pressure (PEEP) and/or change in other ventilatory settings and/or crossover studies...9

Non-RCT study…………………………………………………………………………………………………10

Studies not on ICU patients………………………………………………………………………………………11

Study on ARDS patients…………………………………………………………………………………………11

Conference proceeding……………………………………………………………………………………………11

Online Resource 3. Preferred Reporting Items for Systematic Reviews and Meta-Analyses (PRISMA) checklist…………12

Table S1. PRISMA checklist………………………………………………………………………………………12

Online Resource 4. Characteristics of included studies…………………………………………………………………14

Table S2. General study characteristics……………………………………………………………………………14

Table S3. Patients and ventilation settings…………………………………………………………………………16

Table S4. Outcomes in the higher PEEP group……………………………………………………………………18

Table S5. Outcomes in the lower PEEP group………………………………………………………………………20

Online Resource 5. Risk of bias assessment……………………………………………………………………………22

Table S6. Risk of bias summary……………………………………………………………………………………22

Fig. S1. Risk of bias summary………………………………………………………………………………………23

Fig. S2. Risk of bias graph……………………………………………………………………………………………24

Table S7. Risk of bias for each study with signaling questions……………………………………………………25

Online Resource 6. Primary and secondary outcomes……………………………………………………………………75

Table S8. Summary of main meta-analysis…………………………………………………………………………75

Forest plots of other secondary outcomes ……………………………………………………………………………76

Online Resource 7. Funnel plots…………………………………………………………………………………………92

Online Resource 8. Sensitivity analyses………………………………………………………………………………112

Sensitivity analyses: timing of measurement………………………………………………………………………112

Sensitivity analyses: odds ratio……115
Sensitivity analyses: without studies at high risk of bias……116
Forest plots of sensitivity analyses……118
Online Resource 9. Subgroup analyses……170
Subgroup analyses: medical vs. surgical patients……170
Subgroup analyses: zero end-expiratory pressure vs. lower PEEP……172
Subgroup analyses: tidal volume > 8 mL/kg vs. tidal volume < 8 mL/kg. ……176
Subgroup analyses: studies published before 2000 vs. studies published after 2000……179
Forest plots of subgroup analyses……182
Online Resource 10. Meta-regression……242
Table S9. Meta-regression for the association between PEEP level and primary outcome and main secondary outcomes using tidal volume as covariate……242
Online Resource 11. Trial sequential analysis……243
References……244

Online Resource 1. Methods

Participants, Interventions, Comparisons, Outcomes, and Study design question

- Participants: adult patients in the intensive care unit (ICU) undergoing invasive mechanical ventilation
- Interventions: higher positive end-expiratory pressure (PEEP)
- Comparisons: lower PEEP
- Outcomes:
  - Primary outcome: hospital mortality
  - Secondary outcomes:
    - Arterial partial pressure of oxygen to fraction of inspired oxygen ratio
    - Alveolar-arterial oxygen pressure difference
    - Hypoxemia
    - Respiratory system compliance
    - Atelectasis
    - Barotrauma
    - Development of acute respiratory distress syndrome (ARDS)
    - Ventilator-associated pneumonia
    - Cardiac index
    - Central venous pressure
    - Hypotension
    - Postoperative bleeding and transfusion
    - Duration of ventilation
    - ICU length of stay
    - Hospital length of stay
    - ICU mortality
    - 28-day mortality
- Study design: randomized controlled trials.

Database Search Strategies

MEDLINE search strategy (744 citations):

Ovid MEDLINE(R) 1946 to June Week 3 2021

Ovid MEDLINE(R) Epub Ahead of Print and In-Process & Other Non-Indexed Citations June 16, 2021

1. positive end expiratory pressure.mp,kw.
2. positive end-expiratory pressure.mp,kw.
3. PEEP.mp,kw.
4. or/1-3 [PEEP]
5. randomized controlled trial.pt.
6. RCT.mp,kw.
7. random*.mp,kw.
8. or/5-7 [RCT]
9. 4 and 8 [PEEP + RCT]

EMBASE search strategy (3635 citations): see MEDLINE search strategy

Scopus search strategy (5511 citations):

((POSITIVE END-EXPIRATORY PRESSURE) OR PEEP) AND ((RANDOMIZED CONTROLLED TRIAL*) OR RCT)

Cochrane Central Register of Controlled Trials search strategy (2942 citations): see Scopus search strategy

CINAHL search strategy (404 citations): see Scopus search strategy

Web of Science search strategy (672 citations): see Scopus search strategy

OpenGrey search strategy (22 citations): (POSITIVE END-EXPIRATORY PRESSURE) OR PEEP

Risk of Bias Assessment

The risk of bias (ROB) of the included studies was independently assessed by three authors (TP, PP, FZ) according to the revised Cochrane ROB tool for randomized trials (RoB 2) [S1]. RoB2 examines 5 domains of bias: 1) randomization process; 2) deviations from intended interventions; 3) missing outcome data; 4) measurement of the outcome; and 5) selection of the reported results. The overall RoB judgment for each domain was attributed according to the criteria specified in the RoB 2 tool. The study was considered at low risk of bias when it was judged to be at low risk of bias for all domains; the study was considered to raise some concern when it was judged to raise some concerns in at least one domain, but not to be at high risk of bias for any domain; the study was considered at high risk of bias when it was judged to be at high risk of bias in at least one domain or it was judged to have some concerns for multiple domains in a way that substantially lowered confidence in the result. The risk of bias of individual studies was examined at the study level. All disagreements were resolved by discussion or referral to a third author (LP) if necessary.

Online Resource 2. Included Studies and Major Exclusions

Studies included in both the qualitative and quantitative review

1. Good JT Jr, Wolz JF, Anderson JT, Dreisin RB, Petty TL. The routine use of positive end-expiratory pressure after open heart surgery. Chest. 1979;76:397–400.
2. Zurick AM, Urzua J, Ghattas M, Cosgrove DM, Estafanous FG, Greenstreet R. Failure of positive end-expiratory pressure to decrease postoperative bleeding after cardiac surgery. Ann Thorac Surg. 1982;34:608–11.
3. Marvel SL, Elliott CG, Tocino I, Greenway LW, Metcalf SM, Chapman RH. Positive end-expiratory pressure following coronary artery bypass graft- ing. Chest. 1986;90:537–41.
4. Michalopoulos A, Anthi A, Rellos K, Geroulanos S. Effects of positive end-expiratory pressure (PEEP) in cardiac surgery patients. Respir Med. 1998;92:858–62.
5. Collier B, Kolff J, Devineni R, Gonzalez LS. Prophylactic positive end-expiratory pressure and reduction of postoperative blood loss in open-heart surgery. Ann Thorac Surg. 2002;74(4):1191-1194.
6. Dyhr T, Laursen N, Larsson A. Effects of lung recruitment maneuver and positive end-expiratory pressure on lung volume, respiratory mechanics and alveolar gas mixing in patients ventilated after cardiac surgery. Acta Anaesthesiol Scand. 2002;46:717–25.
7. Koutsoukou A, Perraki H, Raftopoulou A, et al. Respiratory mechanics in brain-damaged patients. Intensive Care Med. 2006;32(12):1947-1954.
8. Holland A, Thuemer O, Schelenz C, van Hout N, Sakka SG. Positive end-expiratory pressure does not affect indocyanine green plasma disap- pearance rate or gastric mucosal perfusion after cardiac surgery. Eur J Anaesthesiol. 2007;24:141–7.
9. Korovesi I, Papadomichelakis E, Orfanos SE, et al. Exhaled breath condensate in mechanically ventilated brain-injured patients with no lung injury or sepsis. Anesthesiology. 2011;114(5):1118-1129.
10. Korovesi I, Kotanidou A, Papadomichelakis E, et al. Exhaled nitric oxide and carbon monoxide in mechanically ventilated brain-injured patients. J Breath Res. 2016;10(1):017107.
11. Lago Borges D, Nina VJ, Costa Mde A, Baldez TE, Santos NP, Lima IM, et al. Effects of different PEEP levels on respiratory mechanics and oxygena- tion after coronary artery bypass grafting. Rev Bras Cir Cardiovasc. 2013;28:380–5.

12. Lago Borges D, da Silva José, Nina V, Pereira Baldez TE, de Albuquerque Gonçalves Costa M, Pereira dos Santos N, Mendes Limaf I, et al. Effects of positive end-expiratory pressure on mechanical ventilation duration after coronary artery bypass grafting: a randomized clinical trial. Ann Thorac Cardiovasc Surg. 2014;20(Suppl):773–7.
13. Feeley TW, Saumarez R, Klick JM, McNabb TG, Skillman JJ. Positive end- expiratory pressure in weaning patients from controlled ventilation. A prospective randomised trial. Lancet. 1975;2:725–9.
14. Weigelt JA, Mitchell RA, Snyder WH 3rd. Early positive end-expiratory pressure in the adult respiratory distress syndrome. Arch Surg. 1979;114:497–501.
15. Pepe PE, Hudson LD, Carrico CJ. Early application of positive end- expiratory pressure in patients at risk for the adult respiratory-distress syndrome. N Engl J Med. 1984;311:281–6.
16. Nelson LD, Civetta JM, Hudson-Civetta J. Titrating positive end-expiratory pressure therapy in patients with early, moderate arterial hypoxemia. Crit Care Med. 1987;15:14–9.
17. Manzano F, Fernández-Mondéjar E, Colmenero M, Poyatos ME, Rivera R, Machado J, et al. Positive-end expiratory pressure reduces incidence of ventilator-associated pneumonia in nonhypoxemic patients. Crit Care Med. 2008;36:2225–31.
18. Lesur O, Remillard MA, St-Pierre C, Falardeau S. Prophylactic positive end- expiratory pressure and postintubation hemodynamics: an interven- tional, randomized study. Can Respir J. 2010;17:e45–50.
19. Ma C, Liang D, Zheng F. Effect of high positive end-expiratory pressure for mechanical ventilation in the treatment of neurological pulmonary edema. Zhonghua Wei Zhong Bing Ji Jiu Yi Xue. 2014;26:339–42.
20. Algera AG, Pisani L, Serpa Neto A, et al; Writing Committee and Steering Committee for the RELAx Collaborative Group. Effect of a lower vs. higher positive end-expiratory pressure strategy on ventilator-free days in ICU patients without ARDS: a randomized clinical trial. JAMA. 2020;324(24):2509.

Studies included in the qualitative review only

1. Murphy DA, Finlayson DC, Craver JM, Jones EL, Kopel M, Tobia V, et al. Effect of positive end-expiratory pressure on excessive mediastinal bleed- ing after cardiac operations. A controlled study. J Thorac Cardiovasc Surg. 1983;85:864–9.
2. Vigil AR, Clevenger FW. The effects of positive end-expiratory pressure of intrapulmonary shunt and ventilatory deadspace in nonhypoxic trauma patients. J Trauma. 1996;40:618–22.

Studies on non-invasive mechanical ventilation

1. Carroll GC, Tuman KJ, Braverman B, et al. Minimal positive end-expiratory pressure (PEEP) may be "best PEEP". Chest. 1988;93:1020–1025.
2. Choo-Kang YFJ, Parker SS, Grant IWB. Response of asthmatics to isoprenaline and salbutamol aerosols administered by intermittent positive-pressure ventilation. BMJ. 1970;4(5733):465-468.

Studies with less than 2 levels of peep and/or change in other ventilatory settings and/or crossover studies

1. Auler Jr. JOC, Carmona MJC, Barbas CV, Saldiva PHN, Malbouisson LMS. The effects of positive end-expiratory pressure on respiratory system mechanics and hemodynamics in postoperative cardiac surgery patients. Braz J Med Biol Res. 2000;33(1):31-42.
2. Baxter WD. An evaluation of intermittent positive pressure breathing in the prevention of postoperative pulmonary complications. Arch Surg. 1969;98(6):795.
3. Cao F, Chen R, Liu X, He R. Effect of positive end-expiratory pressure on the pressure gradient of venous return in hypovolemic patients under mechanical ventilation. Zhongguo Wei Zhong Bing Ji Jiu Yi Xue. 2009;21(10):583-586.
4. Cujec B, Polasek P, Mayers I, Johnson D. Positive end-expiratory pressure increases the right-to-left shunt in mechanically ventilated patients with patent foramen ovale. Ann Intern Med. 1993;119:887–894.
5. Saner FH, Olde Damink SWM, Pavlaković G, et al. Positive end-expiratory pressure induces liver congestion in living donor liver transplant patients: myth or fact. Transplantation. 2008;85(12):1863-1866.
6. Saner FH, Pavlakovic G, Gu Y, et al. Effects of positive end-expiratory pressure on systemic haemodynamics, with special interest to central venous and common iliac venous pressure in liver transplanted patients. Eur J Anaesthesiol. 2006;23(9):766-771.
7. Saner FH, Olde Damink SWM, Pavlaković G, et al. How far can we go with positive end-expiratory pressure (PEEP) in liver transplant patients? J Clin Anesth. 2010;22(2):104-109.
8. Celebi S, Köner O, Menda F, Korkut K, Suzer K, Cakar N. The pulmonary and hemodynamic effects of two different recruitment maneuvers after cardiac surgery. Anesth Analg. 2007;104:384–90.
9. Huang C-C, Tsai Y-H, Lin M-C, Tsao TCY, Hsu K-H. Gastric intramucosal PCO2 and pH variability in ventilated critically ill patients. Crit Care Med. 2001;29(1):88-95.

10. Kong W, Wang C, Yang Y, Huang K, Jiang C. Effects of extrinsic positive end-expiratory pressure on work of breathing in patients with chronic obstructive pulmonary disease. Chin Med J (Engl). 2001;114(8):791-794.
11. Kumar A, Pontoppidan H, Baratz RA, Laver MB. Inappropriate response to increased plasma ADH during mechanical ventilation in acute respiratory failure. Anesthesiology. 1974;40(3):215-221.
12. Mascia L, Grasso S, Fiore T, Bruno F, Berardino M, Ducati A. Cerebro-pulmonary interactions during the application of low levels of positive end-expiratory pressure. Intensive Care Med. 2005;31(3):373-379.
13. Mauri T, Eronia N, Turrini C, et al. Bedside assessment of the effects of positive end-expiratory pressure on lung inflation and recruitment by the helium dilution technique and electrical impedance tomography. Intensive Care Med. 2016;42(10):1576-1587.
14. Nikki P, Räsänen J, Tahvanainen J, Mäkeläinen A. Ventilatory pattern in respiratory failure arising from acute myocardial infarction. I. Respiratory and hemodynamic effects of IMV4 vs IPPV12 and PEEP0 vs PEEP10. Crit Care Med. 1982;10(2):75-78.
15. Reis Miranda D, Gommers D, Struijs A, et al. The open lung concept: effects on right ventricular afterload after cardiac surgery. Br J Anaesth. 2004;93(3):327-332.
16. Reissmann HK, Ranieri VM, Goldberg P, Gottfried SB. Continuous positive airway pressure facilitates spontaneous breathing in weaning chronic obstructive pulmonary disease patients by improving breathing pattern and gas exchange. Intensive Care Med. 2000;26(12):1764-1772.
17. Tobin MJ, Jenouri G, Birch S, et al. Effect of positive end-expiratory pressure on breathing patterns of normal subjects and intubated patients with respiratory failure. Crit Care Med. 1983;11(11):859-867.
18. Torelli L, Zoccali G, Casarin M, Dalla Zuanna F, Lieta E, Conti G. Comparative evaluation of the haemodynamic effects of continuous negative external pressure (CNEP) and positive end-expiratory pressure (PEEP) in mechanically ventilated trauma patients. Intensive Care Med. 1995;21(1):67-70.
19. Tsai Y-H, Lin M-C, Hsieh M-J, et al. Spontaneous variability of arterial oxygenation in critically ill mechanically ventilated patients. Intensive Care Med. 1999;25(1):37-43.
20. Vitacca M, Bianchi L, Zanotti E, et al. Assessment of physiologic variables and subjective comfort under different levels of pressure support ventilation. Chest. 2004;126(3):851-859.

Non-RCT study

1. Dongelmans DA, Hemmes SN, Kudoga AC, Veelo DP, Binnekade JM, Schultz MJ. Positive end-expiratory pressure following coronary artery bypass grafting. Minerva Anestesiol. 2012;78:790–800.

Studies not on ICU patients

1. Calzia E, Lindner KH, Stahl W, Martin A, Radermacher P, Georgieff M. Work of breathing, inspiratory flow response, and expiratory resistance during continuous positive airway pressure with the ventilators EVITA-2, EVITA-4 and SV 300. Intensive Care Med. 1998;24(9):931-938.
2. Claxton BA, Morgan P, Mckeague H, Mulpur A, Berridge J. Alveolar recruitment strategy improves arterial oxygenation after cardiopulmonary bypass: Arterial oxygenation after cardiopulmonary bypass. Anaesthesia. 2003;58(2):111-116.
3. Oliveira CC, Carrascosa CR, Borghi-Silva A, et al. Influence of respiratory pressure support on hemodynamics and exercise tolerance in patients with COPD. Eur J Appl Physiol. 2010;109(4):681-689.

Study on ARDS patients

1. Borelli M, Fumagalli R, Bernasconi F, Cereda M, Gattinoni L, Pesenti A. Relief of hypoxemia contributes to a reduction in cardiac index related to the use of positive end-expiratory pressure. Intensive Care Med. 1996;22(5):382-386.

Conference proceeding

1. Schmidt GB, O'Neill WW, Kotb K, Hwang KK, Bennett EJ, Bombeck CT. Continuous positive airway pressure in the prophylaxis of the adult respiratory distress syndrome. Surg Gynecol Obstet. 1976;143:613–8.

Online Resource 3. Preferred Reporting Items for Systematic Reviews and Meta-Analyses (PRISMA) checklist

Table S1. PRISMA checklist			
**Section/topic**	**#**	**Checklist item**	**Reported on page #**
**Title**			
Title	1	Identify the report as a systematic review, meta-analysis, or both.	1
**Abstract**			
Structured summary	2	Provide a structured summary including, as applicable: background; objectives; data sources; study eligibility criteria, participants, and interventions; study appraisal and synthesis methods; results; limitations; conclusions and implications of key findings; systematic review registration number.	1, 2
**Introduction**			
Rationale	3	Describe the rationale for the review in the context of what is already known.	2, 3
Objectives	4	Provide an explicit statement of questions being addressed with reference to participants, interventions, comparisons, outcomes, and study design (PICOS).	3, Online Resource 1
**Methods**			
Protocol and registration	5	Indicate if a review protocol exists, if and where it can be accessed (e.g., Web address), and, if available, provide registration information including registration number.	5
Eligibility criteria	6	Specify study characteristics (e.g., PICOS, length of follow-up) and report characteristics (e.g., years considered, language, publication status) used as criteria for eligibility, giving rationale.	3, Online Resource 1
Information sources	7	Describe all information sources (e.g., databases with dates of coverage, contact with study authors to identify additional studies) in the search and date last searched.	3, Online Resource 1
Search	8	Present full electronic search strategy for at least one database, including any limits used, such that it could be repeated.	Online Resource 1
Study selection	9	State the process for selecting studies (i.e., screening, eligibility, included in systematic review, and, if applicable, included in the meta-analysis).	4
Data collection process	10	Describe method of data extraction from reports (e.g., piloted forms, independently, in duplicate) and any processes for obtaining and confirming data from investigators.	4

Table S1 (continued)			
Data items	11	List and define all variables for which data were sought (e.g., PICOS, funding sources) and any assumptions and simplifications made.	3, Online Resource 1
Risk of bias in individual studies	12	Describe methods used for assessing risk of bias of individual studies (including specification of whether this was done at the study or outcome level), and how this information is to be used in any data synthesis.	4, Online Resource 1
Summary measures	13	State the principal summary measures (e.g., risk ratio, difference in means).	4
Synthesis of results	14	Describe the methods of handling data and combining results of studies, if done, including measures of consistency (e.g., I^2) for each meta-analysis.	4, 5

Online Resource 4. Characteristics of Included Studies

Table S2. General study characteristics				
**First author**	**Year**	**Type of patients**	**N**	**Main findings**
Collier	2002	Post-cardiac surgery	84	Increased postoperative bleeding with higher PEEP
Dyhr	2002	Post-cardiac surgery	15	Increased oxygenation and end-expiratory lung volume and decreased atelectasis with higher PEEP
Feeley	1975	ARF during weaning from MV	25	Improvement in vital capacity and maximum inspiratory force and less increase in intra-pulmonary shunt with higher PEEP
Good	1979	Post-cardiac surgery	24	No differences regarding atelectasis and oxygenation
Holland	2007	Post-cardiac surgery	28	No differences regarding cardiac function, liver function, and gastric mucosal perfusion.
Korovesi	2011-2016[a]	Brain injury with MV < 24 hours	27[a]	Korovesi 2011: no differences regarding exhaled breath condensate markers, with the exception of interleukin-10, and lower systemic inflammatory indices with higher PEEP. Korovesi 2016: no differences regarding exhaled nitric oxide trend with significant decrease in exhaled carbon monoxide in the ZEEP group.
Koutsoukou	2006	Severe brain damage	21	No differences regarding exhaled NO trend and significant decrease in exhaled CO in the ZEEP group.
Lago Borges	2013-2014[b]	Post-cardiac surgery	136[b]	Lago Borges 2013: higher compliance and oxygenation with higher PEEP. Lago Borges 2014: shorter duration of ventilation with higher PEEP[c].
Lesur	2010	ARF	63	No differences regarding incidence of hypotension, duration of ventilation and mortality[d].
Ma	2014	Brain injury with neurological pulmonary edema	120	Lower blood pressure, higher oxygenation and lower 28-day mortality with higher PEEP
Manzano	2008	Nonhypoxemic patients (PaO_2/FiO_2 > 250)	127	Lower incidence of VAP and hypoxemia with higher PEEP; no differences regarding development of ARDS, barotrauma, or atelectasis, and hospital mortality.
Marvel	1986	Post-cardiac surgery	44[e]	Better oxygenation with higher PEEP; no differences regarding atelectasis and hospital length of stay.
Michalopoulos	1998	Post-cardiac surgery	67[f]	No differences regarding oxygenation, cardiac index, incidence of pneumothorax and atelectasis, duration of ventilation, and mortality
Murphy	1983	Post-cardiac surgery	139	No differences regarding blood loss and blood products or fluids administration
Nelson	1987	Hypoxemic patients (PaO_2/FiO_2 144-244)	38	No differences regarding barotrauma, duration of ventilation, length of stay, and mortality
Pepe	1984	ARF patients at risk of ARDS	92	No differences regarding incidence of ARDS, barotrauma, atelectasis, hypotension, duration of ventilation, length of stay, and mortality
Relax	2020	ARF patients expected not to be extubated within 24 hours	969	No differences regarding 28-day ventilator free days, duration of ventilation, incidence of ARDS, VAP, pneumothorax, atelectasis, length of stay, and mortality.
Vigil	1996	Nonhypoxic patients after trauma	44	No differences regarding shunt, dead space volume, and oxygenation after correction for baseline values
Weigelt	1979	ARF patients at risk of ARDS	79	Lower incidence of ARDS and pulmonary mortality and higher incidence of pulmonary dysfunction with higher PEEP

Table S2 (continued)				
**First author**	**Year**	**Type of patients**	**N**	**Main findings**
Zurick	1982	Post-cardiac surgery	83	No differences regarding postoperative blood loss, need for reexploration for bleeding, and blood requirement

Abbreviations: N, total number of patients; PEEP, positive end-expiratory pressure; ARF, acute respiratory failure; MV, mechanical ventilation; ZEEP, zero end-expiratory pressure; ICU, intensive care unit; PaO_2/FiO_2, arterial partial pressure of oxygen to fraction of inspired oxygen ratio; VAP, ventilator-associated pneumonia; ARDS, acute respiratory distress syndrome.

Twenty-two randomized controlled trials (2225 patients), which compared higher PEEP (1007 patients) to lower PEEP (991 patients), were included. The Murphy study did not report the number of patients that were randomized to the two groups and some study groups of the Lago Borges, Marvel, and Michalopoulos studies were excluded (see below); this explains why the total number of patients overall included in the studies does not match the sum of the patients in the two groups.

[a]The same patient population was included in Korovesi 2011 and Korovesi 2016.

[b]The same patient population was included in Lago Borges 2013 and Lago Borges 2014. One group of 47 patients with intermediate level of PEEP (8 cmH2O) was not included in this systematic review and meta-analysis.

[c]Only patients extubated within 12 h after ICU admission were considered by the authors of the original article.

[d]PEEP was maintained for the first 90 minutes after intubation only.

[e]One group of 17 patients, who exhaled to ambient pressure, was not included in this systematic review and meta-analysis.

[f]One group of 24 patients with intermediate level of PEEP (5 cmH2O) was not included in this systematic review and meta-analysis.

Table S3. Patients and ventilation settings														
	**Higher PEEP**							**Lower PEEP**						
**First author**	**N**	**Age** (years)	**Female gender** (N [%])	**PEEP titration**	**PEEP level** (cmH_2O)	**Tidal volume** (mL/kg)[a]	**Ventilatory mode**	**N**	**Age** (years)	**Female gender** (N [%])	**PEEP titration**	**PEEP level** (cmH_2O)	**Tidal volume** (mL/kg)[a]	**Ventilatory mode**
Collier	40	67 ± 11	9 (22)	Arbitrarily	10	10	SIMV	44	65 ± 8	16 (36)	Arbitrarily	5	10	SIMV
Dyhr	7	61 ± 24	0	1 cmH2O above lower inflection point	15	6	VCV[b]	8	63 ± 22	3 (37)	Arbitrarily	0	6	VCV[b]
Feeley	12	59 ± 22	7 (58)	Arbitrarily	5	10	VCV	13	64 ± 10	7 (54)	Arbitrarily	0	10	VCV
Good	10	51 ± 2	n.a.	Maximum respiratory system compliance	6.3	10-12	VCV	14	57 ± 2	n.a.	Arbitrarily	0	10-12	VCV
Holland	14	63 ± 7	1 (7)	Arbitrarily	10	6-8	PCV	14	68 ± 11	6 (43)	Arbitrarily	5	6-8	PCV
Korovesi[c]	15	33 ± 29	3 (20)	Arbitrarily	8	8	VCV	12	23 ± 5	2 (17)	Arbitrarily	0	8	VCV
Koutsoukou	11	42 ± 19	2 (18)	Arbitrarily	8	8	VCV	10	40 ± 12	3 (30)	Arbitrarily	0	8	VCV
Lago Borges[d]	45	n.a.	10 (22)	Arbitrarily	10	6-8	VCV	44	n.a.	15 (34)	Arbitrarily	5	6-8	VCV
Lesur	30	65 ± 14	13 (43)	Arbitrarily	5	8	VCV/PCV	33	64 ± 18	12 (36)	Arbitrarily	0	7	VCV/PCV
Ma	60	n.a.	n.a.	Arbitrarily	11-30	6-8	n.a.	60	n.a.	n.a.	Arbitrarily	3-10	6-8	n.a.
Manzano	64	44 ± 18	17 (26)	Level of abdomen relative to level of chest	5-8	8	n.a.	63	47 ± 19	20 (32)	Arbitrarily	0	8	n.a.
Marvel[e]	12	56 ± 3	n.a.	Arbitrarily	10	12	VCV	15	61 ± 3	n.a.	Arbitrarily	5	12	VCV
Michalopoulos[f]	21	62 ± 7	5 (24)	Arbitrarily	10	n.a.	ACV	22	61 ± 6	4 (18)	Arbitrarily	0	n.a.	ACV
Murphy	n.a.	n.a.	n.a.	Arbitrarily	10	n.a.	n.a.	n.a.	n.a.	n.a.	Arbitrarily	0	n.a.	n.a.
Nelson	20	53 ± 17	n.a.	Incremental until $PaO_2/FiO_2 > 300$ or shunt < 0.2	15	n.a.	IMV	18	55 ± 20	n.a.	Incremental until $PaO_2 > 65$ mmHg	8	n.a.	IMV
Pepe	44	46 ± 19	14 (32)	Arbitrarily	8	12	VCV	48	42 ± 19	12 (25)	Arbitrarily	0	12	VCV

**Table S3 (continued)**														
	**Higher PEEP**							**Lower PEEP**						
**First author**	**N**	**Age** (years)	**Female gender** (N [%])	**PEEP titration**	**PEEP level** (cmH_2O)	**Tidal volume** (mL/kg)[a]	**Ventilatory mode**	**N**	**Age** (years)	**Female gender** (N [%])	**PEEP titration**	**PEEP level** (cmH_2O)	**Tidal volume** (mL/kg)[a]	**Ventilatory mode**
Relax	493	66 ± 13	182 (37)	Clinical practice from The Netherlands	8	7	VCV/PCV/PSV	476	65 ± 13	164 (34)	Decremental until SpO_2 > 92% or PaO_2 > 60 mmHg	2	7	VCV/PCV/PSV
Vigil	23	n.a.	n.a.	Arbitrarily	5	12	n.a.	21	n.a.	n.a.	Arbitrarily	0	12	n.a.
Weigelt	45	Median 45	15 (33)	Arbitrarily	5	15	n.a.	34	Median 45	7 (21)	Arbitrarily	0	15	n.a.
Zurich	41	56 ± 8	4 (10)	Arbitrarily	10	n.a.	VCV	42	57 ± 8	8 (19)	Arbitrarily	0	n.a.	VCV/PVC

Data are reported as mean (± standard deviation) or number (%), as appropriate, unless otherwise specified.
Abbreviations: N, total number of patients; PEEP, positive end-expiratory pressure; SIMV, synchronized intermittent mandatory ventilation; VCV, volume-controlled ventilation; n.a., not available; PCV, pressure-controlled ventilation; ACV, assist control ventilation; PaO_2/FiO_2, arterial partial pressure of oxygen to fraction of inspired oxygen ratio; PaO_2, arterial partial pressure of oxygen; IMV, intermitted mandatory ventilation; SpO_2, pulse oximetry–measured oxygen saturation.
Twenty-two randomized controlled trials (2225 patients), which compared higher PEEP (1007 patients) to lower PEEP (991 patients), were included. The Murphy study did not report the number of patients that were randomized to the two groups and some study groups of the Lago Borges, Marvel, and Michalopoulos studies were excluded (see below); this explains why the total number of patients overall included in the studies does not match the sum of the patients in the two groups.
[a]Many authors did not specify whether the tidal volume was based on ideal body weight or actual body weight.
[b]This study was the only study including recruitment maneuvers in the ventilatory protocol.
[c]The same patient population was included in Korovesi 2011 and Korovesi 2016.
[d]The same patient population was included in Lago Borges 2013 and Lago Borges 2014. One group of 47 patients with intermediate level of PEEP 8 cmH2O was not included in this systematic review and meta-analysis.
[e]One group of 17 patients, who exhaled to ambient pressure, was not included in this systematic review and meta-analysis.
[f]One group of 24 patients with intermediate level of PEEP (5 cmH2O) was not included in this systematic review and meta-analysis.

Table S4. Outcomes in the higher PEEP group

**First author**	**PaO_2/FiO_2** (mmHg)	**A-aDO_2** (mmHg)	**Crs** (mL/cmH$_2$O)	**CI** (L/min/m^2)	**CVP** (mmHg)	**Hypoxemia** (n [%])	**Pneumonia** (n [%])	**Atelectasis** (n [%])	**ARDS** (n [%])	**Bleeding 24 h** (mL)	**PRBC** (units)	**Hypotension** (n [%])	**Barotrauma** (n [%])	**Duration of ventilation**	**Hospital stay (days)**	**ICU stay (days)**	**ICU mortality** (n [%])	**28-day mortality** (n [%])	**Hospital mortality** (n [%])
Collier				3.10 ± 0.86						395 ± 392	0.8 ± 1.4			409 ± 209 min	5.2 ± 1.7				1 (2%)
Dyhr				2.2 ± 0.6	13 ± 11												0 (0%)		0 (0%)
Feeley		Increase of 10 ± 22										0 (0%)		258 ± 217 min			2 (17%)		
Good								9 (90%)				0 (0%)	0 (0%)						
Holland	307 ± 82			3.0 ± 0.6	9 ± 3														
Korovesi[a]	498 ± 75								0 (0%)							17.2 ± 10.1	3 (20%)		
Koutsoukou	409 ± 65	100 ± 41	62 ± 14						0 (0%)										
Lago Borges[b]	328 ± 85	117 ± 33	56 ± 19			19 (42%)								5.1 ± 2.9 hours					
Lesur	293 ± 135				12 ± 1									9.2 ± 8.8 days				9 (30%)	12 (40%)
Ma	196 ± 45																	15 (25%)	
Manzano[c]	359 ± 104					12 (19%)	6 (9%)	12 (19%)	3 (5%)				1 (%)	5.8 ± 6.8 days	19.5 ± 18.2	10.5 ± 9.8			19 (30%)
Marvel[d]		168 ± 10												9.3 ± 0.6 hours	8.8 ± 0.5				
Michalopoulos[e]	315			3				2 (9%)					0 (0%)						0 (0%)
Nelson													1 (5%)	5.3 ± 5.0 days	28 ± 24	6.6 ± 5.0	4 (20%)		5 (25%)
Pepe						1 (2%)	4 (9%)	27 (61%)	11 (25 %)			1 (2%)	19 (43%)						13 (30%)

Table S4 (continued)

First author	PaO_2/FiO_2 (mmHg)	A-aDO_2 (mmHg)	Crs (mL/cmH$_2$O)	CI (L/min/m^2)	CVP (mmHg)	Hypoxemia (n [%])	Pneumonia (n [%])	Atelectasis (n [%])	ARDS (n [%])	Bleeding 24 h (mL)	PRBC (units)	Hypotension (n [%])	Barotrauma (n [%])	Duration of ventilation	Hospital stay (days)	ICU stay (days)	ICU mortality (n [%])	28-day mortality (n [%])	Hospital mortality (n [%])
Relax[f]	248 ± 112					87 (18%)	7 (1%)	15 (3%)	5 (1%)		1.7 ± 0.7		12 (2%)	4.8 ± 6.6 days	19 ± 21	7.2 ± 10.3	185 (38%)	207 (50%)	208 (42%)
Vigil														3.2 days					
Weigelt			42 ± 36						9 (20 %)			1 (2%)	5 (11%)	9.3 ± 13 days		11.7 ± 16.8			16 (35%)
Zurich										542 ± 239	0.33 ± 0.87								

Data are reported as mean (± standard deviation) or number (%), as appropriate, unless otherwise specified. Empty cells are due to not available data; no data were available for the Murphy study.

Abbreviations: PEEP, positive end-expiratory pressure; PaO_2/FiO_2, arterial partial pressure of oxygen to fraction of inspired oxygen ratio; A-aDO_2, alveolar-arterial oxygen pressure difference; Crs, respiratory system compliance; CI, cardiac index; CVP, central venous pressure; ARDS, acute respiratory distress syndrome; bleeding 24 h, bleeding 24 hours after the surgery; PRBC, packed red blood cells; ICU, intensive care unit.

Quantitative variables are expressed as mean ± standard deviations (only mean in Michalopoulos and Vigil), qualitative variables as number (percentage).

[a]The same patient population was included in Korovesi 2011 and Korovesi 2016. Variables included in the table are the variables collected at day 3 in the original study.

[b]The same patient population was included in Lago Borges 2013 and Lago Borges 2014. One group of 47 patients with intermediate level of PEEP 8 cmH2O was not included in this systematic review and meta-analysis.

[c]Variables included in the table are the variables collected at day 2 in the original study.

[d]One group of 17 patients, who exhaled to ambient pressure, was not included in this systematic review and meta-analysis.

[e]One group of 24 patients with intermediate level of PEEP (5 cmH2O) was not included in this systematic review and meta-analysis. Variables included in the table are the variables collected before the extubation in the original study.

[f]Variables included in the table are the variables collected at day 3 in the original study.

Table S5. Outcomes in the lower PEEP group

First author	PaO_2/FiO_2 (mmHg)	$A\text{-}aDO_2$ (mmHg)	Crs (mL/cmH_2O)	CI (L/min/m^2)	CVP (mmHg)	Hypoxemia (n [%])	Pneumonia (n [%])	Atelectasis (n [%])	ARDS (n [%])	Bleeding 24 h (mL)	PRBC (units)	Hypotension (n [%])	Barotrauma (n [%])	Duration of ventilation	Hospital stay (days)	ICU stay (days)	ICU mortality (n [%])	28-day mortality (n [%])	Hospital mortality (n [%])
Collier				3.10 ± 0.64						587 ± 392	1.1 ± 1.6			440 ± 278 min	5.7 ± 2.5				1 (2%)
Dyhr				2.1 ± 1.1	10 ± 7												0 (0%)		0 (0%)
Feeley		Increase of 102 ± 35										0 (0%)		259 ± 149 min			1 (8%)		
Good								12 (86%)				0 (0%)	0 (0%)						
Holland	337 ± 82			2.9 ± 0.6	9 ± 3														
Korovesi[a]	420 ± 73								0 (0%)							14.40 ± 8.44	4 (33%)		
Koutsoukou	437 ± 74	87 ± 40	53 ± 11						1 (10 %)										
Lago Borges[b]	270 ± 90	139 ± 34	47 ± 12			30 (68%)								6.8 ± 3.2 hours					
Lesur	228 ± 67				11 ± 3									9.2 ± 8.5 days				14 (42%)	16 (48%)
Ma	134 ± 22																	39 (65%)	
Manzano[c]	301 ± 84					34 (54%)	16 (25%)	17 (27%)	9 (14 %)				5 (8%)	6.5 ± 6.2 days	26.3 ± 22.0	12.3 ± 11.4			16 (25%)
Marvel[d]		224 ± 12												11.1 ± 1.3 hours	8.9 ± 0.4				
Michalopoulos[e]	325			3.2				2 (9%)					0 (0%)						0 (0%)
Nelson													0 (0%)	3.4 ± 3.0 days	26 ± 24	5.3 ± 3.0	4 (22%)		6 (33%)
Pepe						4 (8%)	6 (12%)	23 (48%)	13 (27 %)			0 (0%)	24 (50%)						18 (37%)

Table S5 (continued)

First author	PaO_2/FiO_2 (mmHg)	$A\text{-}aDO_2$ (mmHg)	Crs (mL/cmH_2O)	CI ($L/min/m^2$)	CVP (mmHg)	Hypoxemia (n [%])	Pneumonia (n [%])	Atelectasis (n [%])	ARDS (n [%])	Bleeding 24 h (mL)	PRBC (units)	Hypotension (n [%])	Barotrauma (n [%])	Duration of ventilation	Hospital stay (days)	ICU stay (days)	ICU mortality (n [%])	28-day mortality (n [%])	Hospital mortality (n [%])
Relax[f]	190 ± 84					98 (21%)	6 (1%)	20 (4%)	13 (3%)		1 ± 0		19 (4%)	5.5 ± 7.4 days	19.9 ± 22.1	8.1 ± 11.5	163 (34%)	183 (38%)	185 (39%)
Vigil														3.6 days					
Weigelt			39 ± 43						18 (53 %)			0 (0%)	4 (12%)	14.0 ± 21.7 days		21.0 ± 32.5			17 (50%)
Zurich										562 ± 261	0.75 ± 1.42								

Data are reported as mean (± standard deviation) or number (%), as appropriate, unless otherwise specified. Empty cells are due to not available data; no data were available for the Murphy study.

Abbreviations: PEEP, positive end-expiratory pressure; PaO_2/FiO_2, arterial partial pressure of oxygen to fraction of inspired oxygen ratio; $A\text{-}aDO_2$, alveolar-arterial oxygen pressure difference; Crs, respiratory system compliance; CI, cardiac index; CVP, central venous pressure; ARDS, acute respiratory distress syndrome; bleeding 24 h, bleeding 24 hours after the surgery; PRBC, packed red blood cells; ICU, intensive care unit.

Quantitative variables are expressed as mean ± standard deviations (only mean in Michalopoulos and Vigil), qualitative variables as number (percentage).

[a]The same patient population was included in Korovesi 2011 and Korovesi 2016. Variables included in the table are the variables collected at day 3 in the original study.

[b]The same patient population was included in Lago Borges 2013 and Lago Borges 2014. One group of 47 patients with intermediate level of PEEP 8 cmH2O was not included in this systematic review and meta-analysis.

[c]Variables included in the table are the variables collected at day 2 in the original study.

[d]One group of 17 patients, who exhaled to ambient pressure, was not included in this systematic review and meta-analysis.

[e]One group of 24 patients with intermediate level of PEEP (5 cmH2O) was not included in this systematic review and meta-analysis. Variables included in the table are the variables collected before the extubation in the original study.

[f]Variables included in the table are the variables collected at day 3 in the original study.

Online Resource 5. Risk of Bias Assessment

**Table S6. Risk of bias summary**						
**Study**	**Randomization process**	**Deviations from intended interventions**	**Missing outcome data**	**Measurement of the outcome**	**Selection of the reported result**	**Overall risk of bias**
Collier 2002	Low	Low	Low	Some concerns	Some concerns	Some concerns
Dyhr 2002	Some concerns	Some concerns	Low	High	Low	High
Feeley 1975	High	Some concerns	Low	High	Some concerns	High
Good 1979	High	Some concerns	Low	High	Some concerns	High
Holland 2007	Some concerns	Some concerns	Low	Low	Low	Some concerns
Korovesi 2011	Some concerns	Some concerns	Low	Some concerns	Low	Some concerns
Korovesi 2016	High	Some concerns	Low	Some concerns	Low	High
Koutsoukou 2006	High	Some concerns	Low	Some concerns	Some concerns	High
Lago Borges 2013	High	Some concerns	Low	High	High	High
Lago Borges 2014	High	Some concerns	Low	High	High	High
Lesur 2010	Low	Low	Low	Some concerns	Low	Some concerns
Ma 2014	Some concerns	Low	Low	Some concerns	Some concerns	Some concerns
Manzano 2008	Low	Some concerns	Low	Some concerns	Low	Some concerns
Marvel 1986	Some concerns	Some concerns	Low	High	Low	High
Michalopoulos 1998	High	Some concerns	Low	High	Low	High
Murphy 1983	High	High	High	High	Some concerns	High
Nelson 1987	High	Some concerns	Low	High	Low	High
Pepe 1984	Some concerns	Some concerns	Low	High	Low	High
Relax 2020	Low	Low	Low	Some concerns	Low	Some concerns
Vigil 1996	Some concerns	High	Low	High	Low	High
Weigelt 1979	High	Some concerns	Low	High	Some concerns	High
Zurich 1982	High	Some concerns	Low	High	Low	High

Fig. S1. Risk of bias summary

A summary figure of review authors' judgements for each risk of bias item for each study.

Study	D1	D2	D3	D4	D5	Overall
Collier 2002	+	+	+	!	!	!
Dyhr 2002	!	!	+	-	+	-
Feeley 1975	-	!	+	-	!	-
Good 1979	-	!	+	-	!	-
Holland 2007	!	!	+	+	+	!
Korovesi 2011	!	!	+	!	+	!
Korovesi 2016	-	!	+	!	+	-
Koutsoukou 2006	-	!	+	!	!	-
Lago Borges 2013	-	!	+	-	-	-
Lago Borges 2014	-	!	+	-	-	-
Lesur 2010	+	+	+	!	+	!
Ma 2014	!	+	+	!	!	!
Manzano 2008	+	!	+	!	+	!
Marvel 1986	!	!	+	-	+	-
Michalopoulos 1998	-	!	+	-	+	-
Murphy 1983	-	-	-	-	!	-
Nelson 1987	-	!	+	-	+	-
Pepe 1984	!	!	+	-	+	-
Relax 2020	+	+	+	!	+	!
Vigil 1996	!	-	+	-	+	-
Weigelt 1979	-	!	+	-	!	-
Zurich 1982	-	!	+	-	+	-

+ Low risk
! Some concerns
- High risk

D1 Randomisation process
D2 Deviations from the intended interventions
D3 Missing outcome data
D4 Measurement of the outcome
D5 Selection of the reported result

Fig. S2. Risk of bias graph

A plot of the distribution of review authors' judgements across studies for each risk of bias item.

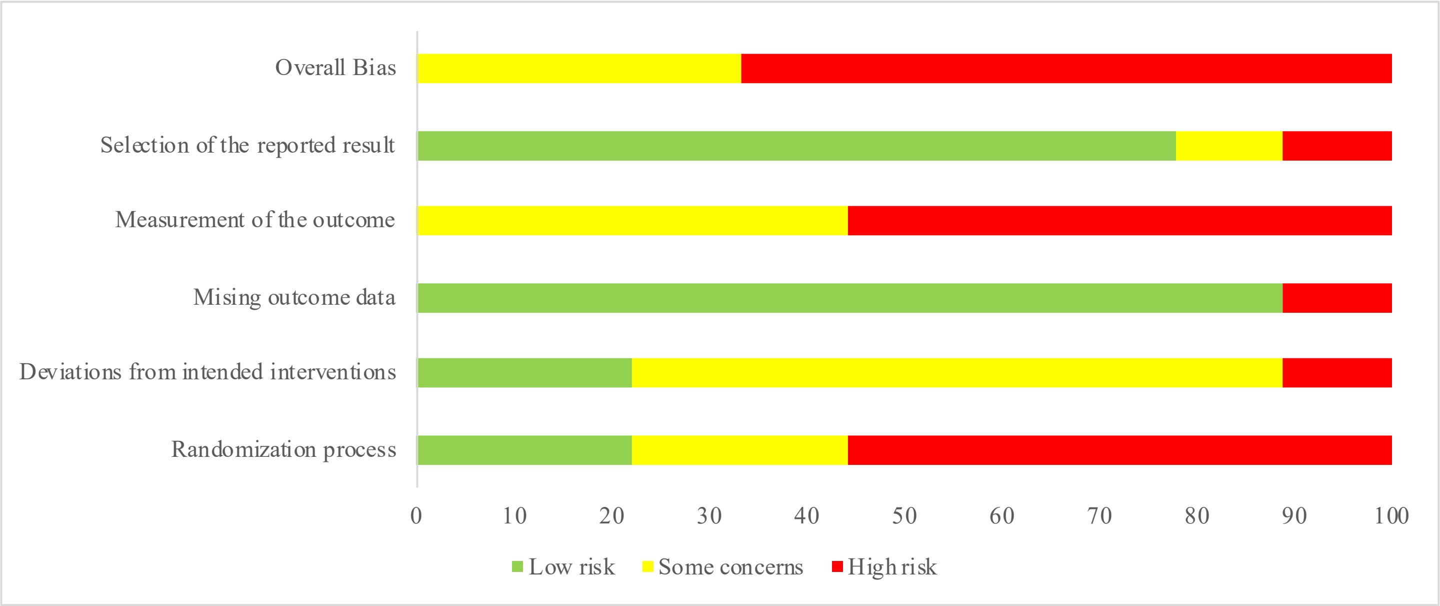

**Table S7. Risk of bias for each study with signaling questions**			
**Study**	Collier 2002		
**Domain**	**Signalling question**	**Response**	**Comments**
**Bias arising from the randomization process**	1.1 Was the allocation sequence random?	Y	"The study design was a prospective, randomized clinical trial. Consecutive patients were randomized to either a PEEP of 10 cm of H_2O (experimental group) or a PEEP of 5 cm of H_2O (control group). Sealed envelopes arranged in a computer-generated random order were opened sequentially to determine the patients' treatment."
	1.2 Was the allocation sequence concealed until participants were enrolled and assigned to interventions?	PY	
	1.3 Did baseline differences between intervention groups suggest a problem with the randomization process?	N	
	**Risk of bias judgement**	**Low**	
**Bias due to deviations from intended interventions**	2.1.Were participants aware of their assigned intervention during the trial?	PN	
	2.2.Were carers and people delivering the interventions aware of participants' assigned intervention during the trial?	NI	
	2.3. If Y/PY/NI to 2.1 or 2.2: Were there deviations from the intended intervention that arose because of the experimental context?	N	
	2.4 If Y/PY to 2.3: Were these deviations likely to have affected the outcome?	NA	
	2.5. If Y/PY/NI to 2.4: Were these deviations from intended intervention balanced between groups?	NA	
	2.6 Was an appropriate analysis used to estimate the effect of assignment to intervention?	Y	

	2.7 If N/PN/NI to 2.6: Was there potential for a substantial impact (on the result) of the failure to analyse participants in the group to which they were randomized?	NA	
	**Risk of bias judgement**	**Low**	
**Bias due to missing outcome data**	3.1 Were data for this outcome available for all, or nearly all, participants randomized?	Y	
	3.2 If N/PN/NI to 3.1: Is there evidence that result was not biased by missing outcome data?	NA	
	3.3 If N/PN to 3.2: Could missingness in the outcome depend on its true value?	NA	
	3.4 If Y/PY/NI to 3.3: Is it likely that missingness in the outcome depended on its true value?	NA	
	**Risk of bias judgement**	**Low**	
**Bias in measurement of the outcome**	4.1 Was the method of measuring the outcome inappropriate?	N	
	4.2 Could measurement or ascertainment of the outcome have differed between intervention groups?	N	
	4.3 Were outcome assessors aware of the intervention received by study participants?	NI	
	4.4 If Y/PY/NI to 4.3: Could assessment of the outcome have been influenced by knowledge of intervention received?	NI	
	4.5 If Y/PY/NI to 4.4: Is it likely that assessment of the outcome was influenced by knowledge of intervention received?	PN	
	**Risk of bias judgement**	**Some concerns**	
**Bias in selection of the reported result**	5.1 Were the data that produced this result analysed in accordance with a pre-specified analysis plan that was finalized before unblinded outcome data were available for analysis?	NI	
	5.2 ... multiple eligible outcome measurements (e.g. scales, definitions, time points) within the outcome domain?	PN	

	5.3 ... multiple eligible analyses of the data?	PN	
	**Risk of bias judgement**	**Some concerns**	
**Overall risk of bias**	**Risk of bias judgement**	**Some concerns**	

**Study**	Dyhr 2002		
**Domain**	**Signalling question**	**Response**	**Comments**
**Bias arising from the randomization process**	1.1 Was the allocation sequence random?	PY	"At arrival after surgery in the intensive care unit (ICU) the patients were assigned randomly, using the sealed envelope technique, into two groups".
	1.2 Was the allocation sequence concealed until participants were enrolled and assigned to interventions?	NI	
	1.3 Did baseline differences between intervention groups suggest a problem with the randomization process?	N	
	**Risk of bias judgement**	**Some concerns**	
**Bias due to deviations from intended interventions**	2.1.Were participants aware of their assigned intervention during the trial?	PN	
	2.2.Were carers and people delivering the interventions aware of participants' assigned intervention during the trial?	PY	
	2.3. If Y/PY/NI to 2.1 or 2.2: Were there deviations from the intended intervention that arose because of the experimental context?	NI	
	2.4 If Y/PY to 2.3: Were these deviations likely to have affected the outcome?	NA	
	2.5. If Y/PY/NI to 2.4: Were these deviations from intended intervention balanced between groups?	NA	

	2.6 Was an appropriate analysis used to estimate the effect of assignment to intervention?	Y	
	2.7 If N/PN/NI to 2.6: Was there potential for a substantial impact (on the result) of the failure to analyse participants in the group to which they were randomized?	NA	
	**Risk of bias judgement**	**Some concerns**	
**Bias due to missing outcome data**	3.1 Were data for this outcome available for all, or nearly all, participants randomized?	Y	
	3.2 If N/PN/NI to 3.1: Is there evidence that result was not biased by missing outcome data?	NA	
	3.3 If N/PN to 3.2: Could missingness in the outcome depend on its true value?	NA	
	3.4 If Y/PY/NI to 3.3: Is it likely that missingness in the outcome depended on its true value?	NA	
	**Risk of bias judgement**	**Low**	
**Bias in measurement of the outcome**	4.1 Was the method of measuring the outcome inappropriate?	N	
	4.2 Could measurement or ascertainment of the outcome have differed between intervention groups?	PY	
	4.3 Were outcome assessors aware of the intervention received by study participants?	NA	
	4.4 If Y/PY/NI to 4.3: Could assessment of the outcome have been influenced by knowledge of intervention received?	NA	
	4.5 If Y/PY/NI to 4.4: Is it likely that assessment of the outcome was influenced by knowledge of intervention received?	NA	
	**Risk of bias judgement**	**High**	
**Bias in selection of the reported result**	5.1 Were the data that produced this result analysed in accordance with a pre-specified analysis plan that was finalized before unblinded outcome data were available for analysis?	PY	

	5.2 ... multiple eligible outcome measurements (e.g. scales, definitions, time points) within the outcome domain?	PN	
	5.3 ... multiple eligible analyses of the data?	PN	
	**Risk of bias judgement**	**Low**	
**Overall risk of bias**	**Risk of bias judgement**	**High**	

**Study**	Feeley 1975		
**Domain**	**Signalling question**	**Response**	**Comments**
**Bias arising from the randomization process**	1.1 Was the allocation sequence random?	NI	"Patients selected for study were assigned randomly to receive either 5 cm of positive end-expiratory pressure or no positive end-expiratory pressure during weaning."
	1.2 Was the allocation sequence concealed until participants were enrolled and assigned to interventions?	PN	
	1.3 Did baseline differences between intervention groups suggest a problem with the randomization process?	N	
	**Risk of bias judgement**	**High**	
**Bias due to deviations from intended interventions**	2.1.Were participants aware of their assigned intervention during the trial?	PN	
	2.2.Were carers and people delivering the interventions aware of participants' assigned intervention during the trial?	NI	
	2.3. If Y/PY/NI to 2.1 or 2.2: Were there deviations from the intended intervention that arose because of the experimental context?	NI	

	2.4 If Y/PY to 2.3: Were these deviations likely to have affected the outcome?	NA	
	2.5. If Y/PY/NI to 2.4: Were these deviations from intended intervention balanced between groups?	NA	
	2.6 Was an appropriate analysis used to estimate the effect of assignment to intervention?	PY	
	2.7 If N/PN/NI to 2.6: Was there potential for a substantial impact (on the result) of the failure to analyse participants in the group to which they were randomized?	NA	
	**Risk of bias judgement**	**Some concerns**	
**Bias due to missing outcome data**	3.1 Were data for this outcome available for all, or nearly all, participants randomized?	Y	
	3.2 If N/PN/NI to 3.1: Is there evidence that result was not biased by missing outcome data?	NA	
	3.3 If N/PN to 3.2: Could missingness in the outcome depend on its true value?	NA	
	3.4 If Y/PY/NI to 3.3: Is it likely that missingness in the outcome depended on its true value?	NA	
	**Risk of bias judgement**	**Low**	
**Bias in measurement of the outcome**	4.1 Was the method of measuring the outcome inappropriate?	PN	
	4.2 Could measurement or ascertainment of the outcome have differed between intervention groups?	PY	
	4.3 Were outcome assessors aware of the intervention received by study participants?	NA	
	4.4 If Y/PY/NI to 4.3: Could assessment of the outcome have been influenced by knowledge of intervention received?	NA	
	4.5 If Y/PY/NI to 4.4: Is it likely that assessment of the outcome was influenced by knowledge of intervention received?	NA	

	**Risk of bias judgement**	**High**	
**Bias in selection of the reported result**	5.1 Were the data that produced this result analysed in accordance with a pre-specified analysis plan that was finalized before unblinded outcome data were available for analysis?	NI	
	5.2 ... multiple eligible outcome measurements (e.g. scales, definitions, time points) within the outcome domain?	PN	
	5.3 ... multiple eligible analyses of the data?	PN	
	**Risk of bias judgement**	**Some concerns**	
**Overall risk of bias**	**Risk of bias judgement**	**High**	

**Study**	Good 1979		
**Domain**	**Signalling question**	**Response**	**Comments**
**Bias arising from the randomization process**	1.1 Was the allocation sequence random?	NI	"Patients were randomly assigned to a group receiving therapy with PEEP (ten patients) or to a group with no PEEP (14 patients)."
	1.2 Was the allocation sequence concealed until participants were enrolled and assigned to interventions?	PN	
	1.3 Did baseline differences between intervention groups suggest a problem with the randomization process?	N	
	**Risk of bias judgement**	**High**	
**Bias due to deviations from intended interventions**	2.1.Were participants aware of their assigned intervention during the trial?	PN	

	2.2.Were carers and people delivering the interventions aware of participants' assigned intervention during the trial?	NI	
	2.3. If Y/PY/NI to 2.1 or 2.2: Were there deviations from the intended intervention that arose because of the experimental context?	NI	
	2.4 If Y/PY to 2.3: Were these deviations likely to have affected the outcome?	NA	
	2.5. If Y/PY/NI to 2.4: Were these deviations from intended intervention balanced between groups?	NA	
	2.6 Was an appropriate analysis used to estimate the effect of assignment to intervention?	Y	
	2.7 If N/PN/NI to 2.6: Was there potential for a substantial impact (on the result) of the failure to analyse participants in the group to which they were randomized?	NA	
	**Risk of bias judgement**	**Some concerns**	
**Bias due to missing outcome data**	3.1 Were data for this outcome available for all, or nearly all, participants randomized?	Y	
	3.2 If N/PN/NI to 3.1: Is there evidence that result was not biased by missing outcome data?	NA	
	3.3 If N/PN to 3.2: Could missingness in the outcome depend on its true value?	NA	
	3.4 If Y/PY/NI to 3.3: Is it likely that missingness in the outcome depended on its true value?	NA	
	**Risk of bias judgement**	**Low**	
**Bias in measurement of the outcome**	4.1 Was the method of measuring the outcome inappropriate?	PN	
	4.2 Could measurement or ascertainment of the outcome have differed between intervention groups?	PY	
	4.3 Were outcome assessors aware of the intervention received by study participants?	NA	

	4.4 If Y/PY/NI to 4.3: Could assessment of the outcome have been influenced by knowledge of intervention received?	NA	
	4.5 If Y/PY/NI to 4.4: Is it likely that assessment of the outcome was influenced by knowledge of intervention received?	NA	
	**Risk of bias judgement**	**High**	
**Bias in selection of the reported result**	5.1 Were the data that produced this result analysed in accordance with a pre-specified analysis plan that was finalized before unblinded outcome data were available for analysis?	NI	
	5.2 ... multiple eligible outcome measurements (e.g. scales, definitions, time points) within the outcome domain?	PN	
	5.3 ... multiple eligible analyses of the data?	PN	
	**Risk of bias judgement**	**Some concerns**	
**Overall risk of bias**	**Risk of bias judgement**	**High**	

**Study**	Holland 2007		
**Domain**	**Signalling question**	**Response**	**Comments**
**Bias arising from the randomization process**	1.1 Was the allocation sequence random?	Y	"On admission to the ICU, patients were randomised by using sealed envelopes into two groups and baseline measurements were taken."
	1.2 Was the allocation sequence concealed until participants were enrolled and assigned to interventions?	NI	
	1.3 Did baseline differences between intervention groups suggest a problem with the randomization process?	PN	

	**Risk of bias judgement**	**Some concerns**	
**Bias due to deviations from intended interventions**	2.1.Were participants aware of their assigned intervention during the trial?	PN	
	2.2.Were carers and people delivering the interventions aware of participants' assigned intervention during the trial?	NI	
	2.3. If Y/PY/NI to 2.1 or 2.2: Were there deviations from the intended intervention that arose because of the experimental context?	NI	
	2.4 If Y/PY to 2.3: Were these deviations likely to have affected the outcome?	NA	
	2.5. If Y/PY/NI to 2.4: Were these deviations from intended intervention balanced between groups?	NA	
	2.6 Was an appropriate analysis used to estimate the effect of assignment to intervention?	Y	
	2.7 If N/PN/NI to 2.6: Was there potential for a substantial impact (on the result) of the failure to analyse participants in the group to which they were randomized?	NA	
	**Risk of bias judgement**	**Some concerns**	
**Bias due to missing outcome data**	3.1 Were data for this outcome available for all, or nearly all, participants randomized?	Y	
	3.2 If N/PN/NI to 3.1: Is there evidence that result was not biased by missing outcome data?	NA	
	3.3 If N/PN to 3.2: Could missingness in the outcome depend on its true value?	NA	
	3.4 If Y/PY/NI to 3.3: Is it likely that missingness in the outcome depended on its true value?	NA	
	**Risk of bias judgement**	**Low**	
**Bias in measurement of the outcome**	4.1 Was the method of measuring the outcome inappropriate?	N	

	4.2 Could measurement or ascertainment of the outcome have differed between intervention groups?	PN	
	4.3 Were outcome assessors aware of the intervention received by study participants?	PN	
	4.4 If Y/PY/NI to 4.3: Could assessment of the outcome have been influenced by knowledge of intervention received?	NA	
	4.5 If Y/PY/NI to 4.4: Is it likely that assessment of the outcome was influenced by knowledge of intervention received?	NA	
	**Risk of bias judgement**	**Low**	
**Bias in selection of the reported result**	5.1 Were the data that produced this result analysed in accordance with a pre-specified analysis plan that was finalized before unblinded outcome data were available for analysis?	Y	
	5.2 ... multiple eligible outcome measurements (e.g. scales, definitions, time points) within the outcome domain?	PN	
	5.3 ... multiple eligible analyses of the data?	PN	
	**Risk of bias judgement**	**Low**	
**Overall risk of bias**	**Risk of bias judgement**	**Some concerns**	

**Study**	Korovesi 2011		
**Domain**	**Signalling question**	**Response**	**Comments**
**Bias arising from the randomization process**	1.1 Was the allocation sequence random?	PY	“All patients […] were randomly assigned to receive either zero end-

	1.2 Was the allocation sequence concealed until participants were enrolled and assigned to interventions?	NI	expiratory pressure (ZEEP; ZEEP group) or 8 cm H_2O of PEEP (PEEP group) following a predesigned chart of randomization.”
	1.3 Did baseline differences between intervention groups suggest a problem with the randomization process?	N	
	**Risk of bias judgement**	**Some concerns**	
**Bias due to deviations from intended interventions**	2.1.Were participants aware of their assigned intervention during the trial?	PN	
	2.2.Were carers and people delivering the interventions aware of participants' assigned intervention during the trial?	PY	
	2.3. If Y/PY/NI to 2.1 or 2.2: Were there deviations from the intended intervention that arose because of the experimental context?	NI	
	2.4 If Y/PY to 2.3: Were these deviations likely to have affected the outcome?	NA	
	2.5. If Y/PY/NI to 2.4: Were these deviations from intended intervention balanced between groups?	NA	
	2.6 Was an appropriate analysis used to estimate the effect of assignment to intervention?	Y	
	2.7 If N/PN/NI to 2.6: Was there potential for a substantial impact (on the result) of the failure to analyse participants in the group to which they were randomized?	NA	
	**Risk of bias judgement**	**Some concerns**	
**Bias due to missing outcome data**	3.1 Were data for this outcome available for all, or nearly all, participants randomized?	Y	
	3.2 If N/PN/NI to 3.1: Is there evidence that result was not biased by missing outcome data?	NA	
	3.3 If N/PN to 3.2: Could missingness in the outcome depend on its true value?	NA	
	3.4 If Y/PY/NI to 3.3: Is it likely that missingness in the outcome depended on its true value?	NA	

	**Risk of bias judgement**	**Low**	
**Bias in measurement of the outcome**	4.1 Was the method of measuring the outcome inappropriate?	N	
	4.2 Could measurement or ascertainment of the outcome have differed between intervention groups?	PN	
	4.3 Were outcome assessors aware of the intervention received by study participants?	PY	
	4.4 If Y/PY/NI to 4.3: Could assessment of the outcome have been influenced by knowledge of intervention received?	PY	
	4.5 If Y/PY/NI to 4.4: Is it likely that assessment of the outcome was influenced by knowledge of intervention received?	N	
	**Risk of bias judgement**	**Some concerns**	
**Bias in selection of the reported result**	5.1 Were the data that produced this result analysed in accordance with a pre-specified analysis plan that was finalized before unblinded outcome data were available for analysis?	Y	
	5.2 ... multiple eligible outcome measurements (e.g. scales, definitions, time points) within the outcome domain?	PN	
	5.3 ... multiple eligible analyses of the data?	PN	
	**Risk of bias judgement**	**Low**	
**Overall risk of bias**	**Risk of bias judgement**	**Some concerns**	

**Study**	Korovesi 2016

Domain	Signalling question	Response	Comments
**Bias arising from the randomization process**	1.1 Was the allocation sequence random?	NI	"All patients […] were randomly assigned to receive 0 cmH2O of PEEP (ZEEP, n = 12) or 8 (PEEP, n = 15)."
	1.2 Was the allocation sequence concealed until participants were enrolled and assigned to interventions?	PN	
	1.3 Did baseline differences between intervention groups suggest a problem with the randomization process?	PN	
	**Risk of bias judgement**	**High**	
**Bias due to deviations from intended interventions**	2.1.Were participants aware of their assigned intervention during the trial?	PN	
	2.2.Were carers and people delivering the interventions aware of participants' assigned intervention during the trial?	PY	
	2.3. If Y/PY/NI to 2.1 or 2.2: Were there deviations from the intended intervention that arose because of the experimental context?	NI	
	2.4 If Y/PY to 2.3: Were these deviations likely to have affected the outcome?	NA	
	2.5. If Y/PY/NI to 2.4: Were these deviations from intended intervention balanced between groups?	NA	
	2.6 Was an appropriate analysis used to estimate the effect of assignment to intervention?	Y	
	2.7 If N/PN/NI to 2.6: Was there potential for a substantial impact (on the result) of the failure to analyse participants in the group to which they were randomized?	NA	
	**Risk of bias judgement**	**Some concerns**	
**Bias due to missing outcome data**	3.1 Were data for this outcome available for all, or nearly all, participants randomized?	Y	
	3.2 If N/PN/NI to 3.1: Is there evidence that result was not biased by missing outcome data?	NA	

	3.3 If N/PN to 3.2: Could missingness in the outcome depend on its true value?	NA	
	3.4 If Y/PY/NI to 3.3: Is it likely that missingness in the outcome depended on its true value?	NA	
	**Risk of bias judgement**	**Low**	
**Bias in measurement of the outcome**	4.1 Was the method of measuring the outcome inappropriate?	N	
	4.2 Could measurement or ascertainment of the outcome have differed between intervention groups?	PN	
	4.3 Were outcome assessors aware of the intervention received by study participants?	PY	
	4.4 If Y/PY/NI to 4.3: Could assessment of the outcome have been influenced by knowledge of intervention received?	PY	
	4.5 If Y/PY/NI to 4.4: Is it likely that assessment of the outcome was influenced by knowledge of intervention received?	PN	
	**Risk of bias judgement**	**Some concerns**	
**Bias in selection of the reported result**	5.1 Were the data that produced this result analysed in accordance with a pre-specified analysis plan that was finalized before unblinded outcome data were available for analysis?	Y	
	5.2 ... multiple eligible outcome measurements (e.g. scales, definitions, time points) within the outcome domain?	PN	
	5.3 ... multiple eligible analyses of the data?	PN	
	**Risk of bias judgement**	**Low**	
**Overall risk of bias**	**Risk of bias judgement**	**High**	

Study	Koutsoukou		
**Domain**	**Signalling question**	**Response**	**Comments**
**Bias arising from the randomization process**	1.1 Was the allocation sequence random?	NI	“Patients were randomly assigned to receive 8 cmH2O of PEEP or ZEEP.”
	1.2 Was the allocation sequence concealed until participants were enrolled and assigned to interventions?	PN	
	1.3 Did baseline differences between intervention groups suggest a problem with the randomization process?	PN	
	**Risk of bias judgement**	**High**	
**Bias due to deviations from intended interventions**	2.1.Were participants aware of their assigned intervention during the trial?	N	
	2.2.Were carers and people delivering the interventions aware of participants' assigned intervention during the trial?	NI	
	2.3. If Y/PY/NI to 2.1 or 2.2: Were there deviations from the intended intervention that arose because of the experimental context?	NI	
	2.4 If Y/PY to 2.3: Were these deviations likely to have affected the outcome?	NA	
	2.5. If Y/PY/NI to 2.4: Were these deviations from intended intervention balanced between groups?	NA	
	2.6 Was an appropriate analysis used to estimate the effect of assignment to intervention?	Y	
	2.7 If N/PN/NI to 2.6: Was there potential for a substantial impact (on the result) of the failure to analyse participants in the group to which they were randomized?	NA	
	**Risk of bias judgement**	**Some concerns**	

**Bias due to missing outcome data**	3.1 Were data for this outcome available for all, or nearly all, participants randomized?	Y	
	3.2 If N/PN/NI to 3.1: Is there evidence that result was not biased by missing outcome data?	NA	
	3.3 If N/PN to 3.2: Could missingness in the outcome depend on its true value?	NA	
	3.4 If Y/PY/NI to 3.3: Is it likely that missingness in the outcome depended on its true value?	NA	
	**Risk of bias judgement**	**Low**	
**Bias in measurement of the outcome**	4.1 Was the method of measuring the outcome inappropriate?	PN	
	4.2 Could measurement or ascertainment of the outcome have differed between intervention groups?	NI	
	4.3 Were outcome assessors aware of the intervention received by study participants?	NI	
	4.4 If Y/PY/NI to 4.3: Could assessment of the outcome have been influenced by knowledge of intervention received?	NI	
	4.5 If Y/PY/NI to 4.4: Is it likely that assessment of the outcome was influenced by knowledge of intervention received?	PN	
	**Risk of bias judgement**	**Some concerns**	
**Bias in selection of the reported result**	5.1 Were the data that produced this result analysed in accordance with a pre-specified analysis plan that was finalized before unblinded outcome data were available for analysis?	N	
	5.2 ... multiple eligible outcome measurements (e.g. scales, definitions, time points) within the outcome domain?	PN	
	5.3 ... multiple eligible analyses of the data?	PN	
	**Risk of bias judgement**	**Some concerns**	

**Overall risk of bias**	**Risk of bias judgement**	**High**	

**Study**	Lago Borges 2013		
**Domain**	**Signalling question**	**Response**	**Comments**
**Bias arising from the randomization process**	1.1 Was the allocation sequence random?	NI	Randomized clinical trial in a northeastern Brazilian federal university hospital."
	1.2 Was the allocation sequence concealed until participants were enrolled and assigned to interventions?	PN	
	1.3 Did baseline differences between intervention groups suggest a problem with the randomization process?	N	
	**Risk of bias judgement**	**High**	
**Bias due to deviations from intended interventions**	2.1.Were participants aware of their assigned intervention during the trial?	PN	"After draw, information about which group the patient would be allocated, was given to ICU members."
	2.2.Were carers and people delivering the interventions aware of participants' assigned intervention during the trial?	PY	
	2.3. If Y/PY/NI to 2.1 or 2.2: Were there deviations from the intended intervention that arose because of the experimental context?	NI	
	2.4 If Y/PY to 2.3: Were these deviations likely to have affected the outcome?	NA	
	2.5. If Y/PY/NI to 2.4: Were these deviations from intended intervention balanced between groups?	NA	
	2.6 Was an appropriate analysis used to estimate the effect of assignment to intervention?	Y	

	2.7 If N/PN/NI to 2.6: Was there potential for a substantial impact (on the result) of the failure to analyse participants in the group to which they were randomized?	NA	
	**Risk of bias judgement**	**Some concerns**	
**Bias due to missing outcome data**	3.1 Were data for this outcome available for all, or nearly all, participants randomized?	Y	
	3.2 If N/PN/NI to 3.1: Is there evidence that result was not biased by missing outcome data?	NA	
	3.3 If N/PN to 3.2: Could missingness in the outcome depend on its true value?	NA	
	3.4 If Y/PY/NI to 3.3: Is it likely that missingness in the outcome depended on its true value?	NA	
	**Risk of bias judgement**	**Low**	
**Bias in measurement of the outcome**	4.1 Was the method of measuring the outcome inappropriate?	PN	
	4.2 Could measurement or ascertainment of the outcome have differed between intervention groups?	PY	
	4.3 Were outcome assessors aware of the intervention received by study participants?	NA	
	4.4 If Y/PY/NI to 4.3: Could assessment of the outcome have been influenced by knowledge of intervention received?	NA	
	4.5 If Y/PY/NI to 4.4: Is it likely that assessment of the outcome was influenced by knowledge of intervention received?	NA	
	**Risk of bias judgement**	**High**	
**Bias in selection of the reported result**	5.1 Were the data that produced this result analysed in accordance with a pre-specified analysis plan that was finalized before unblinded outcome data were available for analysis?	N	
	5.2 ... multiple eligible outcome measurements (e.g. scales, definitions, time points) within the outcome domain?	Y	"We also excluded patients who died in the perioperative period before weaning from mechanical ventilation."

	5.3 ... multiple eligible analyses of the data?	PN	
	**Risk of bias judgement**	**High**	
**Overall risk of bias**	**Risk of bias judgement**	**High**	

**Study**	Lago Borges 2014		
**Domain**	**Signalling question**	**Response**	**Comments**
**Bias arising from the randomization process**	1.1 Was the allocation sequence random?	NI	"Randomized clinical trial conducted in a federal university hospital in northeastern Brazil."<br><br>"Patients were assigned to one of three groups using a simple draw"
	1.2 Was the allocation sequence concealed until participants were enrolled and assigned to interventions?	PN	
	1.3 Did baseline differences between intervention groups suggest a problem with the randomization process?	N	
	**Risk of bias judgement**	**High**	
**Bias due to deviations from intended interventions**	2.1.Were participants aware of their assigned intervention during the trial?	PN	"Patients were assigned to one of three groups using a simple draw"
	2.2.Were carers and people delivering the interventions aware of participants' assigned intervention during the trial?	PY	
	2.3. If Y/PY/NI to 2.1 or 2.2: Were there deviations from the intended intervention that arose because of the experimental context?	NI	
	2.4 If Y/PY to 2.3: Were these deviations likely to have affected the outcome?	NA	

	2.5. If Y/PY/NI to 2.4: Were these deviations from intended intervention balanced between groups?	NA	
	2.6 Was an appropriate analysis used to estimate the effect of assignment to intervention?	Y	
	2.7 If N/PN/NI to 2.6: Was there potential for a substantial impact (on the result) of the failure to analyse participants in the group to which they were randomized?	NA	
	**Risk of bias judgement**	**Some concerns**	
**Bias due to missing outcome data**	3.1 Were data for this outcome available for all, or nearly all, participants randomized?	Y	
	3.2 If N/PN/NI to 3.1: Is there evidence that result was not biased by missing outcome data?	NA	
	3.3 If N/PN to 3.2: Could missingness in the outcome depend on its true value?	NA	
	3.4 If Y/PY/NI to 3.3: Is it likely that missingness in the outcome depended on its true value?	NA	
	**Risk of bias judgement**	**Low**	
**Bias in measurement of the outcome**	4.1 Was the method of measuring the outcome inappropriate?	N	
	4.2 Could measurement or ascertainment of the outcome have differed between intervention groups?	PY	
	4.3 Were outcome assessors aware of the intervention received by study participants?	NA	
	4.4 If Y/PY/NI to 4.3: Could assessment of the outcome have been influenced by knowledge of intervention received?	NA	
	4.5 If Y/PY/NI to 4.4: Is it likely that assessment of the outcome was influenced by knowledge of intervention received?	NA	
	**Risk of bias judgement**	**High**	

**Bias in selection of the reported result**	5.1 Were the data that produced this result analysed in accordance with a pre-specified analysis plan that was finalized before unblinded outcome data were available for analysis?	PN	
	5.2 ... multiple eligible outcome measurements (e.g. scales, definitions, time points) within the outcome domain?	Y	"We also excluded patients who died in the perioperative period before weaning from mechanical ventilation."<br><br>"Comparing only patients extubated within 12 hours after ICU admission, i.e., uncomplicated in the immediate postoperative period, we found a statistically significant difference in mechanical ventilation duration between the groups (p = 0.029)."
	5.3 ... multiple eligible analyses of the data?	PN	
	**Risk of bias judgement**	**High**	
**Overall risk of bias**	**Risk of bias judgement**	**High**	

**Study**	Lesur		
**Domain**	**Signalling question**	**Response**	**Comments**
**Bias arising from the randomization process**	1.1 Was the allocation sequence random?	Y	"A computer-generated block randomization list was prepared by the principal investigator (Olivier Lesur). Randomization was concealed using numbered, sealed, opaque envelopes. On assessment of
	1.2 Was the allocation sequence concealed until participants were enrolled and assigned to interventions?	Y	

			the patient's eligibility, the randomization process was initiated by the opening of the first numbered envelope by the 'on ward' respiratory therapist. The ICU physician on duty was blinded to this procedure; the respiratory therapist adjusted the MV parameters (according to the physician's recommendations) with ZEEP or PEEP (according to the study's allocation), masking visual identification of allocation for the following 90 min. The ICU physician could halt the blinding at any moment, whenever he or she was not comfortable with the protocol."
	1.3 Did baseline differences between intervention groups suggest a problem with the randomization process?	N	
	**Risk of bias judgement**	**Low**	
**Bias due to deviations from intended interventions**	2.1.Were participants aware of their assigned intervention during the trial?	PN	
	2.2.Were carers and people delivering the interventions aware of participants' assigned intervention during the trial?	PY	
	2.3. If Y/PY/NI to 2.1 or 2.2: Were there deviations from the intended intervention that arose because of the experimental context?	N	
	2.4 If Y/PY to 2.3: Were these deviations likely to have affected the outcome?	NA	
	2.5. If Y/PY/NI to 2.4: Were these deviations from intended intervention balanced between groups?	NA	
	2.6 Was an appropriate analysis used to estimate the effect of assignment to intervention?	Y	

	2.7 If N/PN/NI to 2.6: Was there potential for a substantial impact (on the result) of the failure to analyse participants in the group to which they were randomized?	NA	
	**Risk of bias judgement**	**Low**	
**Bias due to missing outcome data**	3.1 Were data for this outcome available for all, or nearly all, participants randomized?	Y	
	3.2 If N/PN/NI to 3.1: Is there evidence that result was not biased by missing outcome data?	NA	
	3.3 If N/PN to 3.2: Could missingness in the outcome depend on its true value?	NA	
	3.4 If Y/PY/NI to 3.3: Is it likely that missingness in the outcome depended on its true value?	NA	
	**Risk of bias judgement**	**Low**	
**Bias in measurement of the outcome**	4.1 Was the method of measuring the outcome inappropriate?	N	"The lowest MAP values for each individual and period were selected to be representative for delta calculation and mean measurements."
	4.2 Could measurement or ascertainment of the outcome have differed between intervention groups?	PN	
	4.3 Were outcome assessors aware of the intervention received by study participants?	PY	
	4.4 If Y/PY/NI to 4.3: Could assessment of the outcome have been influenced by knowledge of intervention received?	PY	
	4.5 If Y/PY/NI to 4.4: Is it likely that assessment of the outcome was influenced by knowledge of intervention received?	PN	
	**Risk of bias judgement**	**Some concerns**	
**Bias in selection of the reported result**	5.1 Were the data that produced this result analysed in accordance with a pre-specified analysis plan that was finalized before unblinded outcome data were available for analysis?	Y	"The general characteristics of both intention-to-treat study groups were very similar and representative of a

			typical medical ICU admission profile.”
	5.2 ... multiple eligible outcome measurements (e.g. scales, definitions, time points) within the outcome domain?	PN	
	5.3 ... multiple eligible analyses of the data?	PN	
	**Risk of bias judgement**	**Low**	
**Overall risk of bias**	**Risk of bias judgement**	**Some concerns**	

**Study**	Ma		
**Domain**	**Signalling question**	**Response**	**Comments**
**Bias arising from the randomization process**	1.1 Was the allocation sequence random?	Y	“120 patients with NEP admitted to Department of Critical Care Medicine of the First Affiliated Hospital of Guangxi Traditional Chinese Medical University from January 2010 to August 2013 were enrolled and divided into two groups according to random number table (n=60 in each group).”
	1.2 Was the allocation sequence concealed until participants were enrolled and assigned to interventions?	NI	
	1.3 Did baseline differences between intervention groups suggest a problem with the randomization process?	N	
	**Risk of bias judgement**	**Some concerns**	
**Bias due to deviations from intended interventions**	2.1.Were participants aware of their assigned intervention during the trial?	PN	

	2.2.Were carers and people delivering the interventions aware of participants' assigned intervention during the trial?	NI	
	2.3. If Y/PY/NI to 2.1 or 2.2: Were there deviations from the intended intervention that arose because of the experimental context?	N	
	2.4 If Y/PY to 2.3: Were these deviations likely to have affected the outcome?	NA	
	2.5. If Y/PY/NI to 2.4: Were these deviations from intended intervention balanced between groups?	NA	
	2.6 Was an appropriate analysis used to estimate the effect of assignment to intervention?	Y	
	2.7 If N/PN/NI to 2.6: Was there potential for a substantial impact (on the result) of the failure to analyse participants in the group to which they were randomized?	NA	
	**Risk of bias judgement**	**Low**	
**Bias due to missing outcome data**	3.1 Were data for this outcome available for all, or nearly all, participants randomized?	Y	
	3.2 If N/PN/NI to 3.1: Is there evidence that result was not biased by missing outcome data?	NA	
	3.3 If N/PN to 3.2: Could missingness in the outcome depend on its true value?	NA	
	3.4 If Y/PY/NI to 3.3: Is it likely that missingness in the outcome depended on its true value?	NA	
	**Risk of bias judgement**	**Low**	
**Bias in measurement of the outcome**	4.1 Was the method of measuring the outcome inappropriate?	PN	
	4.2 Could measurement or ascertainment of the outcome have differed between intervention groups?	PN	
	4.3 Were outcome assessors aware of the intervention received by study participants?	NI	

	4.4 If Y/PY/NI to 4.3: Could assessment of the outcome have been influenced by knowledge of intervention received?	NI	
	4.5 If Y/PY/NI to 4.4: Is it likely that assessment of the outcome was influenced by knowledge of intervention received?	PN	
	**Risk of bias judgement**	**Some concerns**	
**Bias in selection of the reported result**	5.1 Were the data that produced this result analysed in accordance with a pre-specified analysis plan that was finalized before unblinded outcome data were available for analysis?	PY	
	5.2 ... multiple eligible outcome measurements (e.g. scales, definitions, time points) within the outcome domain?	NI	
	5.3 ... multiple eligible analyses of the data?	PN	
	**Risk of bias judgement**	**Some concerns**	
**Overall risk of bias**	**Risk of bias judgement**	**Some concerns**	

**Study**	Manzano		
**Domain**	**Signalling question**	**Response**	**Comments**
**Bias arising from the randomization process**	1.1 Was the allocation sequence random?	Y	"This prospective, randomized, nonblinded, controlled clinical trial was performed in two general intensive care units (ICUs) and one trauma ICU in two reference centers in Spain."
	1.2 Was the allocation sequence concealed until participants were enrolled and assigned to interventions?	PY	

			“Patients were assigned to the PEEP group or control group using a computer-generated randomization list in blocks of 12. Allocation to control group or PEEP group was concealed in a closed envelope by an assistant not involved in the study.”
	1.3 Did baseline differences between intervention groups suggest a problem with the randomization process?	N	
	**Risk of bias judgement**	**Low**	
**Bias due to deviations from intended interventions**	2.1.Were participants aware of their assigned intervention during the trial?	PN	
	2.2.Were carers and people delivering the interventions aware of participants' assigned intervention during the trial?	Y	
	2.3. If Y/PY/NI to 2.1 or 2.2: Were there deviations from the intended intervention that arose because of the experimental context?	NI	
	2.4 If Y/PY to 2.3: Were these deviations likely to have affected the outcome?	NA	
	2.5. If Y/PY/NI to 2.4: Were these deviations from intended intervention balanced between groups?	NA	
	2.6 Was an appropriate analysis used to estimate the effect of assignment to intervention?	Y	“All analyses were conducted on an intention-to-treat basis.”
	2.7 If N/PN/NI to 2.6: Was there potential for a substantial impact (on the result) of the failure to analyse participants in the group to which they were randomized?	NA	
	**Risk of bias judgement**	**Some concerns**	
**Bias due to missing outcome data**	3.1 Were data for this outcome available for all, or nearly all, participants randomized?	Y	
	3.2 If N/PN/NI to 3.1: Is there evidence that result was not biased by missing outcome data?	NA	

	3.3 If N/PN to 3.2: Could missingness in the outcome depend on its true value?	NA	
	3.4 If Y/PY/NI to 3.3: Is it likely that missingness in the outcome depended on its true value?	NA	
	**Risk of bias judgement**	**Low**	
**Bias in measurement of the outcome**	4.1 Was the method of measuring the outcome inappropriate?	N	"Radiographs were interpreted by consensus between two physicians (intensivists with >5 yrs experience) and, if agree- ment could not be reached, by decision of a third physician."
	4.2 Could measurement or ascertainment of the outcome have differed between intervention groups?	PN	
	4.3 Were outcome assessors aware of the intervention received by study participants?	Y	
	4.4 If Y/PY/NI to 4.3: Could assessment of the outcome have been influenced by knowledge of intervention received?	PY	The precise definitions of the outcomes reduce the likelihood of subjective outcome assessment.
	4.5 If Y/PY/NI to 4.4: Is it likely that assessment of the outcome was influenced by knowledge of intervention received?	PN	
	**Risk of bias judgement**	**Some concerns**	
**Bias in selection of the reported result**	5.1 Were the data that produced this result analysed in accordance with a pre-specified analysis plan that was finalized before unblinded outcome data were available for analysis?	Y	
	5.2 ... multiple eligible outcome measurements (e.g. scales, definitions, time points) within the outcome domain?	PN	
	5.3 ... multiple eligible analyses of the data?	PN	
	**Risk of bias judgement**	**Low**	
**Overall risk of bias**	**Risk of bias judgement**	**Some concerns**	

Study	Marvel		
**Domain**	**Signalling question**	**Response**	**Comments**
**Bias arising from the randomization process**	1.1 Was the allocation sequence random?	Y	"The remaining 44 patients were randomly assigned by computer to one of three groups. The computer program maintained an equal distribution of any individual surgeon's patients among the three treatment groups."
	1.2 Was the allocation sequence concealed until participants were enrolled and assigned to interventions?	NI	
	1.3 Did baseline differences between intervention groups suggest a problem with the randomization process?	PN	
	**Risk of bias judgement**	**Some concerns**	
**Bias due to deviations from intended interventions**	2.1.Were participants aware of their assigned intervention during the trial?	PN	
	2.2.Were carers and people delivering the interventions aware of participants' assigned intervention during the trial?	PY	
	2.3. If Y/PY/NI to 2.1 or 2.2: Were there deviations from the intended intervention that arose because of the experimental context?	NI	
	2.4 If Y/PY to 2.3: Were these deviations likely to have affected the outcome?	NA	
	2.5. If Y/PY/NI to 2.4: Were these deviations from intended intervention balanced between groups?	NA	
	2.6 Was an appropriate analysis used to estimate the effect of assignment to intervention?	PY	

	2.7 If N/PN/NI to 2.6: Was there potential for a substantial impact (on the result) of the failure to analyse participants in the group to which they were randomized?	NA	
	**Risk of bias judgement**	**Some concerns**	
**Bias due to missing outcome data**	3.1 Were data for this outcome available for all, or nearly all, participants randomized?	Y	
	3.2 If N/PN/NI to 3.1: Is there evidence that result was not biased by missing outcome data?	NA	
	3.3 If N/PN to 3.2: Could missingness in the outcome depend on its true value?	NA	
	3.4 If Y/PY/NI to 3.3: Is it likely that missingness in the outcome depended on its true value?	NA	
	**Risk of bias judgement**	**Low**	
**Bias in measurement of the outcome**	4.1 Was the method of measuring the outcome inappropriate?	PN	"These roentgenograms were graded for atelectasis by a chest radiologist who did not know the treatment assignments."
	4.2 Could measurement or ascertainment of the outcome have differed between intervention groups?	PY	
	4.3 Were outcome assessors aware of the intervention received by study participants?	NA	
	4.4 If Y/PY/NI to 4.3: Could assessment of the outcome have been influenced by knowledge of intervention received?	NA	
	4.5 If Y/PY/NI to 4.4: Is it likely that assessment of the outcome was influenced by knowledge of intervention received?	NA	
	**Risk of bias judgement**	**High**	
**Bias in selection of the reported result**	5.1 Were the data that produced this result analysed in accordance with a pre-specified analysis plan that was finalized before unblinded outcome data were available for analysis?	PY	

	5.2 ... multiple eligible outcome measurements (e.g. scales, definitions, time points) within the outcome domain?	PN	
	5.3 ... multiple eligible analyses of the data?	PN	
	**Risk of bias judgement**	**Low**	
**Overall risk of bias**	**Risk of bias judgement**	**High**	

**Study**	Michalopoulos		
**Domain**	**Signalling question**	**Response**	**Comments**
**Bias arising from the randomization process**	1.1 Was the allocation sequence random?	NI	"Patients were randomly assigned to receive zero PEEP (Group A, n=22), 5 cmH2O PEEP (Group B, n=24), or 10 cmH2O PEEP (Group C, n=21) during mechanical ventilatory support."
	1.2 Was the allocation sequence concealed until participants were enrolled and assigned to interventions?	PN	
	1.3 Did baseline differences between intervention groups suggest a problem with the randomization process?	N	
	**Risk of bias judgement**	**High**	
**Bias due to deviations from intended interventions**	2.1.Were participants aware of their assigned intervention during the trial?	PN	
	2.2.Were carers and people delivering the interventions aware of participants' assigned intervention during the trial?	PY	

	2.3. If Y/PY/NI to 2.1 or 2.2: Were there deviations from the intended intervention that arose because of the experimental context?	NI	
	2.4 If Y/PY to 2.3: Were these deviations likely to have affected the outcome?	NA	
	2.5. If Y/PY/NI to 2.4: Were these deviations from intended intervention balanced between groups?	NA	
	2.6 Was an appropriate analysis used to estimate the effect of assignment to intervention?	Y	
	2.7 If N/PN/NI to 2.6: Was there potential for a substantial impact (on the result) of the failure to analyse participants in the group to which they were randomized?	NA	
	**Risk of bias judgement**	**Some concerns**	
**Bias due to missing outcome data**	3.1 Were data for this outcome available for all, or nearly all, participants randomized?	Y	
	3.2 If N/PN/NI to 3.1: Is there evidence that result was not biased by missing outcome data?	NA	
	3.3 If N/PN to 3.2: Could missingness in the outcome depend on its true value?	NA	
	3.4 If Y/PY/NI to 3.3: Is it likely that missingness in the outcome depended on its true value?	NA	
	**Risk of bias judgement**	**Low**	
**Bias in measurement of the outcome**	4.1 Was the method of measuring the outcome inappropriate?	PN	
	4.2 Could measurement or ascertainment of the outcome have differed between intervention groups?	PY	
	4.3 Were outcome assessors aware of the intervention received by study participants?	NA	
	4.4 If Y/PY/NI to 4.3: Could assessment of the outcome have been influenced by knowledge of intervention received?	NA	

	4.5 If Y/PY/NI to 4.4: Is it likely that assessment of the outcome was influenced by knowledge of intervention received?	NA	
	**Risk of bias judgement**	**High**	
**Bias in selection of the reported result**	5.1 Were the data that produced this result analysed in accordance with a pre-specified analysis plan that was finalized before unblinded outcome data were available for analysis?	PY	
	5.2 ... multiple eligible outcome measurements (e.g. scales, definitions, time points) within the outcome domain?	PN	
	5.3 ... multiple eligible analyses of the data?	PN	
	**Risk of bias judgement**	**Low**	
**Overall risk of bias**	**Risk of bias judgement**	**High**	

**Study**	Murphy		
**Domain**	**Signalling question**	**Response**	**Comments**
**Bias arising from the randomization process**	1.1 Was the allocation sequence random?	NI	"After admission to the intensive care unit and after hematologic evaluations, patients in both groups were randomized to receive either 10 cm H20 of PEEP beginning 1 hour after the completion of the operation or no PEEP, and were studied for 8 hours."
	1.2 Was the allocation sequence concealed until participants were enrolled and assigned to interventions?	PN	
	1.3 Did baseline differences between intervention groups suggest a problem with the randomization process?	NI	

	**Risk of bias judgement**	**High**	
**Bias due to deviations from intended interventions**	2.1.Were participants aware of their assigned intervention during the trial?	PN	
	2.2.Were carers and people delivering the interventions aware of participants' assigned intervention during the trial?	PY	
	2.3. If Y/PY/NI to 2.1 or 2.2: Were there deviations from the intended intervention that arose because of the experimental context?	NI	
	2.4 If Y/PY to 2.3: Were these deviations likely to have affected the outcome?	NA	
	2.5. If Y/PY/NI to 2.4: Were these deviations from intended intervention balanced between groups?	NA	
	2.6 Was an appropriate analysis used to estimate the effect of assignment to intervention?	NI	
	2.7 If N/PN/NI to 2.6: Was there potential for a substantial impact (on the result) of the failure to analyse participants in the group to which they were randomized?	NI	
	**Risk of bias judgement**	**High**	
**Bias due to missing outcome data**	3.1 Were data for this outcome available for all, or nearly all, participants randomized?	NI	
	3.2 If N/PN/NI to 3.1: Is there evidence that result was not biased by missing outcome data?	N	
	3.3 If N/PN to 3.2: Could missingness in the outcome depend on its true value?	NI	
	3.4 If Y/PY/NI to 3.3: Is it likely that missingness in the outcome depended on its true value?	NI	
	**Risk of bias judgement**	**High**	
**Bias in measurement of the outcome**	4.1 Was the method of measuring the outcome inappropriate?	NI	

	4.2 Could measurement or ascertainment of the outcome have differed between intervention groups?	PY	
	4.3 Were outcome assessors aware of the intervention received by study participants?	NA	
	4.4 If Y/PY/NI to 4.3: Could assessment of the outcome have been influenced by knowledge of intervention received?	NA	
	4.5 If Y/PY/NI to 4.4: Is it likely that assessment of the outcome was influenced by knowledge of intervention received?	NA	
	**Risk of bias judgement**	**High**	
**Bias in selection of the reported result**	5.1 Were the data that produced this result analysed in accordance with a pre-specified analysis plan that was finalized before unblinded outcome data were available for analysis?	NI	
	5.2 ... multiple eligible outcome measurements (e.g. scales, definitions, time points) within the outcome domain?	NI	
	5.3 ... multiple eligible analyses of the data?	NI	
	**Risk of bias judgement**	**Some concerns**	
**Overall risk of bias**	**Risk of bias judgement**	**High**	

**Study**	Nelson		
**Domain**	**Signalling question**	**Response**	**Comments**
**Bias arising from the randomization process**	1.1 Was the allocation sequence random?	NI	

	1.2 Was the allocation sequence concealed until participants were enrolled and assigned to interventions?	PN	"Patients were assigned randomly to one of two treatment groups."
	1.3 Did baseline differences between intervention groups suggest a problem with the randomization process?	N	
	**Risk of bias judgement**	**High**	
**Bias due to deviations from intended interventions**	2.1.Were participants aware of their assigned intervention during the trial?	PN	
	2.2.Were carers and people delivering the interventions aware of participants' assigned intervention during the trial?	PY	
	2.3. If Y/PY/NI to 2.1 or 2.2: Were there deviations from the intended intervention that arose because of the experimental context?	NI	
	2.4 If Y/PY to 2.3: Were these deviations likely to have affected the outcome?	NA	
	2.5. If Y/PY/NI to 2.4: Were these deviations from intended intervention balanced between groups?	NA	
	2.6 Was an appropriate analysis used to estimate the effect of assignment to intervention?	Y	
	2.7 If N/PN/NI to 2.6: Was there potential for a substantial impact (on the result) of the failure to analyse participants in the group to which they were randomized?	NA	
	**Risk of bias judgement**	**Some concerns**	
**Bias due to missing outcome data**	3.1 Were data for this outcome available for all, or nearly all, participants randomized?	Y	
	3.2 If N/PN/NI to 3.1: Is there evidence that result was not biased by missing outcome data?	NA	
	3.3 If N/PN to 3.2: Could missingness in the outcome depend on its true value?	NA	
	3.4 If Y/PY/NI to 3.3: Is it likely that missingness in the outcome depended on its true value?	NA	

	**Risk of bias judgement**	**Low**	
**Bias in measurement of the outcome**	4.1 Was the method of measuring the outcome inappropriate?	N	
	4.2 Could measurement or ascertainment of the outcome have differed between intervention groups?	PY	
	4.3 Were outcome assessors aware of the intervention received by study participants?	NA	
	4.4 If Y/PY/NI to 4.3: Could assessment of the outcome have been influenced by knowledge of intervention received?	NA	
	4.5 If Y/PY/NI to 4.4: Is it likely that assessment of the outcome was influenced by knowledge of intervention received?	NA	
	**Risk of bias judgement**	**High**	
**Bias in selection of the reported result**	5.1 Were the data that produced this result analysed in accordance with a pre-specified analysis plan that was finalized before unblinded outcome data were available for analysis?	Y	
	5.2 ... multiple eligible outcome measurements (e.g. scales, definitions, time points) within the outcome domain?	PN	
	5.3 ... multiple eligible analyses of the data?	PN	
	**Risk of bias judgement**	**Low**	
**Overall risk of bias**	**Risk of bias judgement**	**High**	

**Study**	Pepe

Domain	Signalling question	Response	Comments
**Bias arising from the randomization process**	1.1 Was the allocation sequence random?	PY	"We randomly assigned patients to receive either no PEEP (control) or PEEP at a level of 8 cmH2O (early PEEP). […] Random permuted blocks of size 4 were used."
	1.2 Was the allocation sequence concealed until participants were enrolled and assigned to interventions?	NI	
	1.3 Did baseline differences between intervention groups suggest a problem with the randomization process?	N	
	**Risk of bias judgement**	**Some concerns**	
**Bias due to deviations from intended interventions**	2.1.Were participants aware of their assigned intervention during the trial?	PN	
	2.2.Were carers and people delivering the interventions aware of participants' assigned intervention during the trial?	PY	
	2.3. If Y/PY/NI to 2.1 or 2.2: Were there deviations from the intended intervention that arose because of the experimental context?	NI	
	2.4 If Y/PY to 2.3: Were these deviations likely to have affected the outcome?	NA	
	2.5. If Y/PY/NI to 2.4: Were these deviations from intended intervention balanced between groups?	NA	
	2.6 Was an appropriate analysis used to estimate the effect of assignment to intervention?	Y	
	2.7 If N/PN/NI to 2.6: Was there potential for a substantial impact (on the result) of the failure to analyse participants in the group to which they were randomized?	NA	
	**Risk of bias judgement**	**Some concerns**	
**Bias due to missing outcome data**	3.1 Were data for this outcome available for all, or nearly all, participants randomized?	Y	

	3.2 If N/PN/NI to 3.1: Is there evidence that result was not biased by missing outcome data?	NA	
	3.3 If N/PN to 3.2: Could missingness in the outcome depend on its true value?	NA	
	3.4 If Y/PY/NI to 3.3: Is it likely that missingness in the outcome depended on its true value?	NA	
	**Risk of bias judgement**	**Low**	
**Bias in measurement of the outcome**	4.1 Was the method of measuring the outcome inappropriate?	PN	
	4.2 Could measurement or ascertainment of the outcome have differed between intervention groups?	PY	
	4.3 Were outcome assessors aware of the intervention received by study participants?	NA	
	4.4 If Y/PY/NI to 4.3: Could assessment of the outcome have been influenced by knowledge of intervention received?	NA	
	4.5 If Y/PY/NI to 4.4: Is it likely that assessment of the outcome was influenced by knowledge of intervention received?	NA	
	**Risk of bias judgement**	**High**	
**Bias in selection of the reported result**	5.1 Were the data that produced this result analysed in accordance with a pre-specified analysis plan that was finalized before unblinded outcome data were available for analysis?	Y	
	5.2 ... multiple eligible outcome measurements (e.g. scales, definitions, time points) within the outcome domain?	PN	
	5.3 ... multiple eligible analyses of the data?	PN	
	**Risk of bias judgement**	**Low**	
**Overall risk of bias**	**Risk of bias judgement**	**High**	

Study	Relax		
**Domain**	**Signalling question**	**Response**	**Comments**
**Bias arising from the randomization process**	1.1 Was the allocation sequence random?	Y	"This was a randomized clinical trial conducted at the ICUs of 8 hospitals in the Netherlands."<br><br>"Patients were randomized in a 1:1 ratio to a lower or higher PEEP strategy group. The local investigators performed randomization using a central, dedicated, password-protected, encrypted, web-based automated randomization system (SSL-encrypted website with ALEA software, TenALEA Consortium). Randomization was conducted using random block sizes with a maximum of 8 patients."
	1.2 Was the allocation sequence concealed until participants were enrolled and assigned to interventions?	PY	
	1.3 Did baseline differences between intervention groups suggest a problem with the randomization process?	N	
	**Risk of bias judgement**	**Low**	
**Bias due to deviations from intended interventions**	2.1.Were participants aware of their assigned intervention during the trial?	PN	
	2.2.Were carers and people delivering the interventions aware of participants' assigned intervention during the trial?	Y	
	2.3. If Y/PY/NI to 2.1 or 2.2: Were there deviations from the intended intervention that arose because of the experimental context?	PN	

	2.4 If Y/PY to 2.3: Were these deviations likely to have affected the outcome?	NA	
	2.5. If Y/PY/NI to 2.4: Were these deviations from intended intervention balanced between groups?	NA	
	2.6 Was an appropriate analysis used to estimate the effect of assignment to intervention?	Y	"In all analyses, patients were analyzed according to their randomization group, with the exception of those who withdrew informed consent or were lost to follow-up in the first 28 days."
	2.7 If N/PN/NI to 2.6: Was there potential for a substantial impact (on the result) of the failure to analyse participants in the group to which they were randomized?	NA	
	**Risk of bias judgement**	**Low**	
**Bias due to missing outcome data**	3.1 Were data for this outcome available for all, or nearly all, participants randomized?	Y	
	3.2 If N/PN/NI to 3.1: Is there evidence that result was not biased by missing outcome data?	NA	
	3.3 If N/PN to 3.2: Could missingness in the outcome depend on its true value?	NA	
	3.4 If Y/PY/NI to 3.3: Is it likely that missingness in the outcome depended on its true value?	NA	
	**Risk of bias judgement**	**Low**	
**Bias in measurement of the outcome**	4.1 Was the method of measuring the outcome inappropriate?	N	"An independent committee oversaw conduct of the trial and adverse events while remaining blind to the primary end point at 3 predefined time points, and recommended the trial be continued."
	4.2 Could measurement or ascertainment of the outcome have differed between intervention groups?	PN	

	4.3 Were outcome assessors aware of the intervention received by study participants?	PY	
	4.4 If Y/PY/NI to 4.3: Could assessment of the outcome have been influenced by knowledge of intervention received?	PY	
	4.5 If Y/PY/NI to 4.4: Is it likely that assessment of the outcome was influenced by knowledge of intervention received?	PN	
	**Risk of bias judgement**	**Some concerns**	
**Bias in selection of the reported result**	5.1 Were the data that produced this result analysed in accordance with a pre-specified analysis plan that was finalized before unblinded outcome data were available for analysis?	Y	“The protocol has been published,15 and the final protocol is available in Online Resource 1. An updated statistical analysis plan was written be- fore closing the database; the final plan and a table describing the changes to the original study design are available in Online Resource 2.”
	5.2 ... multiple eligible outcome measurements (e.g. scales, definitions, time points) within the outcome domain?	PN	
	5.3 ... multiple eligible analyses of the data?	PN	
	**Risk of bias judgement**	**Low**	
**Overall risk of bias**	**Risk of bias judgement**	**Some concerns**	

**Study**	Vigil		
**Domain**	**Signalling question**	**Response**	**Comments**

**Bias arising from the randomization process**	1.1 Was the allocation sequence random?	PY	"Forty-four trauma patients were randomized to receive 5-cm H2O PEEP (PEEP) or 0-cm H2O PEEP (ZEEP)."
	1.2 Was the allocation sequence concealed until participants were enrolled and assigned to interventions?	NI	
	1.3 Did baseline differences between intervention groups suggest a problem with the randomization process?	N	
	**Risk of bias judgement**	**Some concerns**	
**Bias due to deviations from intended interventions**	2.1.Were participants aware of their assigned intervention during the trial?	PN	
	2.2.Were carers and people delivering the interventions aware of participants' assigned intervention during the trial?	PY	
	2.3. If Y/PY/NI to 2.1 or 2.2: Were there deviations from the intended intervention that arose because of the experimental context?	NI	
	2.4 If Y/PY to 2.3: Were these deviations likely to have affected the outcome?	NA	
	2.5. If Y/PY/NI to 2.4: Were these deviations from intended intervention balanced between groups?	NA	
	2.6 Was an appropriate analysis used to estimate the effect of assignment to intervention?	NI	
	2.7 If N/PN/NI to 2.6: Was there potential for a substantial impact (on the result) of the failure to analyse participants in the group to which they were randomized?	NI	
	**Risk of bias judgement**	**High**	
**Bias due to missing outcome data**	3.1 Were data for this outcome available for all, or nearly all, participants randomized?	Y	
	3.2 If N/PN/NI to 3.1: Is there evidence that result was not biased by missing outcome data?	NA	
	3.3 If N/PN to 3.2: Could missingness in the outcome depend on its true value?	NA	

	3.4 If Y/PY/NI to 3.3: Is it likely that missingness in the outcome depended on its true value?	NA	
	**Risk of bias judgement**	**Low**	
**Bias in measurement of the outcome**	4.1 Was the method of measuring the outcome inappropriate?	N	
	4.2 Could measurement or ascertainment of the outcome have differed between intervention groups?	PY	
	4.3 Were outcome assessors aware of the intervention received by study participants?	NA	
	4.4 If Y/PY/NI to 4.3: Could assessment of the outcome have been influenced by knowledge of intervention received?	NA	
	4.5 If Y/PY/NI to 4.4: Is it likely that assessment of the outcome was influenced by knowledge of intervention received?	NA	
	**Risk of bias judgement**	**High**	
**Bias in selection of the reported result**	5.1 Were the data that produced this result analysed in accordance with a pre-specified analysis plan that was finalized before unblinded outcome data were available for analysis?	Y	
	5.2 ... multiple eligible outcome measurements (e.g. scales, definitions, time points) within the outcome domain?	PN	
	5.3 ... multiple eligible analyses of the data?	PN	
	**Risk of bias judgement**	**Low**	
**Overall risk of bias**	**Risk of bias judgement**	**High**	

Study	Weigelt		
**Domain**	**Signalling question**	**Response**	**Comments**
**Bias arising from the randomization process**	1.1 Was the allocation sequence random?	NI	"This prospective randomized study was designed to determine the effect of early therapeutic PEEP on the incidence and severity of ARDS."
	1.2 Was the allocation sequence concealed until participants were enrolled and assigned to interventions?	PN	
	1.3 Did baseline differences between intervention groups suggest a problem with the randomization process?	N	
	**Risk of bias judgement**	**High**	
**Bias due to deviations from intended interventions**	2.1.Were participants aware of their assigned intervention during the trial?	PN	
	2.2.Were carers and people delivering the interventions aware of participants' assigned intervention during the trial?	PY	
	2.3. If Y/PY/NI to 2.1 or 2.2: Were there deviations from the intended intervention that arose because of the experimental context?	NI	
	2.4 If Y/PY to 2.3: Were these deviations likely to have affected the outcome?	NA	
	2.5. If Y/PY/NI to 2.4: Were these deviations from intended intervention balanced between groups?	NA	
	2.6 Was an appropriate analysis used to estimate the effect of assignment to intervention?	PY	
	2.7 If N/PN/NI to 2.6: Was there potential for a substantial impact (on the result) of the failure to analyse participants in the group to which they were randomized?	NA	
	**Risk of bias judgement**	**Some concerns**	

**Bias due to missing outcome data**	3.1 Were data for this outcome available for all, or nearly all, participants randomized?	Y	
	3.2 If N/PN/NI to 3.1: Is there evidence that result was not biased by missing outcome data?	NA	
	3.3 If N/PN to 3.2: Could missingness in the outcome depend on its true value?	NA	
	3.4 If Y/PY/NI to 3.3: Is it likely that missingness in the outcome depended on its true value?	NA	
	**Risk of bias judgement**	**Low**	
**Bias in measurement of the outcome**	4.1 Was the method of measuring the outcome inappropriate?	N	
	4.2 Could measurement or ascertainment of the outcome have differed between intervention groups?	PY	
	4.3 Were outcome assessors aware of the intervention received by study participants?	NA	
	4.4 If Y/PY/NI to 4.3: Could assessment of the outcome have been influenced by knowledge of intervention received?	NA	
	4.5 If Y/PY/NI to 4.4: Is it likely that assessment of the outcome was influenced by knowledge of intervention received?	NA	
	**Risk of bias judgement**	**High**	
**Bias in selection of the reported result**	5.1 Were the data that produced this result analysed in accordance with a pre-specified analysis plan that was finalized before unblinded outcome data were available for analysis?	NI	
	5.2 ... multiple eligible outcome measurements (e.g. scales, definitions, time points) within the outcome domain?	PN	
	5.3 ... multiple eligible analyses of the data?	PN	
	**Risk of bias judgement**	**Some concerns**	

**Overall risk of bias**	**Risk of bias judgement**	**High**	

**Study**	Zurich		
**Domain**	**Signalling question**	**Response**	**Comments**
**Bias arising from the randomization process**	1.1 Was the allocation sequence random?	N	"The patients were randomized preoperatively on the basis of the last digit of their clinical history number; odd-numbered patients were assigned to the group receiving PEEP, and even-numbered patients to the group not receiving PEEP (control group)."
	1.2 Was the allocation sequence concealed until participants were enrolled and assigned to interventions?	PN	
	1.3 Did baseline differences between intervention groups suggest a problem with the randomization process?	N	
	**Risk of bias judgement**	**High**	
**Bias due to deviations from intended interventions**	2.1.Were participants aware of their assigned intervention during the trial?	PN	
	2.2.Were carers and people delivering the interventions aware of participants' assigned intervention during the trial?	PY	
	2.3. If Y/PY/NI to 2.1 or 2.2: Were there deviations from the intended intervention that arose because of the experimental context?	NI	
	2.4 If Y/PY to 2.3: Were these deviations likely to have affected the outcome?	NA	
	2.5. If Y/PY/NI to 2.4: Were these deviations from intended intervention balanced between groups?	NA	

	2.6 Was an appropriate analysis used to estimate the effect of assignment to intervention?	Y	
	2.7 If N/PN/NI to 2.6: Was there potential for a substantial impact (on the result) of the failure to analyse participants in the group to which they were randomized?	NA	
	**Risk of bias judgement**	**Some concerns**	
**Bias due to missing outcome data**	3.1 Were data for this outcome available for all, or nearly all, participants randomized?	Y	
	3.2 If N/PN/NI to 3.1: Is there evidence that result was not biased by missing outcome data?	NA	
	3.3 If N/PN to 3.2: Could missingness in the outcome depend on its true value?	NA	
	3.4 If Y/PY/NI to 3.3: Is it likely that missingness in the outcome depended on its true value?	NA	
	**Risk of bias judgement**	**Low**	
**Bias in measurement of the outcome**	4.1 Was the method of measuring the outcome inappropriate?	PN	
	4.2 Could measurement or ascertainment of the outcome have differed between intervention groups?	PY	
	4.3 Were outcome assessors aware of the intervention received by study participants?	NA	
	4.4 If Y/PY/NI to 4.3: Could assessment of the outcome have been influenced by knowledge of intervention received?	NA	
	4.5 If Y/PY/NI to 4.4: Is it likely that assessment of the outcome was influenced by knowledge of intervention received?	NA	
	**Risk of bias judgement**	**High**	
**Bias in selection of the reported result**	5.1 Were the data that produced this result analysed in accordance with a pre-specified analysis plan that was finalized before unblinded outcome data were available for analysis?	PY	

	5.2 ... multiple eligible outcome measurements (e.g. scales, definitions, time points) within the outcome domain?	PN	
	5.3 ... multiple eligible analyses of the data?	PN	
	**Risk of bias judgement**	**Low**	
**Overall risk of bias**	**Risk of bias judgement**	**High**	

Online Resource 6. Primary and Secondary Outcomes

**Table S8. Summary of main meta-analysis**								
**Variable**	**Studies**	**N**	**Higher PEEP (N/total or N)**	**Lower PEEP**	**Relative effect of higher PEEP (95% CI)**	**Prediction Interval (95% CI)**	**I^2 (%)**	**p (I^2)**
Primary outcome								
Hospital mortality	9	1502	274/ 760	259/ 742	1.02 (0.89, 1.16)	1.02[a]	0	0.62
Secondary outcomes								
PaO_2/FiO_2	8	1444	732	712	50.46 mmHg (33.93, 66.99)	50.46 mmHg (11.87, 89.04)	59	0.02
Hypoxemia	5	1320	121/ 667	168/ 653	0.60 (0.40, 0.92)	0.60 (-1.55, 2.75)	59	0.05
ARDS	6	1315	28/ 672	54/ 643	0.50 (0.32, 0.78)	0.50 (-0.26,1.26)	13	0.33
A-aDO_2	4	164	80	84	-1.62 (-3.12, -0.11)	-1.62 (-9.03, 5.80)	92	< 0.01
Compliance	3	189	101	88	8.46 mL/cmH_2O (3.11, 13.82)	8.46 mL/cmH_2O[a]	0	0.82
Atelectasis	5	1255	65/ 632	74/ 623	1.02 (0.81, 1.28)	1.02 (0.38, 1.66)	11	0.34
Barotrauma	7	1372	38/ 697	52/ 675	0.78 (0.55, 1.11)	0.78[a]	0	0.54
VAP	3	1188	17/ 601	28/ 587	0.62 (0.32, 1.23)	0.62 (-4.01, 5.25)	23	0.27
Hypotension	5	283	18/ 141	16/ 142	1.15 (0.71, 1.84)	1.15[a]	0	0.72
CI	3	127	61	66	0.04 L/min/m^2 (-0.21, 0.29)	0.04[a]	0	0.93
CVP	3	106	51	55	1.37 mmHg (0.38, 2.37)	1.37[a]	0	0.38
24-hour postoperative bleeding	2	601	81	520	26.47 mL (-99.95, 152.89)	26.47 (-251.37, 304.31)	52	0.15
PRBC transfusion	3	1138	574	564	-0.38 units (-0.77, 0.02)	-0.38[a]	0	0.77
Duration of ventilation	10	1510	771	739	-0.03 (-0.27, 0.21)	-0.03 (-1.68, 1.62)	65	< 0.01
ICU stay	4	1202	617	585	-1.00 days (-2.51, 0.51)	-1.00 (-3.20, 1.20)	6	0.37
Hospital stay	5	1245	629	616	-0.02 days (-0.69, 0.66)	-0.02 (-1.53, 1.48)	26	0.25
ICU mortality	5	1073	194/ 546	172/ 527	1.09 (0.92, 1.28)	1.09[a]	0	0.74
28-day mortality	3	1152	231/ 583	236/ 569	0.68 (0.33, 1.40)	0.68 (-12.89, 14.25)	89	< 0.01

Table S8 (continued)								
**Variable**	**Studies**	**N**	**Higher PEEP (N/total or N)**	**Lower PEEP**	**Relative effect of higher PEEP (95% CI)**	**Prediction Interval (95% CI)**	**I^2 (%)**	**p (I^2)**
Abbreviations: N, number of patients; PEEP, positive end-expiratory pressure; CI, confidence interval; I^2, I^2 test; PaO_2/FiO_2, arterial partial pressure of oxygen to fraction of inspired oxygen ratio; A-aDO_2, alveolar-arterial oxygen pressure difference; VAP, ventilator-associated pneumonia; ARDS, acute respiratory distress syndrome; CI, cardiac index; CVP, central venous pressure; PRBC, packed red blood cells; ICU, intensive care unit.<br>Total effect is expressed as risk ratio (Mantel-Haenszel method, random-effects) for hospital mortality, hypoxemia, atelectasis, barotrauma, VAP, ARDS, hypotension, ICU mortality, 28-day mortality; mean difference (inverse variance method, random-effects) for PaO_2/FiO_2, compliance, cardiac index, CVP, postoperative bleeding, PRBC transfusion, ICU stay, hospital stay; and standardized mean difference (inverse variance, random-effects) for A-aDO_2 and duration of ventilation.<br>[a]I^2 and tau are zero.								

Forest plots of other secondary outcomes

Abbreviations: A-aDO_2, alveolar-arterial oxygen pressure difference; SD, standard deviation; IV, inverse variance; CI, confidence interval; PEEP, positive end-expiratory pressure; M-H, Mantel-Haenszel; VAP, ventilator-associated pneumonia; CI, cardiac index; CVP, central venous pressure; PRBC, packed red blood cell transfusion; ICU, intensive care unit; ARDS, acute respiratory distress syndrome.

A-aDO2

Study or Subgroup	Higher PEEP Mean	Higher PEEP SD	Higher PEEP Total	Lower PEEP Mean	Lower PEEP SD	Lower PEEP Total	Weight	Std. Mean Difference IV, Random, 95% CI
Feeley 1975	10	22	12	102	35	13	23.6%	−3.02 [−4.22, −1.81]
Koutsoukou 2006	100	41	11	87	40	10	25.5%	0.31 [−0.55, 1.17]
Lago Borges 2013	117	33	45	139	34	44	27.2%	−0.65 [−1.08, −0.22]
Marvel 1986	168	10	12	203	10	17	23.7%	−3.40 [−4.60, −2.20]
**Total (95% CI)**			**80**			**84**	**100.0%**	**−1.62 [−3.12, −0.11]**

Heterogeneity: $Tau^2 = 2.13$; $Chi^2 = 37.49$, df = 3 ($P < 0.00001$); $I^2 = 92\%$
Test for overall effect: Z = 2.10 (P = 0.04)

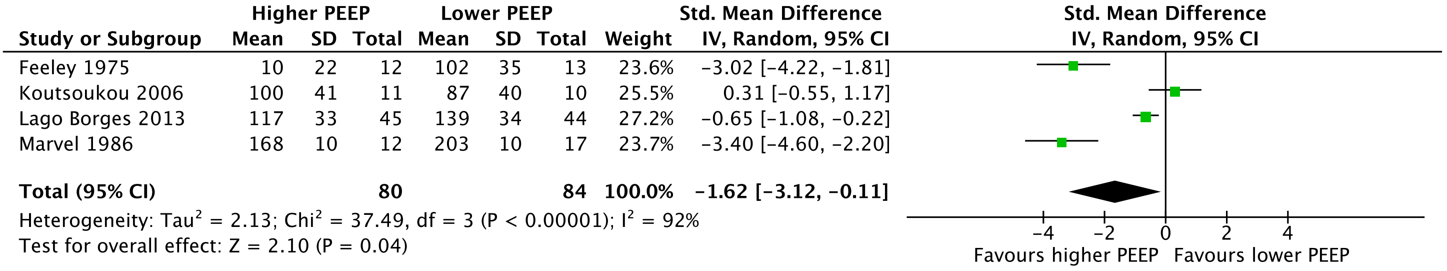

Compliance

Study or Subgroup	Higher PEEP Mean	Higher PEEP SD	Higher PEEP Total	Lower PEEP Mean	Lower PEEP SD	Lower PEEP Total	Weight	Mean Difference IV, Random, 95% CI
Koutsoukou 2006	62	14	11	53	11	10	24.9%	9.00 [−1.72, 19.72]
Lago Borges 2013	56	19	45	47	12	44	66.1%	9.00 [2.41, 15.59]
Weigelt 1979	42	36	45	39	43	34	9.0%	3.00 [−14.88, 20.88]
**Total (95% CI)**			**101**			**88**	**100.0%**	**8.46 [3.11, 13.82]**

Heterogeneity: $Tau^2 = 0.00$; $Chi^2 = 0.39$, df = 2 (P = 0.82); $I^2 = 0\%$
Test for overall effect: Z = 3.10 (P = 0.002)

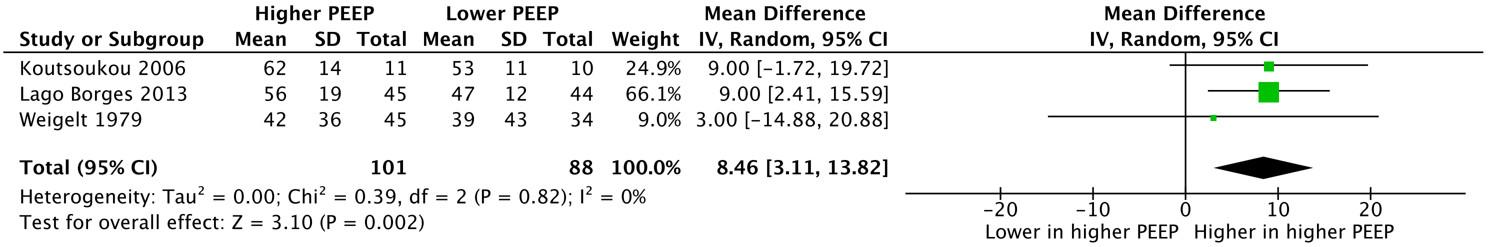

Atelectasis

	Higher PEEP		Lower PEEP			Risk Ratio	Risk Ratio
Study or Subgroup	Events	Total	Events	Total	Weight	M-H, Random, 95% CI	M-H, Random, 95% CI
Good 1979	9	10	12	14	44.3%	1.05 [0.78, 1.41]	
Manzano 2008	12	64	17	63	11.8%	0.69 [0.36, 1.33]	
Michalopoulos 1998	2	21	2	22	1.5%	1.05 [0.16, 6.77]	
Pepe 1984	27	44	23	48	30.8%	1.28 [0.88, 1.87]	
Relax 2020	15	493	20	476	11.6%	0.72 [0.38, 1.40]	
**Total (95% CI)**		**632**		**623**	**100.0%**	**1.02 [0.81, 1.28]**	
Total events	65		74				

Heterogeneity: $Tau^2 = 0.01$; $Chi^2 = 4.51$, df = 4 (P = 0.34); $I^2 = 11\%$
Test for overall effect: Z = 0.15 (P = 0.88)

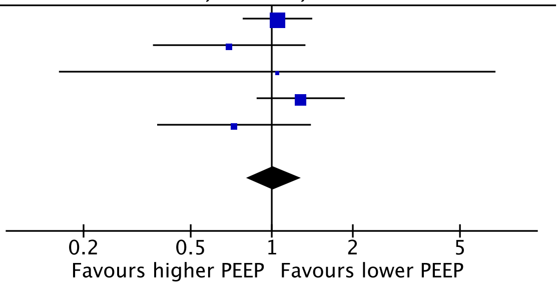

Barotrauma

Study or Subgroup	Higher PEEP Events	Higher PEEP Total	Lower PEEP Events	Lower PEEP Total	Weight	Risk Ratio M-H, Random, 95% CI
Good 1979	0	10	0	14		Not estimable
Manzano 2008	1	64	5	63	2.8%	0.20 [0.02, 1.64]
Michalopoulos 1998	0	21	0	22		Not estimable
Nelson 1987	1	20	0	18	1.3%	2.71 [0.12, 62.70]
Pepe 1984	19	44	24	48	63.5%	0.86 [0.56, 1.34]
Relax 2020	12	493	19	476	24.4%	0.61 [0.30, 1.24]
Weigelt 1979	5	45	4	34	8.1%	0.94 [0.27, 3.25]
**Total (95% CI)**		**697**		**675**	**100.0%**	**0.78 [0.55, 1.11]**
Total events	38		52			

Heterogeneity: $Tau^2 = 0.00$; $Chi^2 = 3.10$, df = 4 (P = 0.54); $I^2 = 0\%$
Test for overall effect: Z = 1.40 (P = 0.16)

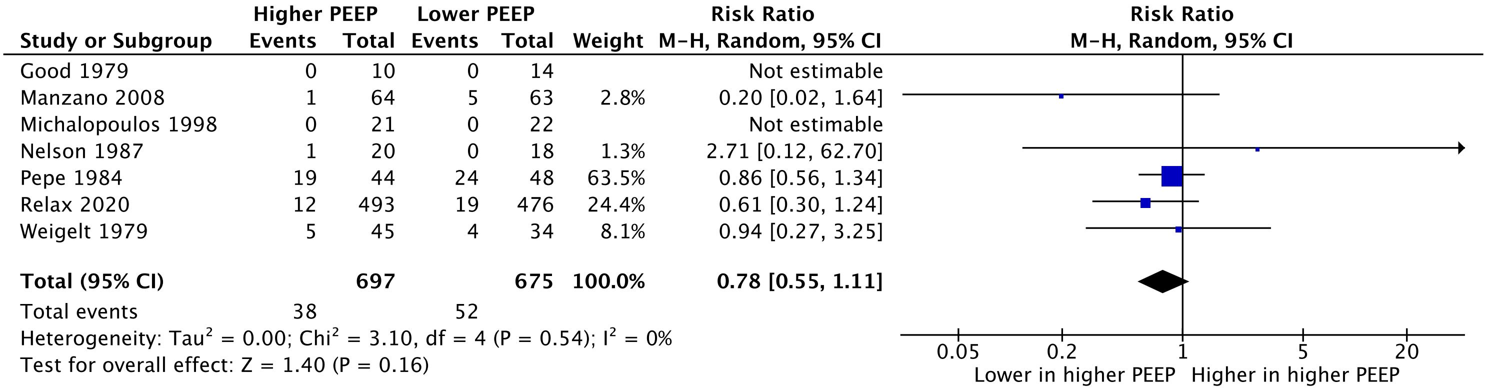

Ventilator-associated pneumonia

Study or Subgroup	Higher PEEP Events	Higher PEEP Total	Lower PEEP Events	Lower PEEP Total	Weight	Risk Ratio M-H, Random, 95% CI	Risk Ratio M-H, Random, 95% CI
Manzano 2008	6	64	16	63	42.8%	0.37 [0.15, 0.88]	
Pepe 1984	4	44	6	48	26.3%	0.73 [0.22, 2.41]	
Relax 2020	7	493	6	476	30.9%	1.13 [0.38, 3.33]	
**Total (95% CI)**		**601**		**587**	**100.0%**	**0.62 [0.32, 1.23]**	
Total events	17		28				

Heterogeneity: $Tau^2 = 0.08$; $Chi^2 = 2.59$, df = 2 (P = 0.27); $I^2 = 23\%$

Test for overall effect: Z = 1.36 (P = 0.17)

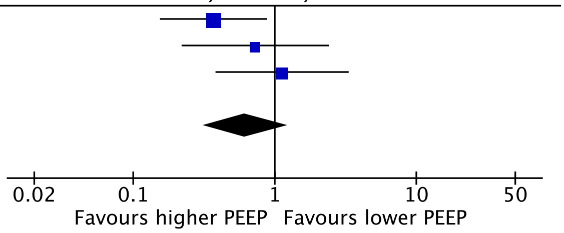

Hypotension

Study or Subgroup	Higher PEEP Events	Higher PEEP Total	Lower PEEP Events	Lower PEEP Total	Weight	Risk Ratio M-H, Random, 95% CI
Feeley 1975	0	12	0	13		Not estimable
Good 1979	0	10	0	14		Not estimable
Lesur 2010	16	30	16	33	95.5%	1.10 [0.68, 1.79]
Pepe 1984	1	44	0	48	2.2%	3.27 [0.14, 78.15]
Weigelt 1979	1	45	0	34	2.2%	2.28 [0.10, 54.36]
**Total (95% CI)**		**141**		**142**	**100.0%**	**1.15 [0.71, 1.84]**
Total events	18		16			

Heterogeneity: $Tau^2 = 0.00$; $Chi^2 = 0.67$, df = 2 (P = 0.72); $I^2 = 0\%$
Test for overall effect: Z = 0.56 (P = 0.57)

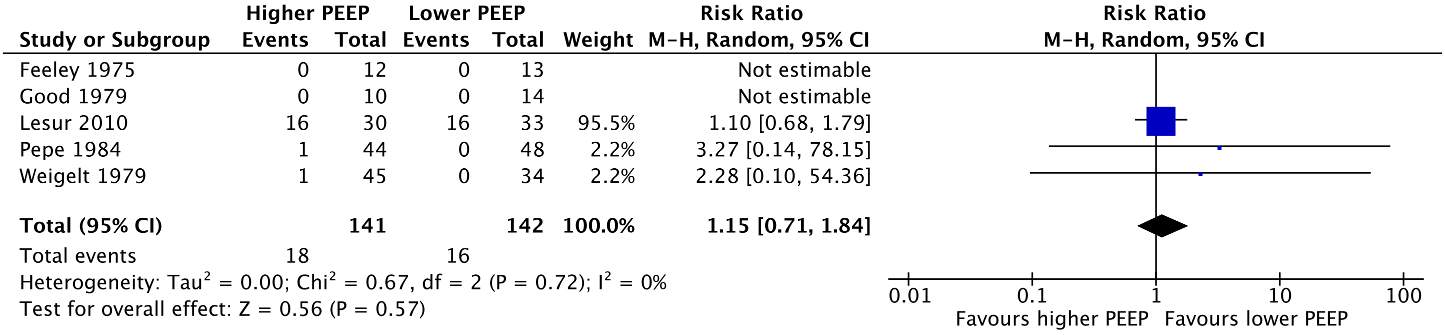

Cardiac index

Study or Subgroup	Higher PEEP Mean	Higher PEEP SD	Higher PEEP Total	Lower PEEP Mean	Lower PEEP SD	Lower PEEP Total	Weight	Mean Difference IV, Random, 95% CI
Collier 2002	3.1	0.86	40	3.1	0.64	44	59.6%	0.00 [−0.33, 0.33]
Dyhr 2002	2.2	0.6	7	2.1	1.1	8	8.2%	0.10 [−0.78, 0.98]
Holland 2007	3	0.6	14	2.9	0.6	14	32.2%	0.10 [−0.34, 0.54]
**Total (95% CI)**			**61**			**66**	**100.0%**	**0.04 [−0.21, 0.29]**

Heterogeneity: $Tau^2 = 0.00$; $Chi^2 = 0.15$, df = 2 (P = 0.93); $I^2 = 0\%$
Test for overall effect: Z = 0.31 (P = 0.75)

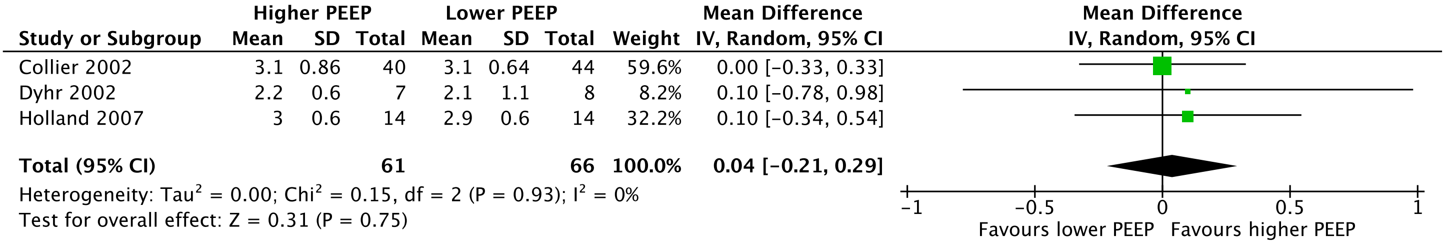

Central venous pressure

Study or Subgroup	Higher PEEP Mean	Higher PEEP SD	Higher PEEP Total	Lower PEEP Mean	Lower PEEP SD	Lower PEEP Total	Weight	Mean Difference IV, Random, 95% CI
Dyhr 2002	13	11	7	9.7	7.1	8	1.1%	3.30 [−6.22, 12.82]
Holland 2007	9	3	14	9	3	14	20.2%	0.00 [−2.22, 2.22]
Lesur 2010	12.5	1.5	30	10.8	2.9	33	78.7%	1.70 [0.57, 2.83]
**Total (95% CI)**			**51**			**55**	**100.0%**	**1.37 [0.38, 2.37]**

Heterogeneity: $Tau^2 = 0.00$; $Chi^2 = 1.95$, df = 2 (P = 0.38); $I^2 = 0\%$

Test for overall effect: Z = 2.70 (P = 0.007)

Mean Difference
IV, Random, 95% CI

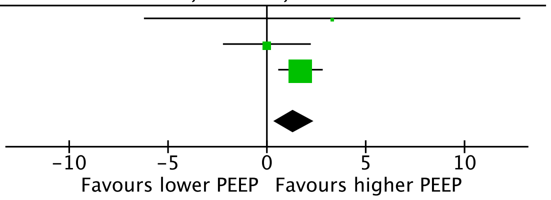

Postoperative bleeding

	Higher PEEP			Lower PEEP				Mean Difference
**Study or Subgroup**	**Mean**	**SD**	**Total**	**Mean**	**SD**	**Total**	**Weight**	**IV, Random, 95% CI**
Collier 2002	703	395	40	587	392	44	34.2%	116.00 [−52.52, 284.52]
Zurich 1982	542	239	41	562	261	476	65.8%	−20.00 [−96.82, 56.82]
**Total (95% CI)**			**81**			**520**	**100.0%**	**26.47 [−99.95, 152.89]**

Heterogeneity: $Tau^2 = 4783.35$; $Chi^2 = 2.07$, df = 1 (P = 0.15); $I^2 = 52\%$
Test for overall effect: Z = 0.41 (P = 0.68)

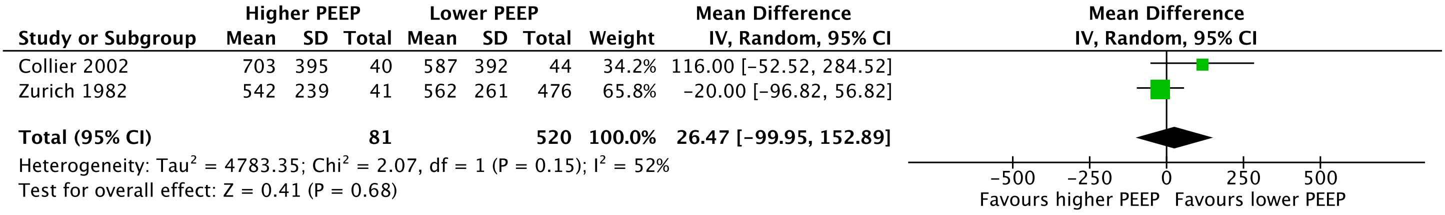

PRBC transfusion

Study or Subgroup	Higher PEEP Mean	Higher PEEP SD	Higher PEEP Total	Lower PEEP Mean	Lower PEEP SD	Lower PEEP Total	Weight	Mean Difference IV, Random, 95% CI
Collier 2002	0.8	1.4	40	1.1	1.6	44	37.5%	−0.30 [−0.94, 0.34]
Relax 2020	1.7	0.7	493	1	0	476		Not estimable
Zurich 1982	0.33	0.87	41	0.75	1.42	44	62.5%	−0.42 [−0.92, 0.08]
**Total (95% CI)**			**574**			**564**	**100.0%**	**−0.38 [−0.77, 0.02]**

Heterogeneity: $Tau^2 = 0.00$; $Chi^2 = 0.08$, df = 1 (P = 0.77); $I^2 = 0\%$
Test for overall effect: Z = 1.87 (P = 0.06)

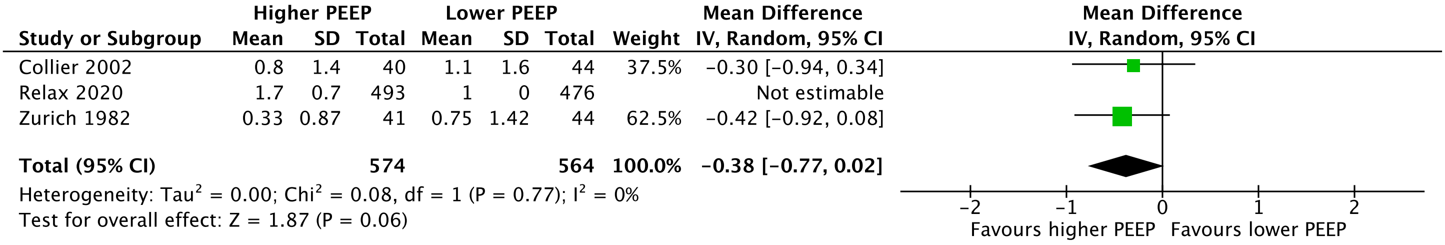

Duration of ventilation

	Higher PEEP			Lower PEEP				Std. Mean Difference	Std. Mean Difference
**Study or Subgroup**	**Mean**	**SD**	**Total**	**Mean**	**SD**	**Total**	**Weight**	**IV, Random, 95% CI**	**IV, Random, 95% CI**
Collier 2002	409.1	208.6	40	439.3	277.7	44	12.3%	−0.12 [−0.55, 0.31]	
Feeley 1975	258	217	12	259	149	13	6.4%	−0.01 [−0.79, 0.78]	
Good 1979	15.7	0.4	10	14.8	0.5	14	4.5%	1.88 [0.88, 2.88]	
Lago Borges 2014	5.1	2.9	45	6.8	3.2	44	12.4%	−0.55 [−0.98, −0.13]	
Lesur 2010	9.2	8.8	30	9.2	8.5	33	10.9%	0.00 [−0.49, 0.49]	
Manzano 2008	5.8	6.8	64	6.5	6.2	63	14.2%	−0.11 [−0.45, 0.24]	
Marvel 1986	9.3	0.6	12	0	0	0		Not estimable	
Nelson 1987	5.3	5	20	3.4	3	18	8.2%	0.45 [−0.20, 1.09]	
Relax 2020	4.8	6.6	493	5.5	7.4	476	19.2%	−0.10 [−0.23, 0.03]	
Weigelt 1979	9.3	13	45	14	21.7	34	11.9%	−0.27 [−0.72, 0.18]	
**Total (95% CI)**			**771**			**739**	**100.0%**	**−0.03 [−0.27, 0.21]**	

Heterogeneity: $Tau^2 = 0.07$; $Chi^2 = 23.01$, df = 8 (P = 0.003); $I^2 = 65\%$
Test for overall effect: Z = 0.24 (P = 0.81)

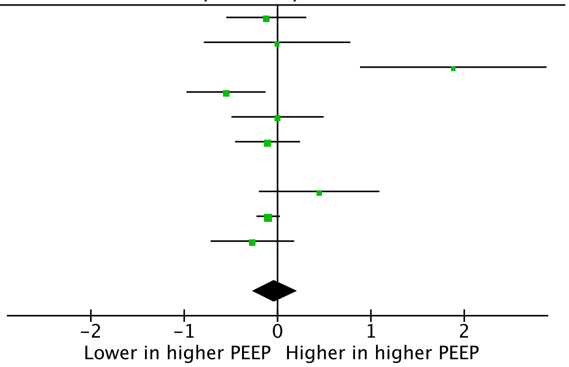

ICU stay

Study or Subgroup	Higher PEEP Mean	Higher PEEP SD	Higher PEEP Total	Lower PEEP Mean	Lower PEEP SD	Lower PEEP Total	Weight	Mean Difference IV, Random, 95% CI
Korovesi 2011	17.2	10.1	15	14.4	8.44	12	4.6%	2.80 [−4.19, 9.79]
Manzano 2008	10.5	9.8	64	12.3	11.4	63	15.5%	−1.80 [−5.50, 1.90]
Relax 2020	7.2	10.3	493	8.1	11.5	476	78.4%	−0.90 [−2.28, 0.48]
Weigelt 1979	11.7	16.8	45	21	32.5	34	1.6%	−9.30 [−21.28, 2.68]
**Total (95% CI)**			**617**			**585**	**100.0%**	**−1.00 [−2.51, 0.51]**

Heterogeneity: $Tau^2 = 0.26$; $Chi^2 = 3.18$, df = 3 (P = 0.37); $I^2 = 6\%$
Test for overall effect: Z = 1.30 (P = 0.19)

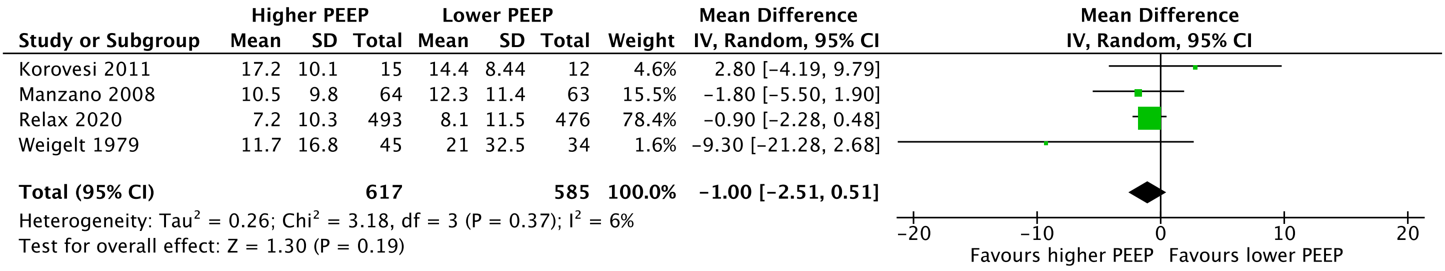

Hospital stay

Study or Subgroup	Higher PEEP Mean	Higher PEEP SD	Higher PEEP Total	Lower PEEP Mean	Lower PEEP SD	Lower PEEP Total	Weight	Mean Difference IV, Random, 95% CI
Collier 2002	5.7	2.5	40	5.2	1.7	44	31.1%	0.50 [−0.42, 1.42]
Manzano 2008	19.5	18.2	64	26.3	22	63	0.9%	−6.80 [−13.83, 0.23]
Marvel 1986	8.8	0.5	12	8.9	0.4	15	62.2%	−0.10 [−0.45, 0.25]
Nelson 1987	28	24	20	26	24	18	0.2%	2.00 [−13.28, 17.28]
Relax 2020	19	21.4	493	19.9	22.1	476	5.6%	−0.90 [−3.64, 1.84]
**Total (95% CI)**			**629**			**616**	**100.0%**	**−0.02 [−0.69, 0.66]**

Heterogeneity: $Tau^2 = 0.16$; $Chi^2 = 5.42$, df = 4 (P = 0.25); $I^2 = 26\%$
Test for overall effect: Z = 0.04 (P = 0.96)

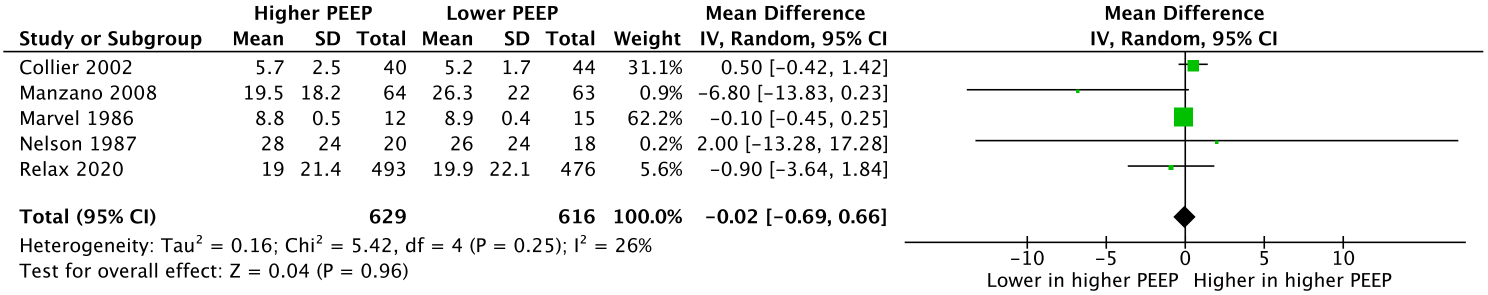

ICU mortality

Study or Subgroup	Higher PEEP Events	Higher PEEP Total	Lower PEEP Events	Lower PEEP Total	Weight	Risk Ratio M-H, Random, 95% CI
Dyhr 2002	0	7	0	8		Not estimable
Feeley 1975	2	12	1	13	0.5%	2.17 [0.22, 20.94]
Korovesi 2011	3	15	4	12	1.6%	0.60 [0.17, 2.18]
Nelson 1987	4	20	4	18	1.8%	0.90 [0.26, 3.08]
Relax 2020	185	492	163	476	96.0%	1.10 [0.93, 1.30]
**Total (95% CI)**		**546**		**527**	**100.0%**	**1.09 [0.92, 1.28]**
Total events	194		172			

Heterogeneity: $Tau^2 = 0.00$; $Chi^2 = 1.27$, df = 3 (P = 0.74); $I^2 = 0\%$

Test for overall effect: Z = 0.99 (P = 0.32)

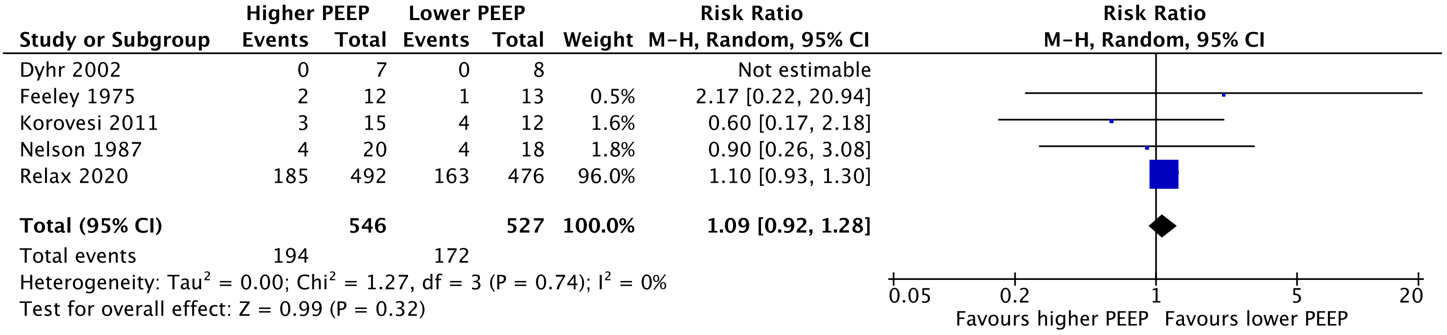

28-day mortality

Study or Subgroup	Higher PEEP Events	Higher PEEP Total	Lower PEEP Events	Lower PEEP Total	Weight	Risk Ratio M-H, Random, 95% CI
Lesur 2010	9	30	14	33	28.8%	0.71 [0.36, 1.39]
Ma 2014	15	60	39	60	33.1%	0.38 [0.24, 0.62]
Relax 2020	207	493	183	476	38.1%	1.09 [0.94, 1.27]
**Total (95% CI)**		**583**		**569**	**100.0%**	**0.68 [0.33, 1.40]**
Total events	231		236			

Heterogeneity: $Tau^2 = 0.35$; $Chi^2 = 17.71$, df = 2 (P = 0.0001); $I^2 = 89\%$
Test for overall effect: Z = 1.05 (P = 0.30)

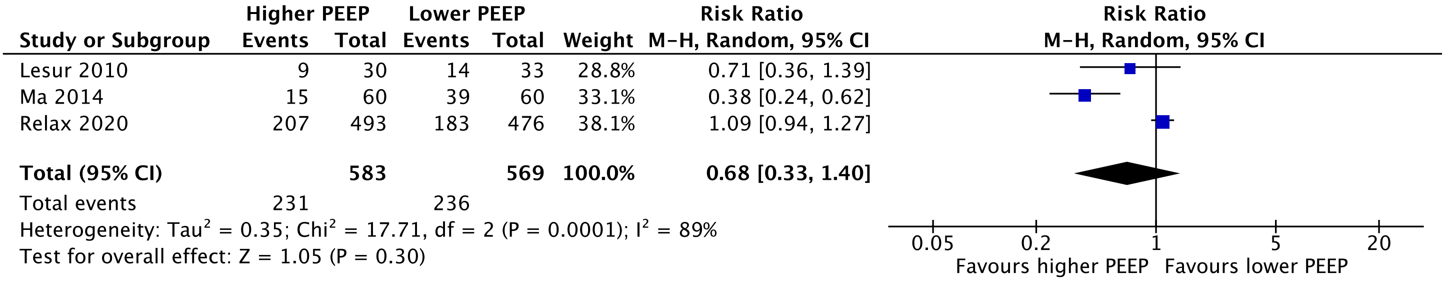

Online Resource 7. Funnel Plots

Abbreviations: SE, standard error; RR, risk ratio; MD, mean difference; SMD, standardized mean difference; PaO_2/FiO_2, arterial partial pressure of oxygen to fraction of inspired oxygen ratio; A-aDO_2, alveolar-arterial oxygen pressure difference; ARDS, acute respiratory distress syndrome; ICU, intensive care unit.

Hospital mortality

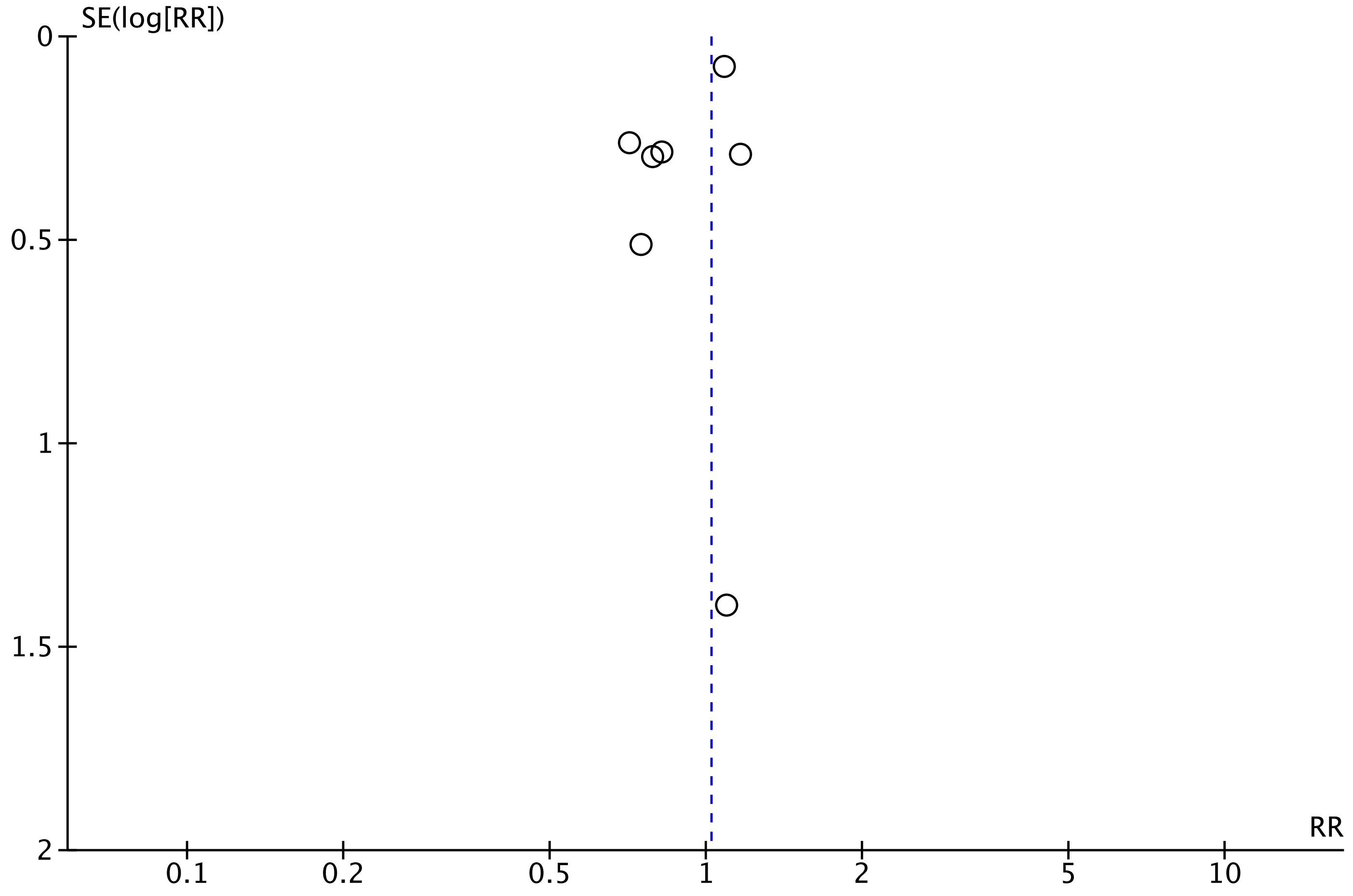

PaO2/FiO2

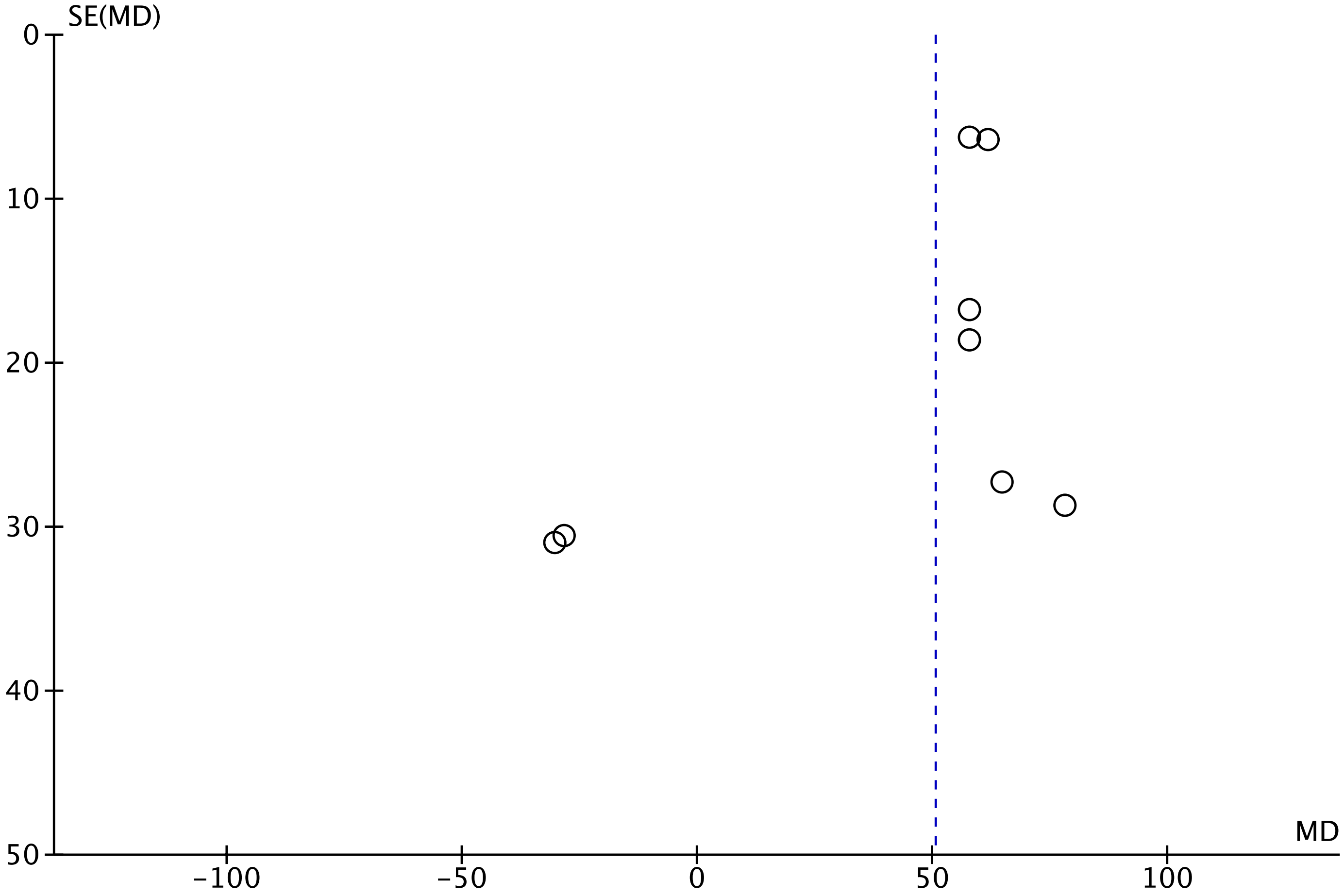

A-aDO2

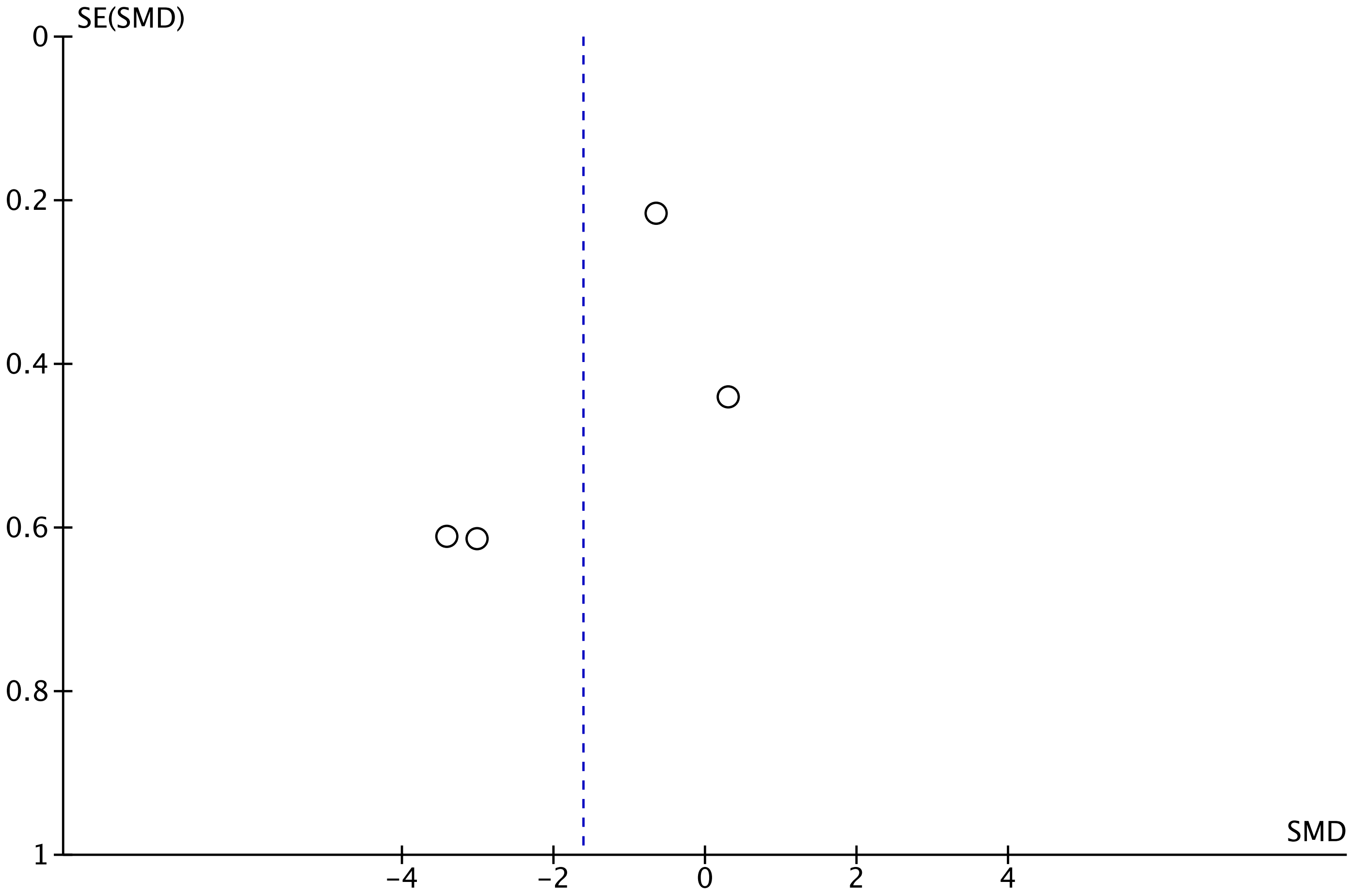

Compliance

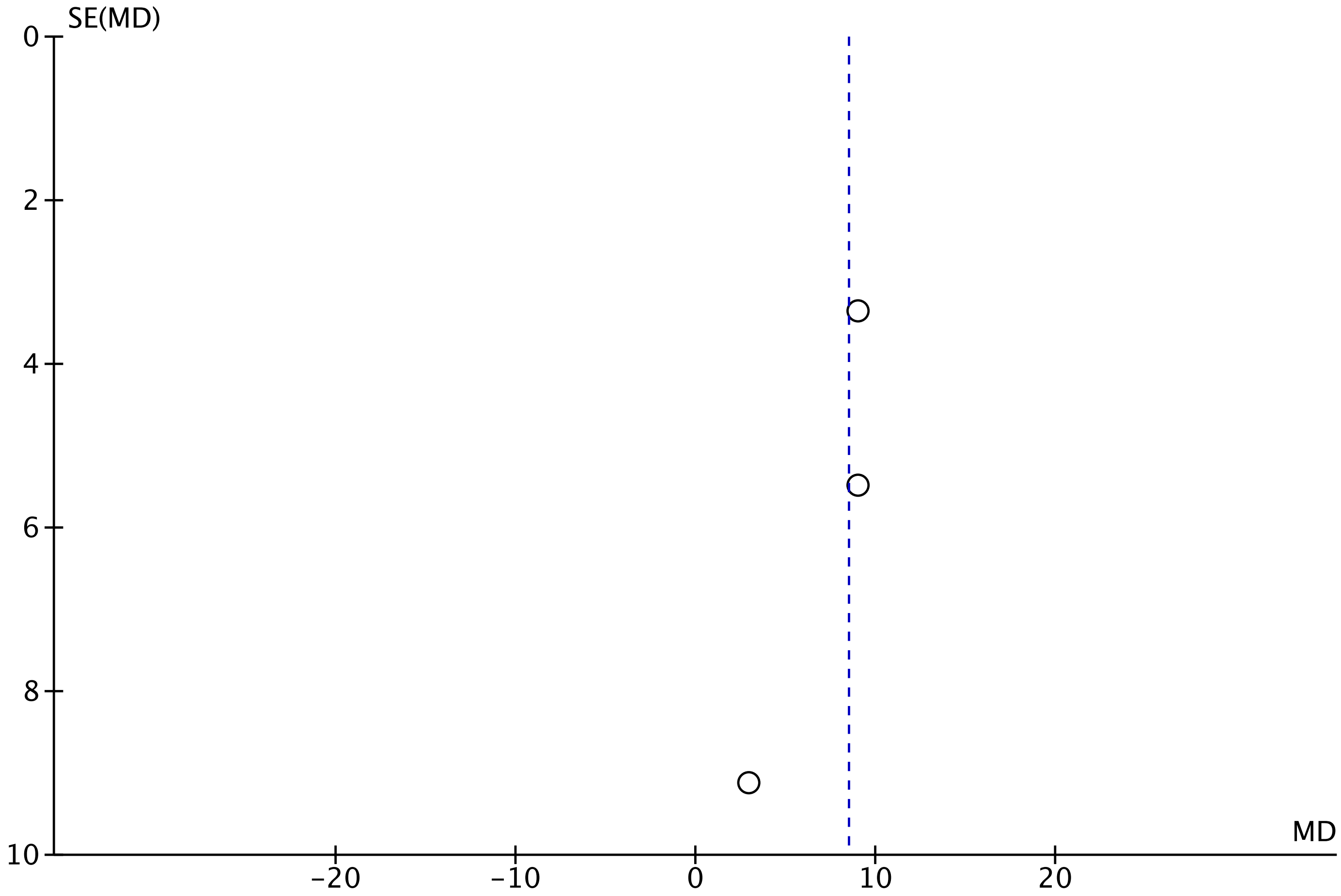

Hypoxemia

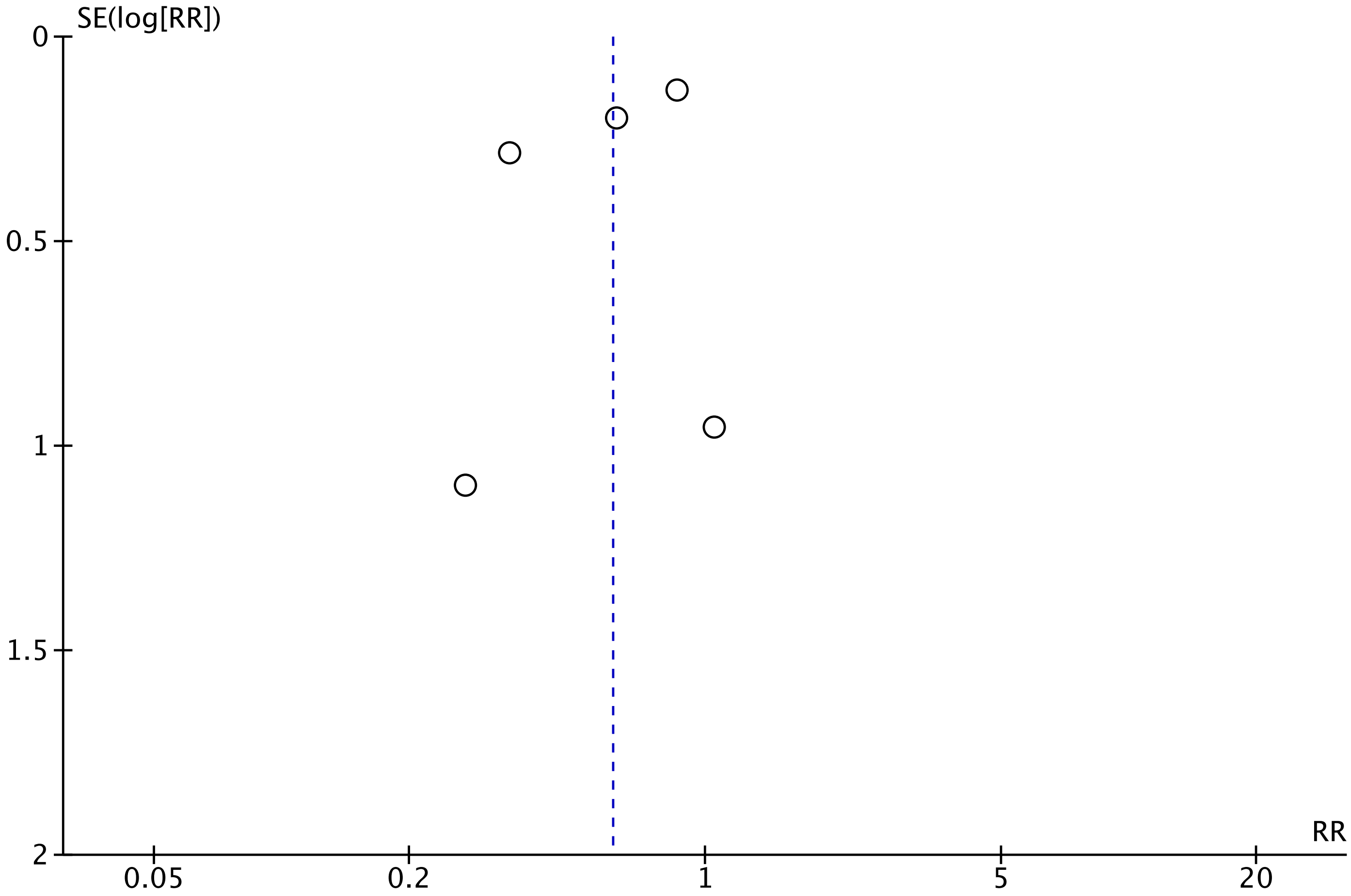

Atelectasis

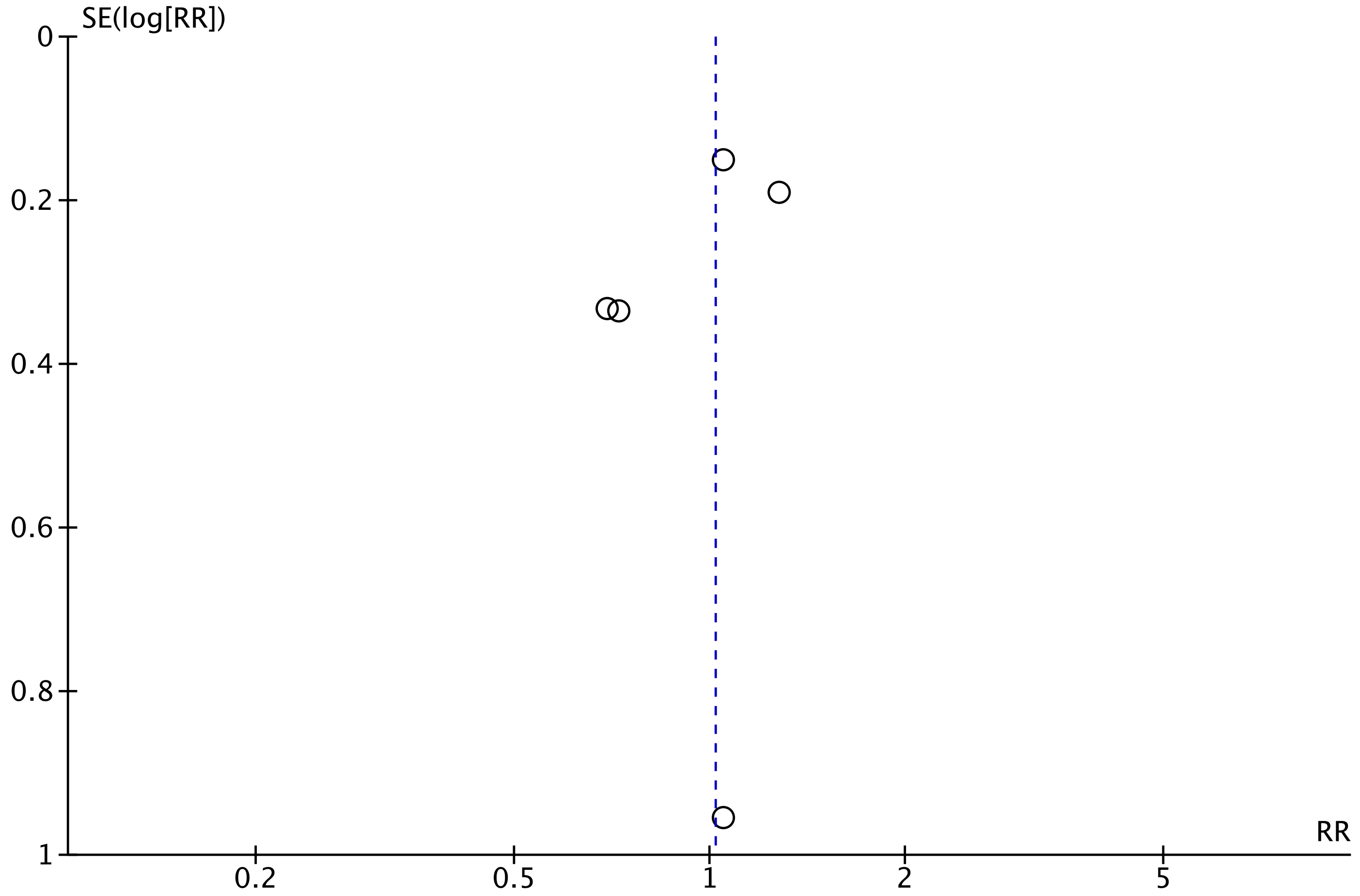

Barotrauma

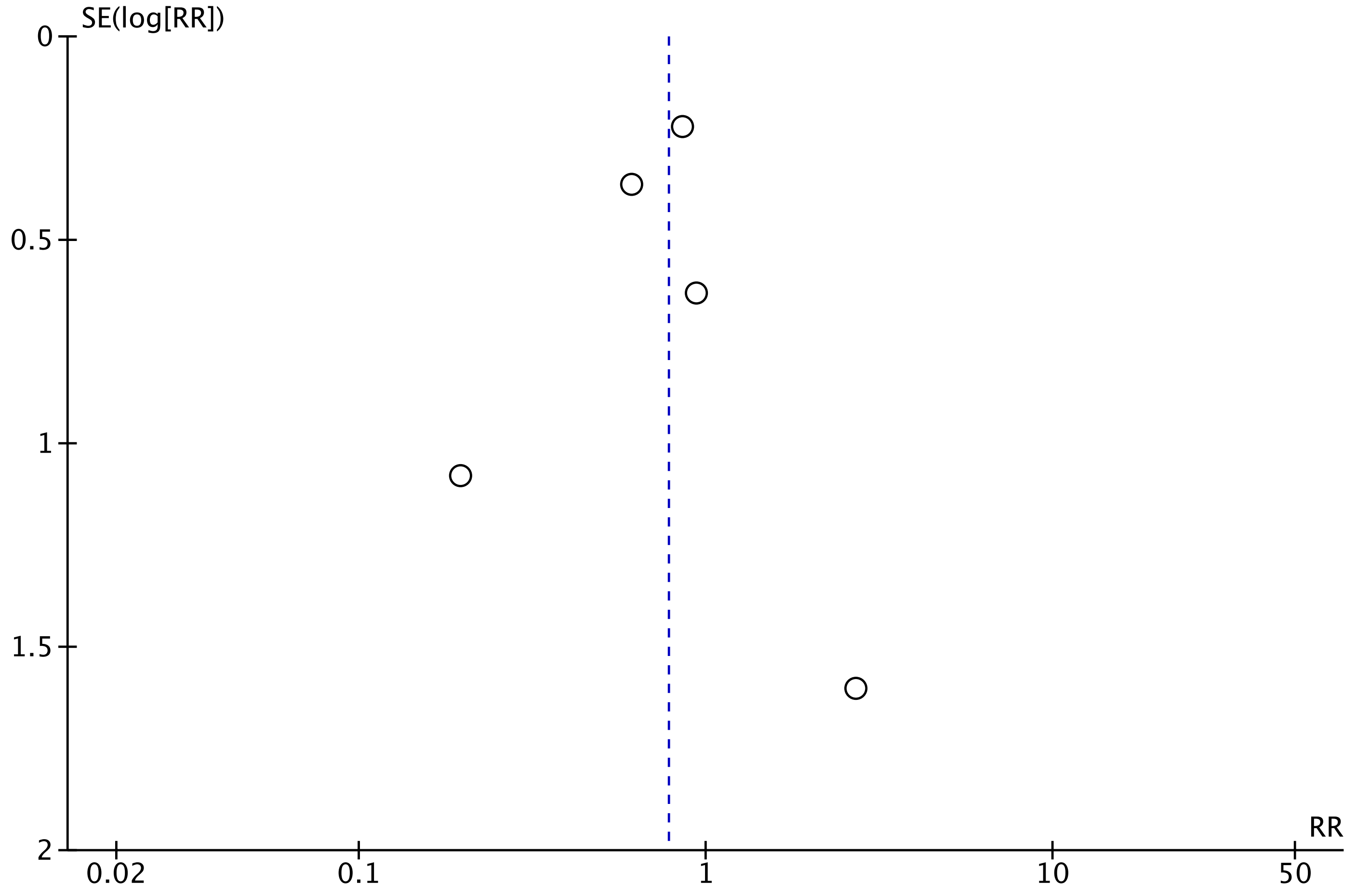

Ventilator-associated pneumonia

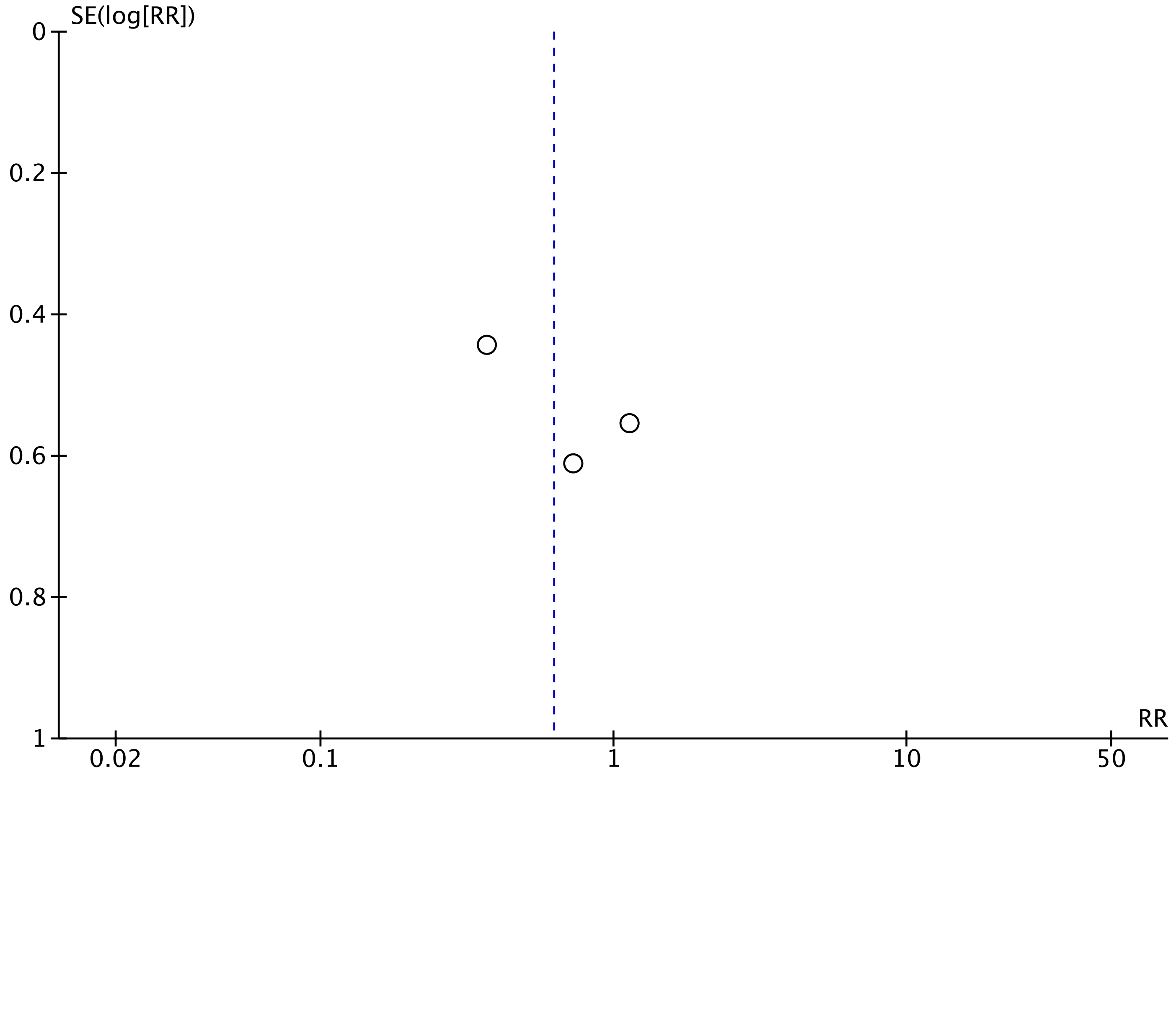

ARDS

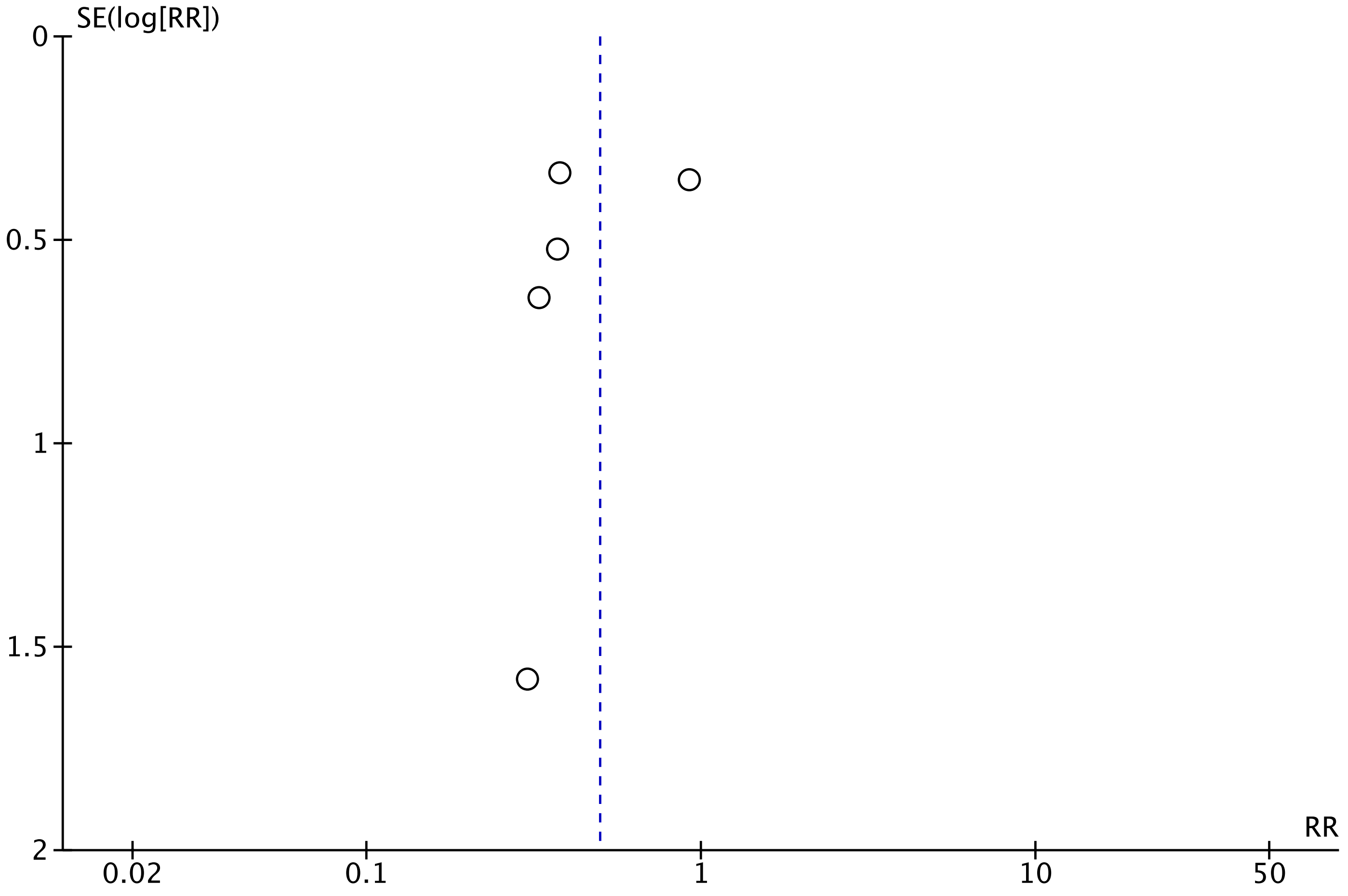

Hypotension

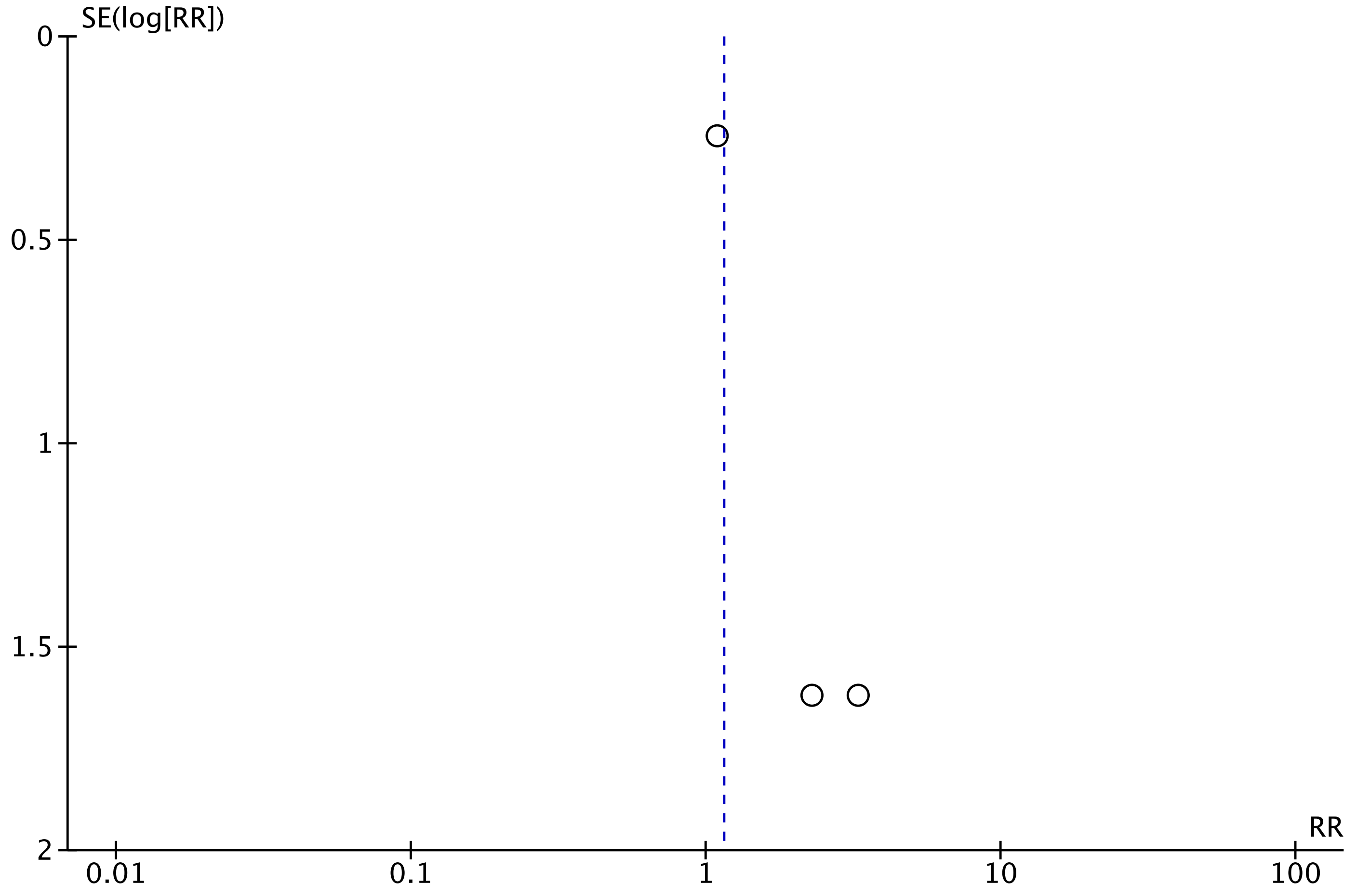

Cardiac index

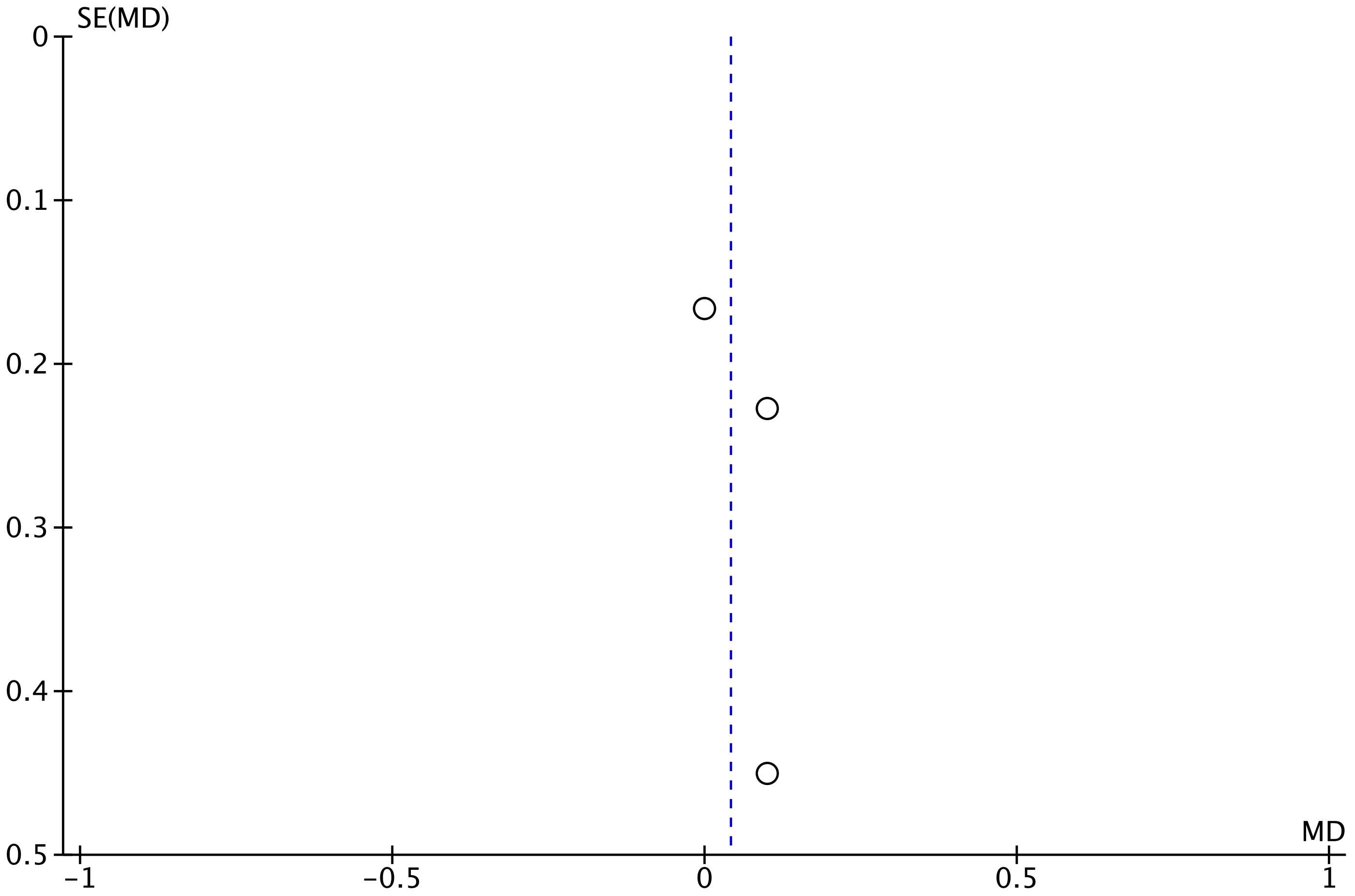

Central venous pressure

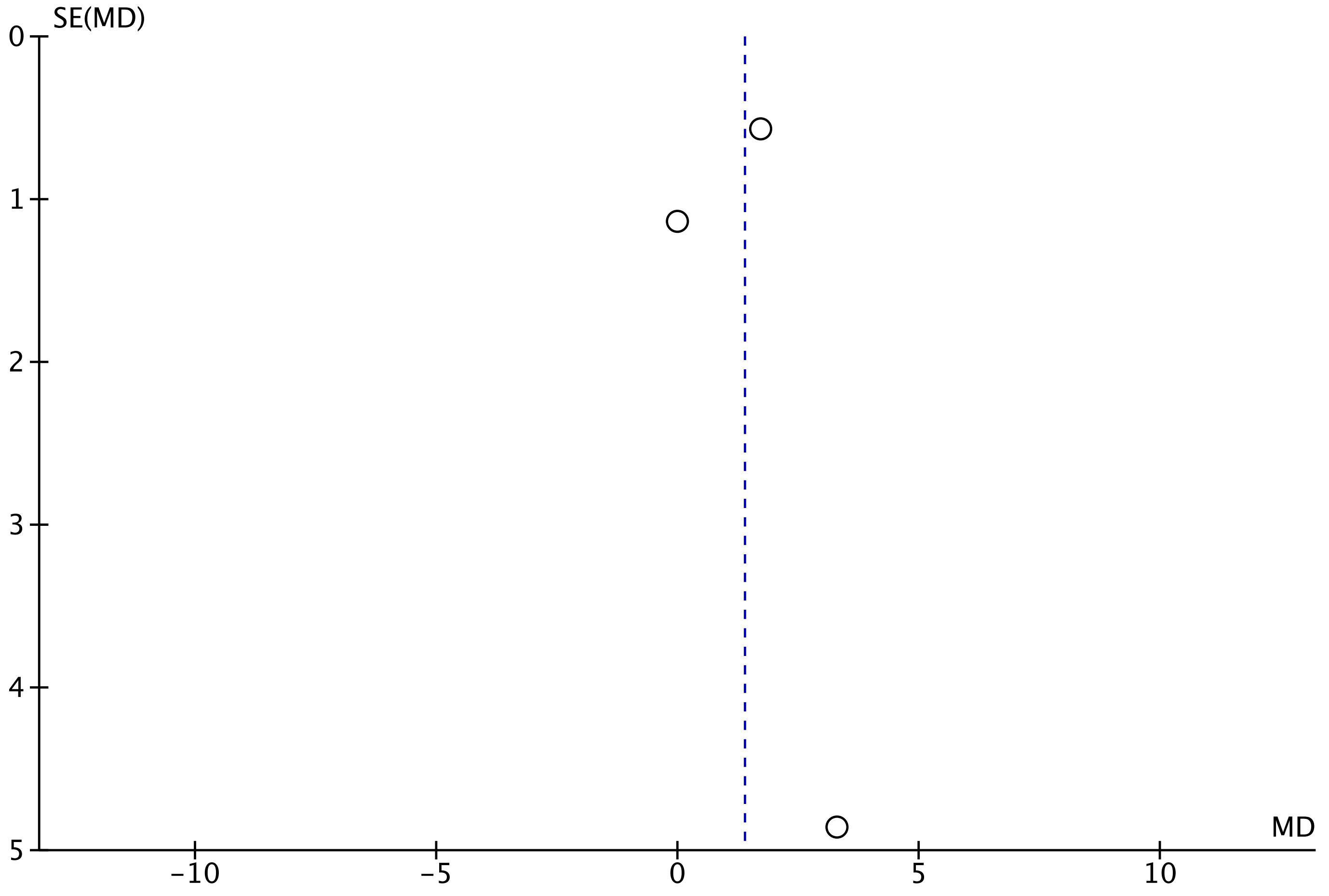

Postoperative bleeding

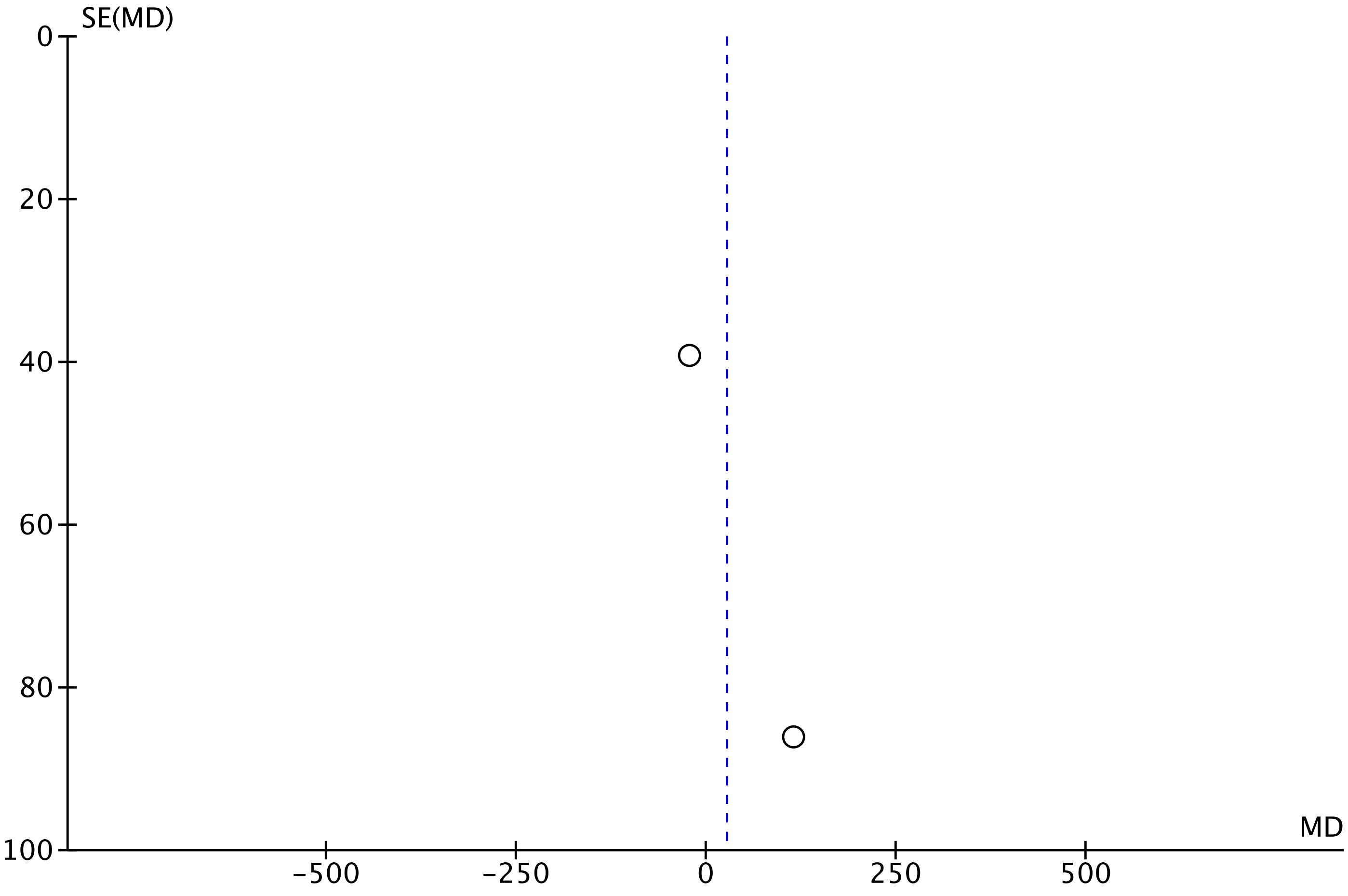

Packed red blood cell transfusion

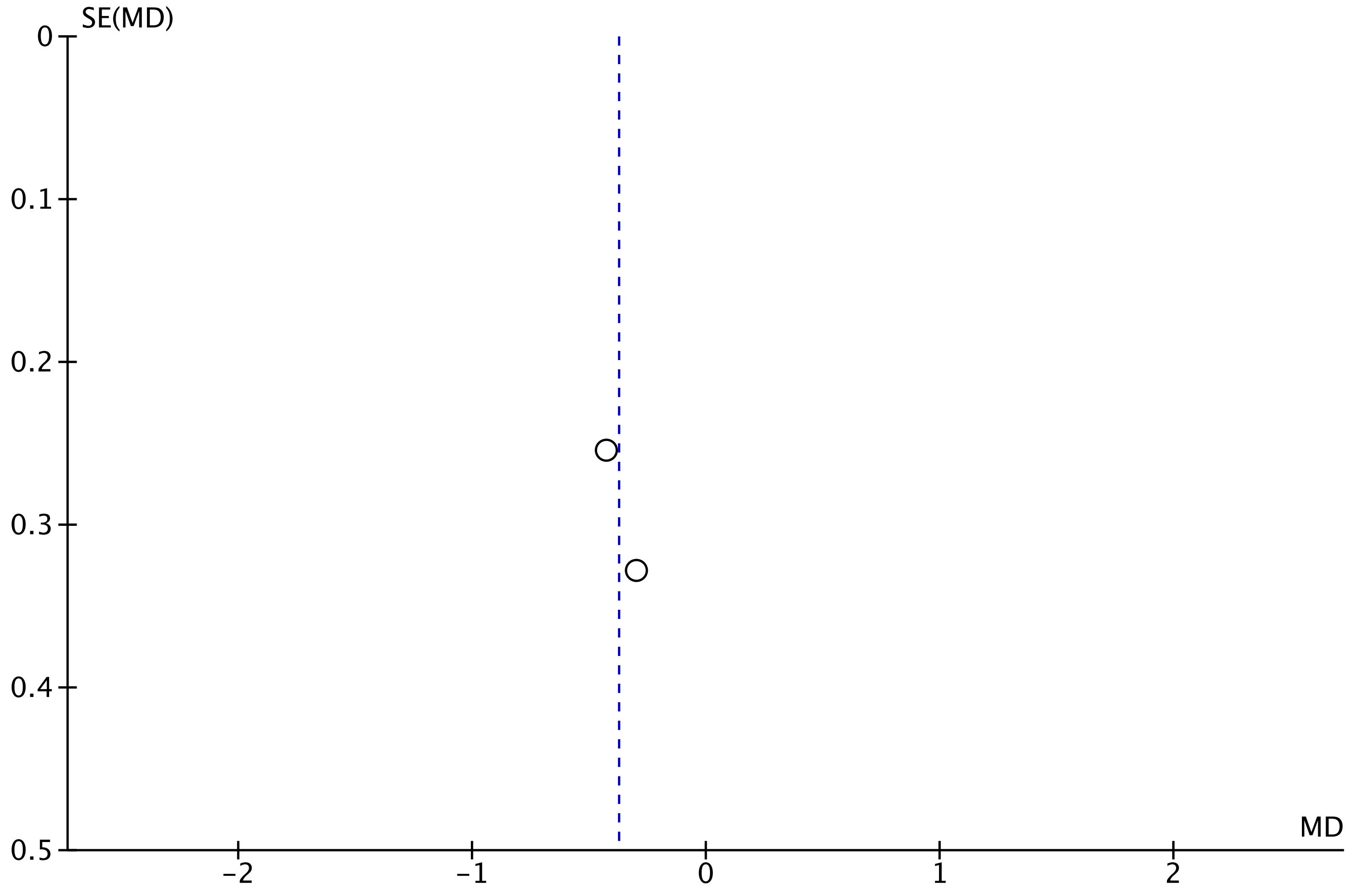

Duration of ventilation

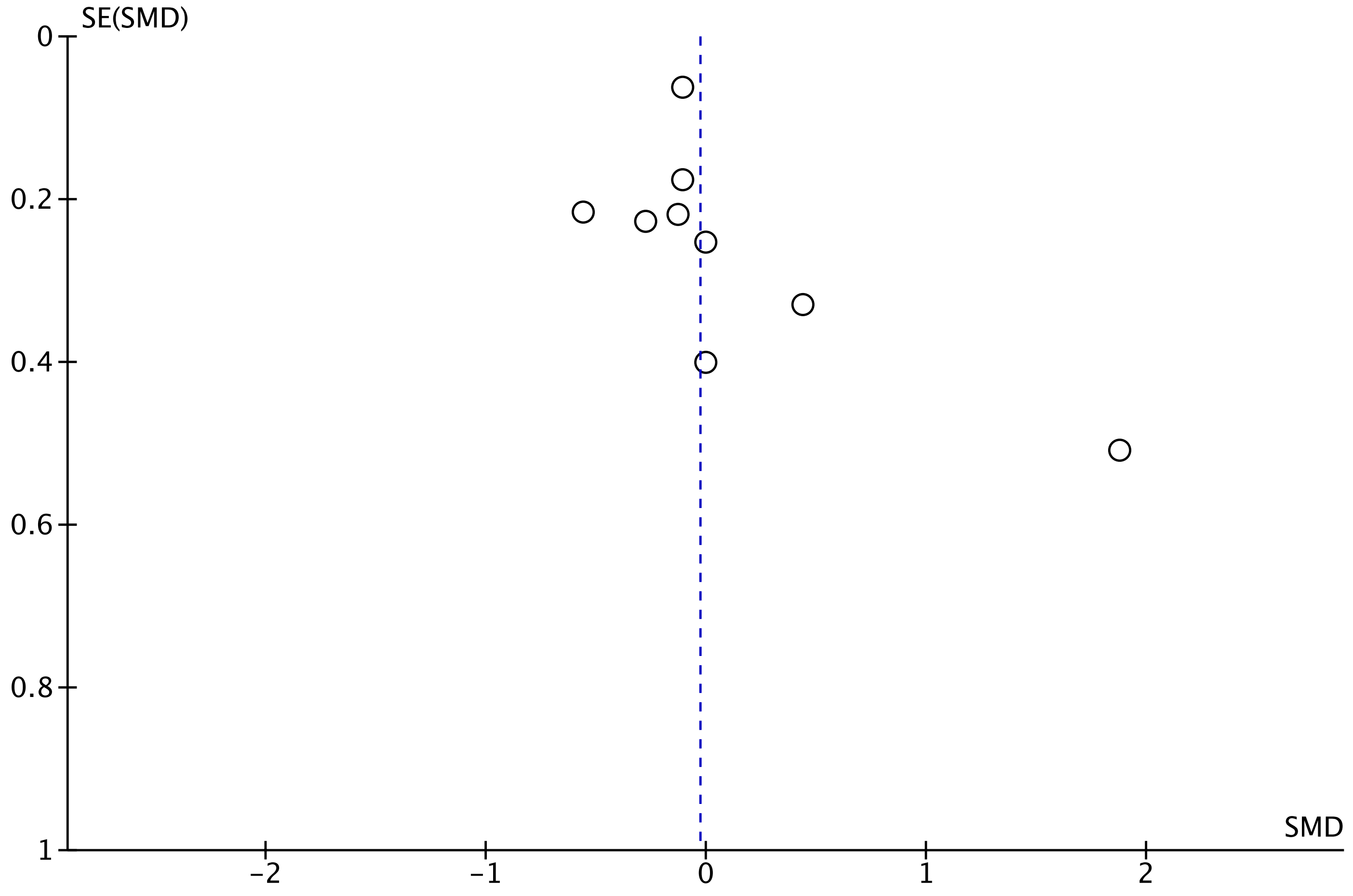

ICU stay

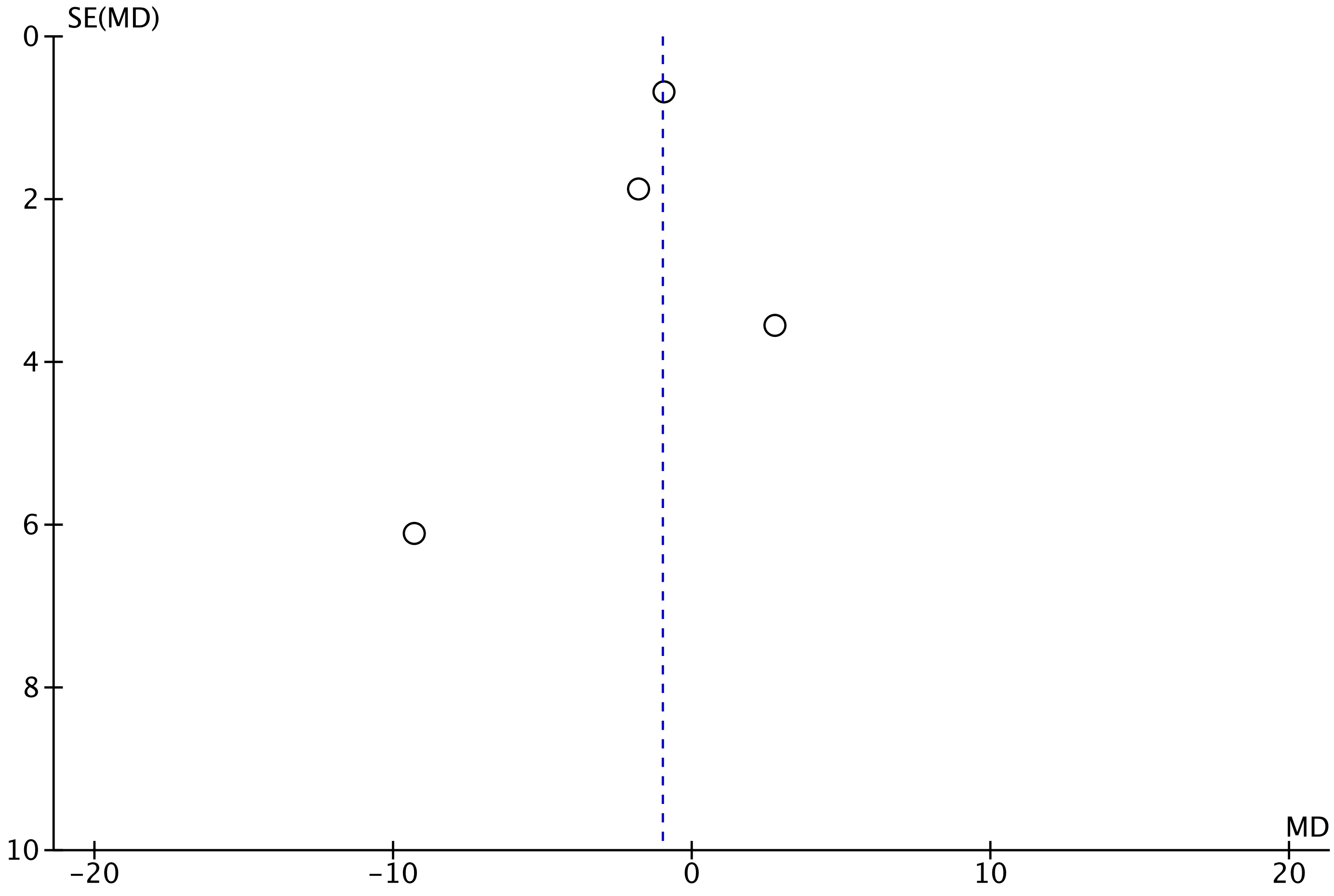

Hospital stay

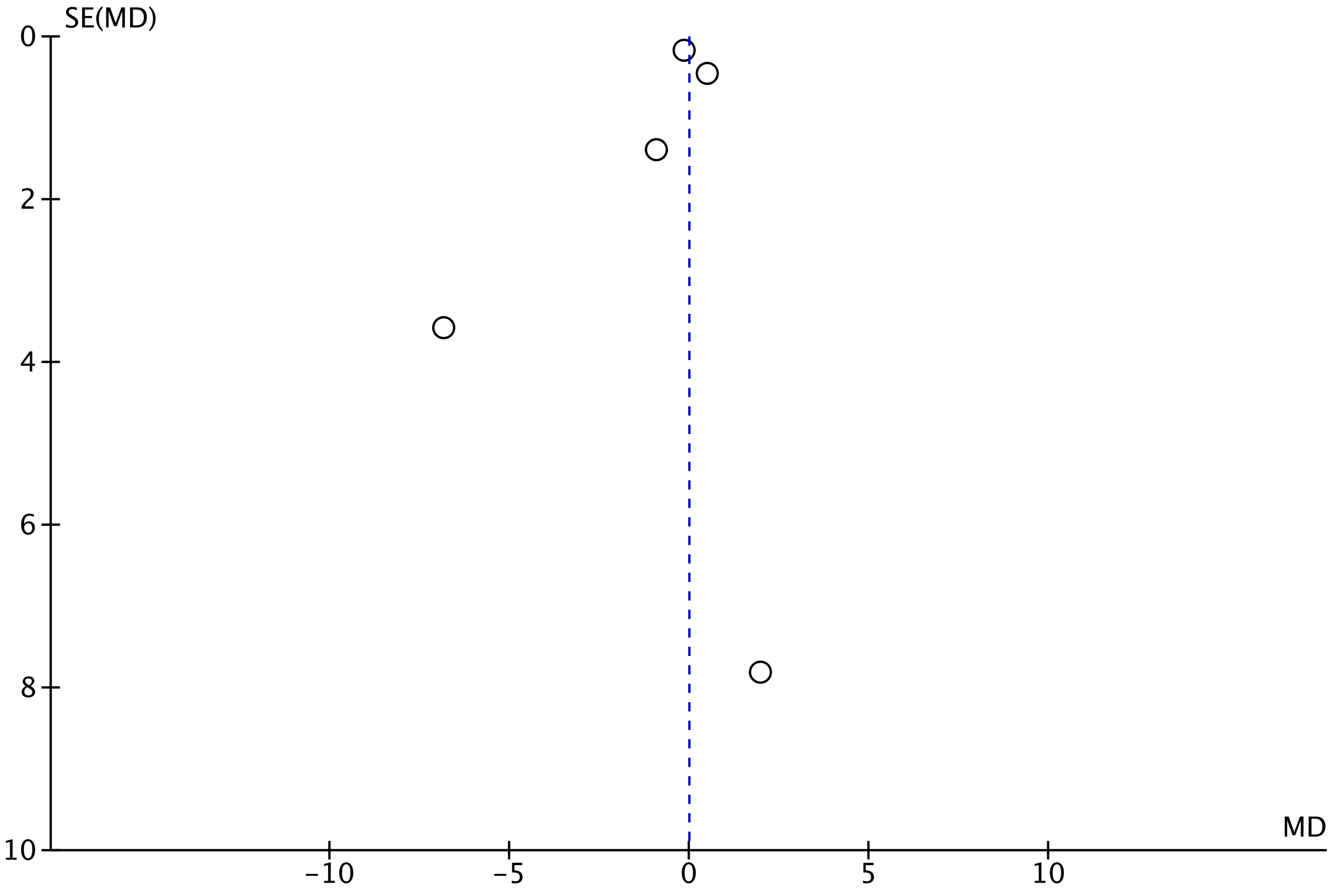

ICU mortality

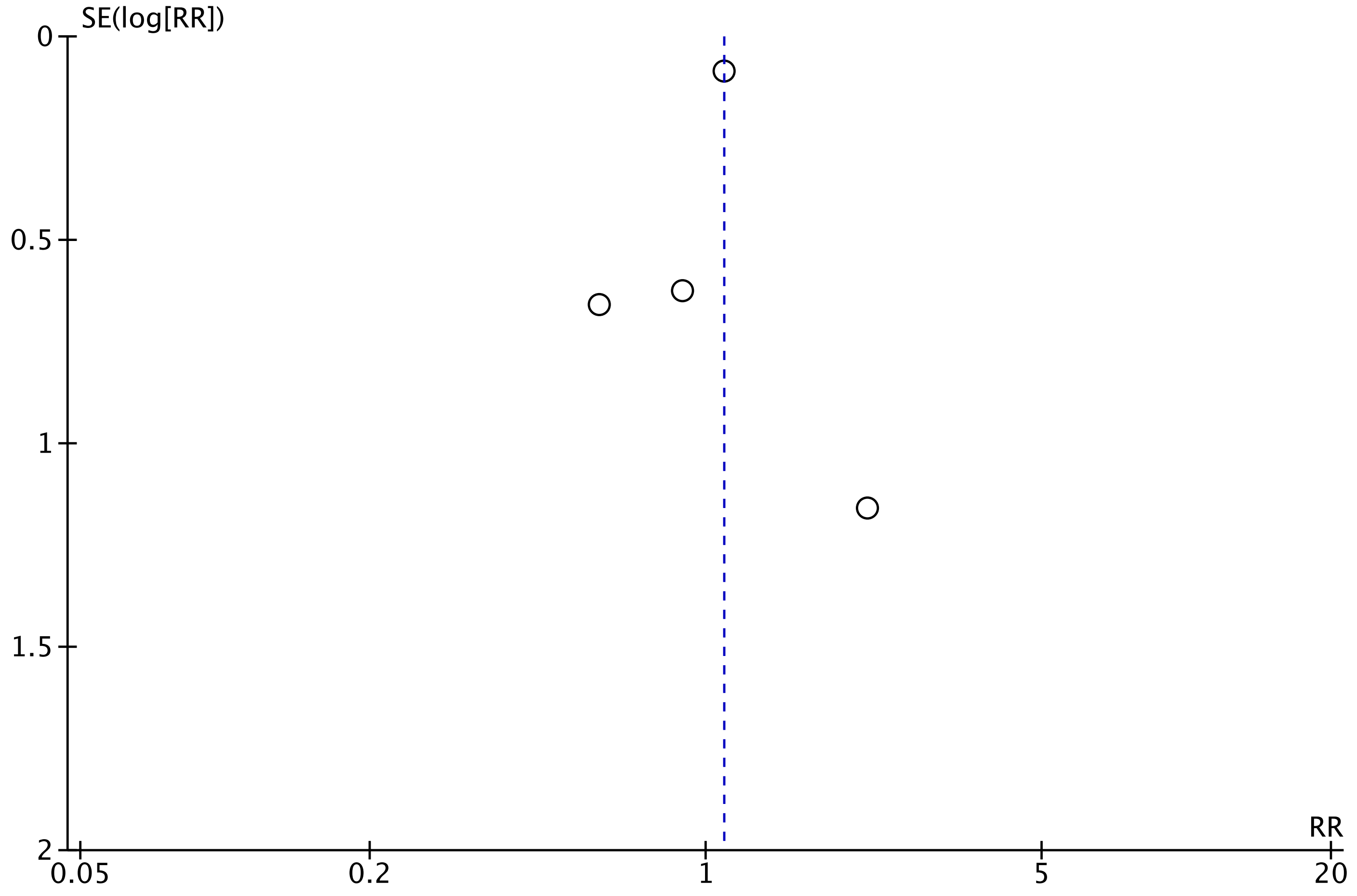

28-day mortality

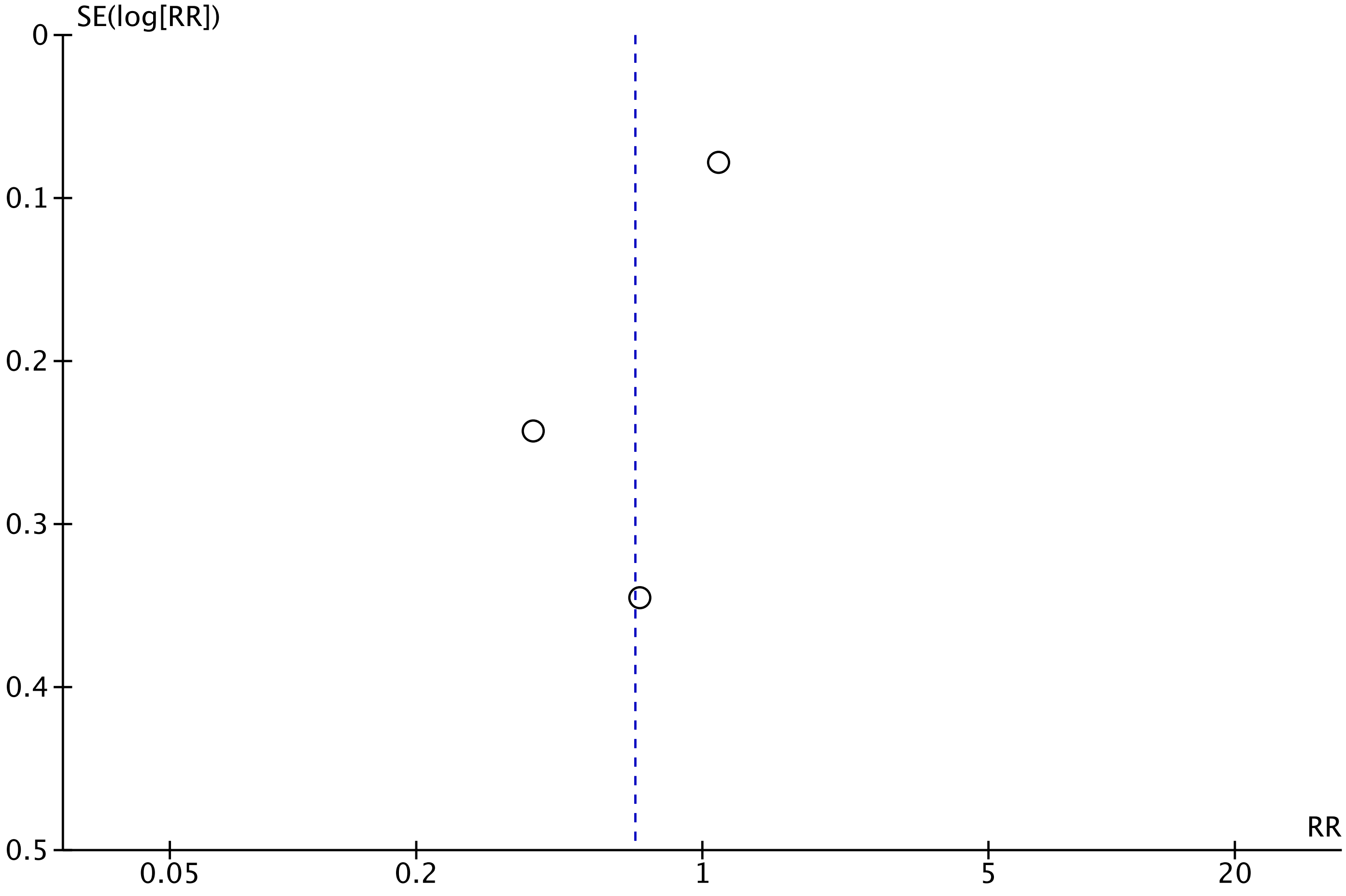

Online Resource 8. Sensitivity Analyses

Sensitivity analyses: timing of measurement

Sensitivity analyses according to the different timing of measurement of the variables in the following studies: Korovesi (day 1 and day 5), Manzano (basal, 6 hours, and day 1), and RELAx (after randomization, day 1, and day 2). Packed red blood cell transfusion became significantly lower in the higher PEEP group when considering this variable after randomization in the RELAx study.

Abbreviations: PRBC, packed red blood cell; afterrand, after randomization; IV, inverse variance; CI, confidence interval; d1, day 1; d2, day 2; 6 h, 6 hours; d5, day 5.

Outcome or Subgroup	Studies	Participants	Statistical Method	Effect Estimate
1.1 PRBC transfusion (relax_afterrand)	3	1138	Mean Difference (IV, Random, 95% CI)	-0.39 [-0.64, -0.14]
1.2 PRBC transfusion (relax_d1)	3	1138	Mean Difference (IV, Random, 95% CI)	-0.11 [-0.58, 0.36]
1.3 PRBC transfusion (relax_d2)	3	1138	Mean Difference (IV, Random, 95% CI)	-0.13 [-0.41, 0.14]

Outcome or Subgroup	Studies	Participants	Statistical Method	Effect Estimate
1.4 PaO_2/FiO_2 (korovesi_d1-manzano_basal-relax_afterrand)	8	1444	Mean Difference (IV, Random, 95% CI)	28.60 [3.69, 53.50]
1.5 PaO_2/FiO_2 (korovesi_d1-manzano_basal-relax_d1)	8	1444	Mean Difference (IV, Random, 95% CI)	37.18 [17.84, 56.53]

1.6 PaO_2/FiO_2 (korovesi_d1-manzano_basal-relax_d2)	8	1444	Mean Difference (IV, Random, 95% CI)	36.37 [17.09, 55.66]
1.7 PaO_2/FiO_2 (korovesi_d1-manzano_6h-relax_afterrand)	8	1444	Mean Difference (IV, Random, 95% CI)	29.70 [4.84, 54.56]
1.8 PaO_2/FiO_2 (korovesi_d1-manzano_6h-relax_d1)	8	1444	Mean Difference (IV, Random, 95% CI)	38.95 [20.20, 57.70]
1.9 PaO_2/FiO_2 (korovesi_d1-manzano_6h-relax_d2)	8	1444	Mean Difference (IV, Random, 95% CI)	38.01 [19.22, 56.81]
1.10 PaO_2/FiO_2 (korovesi_d1-manzano_d1-relax_afterrand)	8	1444	Mean Difference (IV, Random, 95% CI)	33.94 [9.58, 58.29]
1.11 PaO_2/FiO_2 (korovesi_d1-manzano_d1-relax_d1)	8	1444	Mean Difference (IV, Random, 95% CI)	43.71 [26.22, 61.20]
1.12 PaO_2/FiO_2 (korovesi_d1-manzano_d1-relax_d2)	8	1444	Mean Difference (IV, Random, 95% CI)	42.56 [24.81, 60.31]
1.13 PaO_2/FiO_2 (korovesi_d5-manzano_basal-relax_afterrand)	8	1444	Mean Difference (IV, Random, 95% CI)	28.18 [3.27, 53.08]
1.14 PaO_2/FiO_2 (korovesi_d5-manzano_basal-relax_d1)	8	1444	Mean Difference (IV, Random, 95% CI)	36.82 [17.43, 56.20]
1.15 PaO_2/FiO_2 (korovesi_d5-manzano_basal-relax_d2)	8	1444	Mean Difference (IV, Random, 95% CI)	36.01 [16.70, 55.33]

1.16 PaO_2/FiO_2 (korovesi_d5-manzano_6h-relax_afterrand)	8	1444	Mean Difference (IV, Random, 95% CI)	29.28 [4.41, 54.14]
1.17 PaO_2/FiO_2 (korovesi_d5-manzano_6h-relax_d1)	8	1444	Mean Difference (IV, Random, 95% CI)	38.58 [19.78, 57.38]
1.18 PaO_2/FiO_2 (korovesi_d5-manzano_6h-relax_d2)	8	1444	Mean Difference (IV, Random, 95% CI)	37.65 [18.82, 56.49]
1.19 PaO_2/FiO_2 (korovesi_d5-manzano_d1-relax_afterrand)	8	1444	Mean Difference (IV, Random, 95% CI)	33.51 [9.15, 57.88]
1.20 PaO_2/FiO_2 (korovesi_d5-manzano_d1-relax_d1)	8	1444	Mean Difference (IV, Random, 95% CI)	43.35 [25.80, 60.91]
1.21 PaO_2/FiO_2 (korovesi_d5-manzano_d1-relax_d2)	8	1444	Mean Difference (IV, Random, 95% CI)	42.22 [24.41, 60.02]
1.22 PaO_2/FiO_2 (korovesi_d1)	8	1444	Mean Difference (IV, Random, 95% CI)	48.05 [31.50, 64.60]
1.21 PaO_2/FiO_2 (korovesi_d5)	8	1444	Mean Difference (IV, Random, 95% CI)	47.68 [31.04, 64.32]
1.22 PaO_2/FiO_2 (manzano_basal)	8	1444	Mean Difference (IV, Random, 95% CI)	43.04 [24.13, 61.95]
1.23 PaO_2/FiO_2 (manzano_6h)	8	1444	Mean Difference (IV, Random, 95% CI)	44.98 [26.84, 63.12]
1.24 PaO_2/FiO_2 (manzano_d1)	8	1444	Mean Difference (IV, Random, 95% CI)	49.78 [33.24, 66.32]
1.25 PaO_2/FiO_2 (relax_afterrand)	8	1444	Mean Difference (IV, Random, 95% CI)	38.26 [13.53, 62.99]

1.26 PaO_2/FiO_2 (relax_d1)	8	1444	Mean Difference (IV, Random, 95% CI)	46.91 [29.19, 64.62]
1.27 PaO_2/FiO_2 (relax_d2)	8	1444	Mean Difference (IV, Random, 95% CI)	45.79 [27.74, 63.85]

Sensitivity analyses: odds ratio

Sensitivity analyses according to the use of odds ratio instead of risk ratio as effect estimate in dichotomous variables. No difference with respect to the main meta-analyses was observed.

Abbreviations: M-H, Mantel–Haenszel; CI, confidence interval; ARDS, acute respiratory distress syndrome; ICU, intensive care unit.

Outcome or Subgroup	Studies	Participants	Statistical Method	Effect Estimate
2.1 Hospital mortality	9	1502	Odds Ratio (M-H, Random, 95% CI)	1.03 [0.83, 1.29]
2.2 Hypoxemia	5	1320	Odds Ratio (M-H, Random, 95% CI)	0.43 [0.20, 0.94]
2.3 Atelectasis	5	1255	Odds Ratio (M-H, Random, 95% CI)	0.91 [0.59, 1.40]
2.4 Barotrauma	7	1372	Odds Ratio (M-H, Random, 95% CI)	0.67 [0.41, 1.10]
2.5 Ventilator-associated pneumonia	3	1188	Odds Ratio (M-H, Random, 95% CI)	0.60 [0.27, 1.35]
2.6 ARDS	6	1315	Odds Ratio (M-H, Random, 95% CI)	0.40 [0.23, 0.70]
2.7 Hypotension	5	283	Odds Ratio (M-H, Random, 95% CI)	1.39 [0.56, 3.44]
2.8 ICU mortality	5	1073	Odds Ratio (M-H, Random, 95% CI)	1.14 [0.88, 1.47]

2.9 28-day mortality	3	1152	Odds Ratio (M-H, Random, 95% CI)	0.51 [0.15, 1.77]

Sensitivity analyses: without studies at high risk of bias

Sensitivity analyses after removing the studies at high risk of bias. Central venous pressure and the incidence of hypoxemia became not significantly different between the 2 groups. We observed a trend ($p = 0.09$) towards a significantly decreased duration of ventilation with higher PEEP.

Abbreviations: M-H, Mantel–Haenszel; CI, confidence interval; PaO_2/FiO_2, arterial partial pressure of oxygen to fraction of inspired oxygen ratio; IV, inverse variance; ARDS, acute respiratory distress syndrome; CVP, central venous pressure; ICU, intensive care unit.

Outcome or Subgroup	Studies	Participants	Statistical Method	Effect Estimate
3.1 Hospital mortality	4	1235	Risk Ratio (M-H, Random, 95% CI)	1.07 [0.93, 1.24]
3.2 PaO_2/FiO_2	6	1334	Mean Difference (IV, Random, 95% CI)	56.55 [42.12, 70.98]
3.3 Hypoxemia	2	1096	Risk Ratio (M-H, Random, 95% CI)	0.57 [0.23, 1.37]
3.4 Atelectasis	2	1096	Risk Ratio (M-H, Random, 95% CI)	0.71 [0.45, 1.13]
3.5 Barotrauma	2	1096	Risk Ratio (M-H, Random, 95% CI)	0.54 [0.28, 1.07]
3.6 Ventilator-associated pneumonia	2	1096	Risk Ratio (M-H, Random, 95% CI)	0.61 [0.21, 1.83]
3.7 ARDS	2	1096	Risk Ratio (M-H, Random, 95% CI)	0.35 [0.16, 0.78]
3.8 Cardiac index	2	112	Mean Difference (IV, Random, 95% CI)	0.04 [-0.23, 0.30]
3.9 CVP	2	91	Mean Difference (IV, Random, 95% CI)	1.13 [-0.44, 2.70]

3.10 Duration of ventilation	4	1243	Std. Mean Difference (IV, Random, 95% CI)	-0.10 [-0.21, 0.01]
3.11 ICU stay	3	1123	Mean Difference (IV, Random, 95% CI)	-0.88 [-2.15, 0.38]
3.12 Hospital stay	3	1180	Mean Difference (IV, Random, 95% CI)	-0.73 [-3.21, 1.74]
3.13 ICU mortality	2	995	Risk Ratio (M-H, Random, 95% CI)	1.09 [0.92, 1.28]
3.14 28-day mortality	3	1152	Risk Ratio (M-H, Random, 95% CI)	0.68 [0.33, 1.40]

Forest plots of sensitivity analyses

Sensitivity analysis (timing of measurement)

Packed red blood cell transfusion (relax_afterrand)

	Higher PEEP			Lower PEEP				Mean Difference
**Study or Subgroup**	**Mean**	**SD**	**Total**	**Mean**	**SD**	**Total**	**Weight**	**IV, Random, 95% CI**
Collier 2002	0.8	1.4	40	1.1	1.6	44	14.8%	−0.30 [−0.94, 0.34]
Relax 2020	2.3	2.2	493	2.7	2.8	476	60.5%	−0.40 [−0.72, −0.08]
Zurich 1982	0.33	0.87	41	0.75	1.42	44	24.7%	−0.42 [−0.92, 0.08]
**Total (95% CI)**			**574**			**564**	**100.0%**	**−0.39 [−0.64, −0.14]**

Heterogeneity: $Tau^2 = 0.00$; $Chi^2 = 0.09$, df = 2 (P = 0.95); $I^2 = 0\%$
Test for overall effect: Z = 3.09 (P = 0.002)

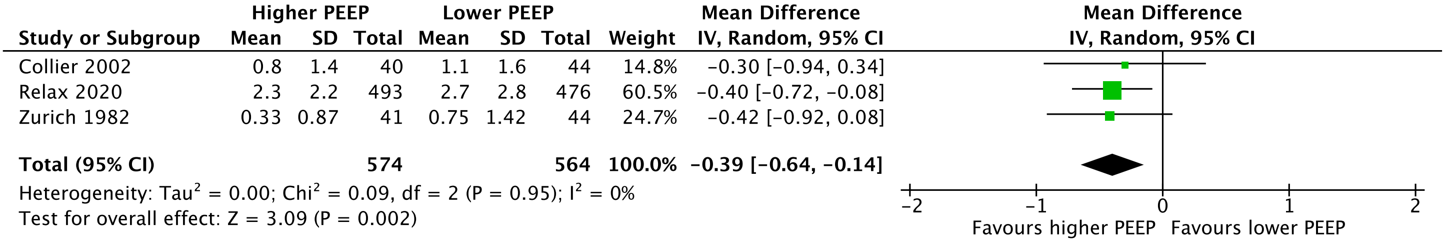

Sensitivity analysis (timing of measurement)

Packed red blood cell transfusion (relax_d1)

	Higher PEEP			Lower PEEP				Mean Difference
**Study or Subgroup**	**Mean**	**SD**	**Total**	**Mean**	**SD**	**Total**	**Weight**	**IV, Random, 95% CI**
Collier 2002	0.8	1.4	40	1.1	1.6	44	24.6%	−0.30 [−0.94, 0.34]
Relax 2020	1.5	0.7	493	1.3	0.7	476	45.2%	0.20 [0.11, 0.29]
Zurich 1982	0.33	0.87	41	0.75	1.42	44	30.2%	−0.42 [−0.92, 0.08]
**Total (95% CI)**			**574**			**564**	**100.0%**	**−0.11 [−0.58, 0.36]**

Heterogeneity: $Tau^2 = 0.12$; $Chi^2 = 7.92$, df = 2 (P = 0.02); $I^2 = 75\%$
Test for overall effect: Z = 0.46 (P = 0.64)

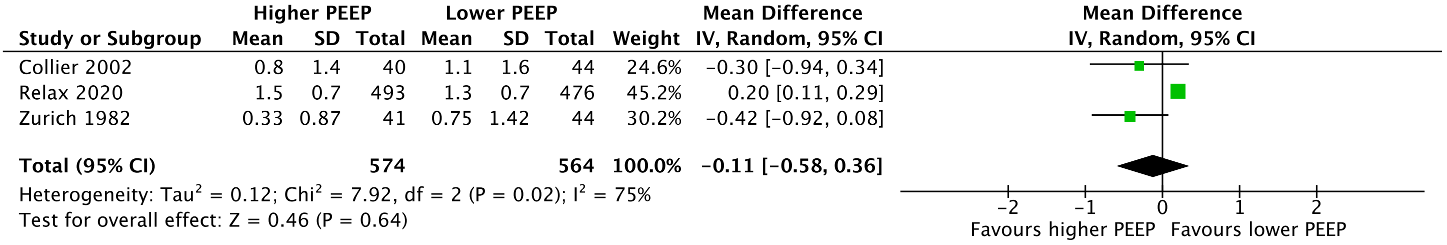

Sensitivity analysis (timing of measurement)

Packed red blood cell transfusion (relax_d2)

	Higher PEEP			Lower PEEP				Mean Difference
**Study or Subgroup**	**Mean**	**SD**	**Total**	**Mean**	**SD**	**Total**	**Weight**	**IV, Random, 95% CI**
Collier 2002	0.8	1.4	40	1.1	1.6	44	14.7%	−0.30 [−0.94, 0.34]
Relax 2020	1.3	0.7	493	1.3	0.7	476	63.8%	0.00 [−0.09, 0.09]
Zurich 1982	0.33	0.87	41	0.75	1.42	44	21.5%	−0.42 [−0.92, 0.08]
**Total (95% CI)**			**574**			**564**	**100.0%**	**−0.13 [−0.41, 0.14]**

Heterogeneity: $Tau^2 = 0.03$; $Chi^2 = 3.42$, df = 2 (P = 0.18); $I^2 = 41\%$
Test for overall effect: Z = 0.95 (P = 0.34)

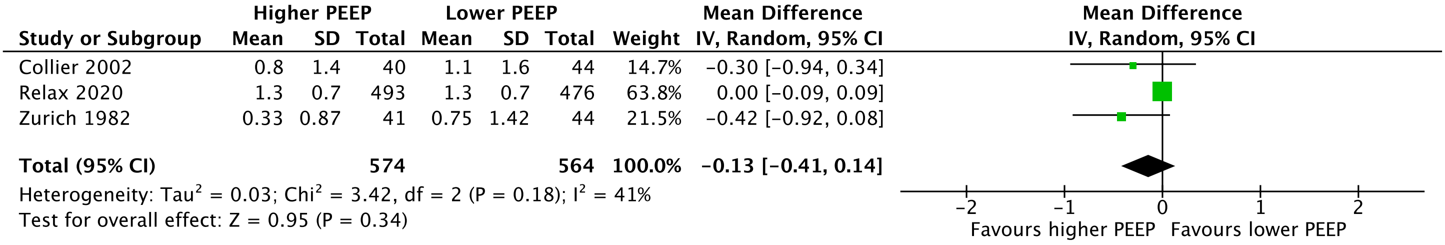

Sensitivity analysis (timing of measurement)

PaO2/FiO2 (korovesi_d1-manzano_basal-relax_afterrand)

	Higher PEEP			Lower PEEP				Mean Difference
**Study or Subgroup**	**Mean**	**SD**	**Total**	**Mean**	**SD**	**Total**	**Weight**	**IV, Random, 95% CI**
Holland 2007	307	82	14	337	82	14	8.9%	−30.00 [−90.75, 30.75]
Korovesi 2011	481	90	15	442	79	12	8.4%	39.00 [−24.81, 102.81]
Koutsoukou 2006	409	65	11	437	74	10	9.1%	−28.00 [−87.83, 31.83]
Lago Borges 2013	328	85	45	270	90	44	13.5%	58.00 [21.61, 94.39]
Lesur 2010	293	135	30	228	67	33	10.1%	65.00 [11.56, 118.44]
Ma 2014	196	45	60	134	22	60	18.1%	62.00 [49.33, 74.67]
Manzano 2008	392	104	64	375	79	63	14.4%	17.00 [−15.09, 49.09]
Relax 2020	230	140	493	216	124	476	17.5%	14.00 [−2.64, 30.64]
**Total (95% CI)**			**732**			**712**	**100.0%**	**28.60 [3.69, 53.50]**

Heterogeneity: $Tau^2 = 851.49$; $Chi^2 = 34.77$, df = 7 (P < 0.0001); $I^2 = 80\%$
Test for overall effect: Z = 2.25 (P = 0.02)

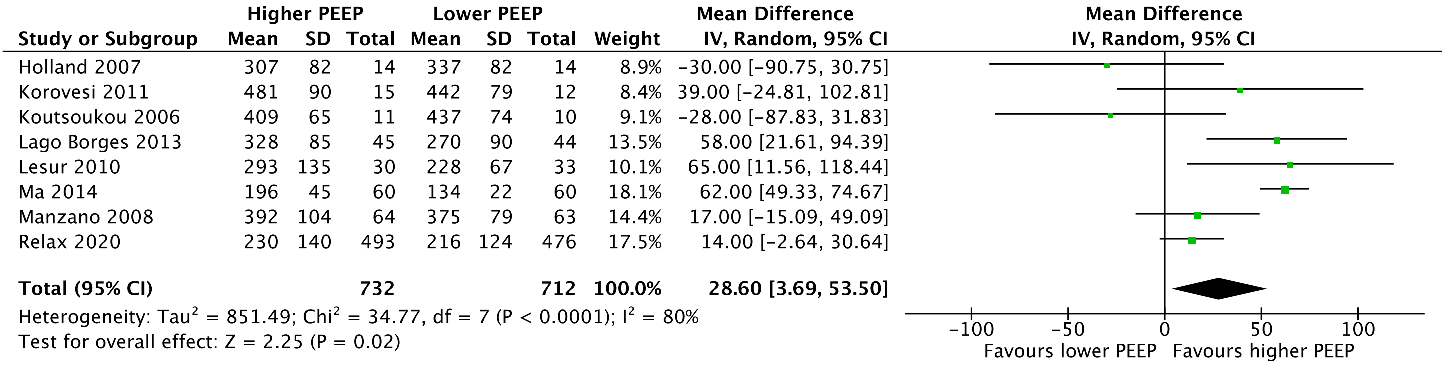

Sensitivity analysis (timing of measurement)

PaO2/FiO2 (korovesi_d1-manzano_basal-relax_d1)

	Higher PEEP			Lower PEEP				Mean Difference
**Study or Subgroup**	**Mean**	**SD**	**Total**	**Mean**	**SD**	**Total**	**Weight**	**IV, Random, 95% CI**
Holland 2007	307	82	14	337	82	14	7.1%	−30.00 [−90.75, 30.75]
Korovesi 2011	481	90	15	442	79	12	6.6%	39.00 [−24.81, 102.81]
Koutsoukou 2006	409	65	11	437	74	10	7.3%	−28.00 [−87.83, 31.83]
Lago Borges 2013	328	85	45	270	90	44	13.0%	58.00 [21.61, 94.39]
Lesur 2010	293	135	30	228	67	33	8.5%	65.00 [11.56, 118.44]
Ma 2014	196	45	60	134	22	60	21.8%	62.00 [49.33, 74.67]
Manzano 2008	392	104	64	375	79	63	14.5%	17.00 [−15.09, 49.09]
Relax 2020	272	126	493	226	101	476	21.2%	46.00 [31.65, 60.35]
**Total (95% CI)**			**732**			**712**	**100.0%**	**37.18 [17.84, 56.53]**

Heterogeneity: $Tau^2 = 405.32$; $Chi^2 = 21.56$, df = 7 (P = 0.003); $I^2 = 68\%$
Test for overall effect: Z = 3.77 (P = 0.0002)

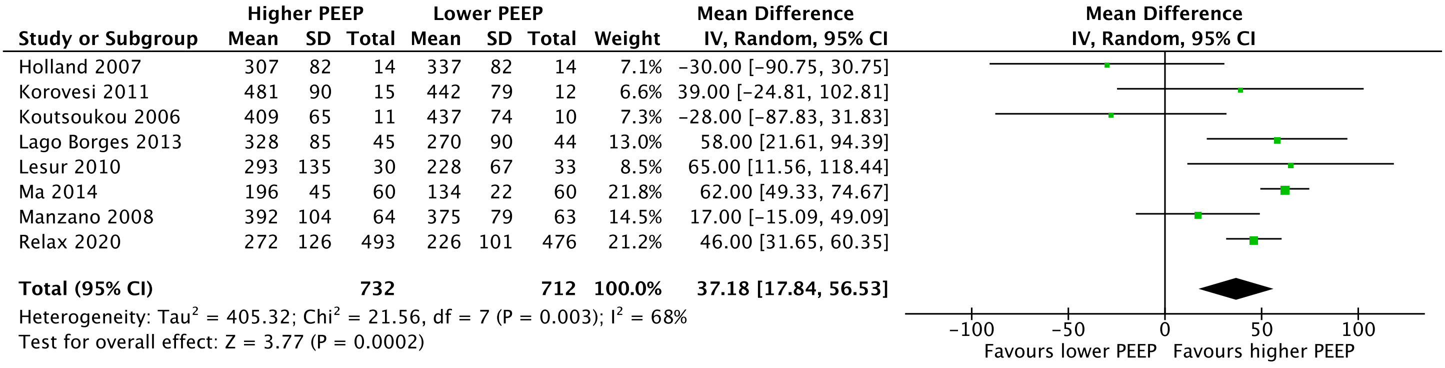

Sensitivity analysis (timing of measurement)

PaO2/FiO2 (korovesi_d1-manzano_basal-relax_d2)

	Higher PEEP			Lower PEEP				Mean Difference	Mean Difference
**Study or Subgroup**	**Mean**	**SD**	**Total**	**Mean**	**SD**	**Total**	**Weight**	**IV, Random, 95% CI**	**IV, Random, 95% CI**
Holland 2007	307	82	14	337	82	14	7.1%	−30.00 [−90.75, 30.75]	
Korovesi 2011	481	90	15	442	79	12	6.6%	39.00 [−24.81, 102.81]	
Koutsoukou 2006	409	65	11	437	74	10	7.2%	−28.00 [−87.83, 31.83]	
Lago Borges 2013	328	85	45	270	90	44	12.9%	58.00 [21.61, 94.39]	
Lesur 2010	293	135	30	228	67	33	8.4%	65.00 [11.56, 118.44]	
Ma 2014	196	45	60	134	22	60	21.7%	62.00 [49.33, 74.67]	
Manzano 2008	392	104	64	375	79	63	14.4%	17.00 [−15.09, 49.09]	
Relax 2020	250	113	493	208	96	476	21.6%	42.00 [28.81, 55.19]	
**Total (95% CI)**			**732**			**712**	**100.0%**	**36.37 [17.09, 55.66]**	

Heterogeneity: $Tau^2 = 403.81$; $Chi^2 = 22.34$, df = 7 (P = 0.002); $I^2 = 69\%$
Test for overall effect: Z = 3.70 (P = 0.0002)

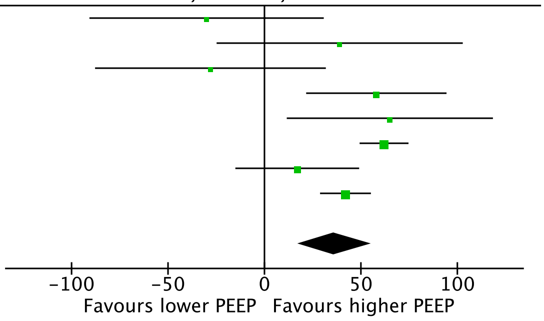

Sensitivity analysis (timing of measurement)

PaO2/FiO2 (korovesi_d1-manzano_6h-relax_afterrand)

	Higher PEEP			Lower PEEP				Mean Difference	Mean Difference
**Study or Subgroup**	**Mean**	**SD**	**Total**	**Mean**	**SD**	**Total**	**Weight**	**IV, Random, 95% CI**	**IV, Random, 95% CI**
Holland 2007	307	82	14	337	82	14	9.0%	−30.00 [−90.75, 30.75]	
Korovesi 2011	481	90	15	442	79	12	8.5%	39.00 [−24.81, 102.81]	
Koutsoukou 2006	409	65	11	437	74	10	9.1%	−28.00 [−87.83, 31.83]	
Lago Borges 2013	328	85	45	270	90	44	13.6%	58.00 [21.61, 94.39]	
Lesur 2010	293	135	30	228	67	33	10.2%	65.00 [11.56, 118.44]	
Ma 2014	196	45	60	134	22	60	18.3%	62.00 [49.33, 74.67]	
Manzano 2008	356	100	64	332	108	63	13.7%	24.00 [−12.21, 60.21]	
Relax 2020	230	140	493	216	124	476	17.7%	14.00 [−2.64, 30.64]	
**Total (95% CI)**			**732**			**712**	**100.0%**	**29.70 [4.84, 54.56]**	

Heterogeneity: $Tau^2 = 836.73$; $Chi^2 = 33.48$, df = 7 (P < 0.0001); $I^2 = 79\%$
Test for overall effect: Z = 2.34 (P = 0.02)

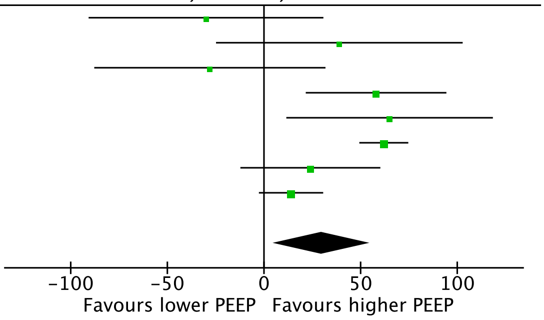

Sensitivity analysis (timing of measurement)

PaO2/FiO2 (korovesi_d1-manzano_6h-relax_d1)

	Higher PEEP			Lower PEEP				Mean Difference
**Study or Subgroup**	**Mean**	**SD**	**Total**	**Mean**	**SD**	**Total**	**Weight**	**IV, Random, 95% CI**
Holland 2007	307	82	14	337	82	14	6.9%	−30.00 [−90.75, 30.75]
Korovesi 2011	481	90	15	442	79	12	6.5%	39.00 [−24.81, 102.81]
Koutsoukou 2006	409	65	11	437	74	10	7.1%	−28.00 [−87.83, 31.83]
Lago Borges 2013	328	85	45	270	90	44	13.0%	58.00 [21.61, 94.39]
Lesur 2010	293	135	30	228	67	33	8.3%	65.00 [11.56, 118.44]
Ma 2014	196	45	60	134	22	60	22.9%	62.00 [49.33, 74.67]
Manzano 2008	356	100	64	332	108	63	13.1%	24.00 [−12.21, 60.21]
Relax 2020	272	126	493	226	101	476	22.2%	46.00 [31.65, 60.35]
**Total (95% CI)**			**732**			**712**	**100.0%**	**38.95 [20.20, 57.70]**

Heterogeneity: $Tau^2 = 358.05$; $Chi^2 = 19.52$, df = 7 (P = 0.007); $I^2 = 64\%$
Test for overall effect: Z = 4.07 (P < 0.0001)

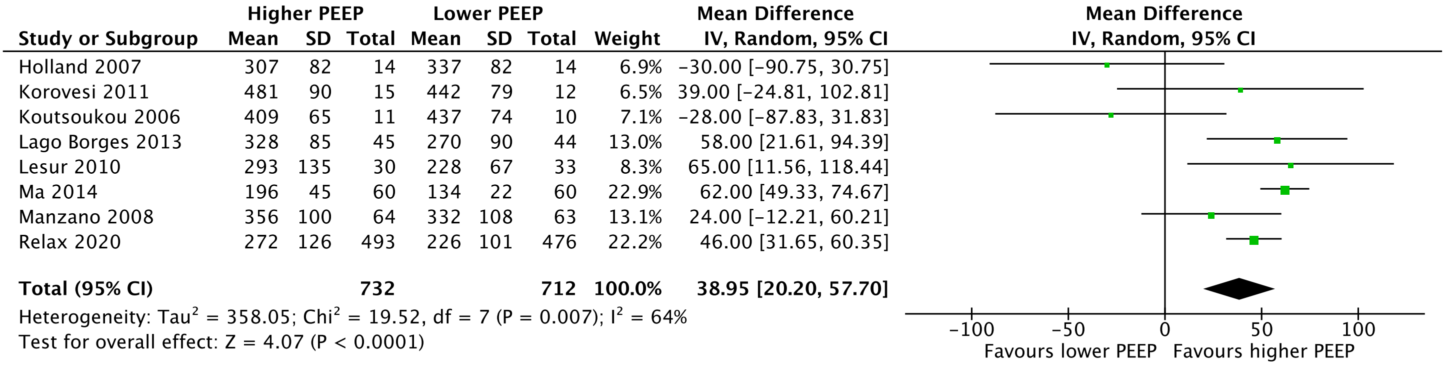

Sensitivity analysis (timing of measurement)

PaO2/FiO2 (korovesi_d1-manzano_6h-relax_d2)

	Higher PEEP			Lower PEEP				Mean Difference
**Study or Subgroup**	**Mean**	**SD**	**Total**	**Mean**	**SD**	**Total**	**Weight**	**IV, Random, 95% CI**
Holland 2007	307	82	14	337	82	14	6.9%	−30.00 [−90.75, 30.75]
Korovesi 2011	481	90	15	442	79	12	6.5%	39.00 [−24.81, 102.81]
Koutsoukou 2006	409	65	11	437	74	10	7.1%	−28.00 [−87.83, 31.83]
Lago Borges 2013	328	85	45	270	90	44	13.0%	58.00 [21.61, 94.39]
Lesur 2010	293	135	30	228	67	33	8.3%	65.00 [11.56, 118.44]
Ma 2014	196	45	60	134	22	60	22.7%	62.00 [49.33, 74.67]
Manzano 2008	356	100	64	332	108	63	13.0%	24.00 [−12.21, 60.21]
Relax 2020	250	113	493	208	96	476	22.5%	42.00 [28.81, 55.19]
**Total (95% CI)**			**732**			**712**	**100.0%**	**38.01 [19.22, 56.81]**

Heterogeneity: $Tau^2 = 363.63$; $Chi^2 = 20.46$, df = 7 (P = 0.005); $I^2 = 66\%$
Test for overall effect: Z = 3.96 (P < 0.0001)

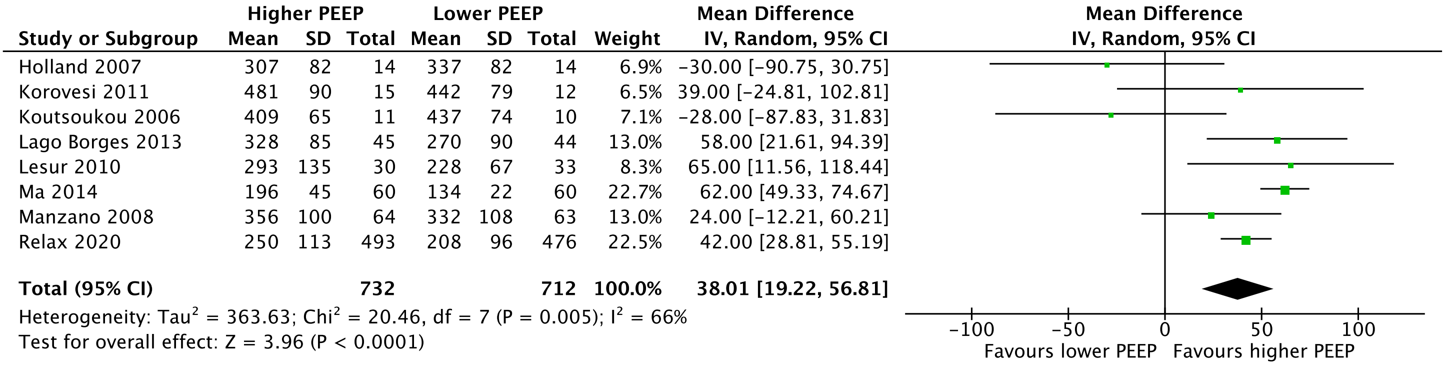

Sensitivity analysis (timing of measurement)

PaO2/FiO2 (korovesi_d1-manzano_d1-relax_afterrand)

	Higher PEEP			Lower PEEP				Mean Difference
**Study or Subgroup**	**Mean**	**SD**	**Total**	**Mean**	**SD**	**Total**	**Weight**	**IV, Random, 95% CI**
Holland 2007	307	82	14	337	82	14	8.8%	-30.00 [-90.75, 30.75]
Korovesi 2011	481	90	15	442	79	12	8.3%	39.00 [-24.81, 102.81]
Koutsoukou 2006	409	65	11	437	74	10	8.9%	-28.00 [-87.83, 31.83]
Lago Borges 2013	328	85	45	270	90	44	13.5%	58.00 [21.61, 94.39]
Lesur 2010	293	135	30	228	67	33	10.0%	65.00 [11.56, 118.44]
Ma 2014	196	45	60	134	22	60	18.4%	62.00 [49.33, 74.67]
Manzano 2008	362	101	64	309	86	63	14.4%	53.00 [20.39, 85.61]
Relax 2020	230	140	493	216	124	476	17.7%	14.00 [-2.64, 30.64]
**Total (95% CI)**			**732**			**712**	**100.0%**	**33.94 [9.58, 58.29]**

Heterogeneity: $Tau^2 = 798.91$; $Chi^2 = 32.94$, df = 7 ($P < 0.0001$); $I^2 = 79\%$
Test for overall effect: Z = 2.73 (P = 0.006)

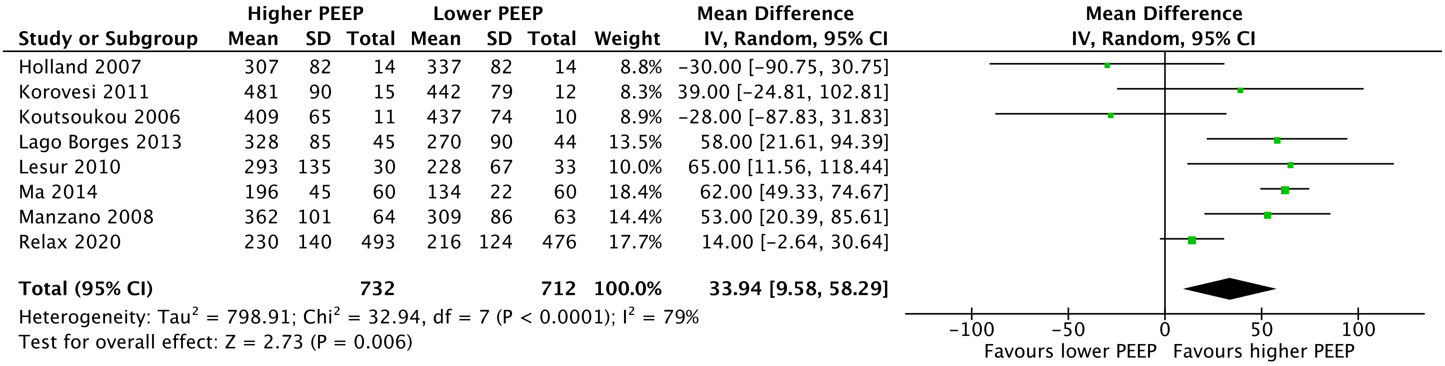

Sensitivity analysis (timing of measurement)

PaO2/FiO2 (korovesi_d1-manzano_d1-relax_d1)

	Higher PEEP			Lower PEEP				Mean Difference
**Study or Subgroup**	**Mean**	**SD**	**Total**	**Mean**	**SD**	**Total**	**Weight**	**IV, Random, 95% CI**
Holland 2007	307	82	14	337	82	14	6.4%	−30.00 [−90.75, 30.75]
Korovesi 2011	481	90	15	442	79	12	5.9%	39.00 [−24.81, 102.81]
Koutsoukou 2006	409	65	11	437	74	10	6.5%	−28.00 [−87.83, 31.83]
Lago Borges 2013	328	85	45	270	90	44	12.5%	58.00 [21.61, 94.39]
Lesur 2010	293	135	30	228	67	33	7.7%	65.00 [11.56, 118.44]
Ma 2014	196	45	60	134	22	60	23.9%	62.00 [49.33, 74.67]
Manzano 2008	362	101	64	309	86	63	14.0%	53.00 [20.39, 85.61]
Relax 2020	272	126	493	226	101	476	23.1%	46.00 [31.65, 60.35]
**Total (95% CI)**			**732**			**712**	**100.0%**	**43.71 [26.22, 61.20]**

Heterogeneity: $Tau^2 = 291.19$; $Chi^2 = 17.42$, df = 7 (P = 0.01); $I^2 = 60\%$
Test for overall effect: Z = 4.90 (P < 0.00001)

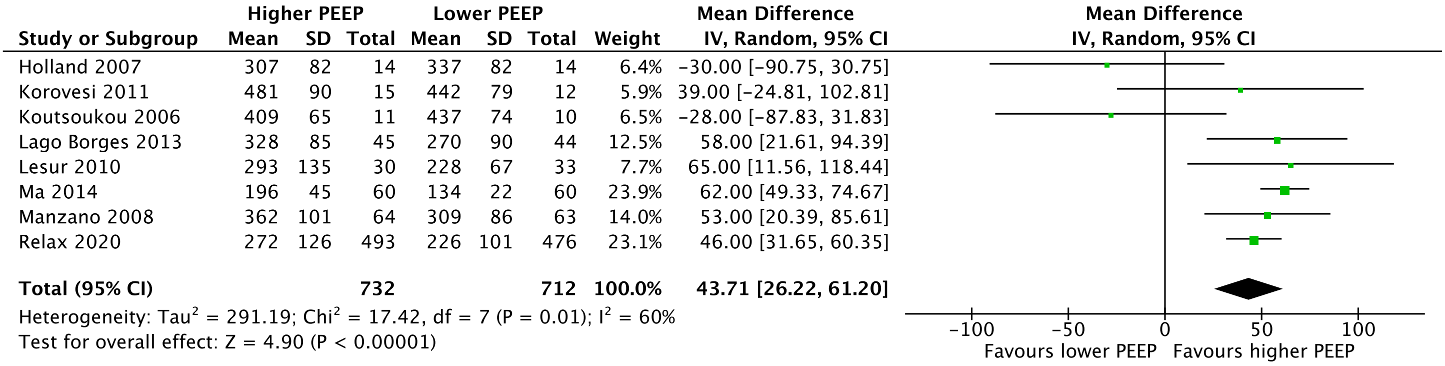

Sensitivity analysis (timing of measurement)

PaO2/FiO2 (korovesi_d1-manzano_d1-relax_d2)

	Higher PEEP			Lower PEEP				Mean Difference
**Study or Subgroup**	**Mean**	**SD**	**Total**	**Mean**	**SD**	**Total**	**Weight**	**IV, Random, 95% CI**
Holland 2007	307	82	14	337	82	14	6.5%	−30.00 [−90.75, 30.75]
Korovesi 2011	481	90	15	442	79	12	6.0%	39.00 [−24.81, 102.81]
Koutsoukou 2006	409	65	11	437	74	10	6.6%	−28.00 [−87.83, 31.83]
Lago Borges 2013	328	85	45	270	90	44	12.6%	58.00 [21.61, 94.39]
Lesur 2010	293	135	30	228	67	33	7.8%	65.00 [11.56, 118.44]
Ma 2014	196	45	60	134	22	60	23.4%	62.00 [49.33, 74.67]
Manzano 2008	362	101	64	309	86	63	14.0%	53.00 [20.39, 85.61]
Relax 2020	250	113	493	208	96	476	23.2%	42.00 [28.81, 55.19]
**Total (95% CI)**			**732**			**712**	**100.0%**	**42.56 [24.81, 60.31]**

Heterogeneity: $Tau^2 = 308.74$; $Chi^2 = 18.68$, df = 7 (P = 0.009); $I^2 = 63\%$
Test for overall effect: Z = 4.70 (P < 0.00001)

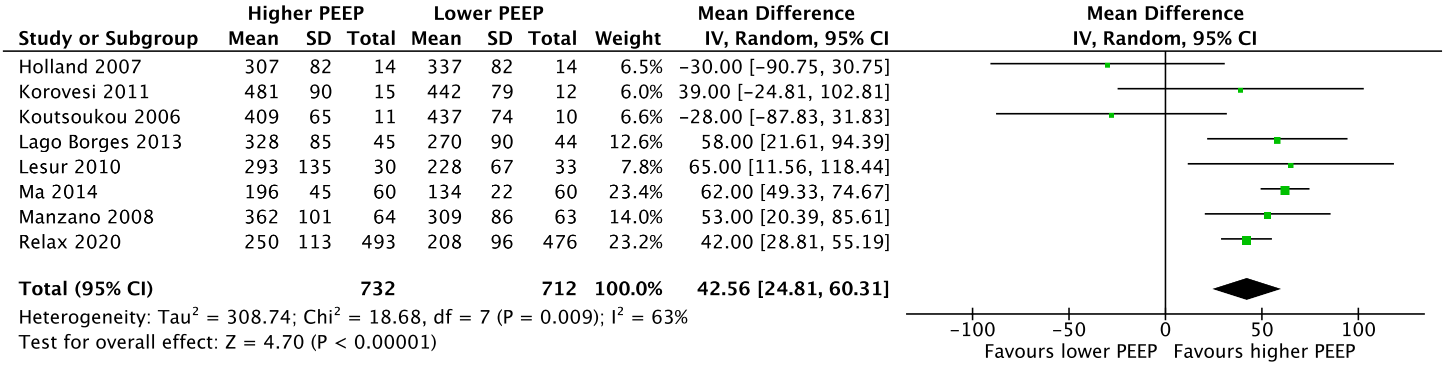

Sensitivity analysis (timing of measurement)

PaO2/FiO2 (korovesi_d5-manzano_basal-relax_afterrand)

	Higher PEEP			Lower PEEP				Mean Difference
Study or Subgroup	Mean	SD	Total	Mean	SD	Total	Weight	IV, Random, 95% CI
Holland 2007	307	82	14	337	82	14	8.9%	−30.00 [−90.75, 30.75]
Korovesi 2011	441	97	15	407	71	12	8.5%	34.00 [−29.43, 97.43]
Koutsoukou 2006	409	65	11	437	74	10	9.1%	−28.00 [−87.83, 31.83]
Lago Borges 2013	328	85	45	270	90	44	13.5%	58.00 [21.61, 94.39]
Lesur 2010	293	135	30	228	67	33	10.1%	65.00 [11.56, 118.44]
Ma 2014	196	45	60	134	22	60	18.1%	62.00 [49.33, 74.67]
Manzano 2008	392	104	64	375	79	63	14.4%	17.00 [−15.09, 49.09]
Relax 2020	230	140	493	216	124	476	17.5%	14.00 [−2.64, 30.64]
**Total (95% CI)**			**732**			**712**	**100.0%**	**28.18 [3.27, 53.08]**

Heterogeneity: $Tau^2 = 852.22$; $Chi^2 = 34.80$, df = 7 (P < 0.0001); $I^2 = 80\%$
Test for overall effect: Z = 2.22 (P = 0.03)

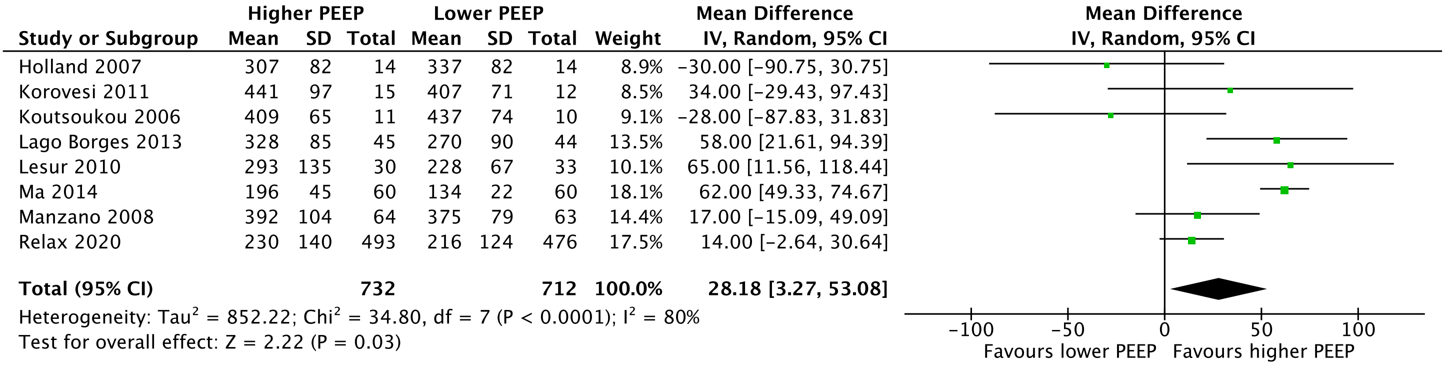

Sensitivity analysis (timing of measurement)

PaO2/FiO2 (korovesi_d5-manzano_basal-relax_d1)

Study or Subgroup	Higher PEEP Mean	Higher PEEP SD	Higher PEEP Total	Lower PEEP Mean	Lower PEEP SD	Lower PEEP Total	Weight	Mean Difference IV, Random, 95% CI
Holland 2007	307	82	14	337	82	14	7.1%	−30.00 [−90.75, 30.75]
Korovesi 2011	441	97	15	407	71	12	6.7%	34.00 [−29.43, 97.43]
Koutsoukou 2006	409	65	11	437	74	10	7.3%	−28.00 [−87.83, 31.83]
Lago Borges 2013	328	85	45	270	90	44	13.0%	58.00 [21.61, 94.39]
Lesur 2010	293	135	30	228	67	33	8.5%	65.00 [11.56, 118.44]
Ma 2014	196	45	60	134	22	60	21.7%	62.00 [49.33, 74.67]
Manzano 2008	392	104	64	375	79	63	14.5%	17.00 [−15.09, 49.09]
Relax 2020	272	126	493	226	101	476	21.2%	46.00 [31.65, 60.35]
**Total (95% CI)**			**732**			**712**	**100.0%**	**36.82 [17.43, 56.20]**

Heterogeneity: $Tau^2 = 408.54$; $Chi^2 = 21.68$, df = 7 (P = 0.003); $I^2 = 68\%$
Test for overall effect: Z = 3.72 (P = 0.0002)

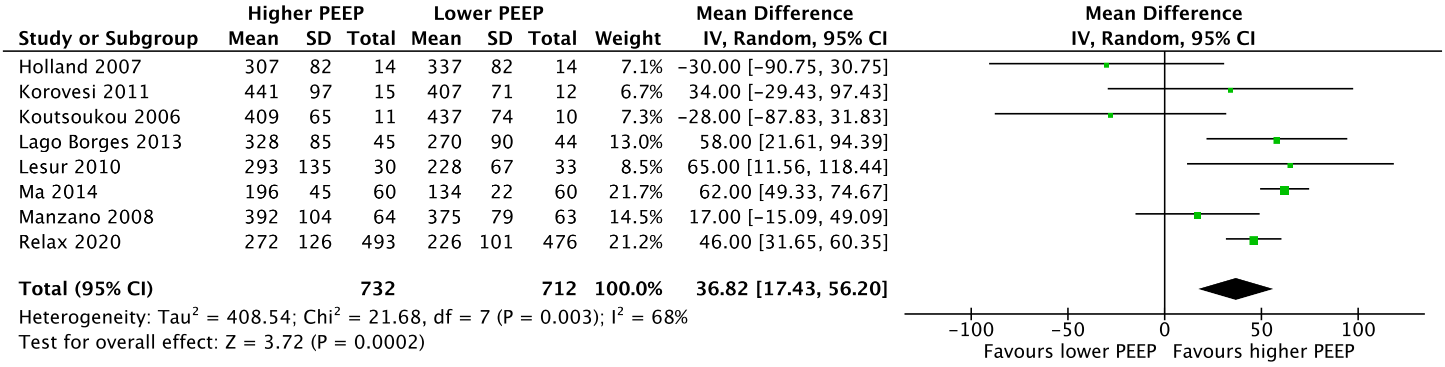

Sensitivity analysis (timing of measurement)

PaO2/FiO2 (korovesi_d5-manzano_basal-relax_d2)

	Higher PEEP			Lower PEEP				Mean Difference
Study or Subgroup	Mean	SD	Total	Mean	SD	Total	Weight	IV, Random, 95% CI
Holland 2007	307	82	14	337	82	14	7.1%	-30.00 [-90.75, 30.75]
Korovesi 2011	441	97	15	407	71	12	6.7%	34.00 [-29.43, 97.43]
Koutsoukou 2006	409	65	11	437	74	10	7.3%	-28.00 [-87.83, 31.83]
Lago Borges 2013	328	85	45	270	90	44	12.9%	58.00 [21.61, 94.39]
Lesur 2010	293	135	30	228	67	33	8.4%	65.00 [11.56, 118.44]
Ma 2014	196	45	60	134	22	60	21.7%	62.00 [49.33, 74.67]
Manzano 2008	392	104	64	375	79	63	14.4%	17.00 [-15.09, 49.09]
Relax 2020	250	113	493	208	96	476	21.5%	42.00 [28.81, 55.19]
**Total (95% CI)**			**732**			**712**	**100.0%**	**36.01 [16.70, 55.33]**

Heterogeneity: $Tau^2 = 406.40$; $Chi^2 = 22.44$, df = 7 (P = 0.002); $I^2 = 69\%$
Test for overall effect: Z = 3.65 (P = 0.0003)

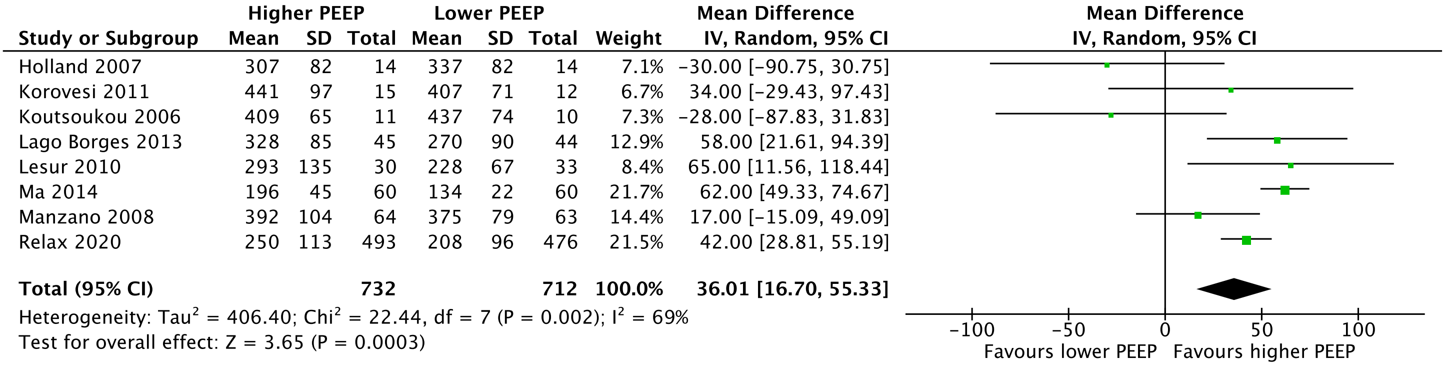

Sensitivity analysis (timing of measurement)

PaO2/FiO2 (korovesi_d5-manzano_6h-relax_afterrand)

	Higher PEEP			Lower PEEP				Mean Difference
**Study or Subgroup**	**Mean**	**SD**	**Total**	**Mean**	**SD**	**Total**	**Weight**	**IV, Random, 95% CI**
Holland 2007	307	82	14	337	82	14	8.9%	−30.00 [−90.75, 30.75]
Korovesi 2011	441	97	15	407	71	12	8.5%	34.00 [−29.43, 97.43]
Koutsoukou 2006	409	65	11	437	74	10	9.1%	−28.00 [−87.83, 31.83]
Lago Borges 2013	328	85	45	270	90	44	13.6%	58.00 [21.61, 94.39]
Lesur 2010	293	135	30	228	67	33	10.2%	65.00 [11.56, 118.44]
Ma 2014	196	45	60	134	22	60	18.3%	62.00 [49.33, 74.67]
Manzano 2008	356	100	64	332	108	63	13.6%	24.00 [−12.21, 60.21]
Relax 2020	230	140	493	216	124	476	17.7%	14.00 [−2.64, 30.64]
**Total (95% CI)**			**732**			**712**	**100.0%**	**29.28 [4.41, 54.14]**

Heterogeneity: $Tau^2 = 837.74$; $Chi^2 = 33.53$, df = 7 ($P < 0.0001$); $I^2 = 79\%$
Test for overall effect: $Z = 2.31$ ($P = 0.02$)

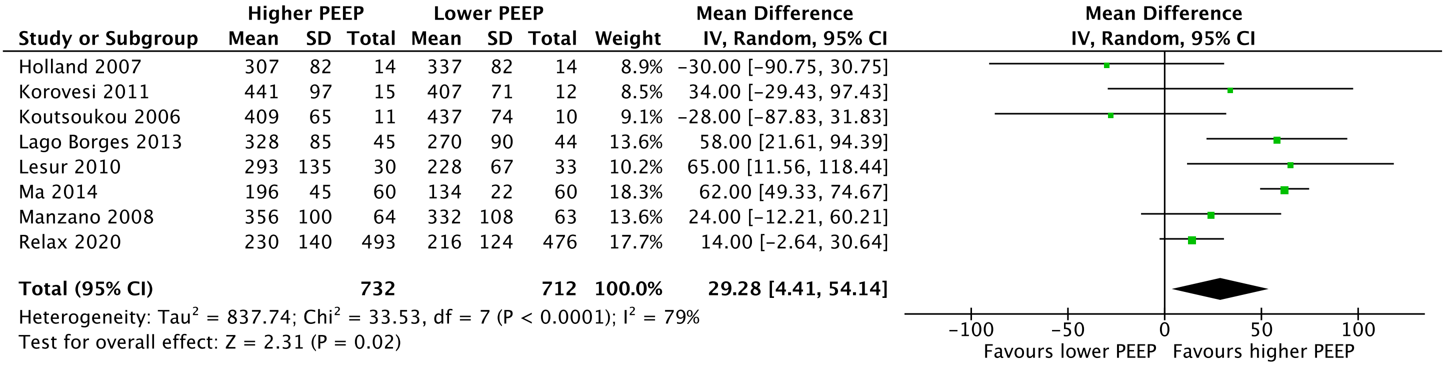

Sensitivity analysis (timing of measurement)

PaO2/FiO2 (korovesi_d5-manzano_6h-relax_d1)

	Higher PEEP			Lower PEEP				Mean Difference
**Study or Subgroup**	**Mean**	**SD**	**Total**	**Mean**	**SD**	**Total**	**Weight**	**IV, Random, 95% CI**
Holland 2007	307	82	14	337	82	14	7.0%	−30.00 [−90.75, 30.75]
Korovesi 2011	441	97	15	407	71	12	6.5%	34.00 [−29.43, 97.43]
Koutsoukou 2006	409	65	11	437	74	10	7.1%	−28.00 [−87.83, 31.83]
Lago Borges 2013	328	85	45	270	90	44	13.0%	58.00 [21.61, 94.39]
Lesur 2010	293	135	30	228	67	33	8.3%	65.00 [11.56, 118.44]
Ma 2014	196	45	60	134	22	60	22.8%	62.00 [49.33, 74.67]
Manzano 2008	356	100	64	332	108	63	13.1%	24.00 [−12.21, 60.21]
Relax 2020	272	126	493	226	101	476	22.2%	46.00 [31.65, 60.35]
**Total (95% CI)**			**732**			**712**	**100.0%**	**38.58 [19.78, 57.38]**

Heterogeneity: $Tau^2 = 361.62$; $Chi^2 = 19.65$, df = 7 (P = 0.006); $I^2 = 64\%$
Test for overall effect: Z = 4.02 (P < 0.0001)

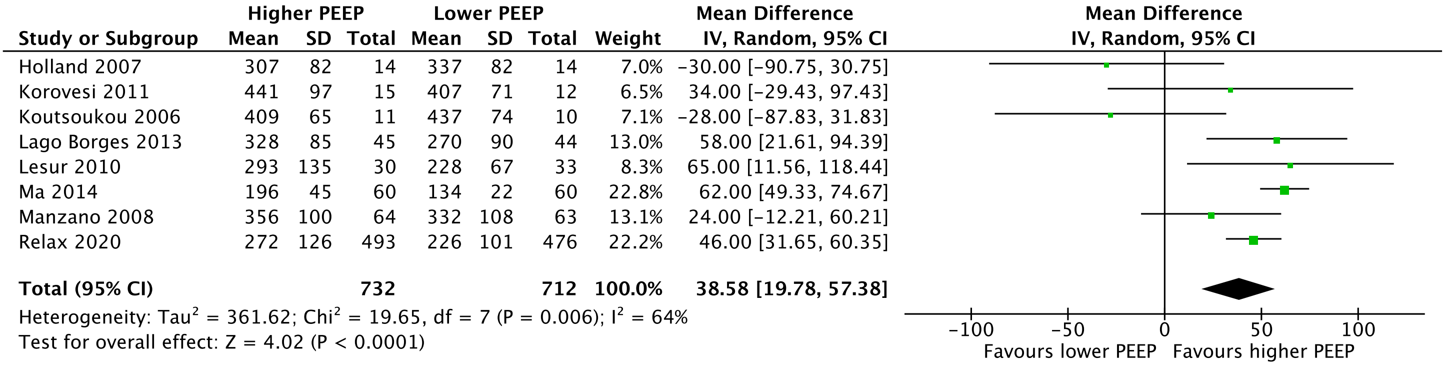

Sensitivity analysis (timing of measurement)

PaO2/FiO2 (korovesi_d5-manzano_6h-relax_d2)

	Higher PEEP			Lower PEEP				Mean Difference
**Study or Subgroup**	**Mean**	**SD**	**Total**	**Mean**	**SD**	**Total**	**Weight**	**IV, Random, 95% CI**
Holland 2007	307	82	14	337	82	14	7.0%	−30.00 [−90.75, 30.75]
Korovesi 2011	441	97	15	407	71	12	6.5%	34.00 [−29.43, 97.43]
Koutsoukou 2006	409	65	11	437	74	10	7.1%	−28.00 [−87.83, 31.83]
Lago Borges 2013	328	85	45	270	90	44	13.0%	58.00 [21.61, 94.39]
Lesur 2010	293	135	30	228	67	33	8.3%	65.00 [11.56, 118.44]
Ma 2014	196	45	60	134	22	60	22.6%	62.00 [49.33, 74.67]
Manzano 2008	356	100	64	332	108	63	13.0%	24.00 [−12.21, 60.21]
Relax 2020	250	113	493	208	96	476	22.4%	42.00 [28.81, 55.19]
**Total (95% CI)**			**732**			**712**	**100.0%**	**37.65 [18.82, 56.49]**

Heterogeneity: $Tau^2 = 366.52$; $Chi^2 = 20.57$, df = 7 (P = 0.004); $I^2 = 66\%$
Test for overall effect: Z = 3.92 (P < 0.0001)

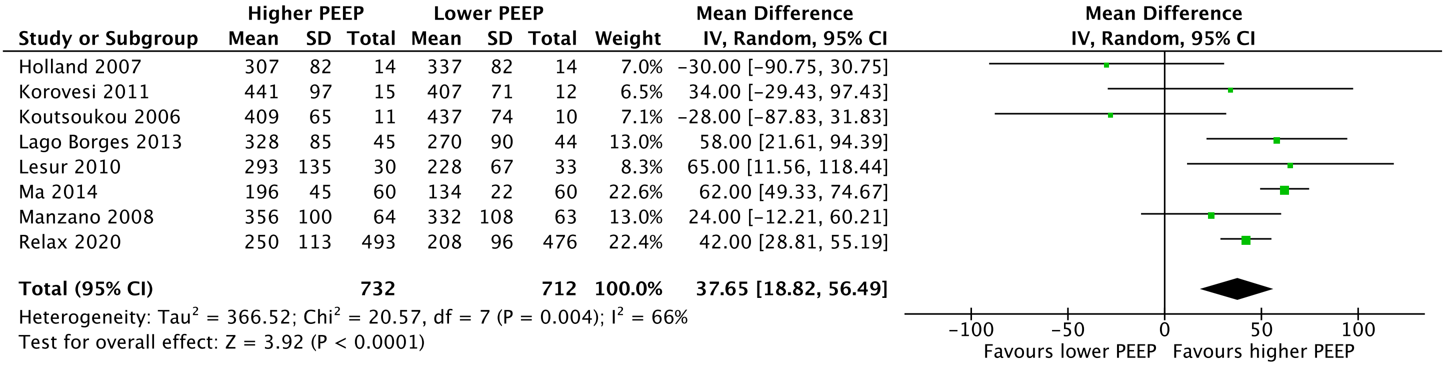

Sensitivity analysis (timing of measurement)

PaO2/FiO2 (korovesi_d5-manzano_d1-relax_afterrand)

	Higher PEEP			Lower PEEP				Mean Difference
**Study or Subgroup**	**Mean**	**SD**	**Total**	**Mean**	**SD**	**Total**	**Weight**	**IV, Random, 95% CI**
Holland 2007	307	82	14	337	82	14	8.8%	−30.00 [−90.75, 30.75]
Korovesi 2011	441	97	15	407	71	12	8.4%	34.00 [−29.43, 97.43]
Koutsoukou 2006	409	65	11	437	74	10	8.9%	−28.00 [−87.83, 31.83]
Lago Borges 2013	328	85	45	270	90	44	13.5%	58.00 [21.61, 94.39]
Lesur 2010	293	135	30	228	67	33	10.0%	65.00 [11.56, 118.44]
Ma 2014	196	45	60	134	22	60	18.4%	62.00 [49.33, 74.67]
Manzano 2008	362	101	64	309	86	63	14.3%	53.00 [20.39, 85.61]
Relax 2020	230	140	493	216	124	476	17.7%	14.00 [−2.64, 30.64]
**Total (95% CI)**			**732**			**712**	**100.0%**	**33.51 [9.15, 57.88]**

Heterogeneity: $Tau^2 = 800.47$; $Chi^2 = 33.00$, df = 7 (P < 0.0001); $I^2 = 79\%$
Test for overall effect: Z = 2.70 (P = 0.007)

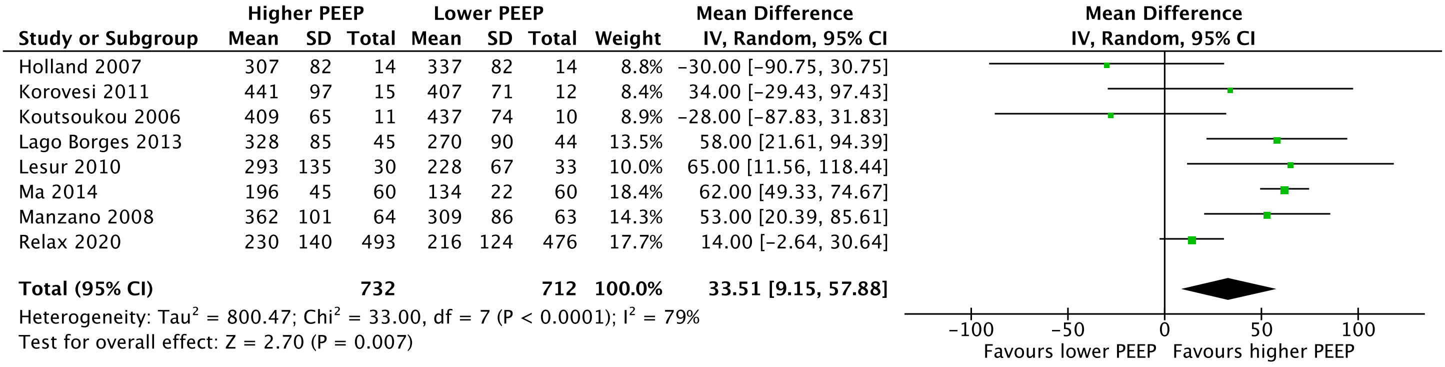

Sensitivity analysis (timing of measurement)

PaO2/FiO2 (korovesi_d5-manzano_d1-relax_d1)

	Higher PEEP			Lower PEEP				Mean Difference
Study or Subgroup	Mean	SD	Total	Mean	SD	Total	Weight	IV, Random, 95% CI
Holland 2007	307	82	14	337	82	14	6.4%	-30.00 [-90.75, 30.75]
Korovesi 2011	441	97	15	407	71	12	6.0%	34.00 [-29.43, 97.43]
Koutsoukou 2006	409	65	11	437	74	10	6.5%	-28.00 [-87.83, 31.83]
Lago Borges 2013	328	85	45	270	90	44	12.5%	58.00 [21.61, 94.39]
Lesur 2010	293	135	30	228	67	33	7.7%	65.00 [11.56, 118.44]
Ma 2014	196	45	60	134	22	60	23.8%	62.00 [49.33, 74.67]
Manzano 2008	362	101	64	309	86	63	14.0%	53.00 [20.39, 85.61]
Relax 2020	272	126	493	226	101	476	23.0%	46.00 [31.65, 60.35]
**Total (95% CI)**			**732**			**712**	**100.0%**	**43.35 [25.80, 60.91]**

Heterogeneity: $Tau^2 = 295.15$; $Chi^2 = 17.57$, df = 7 (P = 0.01); $I^2 = 60\%$
Test for overall effect: Z = 4.84 (P < 0.00001)

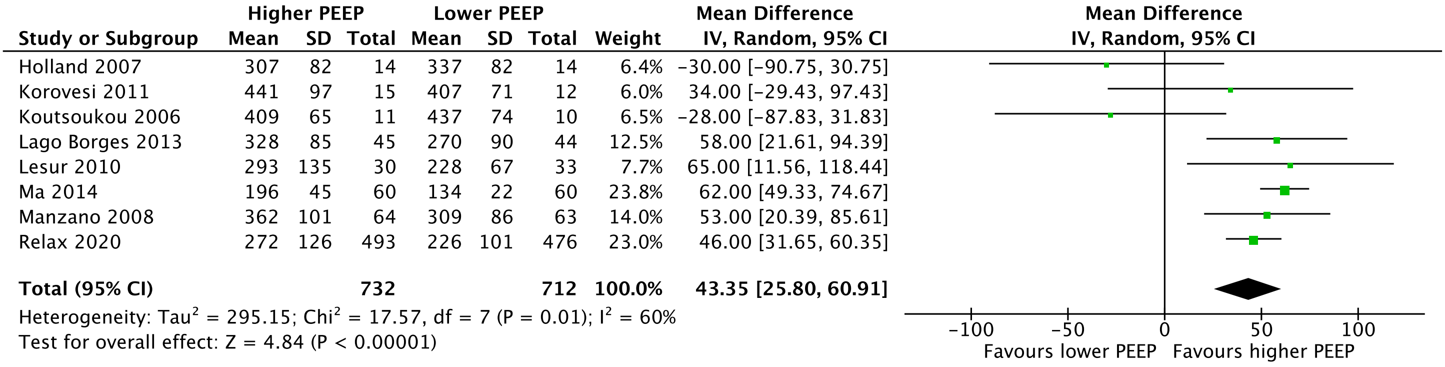

Sensitivity analysis (timing of measurement)

PaO2/FiO2 (korovesi_d5-manzano_d1-relax_d2)

Study or Subgroup	Higher PEEP Mean	Higher PEEP SD	Higher PEEP Total	Lower PEEP Mean	Lower PEEP SD	Lower PEEP Total	Weight	Mean Difference IV, Random, 95% CI
Holland 2007	307	82	14	337	82	14	6.5%	−30.00 [−90.75, 30.75]
Korovesi 2011	441	97	15	407	71	12	6.1%	34.00 [−29.43, 97.43]
Koutsoukou 2006	409	65	11	437	74	10	6.6%	−28.00 [−87.83, 31.83]
Lago Borges 2013	328	85	45	270	90	44	12.6%	58.00 [21.61, 94.39]
Lesur 2010	293	135	30	228	67	33	7.8%	65.00 [11.56, 118.44]
Ma 2014	196	45	60	134	22	60	23.3%	62.00 [49.33, 74.67]
Manzano 2008	362	101	64	309	86	63	14.0%	53.00 [20.39, 85.61]
Relax 2020	250	113	493	208	96	476	23.1%	42.00 [28.81, 55.19]
**Total (95% CI)**			**732**			**712**	**100.0%**	**42.22 [24.41, 60.02]**

Heterogeneity: $Tau^2 = 311.99$; $Chi^2 = 18.81$, df = 7 (P = 0.009); $I^2 = 63\%$
Test for overall effect: Z = 4.65 (P < 0.00001)

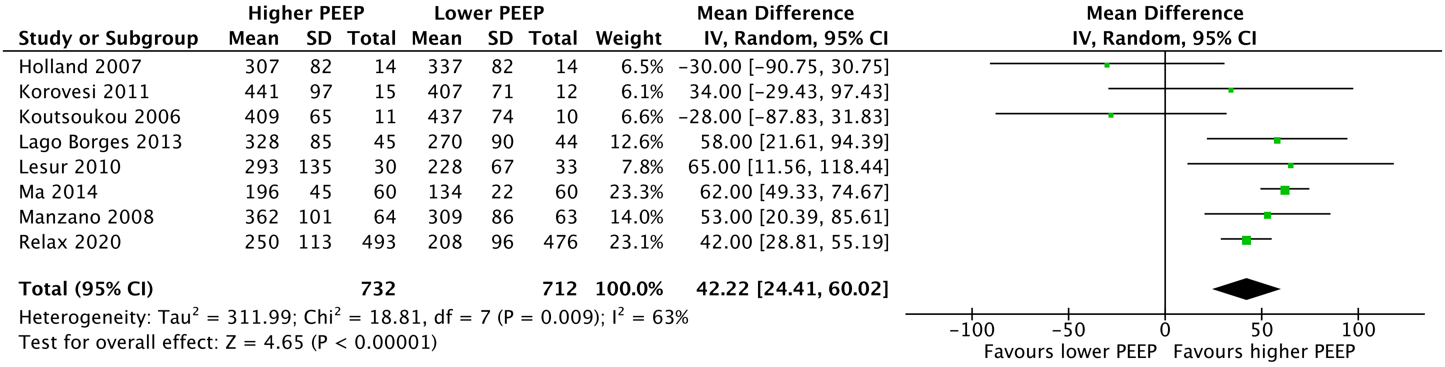

Sensitivity analysis (timing of measurement)

PaO2/FiO2 (korovesi_d1)

	Higher PEEP			Lower PEEP				Mean Difference	Mean Difference
**Study or Subgroup**	**Mean**	**SD**	**Total**	**Mean**	**SD**	**Total**	**Weight**	**IV, Random, 95% CI**	**IV, Random, 95% CI**
Holland 2007	307	82	14	337	82	14	5.9%	−30.00 [−90.75, 30.75]	
Korovesi 2011	481	90	15	442	79	12	5.5%	39.00 [−24.81, 102.81]	
Koutsoukou 2006	409	65	11	437	74	10	6.1%	−28.00 [−87.83, 31.83]	
Lago Borges 2013	328	85	45	270	90	44	12.1%	58.00 [21.61, 94.39]	
Lesur 2010	293	135	30	228	67	33	7.2%	65.00 [11.56, 118.44]	
Ma 2014	196	45	60	134	22	60	24.8%	62.00 [49.33, 74.67]	
Manzano 2008	359	104	64	301	84	63	13.5%	58.00 [25.15, 90.85]	
Relax 2020	248	112	493	190	84	476	24.9%	58.00 [45.56, 70.44]	
**Total (95% CI)**			**732**			**712**	**100.0%**	**48.05 [31.50, 64.60]**	

Heterogeneity: $Tau^2 = 245.50$; $Chi^2 = 16.63$, df = 7 (P = 0.02); $I^2 = 58\%$
Test for overall effect: Z = 5.69 (P < 0.00001)

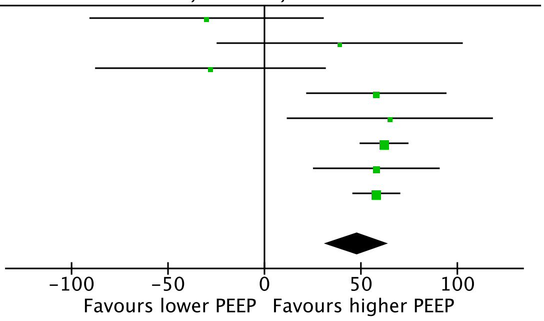

Sensitivity analysis (timing of measurement)

PaO2/FiO2 (korovesi_d5)

	Higher PEEP			Lower PEEP				Mean Difference
Study or Subgroup	Mean	SD	Total	Mean	SD	Total	Weight	IV, Random, 95% CI
Holland 2007	307	82	14	337	82	14	6.0%	-30.00 [-90.75, 30.75]
Korovesi 2011	441	97	15	407	71	12	5.6%	34.00 [-29.43, 97.43]
Koutsoukou 2006	409	65	11	437	74	10	6.1%	-28.00 [-87.83, 31.83]
Lago Borges 2013	328	85	45	270	90	44	12.1%	58.00 [21.61, 94.39]
Lesur 2010	293	135	30	228	67	33	7.3%	65.00 [11.56, 118.44]
Ma 2014	196	45	60	134	22	60	24.7%	62.00 [49.33, 74.67]
Manzano 2008	359	104	64	301	84	63	13.6%	58.00 [25.15, 90.85]
Relax 2020	248	112	493	190	84	476	24.8%	58.00 [45.56, 70.44]
**Total (95% CI)**			**732**			**712**	**100.0%**	**47.68 [31.04, 64.32]**

Heterogeneity: $Tau^2 = 250.32$; $Chi^2 = 16.82$, df = 7 (P = 0.02); $I^2 = 58\%$
Test for overall effect: Z = 5.62 (P < 0.00001)

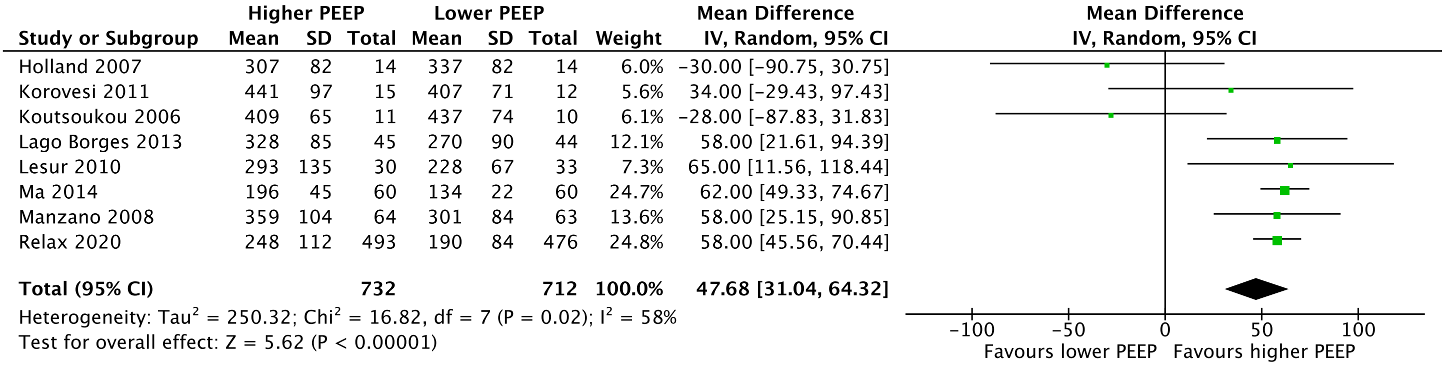

Sensitivity analysis (timing of measurement)

PaO2/FiO2 (manzano_basal)

	Higher PEEP			Lower PEEP				Mean Difference
**Study or Subgroup**	**Mean**	**SD**	**Total**	**Mean**	**SD**	**Total**	**Weight**	**IV, Random, 95% CI**
Holland 2007	307	82	14	337	82	14	6.9%	−30.00 [−90.75, 30.75]
Korovesi 2011	498	75	15	420	73	12	7.7%	78.00 [21.91, 134.09]
Koutsoukou 2006	409	65	11	437	74	10	7.0%	−28.00 [−87.83, 31.83]
Lago Borges 2013	328	85	45	270	90	44	12.7%	58.00 [21.61, 94.39]
Lesur 2010	293	135	30	228	67	33	8.2%	65.00 [11.56, 118.44]
Ma 2014	196	45	60	134	22	60	21.6%	62.00 [49.33, 74.67]
Manzano 2008	392	104	64	375	79	63	14.2%	17.00 [−15.09, 49.09]
Relax 2020	248	112	493	190	84	476	21.7%	58.00 [45.56, 70.44]
**Total (95% CI)**			**732**			**712**	**100.0%**	**43.04 [24.13, 61.95]**

Heterogeneity: $Tau^2 = 389.19$; $Chi^2 = 22.49$, df = 7 (P = 0.002); $I^2 = 69\%$
Test for overall effect: Z = 4.46 (P < 0.00001)

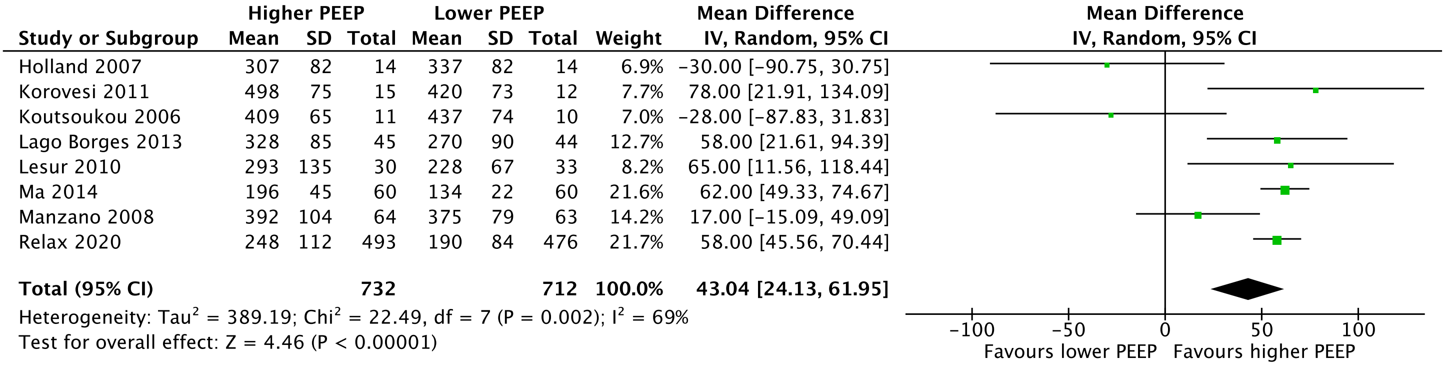

Sensitivity analysis (timing of measurement)

PaO2/FiO2 (manzano_6h)

	Higher PEEP			Lower PEEP				Mean Difference
**Study or Subgroup**	**Mean**	**SD**	**Total**	**Mean**	**SD**	**Total**	**Weight**	**IV, Random, 95% CI**
Holland 2007	307	82	14	337	82	14	6.6%	-30.00 [-90.75, 30.75]
Korovesi 2011	498	75	15	420	73	12	7.4%	78.00 [21.91, 134.09]
Koutsoukou 2006	409	65	11	437	74	10	6.8%	-28.00 [-87.83, 31.83]
Lago Borges 2013	328	85	45	270	90	44	12.6%	58.00 [21.61, 94.39]
Lesur 2010	293	135	30	228	67	33	8.0%	65.00 [11.56, 118.44]
Ma 2014	196	45	60	134	22	60	22.9%	62.00 [49.33, 74.67]
Manzano 2008	356	100	64	332	108	63	12.7%	24.00 [-12.21, 60.21]
Relax 2020	248	112	493	190	84	476	23.0%	58.00 [45.56, 70.44]
**Total (95% CI)**			**732**			**712**	**100.0%**	**44.98 [26.84, 63.12]**

Heterogeneity: Tau^2 = 332.89; Chi^2 = 19.92, df = 7 (P = 0.006); I^2 = 65%
Test for overall effect: Z = 4.86 (P < 0.00001)

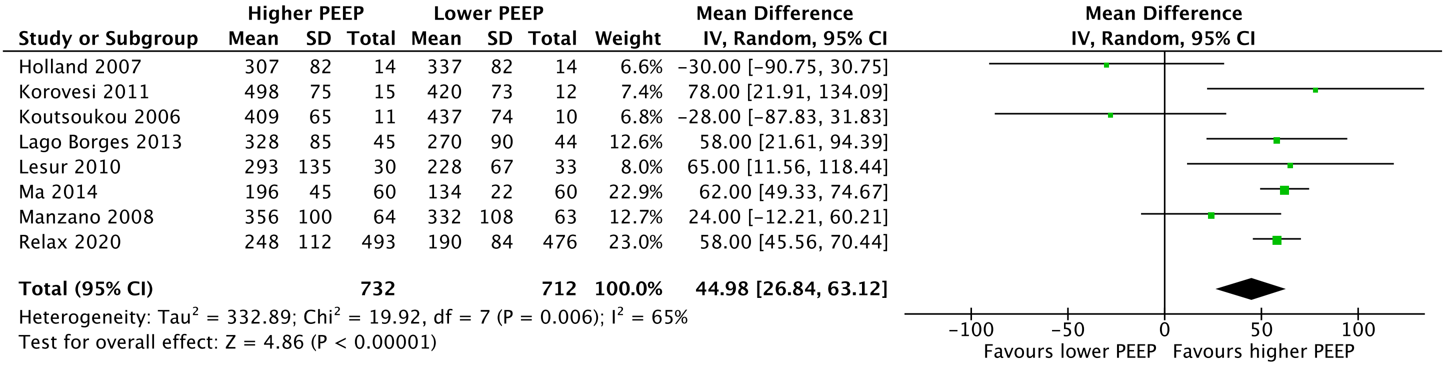

Sensitivity analysis (timing of measurement)

PaO2/FiO2 (manzano_d1)

	Higher PEEP			Lower PEEP				Mean Difference
**Study or Subgroup**	**Mean**	**SD**	**Total**	**Mean**	**SD**	**Total**	**Weight**	**IV, Random, 95% CI**
Holland 2007	307	82	14	337	82	14	5.9%	−30.00 [−90.75, 30.75]
Korovesi 2011	498	75	15	420	73	12	6.7%	78.00 [21.91, 134.09]
Koutsoukou 2006	409	65	11	437	74	10	6.0%	−28.00 [−87.83, 31.83]
Lago Borges 2013	328	85	45	270	90	44	12.0%	58.00 [21.61, 94.39]
Lesur 2010	293	135	30	228	67	33	7.2%	65.00 [11.56, 118.44]
Ma 2014	196	45	60	134	22	60	24.3%	62.00 [49.33, 74.67]
Manzano 2008	362	101	64	309	86	63	13.5%	53.00 [20.39, 85.61]
Relax 2020	248	112	493	190	84	476	24.5%	58.00 [45.56, 70.44]
**Total (95% CI)**			**732**			**712**	**100.0%**	**49.78 [33.24, 66.32]**

Heterogeneity: $Tau^2 = 250.60$; $Chi^2 = 16.94$, df = 7 (P = 0.02); $I^2 = 59\%$
Test for overall effect: Z = 5.90 (P < 0.00001)

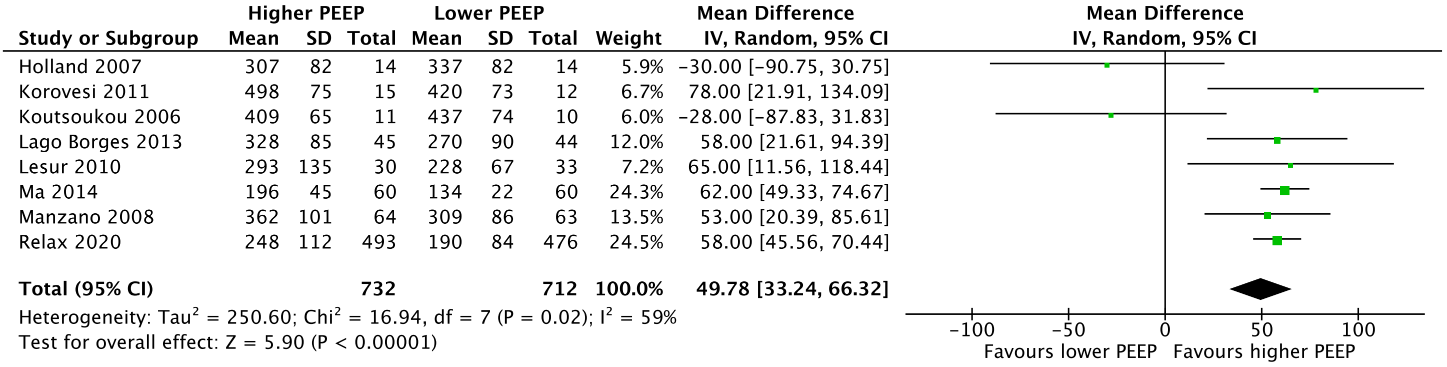

Sensitivity analysis (timing of measurement)

PaO2/FiO2 (relax_afterrand)

	Higher PEEP			Lower PEEP				Mean Difference
**Study or Subgroup**	**Mean**	**SD**	**Total**	**Mean**	**SD**	**Total**	**Weight**	**IV, Random, 95% CI**
Holland 2007	307	82	14	337	82	14	8.8%	−30.00 [−90.75, 30.75]
Korovesi 2011	498	75	15	420	73	12	9.6%	78.00 [21.91, 134.09]
Koutsoukou 2006	409	65	11	437	74	10	8.9%	−28.00 [−87.83, 31.83]
Lago Borges 2013	328	85	45	270	90	44	13.4%	58.00 [21.61, 94.39]
Lesur 2010	293	135	30	228	67	33	10.0%	65.00 [11.56, 118.44]
Ma 2014	196	45	60	134	22	60	17.9%	62.00 [49.33, 74.67]
Manzano 2008	359	104	64	301	84	63	14.1%	58.00 [25.15, 90.85]
Relax 2020	230	140	493	216	124	476	17.3%	14.00 [−2.64, 30.64]
**Total (95% CI)**			**732**			**712**	**100.0%**	**38.26 [13.53, 62.99]**

Heterogeneity: $Tau^2 = 847.41$; $Chi^2 = 34.77$, df = 7 ($P < 0.0001$); $I^2 = 80\%$
Test for overall effect: Z = 3.03 (P = 0.002)

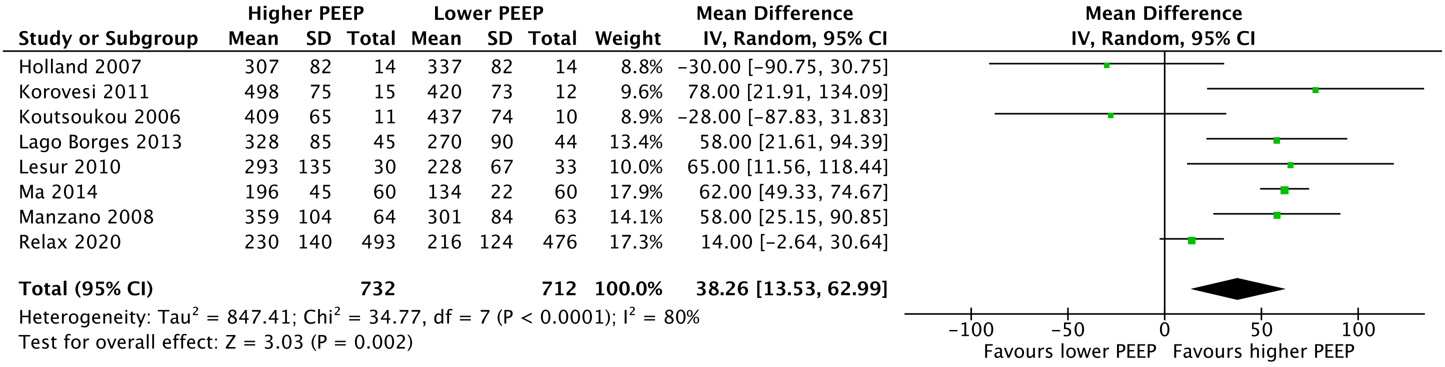

Sensitivity analysis (timing of measurement)

PaO2/FiO2 (relax_d1)

Study or Subgroup	Higher PEEP Mean	Higher PEEP SD	Higher PEEP Total	Lower PEEP Mean	Lower PEEP SD	Lower PEEP Total	Weight	Mean Difference IV, Random, 95% CI
Holland 2007	307	82	14	337	82	14	6.4%	−30.00 [−90.75, 30.75]
Korovesi 2011	498	75	15	420	73	12	7.2%	78.00 [21.91, 134.09]
Koutsoukou 2006	409	65	11	437	74	10	6.6%	−28.00 [−87.83, 31.83]
Lago Borges 2013	328	85	45	270	90	44	12.5%	58.00 [21.61, 94.39]
Lesur 2010	293	135	30	228	67	33	7.8%	65.00 [11.56, 118.44]
Ma 2014	196	45	60	134	22	60	23.2%	62.00 [49.33, 74.67]
Manzano 2008	359	104	64	301	84	63	13.8%	58.00 [25.15, 90.85]
Relax 2020	272	126	493	226	101	476	22.5%	46.00 [31.65, 60.35]
**Total (95% CI)**			**732**			**712**	**100.0%**	**46.91 [29.19, 64.62]**

Heterogeneity: $Tau^2 = 309.89$; $Chi^2 = 18.18$, df = 7 (P = 0.01); $I^2 = 61\%$

Test for overall effect: Z = 5.19 (P < 0.00001)

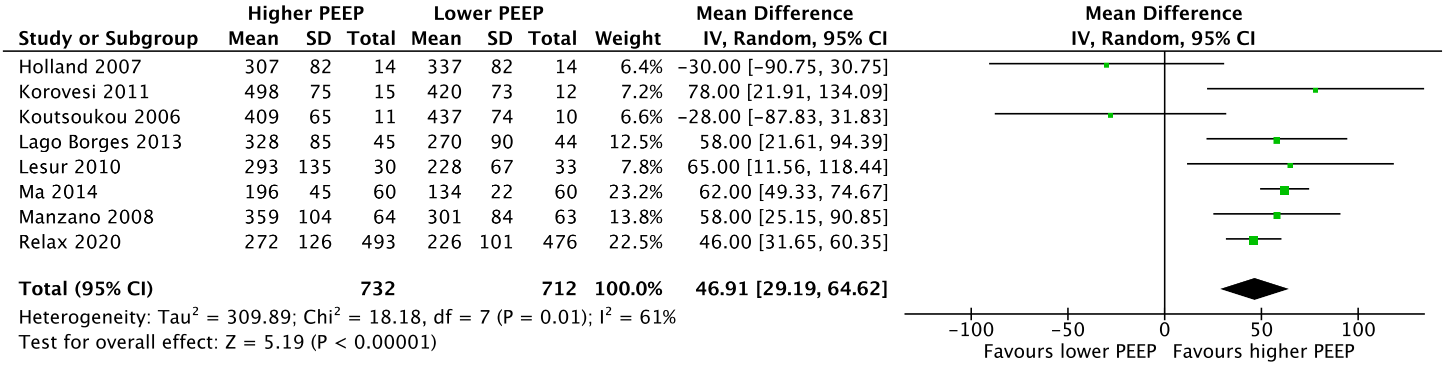

Sensitivity analysis (timing of measurement)

PaO2/FiO2 (relax_d2)

	Higher PEEP			Lower PEEP				Mean Difference
**Study or Subgroup**	**Mean**	**SD**	**Total**	**Mean**	**SD**	**Total**	**Weight**	**IV, Random, 95% CI**
Holland 2007	307	82	14	337	82	14	6.6%	-30.00 [-90.75, 30.75]
Korovesi 2011	498	75	15	420	73	12	7.4%	78.00 [21.91, 134.09]
Koutsoukou 2006	409	65	11	437	74	10	6.7%	-28.00 [-87.83, 31.83]
Lago Borges 2013	328	85	45	270	90	44	12.5%	58.00 [21.61, 94.39]
Lesur 2010	293	135	30	228	67	33	7.9%	65.00 [11.56, 118.44]
Ma 2014	196	45	60	134	22	60	22.7%	62.00 [49.33, 74.67]
Manzano 2008	359	104	64	301	84	63	13.8%	58.00 [25.15, 90.85]
Relax 2020	250	113	493	208	96	476	22.5%	42.00 [28.81, 55.19]
**Total (95% CI)**			**732**			**712**	**100.0%**	**45.79 [27.74, 63.85]**

Heterogeneity: $Tau^2 = 332.54$; $Chi^2 = 19.68$, df = 7 (P = 0.006); $I^2 = 64\%$
Test for overall effect: Z = 4.97 (P < 0.00001)

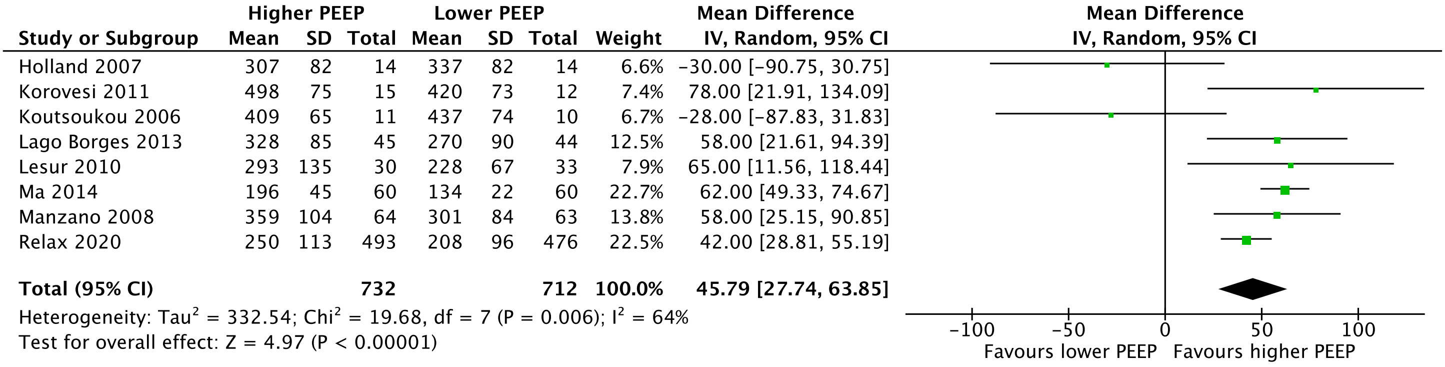

Sensitivity analysis (odds ratio)

Hospital mortality

Study or Subgroup	Higher PEEP Events	Higher PEEP Total	Lower PEEP Events	Lower PEEP Total	Weight	Odds Ratio M-H, Random, 95% CI
Collier 2002	1	40	1	44	0.6%	1.10 [0.07, 18.23]
Dyhr 2002	0	7	0	8		Not estimable
Lesur 2010	12	30	16	33	4.8%	0.71 [0.26, 1.93]
Manzano 2008	19	64	16	63	7.9%	1.24 [0.57, 2.71]
Michalopoulos 1998	0	21	0	22		Not estimable
Nelson 1987	5	20	6	18	2.4%	0.67 [0.16, 2.73]
Pepe 1984	13	44	18	48	6.3%	0.70 [0.29, 1.67]
Relax 2020	208	489	185	472	72.2%	1.15 [0.89, 1.49]
Weigelt 1979	16	45	17	34	5.8%	0.55 [0.22, 1.37]
**Total (95% CI)**		**760**		**742**	**100.0%**	**1.03 [0.83, 1.29]**
Total events	274		259			

Heterogeneity: $Tau^2 = 0.00$; $Chi^2 = 4.38$, df = 6 (P = 0.62); $I^2 = 0\%$
Test for overall effect: Z = 0.30 (P = 0.76)

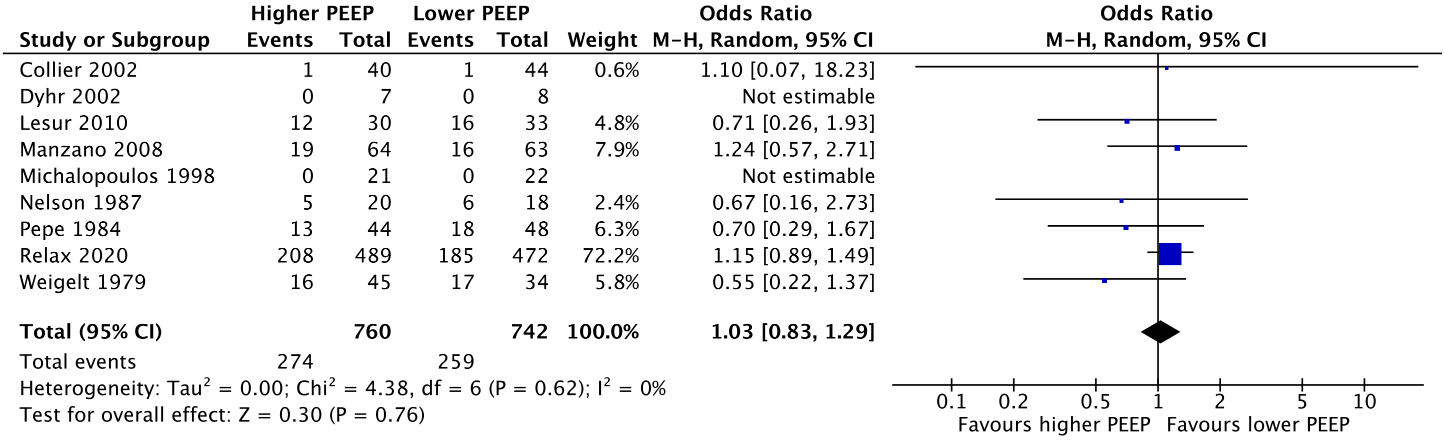

Sensitivity analysis (odds ratio)

Hypoxemia

	Higher PEEP		Lower PEEP			Odds Ratio
Study or Subgroup	Events	Total	Events	Total	Weight	M-H, Random, 95% CI
Lago Borges 2013	19	45	30	44	23.9%	0.34 [0.14, 0.81]
Manzano 2008	12	64	34	63	25.0%	0.20 [0.09, 0.44]
Michalopoulos 1998	2	21	2	22	10.0%	1.05 [0.13, 8.24]
Pepe 1984	1	44	4	48	8.9%	0.26 [0.03, 2.38]
Relax 2020	87	493	98	476	32.2%	0.83 [0.60, 1.14]
**Total (95% CI)**		**667**		**653**	**100.0%**	**0.43 [0.20, 0.94]**
Total events	121		168			

Heterogeneity: $Tau^2 = 0.46$; $Chi^2 = 13.68$, df = 4 (P = 0.008); $I^2 = 71\%$
Test for overall effect: Z = 2.13 (P = 0.03)

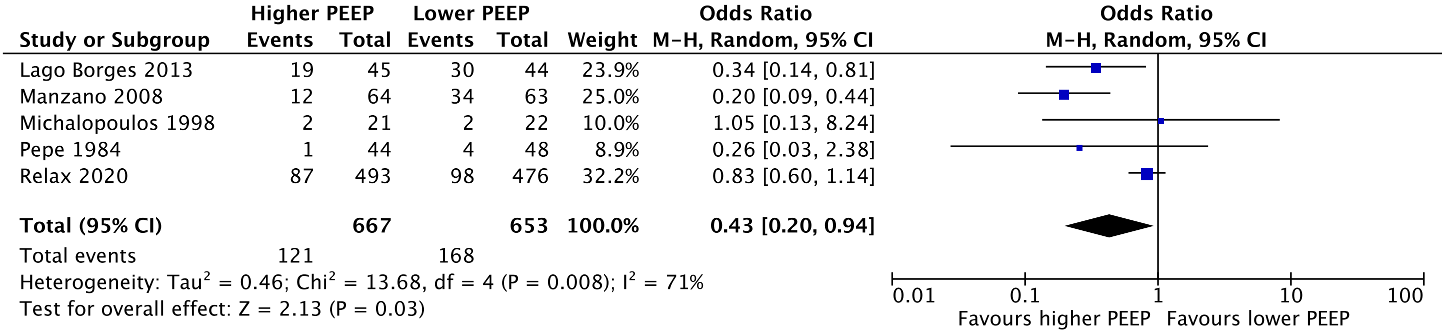

Sensitivity analysis (odds ratio)

Atelectasis

Study or Subgroup	Higher PEEP Events	Higher PEEP Total	Lower PEEP Events	Lower PEEP Total	Weight	Odds Ratio M-H, Random, 95% CI
Good 1979	9	10	12	14	2.8%	1.50 [0.12, 19.24]
Manzano 2008	12	64	17	63	26.3%	0.62 [0.27, 1.44]
Michalopoulos 1998	2	21	2	22	4.4%	1.05 [0.13, 8.24]
Pepe 1984	27	44	23	48	26.8%	1.73 [0.75, 3.96]
Relax 2020	15	493	20	476	39.7%	0.72 [0.36, 1.41]
**Total (95% CI)**		**632**		**623**	**100.0%**	**0.91 [0.59, 1.40]**
Total events	65		74			

Heterogeneity: $Tau^2 = 0.00$; $Chi^2 = 3.71$, df = 4 (P = 0.45); $I^2 = 0\%$

Test for overall effect: Z = 0.44 (P = 0.66)

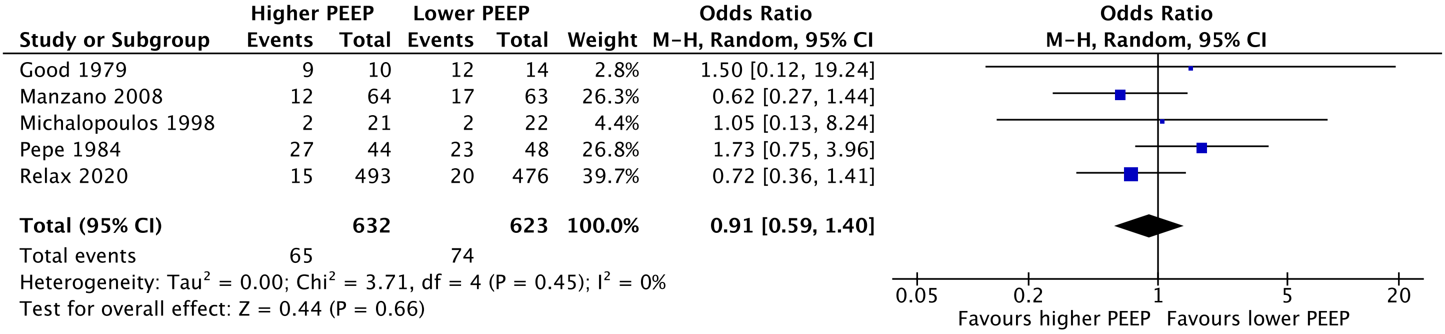

Sensitivity analysis (odds ratio)

Barotrauma

	Higher PEEP		Lower PEEP			Odds Ratio	Odds Ratio
**Study or Subgroup**	**Events**	**Total**	**Events**	**Total**	**Weight**	**M-H, Random, 95% CI**	**M-H, Random, 95% CI**
Good 1979	0	10	0	14		Not estimable	
Manzano 2008	1	64	5	63	5.1%	0.18 [0.02, 1.62]	
Michalopoulos 1998	0	21	0	22		Not estimable	
Nelson 1987	1	20	0	18	2.3%	2.85 [0.11, 74.38]	
Pepe 1984	19	44	24	48	35.6%	0.76 [0.33, 1.73]	
Relax 2020	12	493	19	476	44.7%	0.60 [0.29, 1.25]	
Weigelt 1979	5	45	4	34	12.3%	0.94 [0.23, 3.79]	
**Total (95% CI)**		**697**		**675**	**100.0%**	**0.67 [0.41, 1.10]**	
Total events	38		52				

Heterogeneity: $Tau^2 = 0.00$; $Chi^2 = 2.51$, df = 4 (P = 0.64); $I^2 = 0\%$

Test for overall effect: Z = 1.58 (P = 0.11)

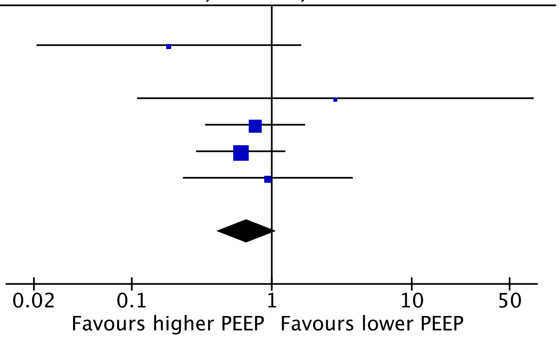

Sensitivity analysis (odds ratio)

Ventilator-associated pneumonia

	Higher PEEP		Lower PEEP			Odds Ratio
Study or Subgroup	Events	Total	Events	Total	Weight	M-H, Random, 95% CI
Manzano 2008	6	64	16	63	38.5%	0.30 [0.11, 0.84]
Pepe 1984	4	44	6	48	26.6%	0.70 [0.18, 2.67]
Relax 2020	7	493	6	476	34.9%	1.13 [0.38, 3.38]
**Total (95% CI)**		**601**		**587**	**100.0%**	**0.60 [0.27, 1.35]**
Total events	17		28			

Heterogeneity: $Tau^2 = 0.18$; $Chi^2 = 3.05$, df = 2 (P = 0.22); $I^2 = 34\%$
Test for overall effect: Z = 1.23 (P = 0.22)

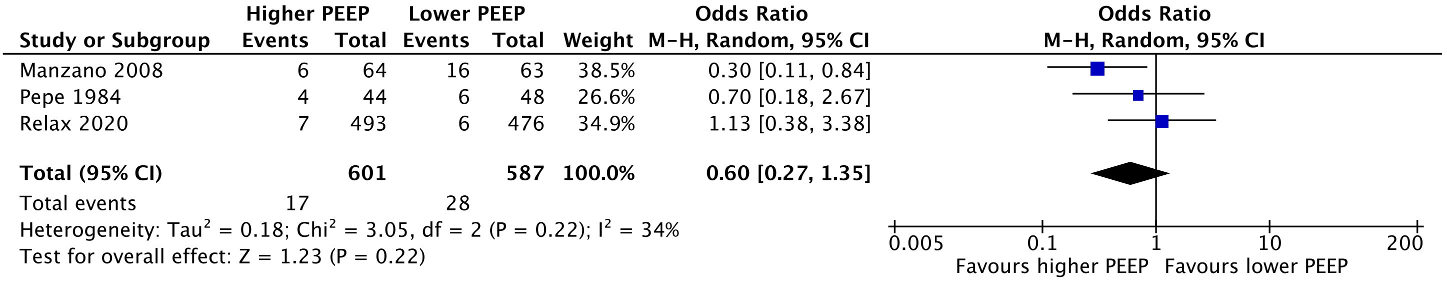

Sensitivity analysis (odds ratio)

ARDS

Study or Subgroup	Higher PEEP Events	Higher PEEP Total	Lower PEEP Events	Lower PEEP Total	Weight	Odds Ratio M-H, Random, 95% CI
Korovesi 2016	0	15	0	12		Not estimable
Koutsoukou 2006	0	11	1	10	2.8%	0.28 [0.01, 7.57]
Manzano 2008	3	64	9	63	15.5%	0.30 [0.08, 1.15]
Pepe 1984	11	44	13	48	29.9%	0.90 [0.35, 2.28]
Relax 2020	5	493	13	476	24.9%	0.36 [0.13, 1.03]
Weigelt 1979	9	45	18	34	26.9%	0.22 [0.08, 0.60]
**Total (95% CI)**		**672**		**643**	**100.0%**	**0.40 [0.23, 0.70]**
Total events	28		54			

Heterogeneity: $Tau^2 = 0.05$; $Chi^2 = 4.49$, df = 4 (P = 0.34); $I^2 = 11\%$
Test for overall effect: Z = 3.20 (P = 0.001)

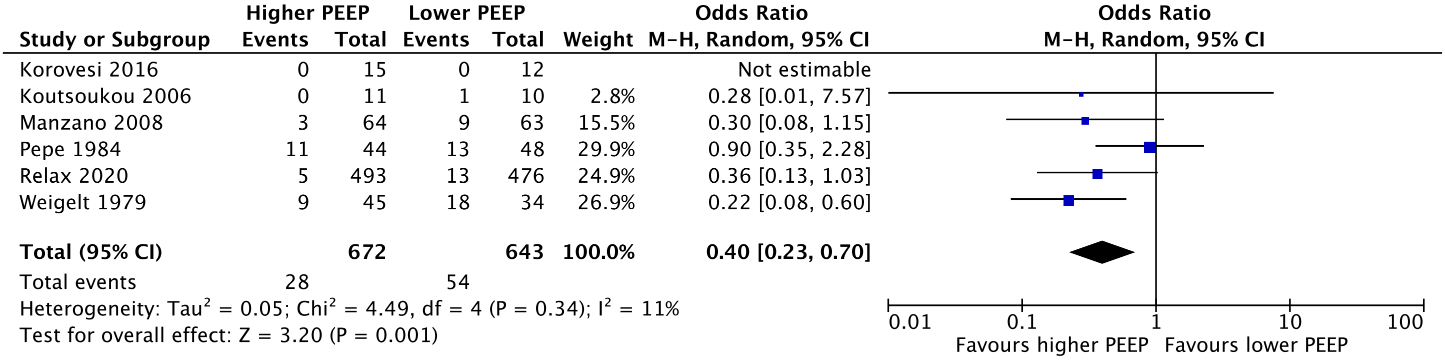

Sensitivity analysis (odds ratio)

Hypotension

Study or Subgroup	Higher PEEP Events	Higher PEEP Total	Lower PEEP Events	Lower PEEP Total	Weight	Odds Ratio M-H, Random, 95% CI
Feeley 1975	0	12	0	13		Not estimable
Good 1979	0	10	0	14		Not estimable
Lesur 2010	16	30	16	33	84.2%	1.21 [0.45, 3.27]
Pepe 1984	1	44	0	48	7.9%	3.34 [0.13, 84.28]
Weigelt 1979	1	45	0	34	7.9%	2.33 [0.09, 58.88]
**Total (95% CI)**		**141**		**142**	**100.0%**	**1.39 [0.56, 3.44]**
Total events	18		16			

Heterogeneity: $Tau^2 = 0.00$; $Chi^2 = 0.46$, df = 2 (P = 0.80); $I^2 = 0\%$
Test for overall effect: Z = 0.70 (P = 0.48)

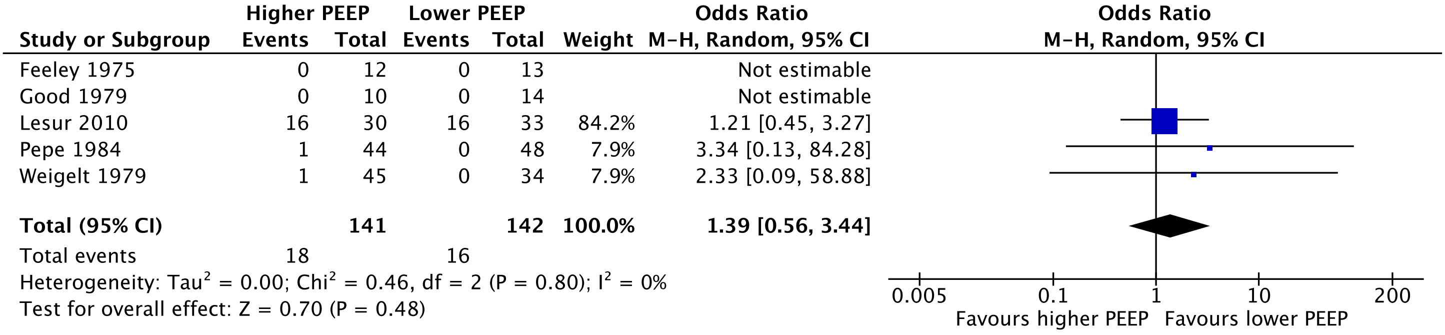

Sensitivity analysis (odds ratio)

ICU mortality

	Higher PEEP		Lower PEEP			Odds Ratio
Study or Subgroup	Events	Total	Events	Total	Weight	M-H, Random, 95% CI
Dyhr 2002	0	7	0	8		Not estimable
Feeley 1975	2	12	1	13	1.0%	2.40 [0.19, 30.52]
Korovesi 2011	3	15	4	12	2.1%	0.50 [0.09, 2.86]
Nelson 1987	4	20	4	18	2.7%	0.88 [0.18, 4.17]
Relax 2020	185	492	163	476	94.2%	1.16 [0.89, 1.51]
**Total (95% CI)**		**546**		**527**	**100.0%**	**1.14 [0.88, 1.47]**
Total events	194		172			

Heterogeneity: $Tau^2 = 0.00$; $Chi^2 = 1.31$, df = 3 (P = 0.73); $I^2 = 0\%$
Test for overall effect: Z = 0.98 (P = 0.33)

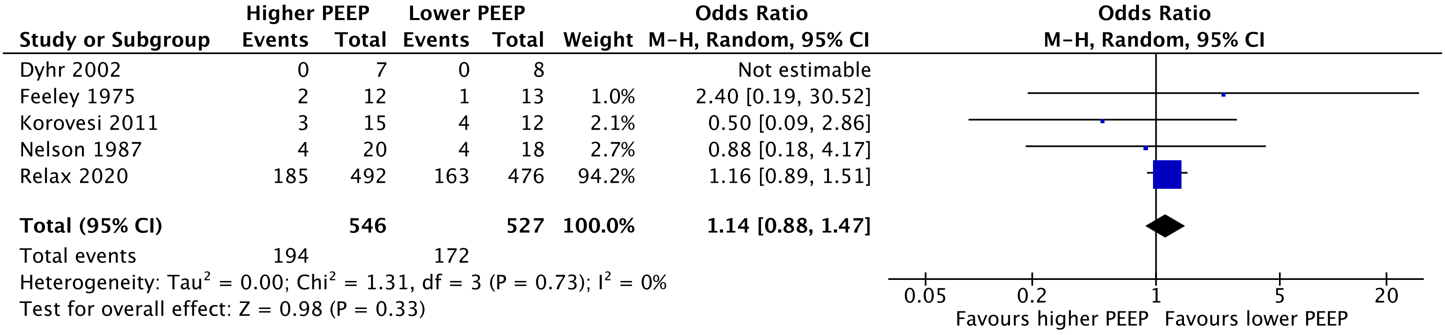

Sensitivity analysis (odds ratio)

28-day mortality

Study or Subgroup	Higher PEEP Events	Higher PEEP Total	Lower PEEP Events	Lower PEEP Total	Weight	Odds Ratio M-H, Random, 95% CI
Lesur 2010	9	30	14	33	29.9%	0.58 [0.21, 1.65]
Ma 2014	15	60	39	60	32.8%	0.18 [0.08, 0.40]
Relax 2020	207	493	183	476	37.3%	1.16 [0.90, 1.50]
**Total (95% CI)**		**583**		**569**	**100.0%**	**0.51 [0.15, 1.77]**
Total events	231		236			

Heterogeneity: $Tau^2 = 1.06$; $Chi^2 = 20.29$, df = 2 ($P < 0.0001$); $I^2 = 90\%$

Test for overall effect: $Z = 1.06$ ($P = 0.29$)

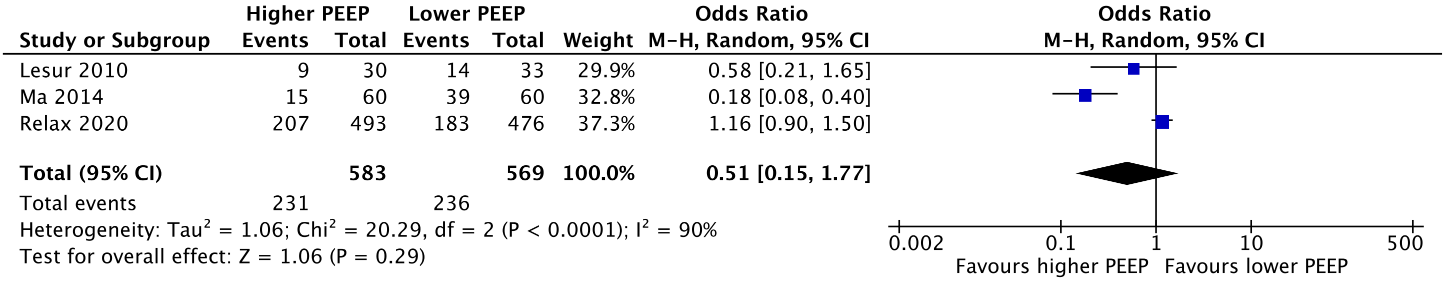

Sensitivity analysis (without high-risk studies)

Hospital mortality

Study or Subgroup	Higher PEEP Events	Higher PEEP Total	Lower PEEP Events	Lower PEEP Total	Weight	Risk Ratio M-H, Random, 95% CI
Collier 2002	1	40	1	44	0.3%	1.10 [0.07, 17.01]
Lesur 2010	12	30	16	33	6.4%	0.82 [0.47, 1.45]
Manzano 2008	19	64	16	63	6.3%	1.17 [0.66, 2.06]
Relax 2020	208	489	185	472	87.0%	1.09 [0.93, 1.26]
**Total (95% CI)**		**623**		**612**	**100.0%**	**1.07 [0.93, 1.24]**
Total events	240		218			

Heterogeneity: $Tau^2 = 0.00$; $Chi^2 = 0.95$, df = 3 (P = 0.81); $I^2 = 0\%$

Test for overall effect: Z = 0.95 (P = 0.34)

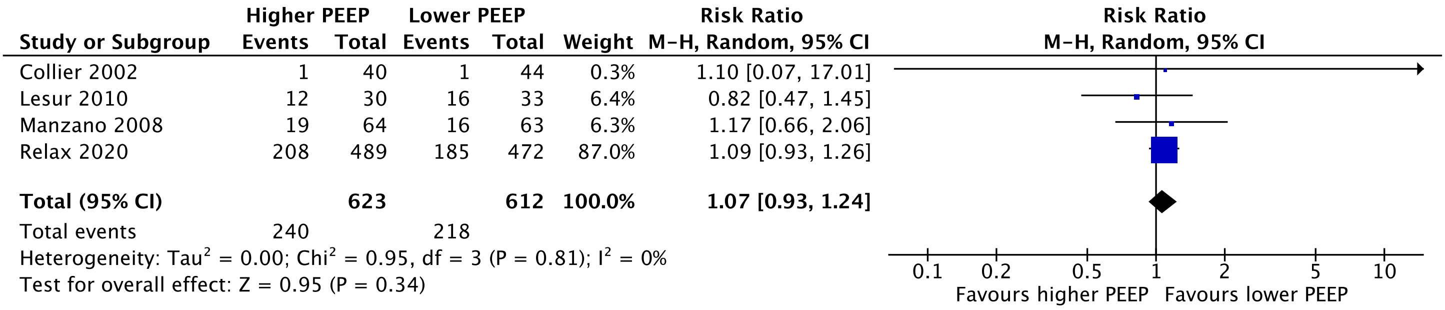

Sensitivity analysis (without high-risk studies)

PaO2/FiO2

	Higher PEEP			Lower PEEP				Mean Difference
**Study or Subgroup**	**Mean**	**SD**	**Total**	**Mean**	**SD**	**Total**	**Weight**	**IV, Random, 95% CI**
Holland 2007	307	82	14	337	82	14	5.0%	−30.00 [−90.75, 30.75]
Korovesi 2011	498	75	15	420	73	12	5.8%	78.00 [21.91, 134.09]
Lesur 2010	293	135	30	228	67	33	6.3%	65.00 [11.56, 118.44]
Ma 2014	196	45	60	134	22	60	34.4%	62.00 [49.33, 74.67]
Manzano 2008	359	104	64	301	84	63	13.7%	58.00 [25.15, 90.85]
Relax 2020	248	112	493	190	84	476	34.8%	58.00 [45.56, 70.44]
**Total (95% CI)**			**676**			**658**	**100.0%**	**56.55 [42.12, 70.98]**

Heterogeneity: $Tau^2 = 115.66$; $Chi^2 = 8.97$, df = 5 (P = 0.11); $I^2 = 44\%$
Test for overall effect: Z = 7.68 (P < 0.00001)

Mean Difference
IV, Random, 95% CI

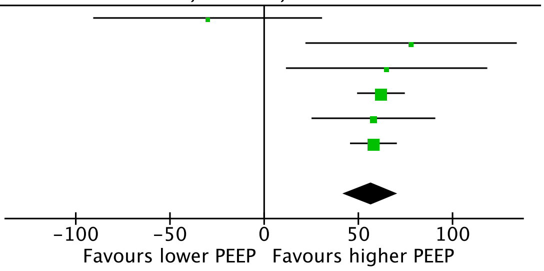

Sensitivity analysis (without high-risk studies)

Hypoxemia

Study or Subgroup	Higher PEEP Events	Higher PEEP Total	Lower PEEP Events	Lower PEEP Total	Weight	Risk Ratio M-H, Random, 95% CI
Manzano 2008	12	64	34	63	46.1%	0.35 [0.20, 0.61]
Relax 2020	87	493	98	476	53.9%	0.86 [0.66, 1.11]
**Total (95% CI)**		**557**		**539**	**100.0%**	**0.57 [0.23, 1.37]**
Total events	99		132			

Heterogeneity: $Tau^2 = 0.36$; $Chi^2 = 8.25$, df = 1 (P = 0.004); $I^2 = 88\%$
Test for overall effect: Z = 1.27 (P = 0.21)

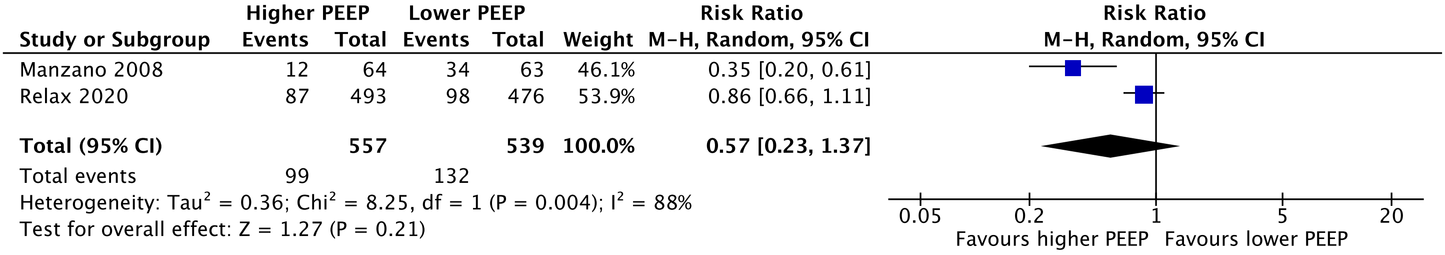

Sensitivity analysis (without high-risk studies)

Atelectasis

	Higher PEEP		Lower PEEP			Risk Ratio
Study or Subgroup	Events	Total	Events	Total	Weight	M-H, Random, 95% CI
Manzano 2008	12	64	17	63	50.4%	0.69 [0.36, 1.33]
Relax 2020	15	493	20	476	49.6%	0.72 [0.38, 1.40]
**Total (95% CI)**		**557**		**539**	**100.0%**	**0.71 [0.45, 1.13]**
Total events	27		37			

Heterogeneity: $Tau^2 = 0.00$; $Chi^2 = 0.01$, df = 1 (P = 0.93); $I^2 = 0\%$
Test for overall effect: Z = 1.45 (P = 0.15)

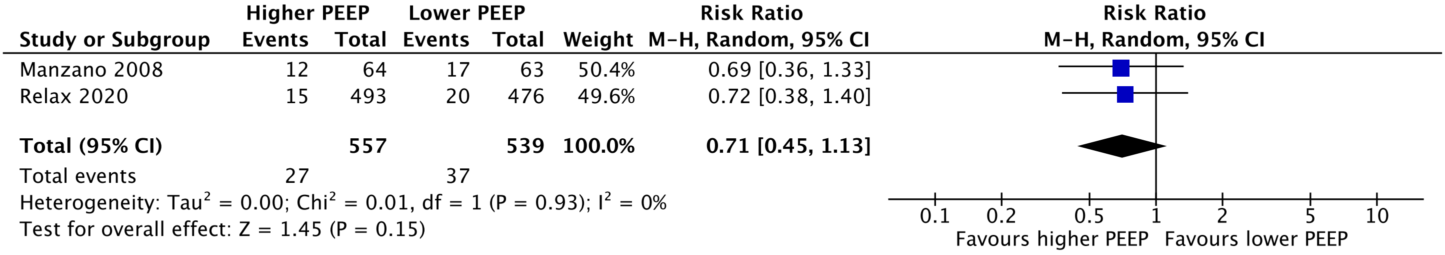

Sensitivity analysis (without high-risk studies)

Barotrauma

Study or Subgroup	Higher PEEP Events	Higher PEEP Total	Lower PEEP Events	Lower PEEP Total	Weight	Risk Ratio M-H, Random, 95% CI
Manzano 2008	1	64	5	63	10.1%	0.20 [0.02, 1.64]
Relax 2020	12	493	19	476	89.9%	0.61 [0.30, 1.24]
**Total (95% CI)**		**557**		**539**	**100.0%**	**0.54 [0.28, 1.07]**
Total events	13		24			

Heterogeneity: $Tau^2 = 0.00$; $Chi^2 = 0.99$, df = 1 (P = 0.32); $I^2 = 0\%$
Test for overall effect: Z = 1.77 (P = 0.08)

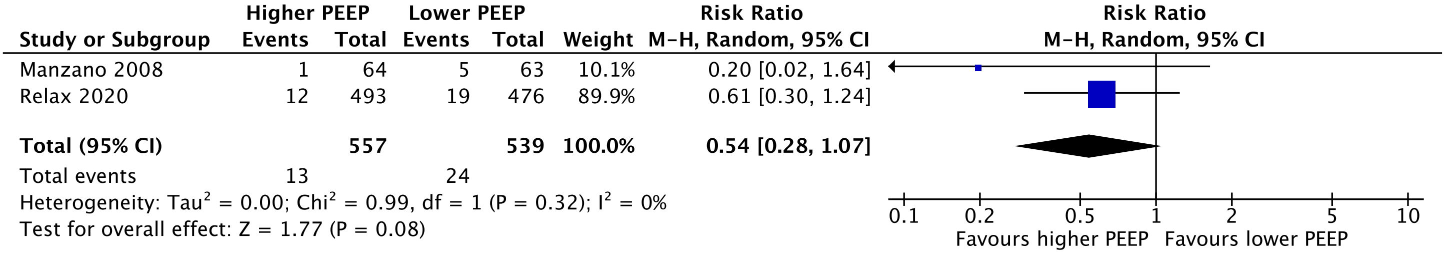

Sensitivity analysis (without high-risk studies)

Ventilator-associated pneumonia

Study or Subgroup	Higher PEEP Events	Higher PEEP Total	Lower PEEP Events	Lower PEEP Total	Weight	Risk Ratio M-H, Random, 95% CI
Manzano 2008	6	64	16	63	54.3%	0.37 [0.15, 0.88]
Relax 2020	7	493	6	476	45.7%	1.13 [0.38, 3.33]
**Total (95% CI)**		**557**		**539**	**100.0%**	**0.61 [0.21, 1.83]**
Total events	13		22			

Heterogeneity: $Tau^2 = 0.37$; $Chi^2 = 2.47$, df = 1 (P = 0.12); $I^2 = 60\%$
Test for overall effect: Z = 0.88 (P = 0.38)

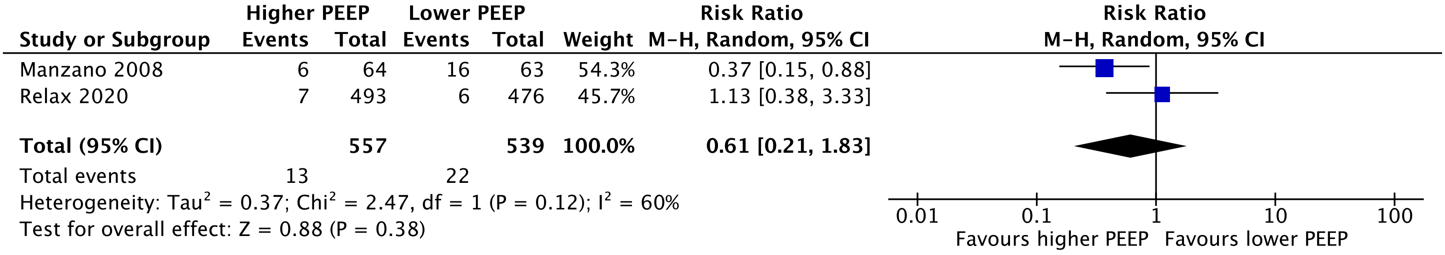

Sensitivity analysis (without high-risk studies)

ARDS

	Higher PEEP		Lower PEEP			Risk Ratio
Study or Subgroup	Events	Total	Events	Total	Weight	M-H, Random, 95% CI
Manzano 2008	3	64	9	63	39.8%	0.33 [0.09, 1.16]
Relax 2020	5	493	13	476	60.2%	0.37 [0.13, 1.03]
**Total (95% CI)**		**557**		**539**	**100.0%**	**0.35 [0.16, 0.78]**
Total events	8		22			

Heterogeneity: $Tau^2 = 0.00$; $Chi^2 = 0.02$, df = 1 (P = 0.88); $I^2 = 0\%$

Test for overall effect: Z = 2.57 (P = 0.01)

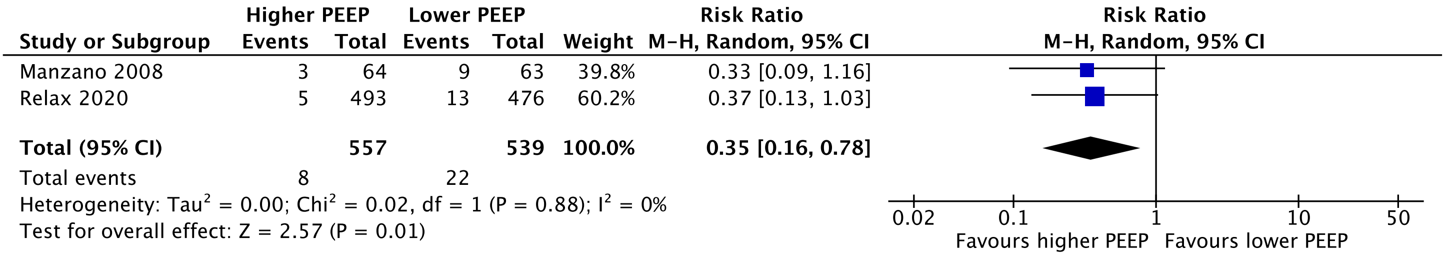

Sensitivity analysis (without high-risk studies)

Cardiac index

Study or Subgroup	Higher PEEP Mean	Higher PEEP SD	Higher PEEP Total	Lower PEEP Mean	Lower PEEP SD	Lower PEEP Total	Weight	Mean Difference IV, Random, 95% CI
Collier 2002	3.1	0.86	40	3.1	0.64	44	64.9%	0.00 [−0.33, 0.33]
Holland 2007	3	0.6	14	2.9	0.6	14	35.1%	0.10 [−0.34, 0.54]
**Total (95% CI)**			**54**			**58**	**100.0%**	**0.04 [−0.23, 0.30]**

Heterogeneity: $Tau^2 = 0.00$; $Chi^2 = 0.13$, df = 1 (P = 0.72); $I^2 = 0\%$
Test for overall effect: Z = 0.26 (P = 0.79)

Mean Difference IV, Random, 95% CI

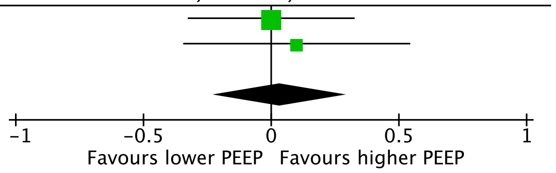

Sensitivity analysis (without high-risk studies)

Central venous pressure

	Higher PEEP			Lower PEEP				Mean Difference
**Study or Subgroup**	**Mean**	**SD**	**Total**	**Mean**	**SD**	**Total**	**Weight**	**IV, Random, 95% CI**
Holland 2007	9	3	14	9	3	14	33.5%	0.00 [−2.22, 2.22]
Lesur 2010	12.5	1.5	30	10.8	2.9	33	66.5%	1.70 [0.57, 2.83]
**Total (95% CI)**			**44**			**47**	**100.0%**	**1.13 [−0.44, 2.70]**

Heterogeneity: $Tau^2 = 0.64$; $Chi^2 = 1.79$, df = 1 (P = 0.18); $I^2 = 44\%$
Test for overall effect: Z = 1.41 (P = 0.16)

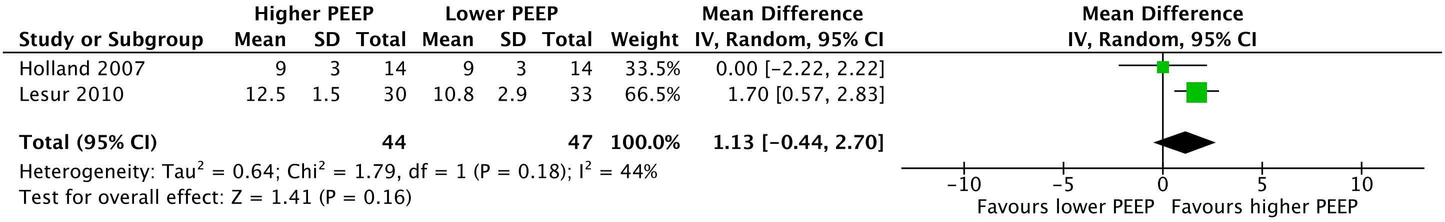

Sensitivity analysis (without high-risk studies)

Duration of ventilation

Study or Subgroup	Higher PEEP Mean	Higher PEEP SD	Higher PEEP Total	Lower PEEP Mean	Lower PEEP SD	Lower PEEP Total	Weight	Std. Mean Difference IV, Random, 95% CI
Collier 2002	409.1	208.6	40	439.3	277.7	44	6.7%	−0.12 [−0.55, 0.31]
Lesur 2010	9.2	8.8	30	9.2	8.5	33	5.1%	0.00 [−0.49, 0.49]
Manzano 2008	5.8	6.8	64	6.5	6.2	63	10.2%	−0.11 [−0.45, 0.24]
Relax 2020	4.8	6.6	493	5.5	7.4	476	78.0%	−0.10 [−0.23, 0.03]
**Total (95% CI)**			**627**			**616**	**100.0%**	**−0.10 [−0.21, 0.01]**

Heterogeneity: $Tau^2 = 0.00$; $Chi^2 = 0.17$, df = 3 (P = 0.98); $I^2 = 0\%$
Test for overall effect: Z = 1.71 (P = 0.09)

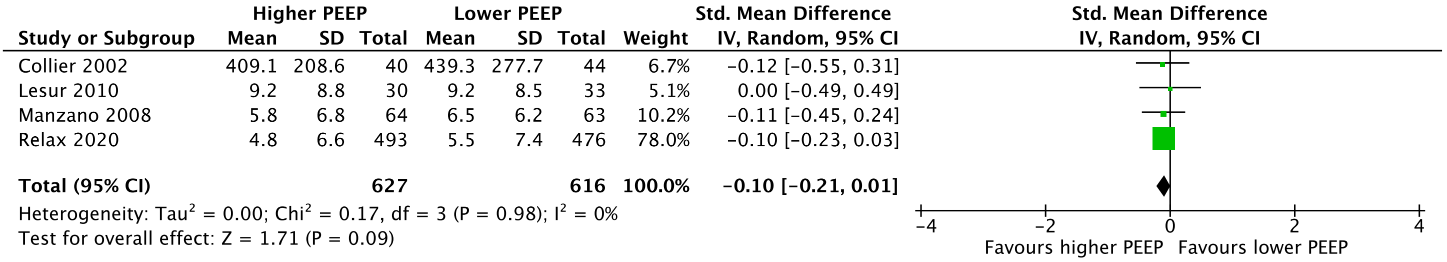

Sensitivity analysis (without high-risk studies)

ICU stay

Study or Subgroup	Higher PEEP Mean	Higher PEEP SD	Higher PEEP Total	Lower PEEP Mean	Lower PEEP SD	Lower PEEP Total	Weight	Mean Difference IV, Random, 95% CI
Korovesi 2011	17.2	10.1	15	14.4	8.44	12	3.3%	2.80 [−4.19, 9.79]
Manzano 2008	10.5	9.8	64	12.3	11.4	63	11.8%	−1.80 [−5.50, 1.90]
Relax 2020	7.2	10.3	493	8.1	11.5	476	85.0%	−0.90 [−2.28, 0.48]
**Total (95% CI)**			**572**			**551**	**100.0%**	**−0.88 [−2.15, 0.38]**

Heterogeneity: $Tau^2 = 0.00$; $Chi^2 = 1.30$, df = 2 (P = 0.52); $I^2 = 0\%$
Test for overall effect: Z = 1.37 (P = 0.17)

Mean Difference IV, Random, 95% CI

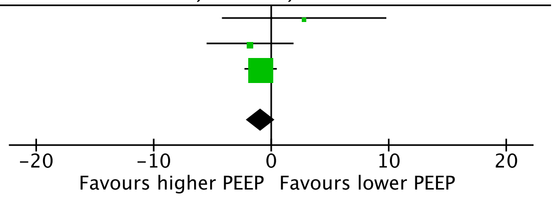

Sensitivity analysis (without high-risk studies)

Hospital stay

	Higher PEEP			Lower PEEP				Mean Difference
**Study or Subgroup**	**Mean**	**SD**	**Total**	**Mean**	**SD**	**Total**	**Weight**	**IV, Random, 95% CI**
Collier 2002	5.7	2.5	40	5.2	1.7	44	55.2%	0.50 [−0.42, 1.42]
Manzano 2008	19.5	18.2	64	26.3	22	63	10.3%	−6.80 [−13.83, 0.23]
Relax 2020	19	21.4	493	19.9	22.1	476	34.5%	−0.90 [−3.64, 1.84]
**Total (95% CI)**			**597**			**583**	**100.0%**	**−0.73 [−3.21, 1.74]**

Heterogeneity: $Tau^2 = 2.67$; $Chi^2 = 4.82$, df = 2 (P = 0.09); $I^2 = 59\%$
Test for overall effect: Z = 0.58 (P = 0.56)

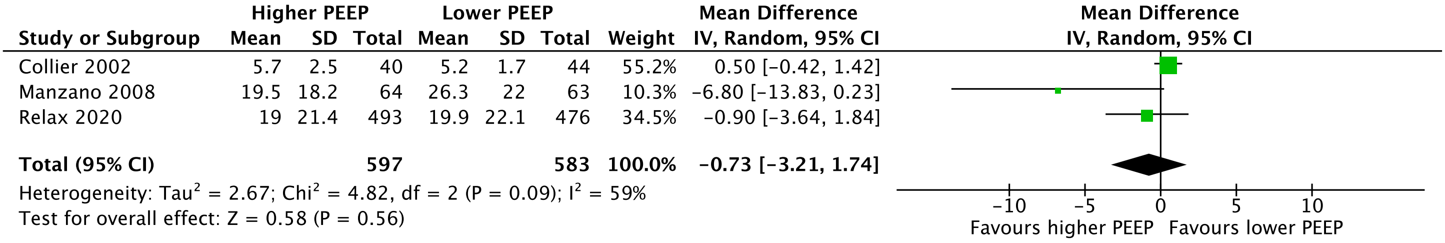

Sensitivity analysis (without high-risk studies)

ICU mortality

	Higher PEEP		Lower PEEP			Risk Ratio	Risk Ratio
Study or Subgroup	Events	Total	Events	Total	Weight	M-H, Random, 95% CI	M-H, Random, 95% CI
Korovesi 2011	3	15	4	12	1.7%	0.60 [0.17, 2.18]	
Relax 2020	185	492	163	476	98.3%	1.10 [0.93, 1.30]	
**Total (95% CI)**		**507**		**488**	**100.0%**	**1.09 [0.92, 1.28]**	
Total events	188		167				

Heterogeneity: $Tau^2 = 0.00$; $Chi^2 = 0.83$, df = 1 (P = 0.36); $I^2 = 0\%$
Test for overall effect: Z = 0.98 (P = 0.33)

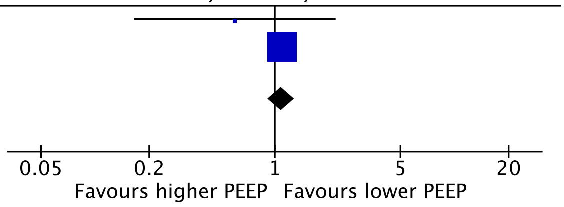

Sensitivity analysis (without high-risk studies)

28-day mortality

Study or Subgroup	Higher PEEP Events	Higher PEEP Total	Lower PEEP Events	Lower PEEP Total	Weight	Risk Ratio M-H, Random, 95% CI
Lesur 2010	9	30	14	33	28.8%	0.71 [0.36, 1.39]
Ma 2014	15	60	39	60	33.1%	0.38 [0.24, 0.62]
Relax 2020	207	493	183	476	38.1%	1.09 [0.94, 1.27]
**Total (95% CI)**		**583**		**569**	**100.0%**	**0.68 [0.33, 1.40]**
Total events	231		236			

Heterogeneity: $Tau^2 = 0.35$; $Chi^2 = 17.71$, df = 2 (P = 0.0001); $I^2 = 89\%$
Test for overall effect: Z = 1.05 (P = 0.30)

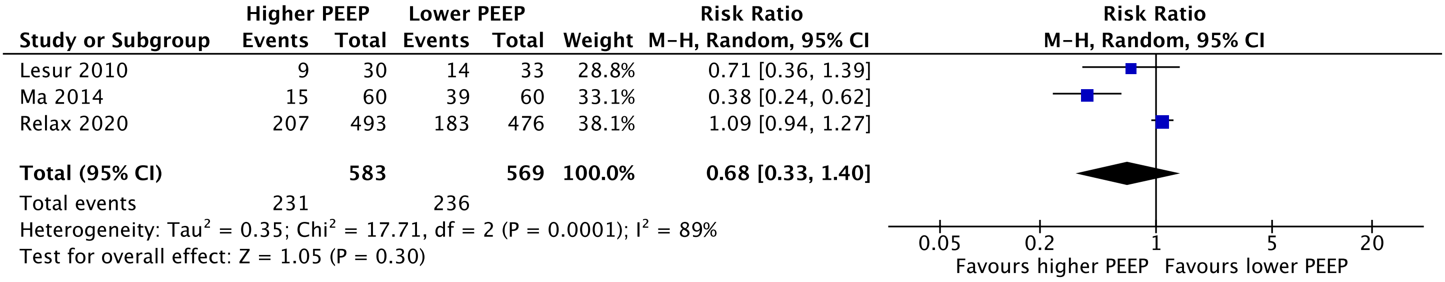

Online Resource 9. Subgroup Analyses

Subgroup analyses: medical vs. surgical patients

Subgroup analyses according to the inclusion of medical or surgical patients in the studies. No subgroup difference was observed.

Abbreviations: M-H, Mantel–Haenszel; CI, confidence interval; PaO_2/FiO_2, arterial partial pressure of oxygen to fraction of inspired oxygen ratio; IV, inverse variance; A-aDO_2, alveolar-arterial oxygen pressure difference; CVP, central venous pressure; PRBC, packed red blood cell; ICU, intensive care unit.

Outcome or Subgroup	Studies	Participants	Statistical Method	Effect Estimate
1.1 Hospital mortality	9	1502	Risk Ratio (M-H, Random, 95% CI)	1.02 [0.89, 1.16]
1.1.1 Medical	6	1360	Risk Ratio (M-H, Random, 95% CI)	1.02 [0.89, 1.16]
1.1.2 Surgical	3	142	Risk Ratio (M-H, Random, 95% CI)	1.10 [0.07, 17.01]
1.2 PaO_2/FiO_2	8	1444	Mean Difference (IV, Random, 95% CI)	50.46 [33.93, 66.99]
1.2.1 Medical	6	1327	Mean Difference (IV, Random, 95% CI)	56.58 [42.29, 70.87]
1.2.2 Surgical	2	117	Mean Difference (IV, Random, 95% CI)	17.50 [-68.47, 103.46]
1.3 A-aDO_2	4	164	Std. Mean Difference (IV, Random, 95% CI)	-1.62 [-3.12, -0.11]
1.3.1 Medical	2	46	Std. Mean Difference (IV, Random, 95% CI)	-1.33 [-4.58, 1.93]
1.3.2 Surgical	2	118	Std. Mean Difference (IV, Random, 95% CI)	-1.97 [-4.66, 0.73]
1.4 Compliance	3	189	Mean Difference (IV, Random, 95% CI)	8.46 [3.11, 13.82]

1.4.1 Medical	2	100	Mean Difference (IV, Random, 95% CI)	7.41 [-1.78, 16.61]
1.4.2 Surgical	1	89	Mean Difference (IV, Random, 95% CI)	9.00 [2.41, 15.59]
1.5 Hypoxemia	5	1320	Risk Ratio (M-H, Random, 95% CI)	0.60 [0.40, 0.92]
1.5.1 Medical	3	1188	Risk Ratio (M-H, Random, 95% CI)	0.52 [0.24, 1.16]
1.5.2 Surgical	2	132	Risk Ratio (M-H, Random, 95% CI)	0.63 [0.43, 0.93]
1.6 Atelectasis	5	1255	Risk Ratio (M-H, Random, 95% CI)	1.02 [0.81, 1.28]
1.6.1 Medical	3	1188	Risk Ratio (M-H, Random, 95% CI)	0.92 [0.58, 1.46]
1.6.2 Surgical	2	67	Risk Ratio (M-H, Random, 95% CI)	1.05 [0.78, 1.41]
1.7 Barotrauma	7	1372	Risk Ratio (M-H, Random, 95% CI)	0.78 [0.55, 1.11]
1.7.1 Medical	5	1305	Risk Ratio (M-H, Random, 95% CI)	0.78 [0.55, 1.11]
1.7.2 Surgical	2	67	Risk Ratio (M-H, Random, 95% CI)	Not estimable
1.8 Hypotension	5	283	Risk Ratio (M-H, Random, 95% CI)	1.15 [0.71, 1.84]
1.8.1 Medical	4	259	Risk Ratio (M-H, Random, 95% CI)	1.15 [0.71, 1.84]
1.8.2 Surgical	1	24	Risk Ratio (M-H, Random, 95% CI)	Not estimable
1.9 CVP	3	106	Mean Difference (IV, Random, 95% CI)	1.37 [0.38, 2.37]
1.9.1 Medical	1	63	Mean Difference (IV, Random, 95% CI)	1.70 [0.57, 2.83]
1.9.2 Surgical	2	43	Mean Difference (IV, Random, 95% CI)	0.17 [-1.99, 2.33]
1.10 PRBC transfusion	3	1138	Mean Difference (IV, Random, 95% CI)	-0.38 [-0.77, 0.02]

1.10.1 Medical	1	969	Mean Difference (IV, Random, 95% CI)	Not estimable
1.10.2 Surgical	2	169	Mean Difference (IV, Random, 95% CI)	-0.38 [-0.77, 0.02]
1.11 Duration of ventilation	10	1510	Std. Mean Difference (IV, Random, 95% CI)	-0.03 [-0.27, 0.21]
1.11.1 Medical	6	1301	Std. Mean Difference (IV, Random, 95% CI)	-0.09 [-0.20, 0.02]
1.11.2 Surgical	4	209	Std. Mean Difference (IV, Random, 95% CI)	0.28 [-0.71, 1.27]
1.12 Hospital stay	5	1245	Mean Difference (IV, Random, 95% CI)	-0.02 [-0.69, 0.66]
1.12.1 Medical	3	1134	Mean Difference (IV, Random, 95% CI)	-2.11 [-5.95, 1.72]
1.12.2 Surgical	2	111	Mean Difference (IV, Random, 95% CI)	0.04 [-0.46, 0.54]
1.13 ICU mortality	5	1073	Risk Ratio (M-H, Random, 95% CI)	1.09 [0.92, 1.28]
1.13.1 Medical	4	1058	Risk Ratio (M-H, Random, 95% CI)	1.09 [0.92, 1.28]
1.13.2 Surgical	1	15	Risk Ratio (M-H, Random, 95% CI)	Not estimable

Subgroup analyses: zero end-expiratory pressure (ZEEP) vs. positive end-expiratory pressure (PEEP) different from ZEEP as lower PEEP

Subgroup analyses according to the use of zero end-expiratory pressure (ZEEP) or positive end-expiratory pressure (PEEP) different from ZEEP as lower PEEP in the studies. A significantly lower incidence of hypoxemia with higher PEEP in studies comparing higher PEEP with ZEEP vs. studies comparing higher PEEP with lower PEEP different from ZEEP ($p = 0.02$) was observed.

Abbreviations: M-H, Mantel–Haenszel; CI, confidence interval; PaO_2/FiO_2, arterial partial pressure of oxygen to fraction of inspired oxygen ratio; IV, inverse variance; A-aDO_2, alveolar-arterial oxygen pressure difference; ARDS, acute respiratory distress syndrome; CVP, central venous pressure; PRBC, packed red blood cell; ICU, intensive care unit.

Outcome or Subgroup	Studies	Participants	Statistical Method	Effect Estimate
2.1 Hospital mortality	9	1502	Risk Ratio (M-H, Random, 95% CI)	1.02 [0.89, 1.16]
2.1.1 ZEEP	6	419	Risk Ratio (M-H, Random, 95% CI)	0.85 [0.64, 1.12]
2.1.2 Lower PEEP	3	1083	Risk Ratio (M-H, Random, 95% CI)	1.08 [0.93, 1.25]
2.2 PaO_2/FiO_2	8	1444	Mean Difference (IV, Random, 95% CI)	50.46 [33.93, 66.99]
2.2.1 ZEEP	4	238	Mean Difference (IV, Random, 95% CI)	45.75 [5.42, 86.09]
2.2.2 Lower PEEP	4	1206	Mean Difference (IV, Random, 95% CI)	52.97 [34.89, 71.05]
2.3 A-aDO_2	4	164	Std. Mean Difference (IV, Random, 95% CI)	-1.62 [-3.12, -0.11]
2.3.1 ZEEP	2	46	Std. Mean Difference (IV, Random, 95% CI)	-1.33 [-4.58, 1.93]
2.3.2 Lower PEEP	2	118	Std. Mean Difference (IV, Random, 95% CI)	-1.97 [-4.66, 0.73]
2.4 Compliance	3	189	Mean Difference (IV, Random, 95% CI)	8.46 [3.11, 13.82]
2.4.1 ZEEP	2	100	Mean Difference (IV, Random, 95% CI)	7.41 [-1.78, 16.61]
2.4.2 Lower PEEP	1	89	Mean Difference (IV, Random, 95% CI)	9.00 [2.41, 15.59]
2.5 Hypoxemia	5	1320	Risk Ratio (M-H, Random, 95% CI)	0.60 [0.40, 0.92]
2.5.1 ZEEP	3	262	Risk Ratio (M-H, Random, 95% CI)	0.37 [0.22, 0.63]
2.5.2 Lower PEEP	2	1058	Risk Ratio (M-H, Random, 95% CI)	0.75 [0.55, 1.04]
2.6 Atelectasis	5	1255	Risk Ratio (M-H, Random, 95% CI)	1.02 [0.81, 1.28]

2.6.1 ZEEP	4	286	Risk Ratio (M-H, Random, 95% CI)	1.07 [0.86, 1.33]
2.6.2 Lower PEEP	1	969	Risk Ratio (M-H, Random, 95% CI)	0.72 [0.38, 1.40]
2.7 Barotrauma	7	1372	Risk Ratio (M-H, Random, 95% CI)	0.78 [0.55, 1.11]
2.7.1 ZEEP	5	365	Risk Ratio (M-H, Random, 95% CI)	0.83 [0.55, 1.24]
2.7.2 Lower PEEP	2	1007	Risk Ratio (M-H, Random, 95% CI)	0.66 [0.33, 1.31]
2.8 Ventilator-associated pneumonia	3	1188	Risk Ratio (M-H, Random, 95% CI)	0.62 [0.32, 1.23]
2.8.1 ZEEP	2	219	Risk Ratio (M-H, Random, 95% CI)	0.47 [0.23, 0.94]
2.8.2 Lower PEEP	1	969	Risk Ratio (M-H, Random, 95% CI)	1.13 [0.38, 3.33]
2.9 ARDS	6	1315	Risk Ratio (M-H, Random, 95% CI)	0.50 [0.32, 0.78]
2.9.1 ZEEP	5	346	Risk Ratio (M-H, Random, 95% CI)	0.52 [0.29, 0.92]
2.9.2 Lower PEEP	1	969	Risk Ratio (M-H, Random, 95% CI)	0.37 [0.13, 1.03]
2.10 Cardiac index	3	127	Mean Difference (IV, Random, 95% CI)	0.04 [-0.21, 0.29]
2.10.1 ZEEP	1	15	Mean Difference (IV, Random, 95% CI)	0.10 [-0.78, 0.98]
2.10.2 Lower PEEP	2	112	Mean Difference (IV, Random, 95% CI)	0.04 [-0.23, 0.30]
2.11 CVP	3	106	Mean Difference (IV, Random, 95% CI)	1.37 [0.38, 2.37]
2.11.1 ZEEP	2	78	Mean Difference (IV, Random, 95% CI)	1.72 [0.60, 2.84]
2.11.2 Lower PEEP	1	28	Mean Difference (IV, Random, 95% CI)	0.00 [-2.22, 2.22]
2.12 Postoperative bleeding	2	601	Mean Difference (IV, Random, 95% CI)	26.47 [-99.95, 152.89]

2.12.1 ZEEP	1	517	Mean Difference (IV, Random, 95% CI)	-20.00 [-96.82, 56.82]
2.12.2 Lower PEEP	1	84	Mean Difference (IV, Random, 95% CI)	116.00 [-52.52, 284.52]
2.13 PRBC transfusion	3	1138	Mean Difference (IV, Random, 95% CI)	-0.38 [-0.77, 0.02]
2.13.1 ZEEP	1	85	Mean Difference (IV, Random, 95% CI)	-0.42 [-0.92, 0.08]
2.13.2 Lower PEEP	2	1053	Mean Difference (IV, Random, 95% CI)	-0.30 [-0.94, 0.34]
2.14 Duration of ventilation	10	1510	Std. Mean Difference (IV, Random, 95% CI)	-0.03 [-0.27, 0.21]
2.14.1 ZEEP	5	318	Std. Mean Difference (IV, Random, 95% CI)	0.15 [-0.33, 0.63]
2.14.2 Lower PEEP	5	1192	Std. Mean Difference (IV, Random, 95% CI)	-0.13 [-0.41, 0.15]
2.15 ICU stay	4	1202	Mean Difference (IV, Random, 95% CI)	-1.00 [-2.51, 0.51]
2.15.1 ZEEP	3	233	Mean Difference (IV, Random, 95% CI)	-1.41 [-6.15, 3.32]
2.15.2 Lower PEEP	1	969	Mean Difference (IV, Random, 95% CI)	-0.90 [-2.28, 0.48]
2.16 Hospital stay	5	1245	Mean Difference (IV, Random, 95% CI)	-0.02 [-0.69, 0.66]
2.16.1 ZEEP	1	127	Mean Difference (IV, Random, 95% CI)	-6.80 [-13.83, 0.23]
2.16.2 Lower PEEP	4	1118	Mean Difference (IV, Random, 95% CI)	-0.04 [-0.36, 0.29]
2.17 ICU mortality	5	1073	Risk Ratio (M-H, Random, 95% CI)	1.09 [0.92, 1.28]
2.17.1 ZEEP	3	67	Risk Ratio (M-H, Random, 95% CI)	0.82 [0.27, 2.52]
2.17.2 Lower PEEP	2	1006	Risk Ratio (M-H, Random, 95% CI)	1.09 [0.93, 1.29]
2.18 28-day mortality	3	1152	Risk Ratio (M-H, Random, 95% CI)	0.68 [0.33, 1.40]

2.18.1 ZEEP	1	63	Risk Ratio (M-H, Random, 95% CI)	0.71 [0.36, 1.39]
2.18.2 Lower PEEP	2	1089	Risk Ratio (M-H, Random, 95% CI)	0.66 [0.24, 1.85]

Subgroup analyses: tidal volume > 8 mL/kg vs. tidal volume < 8 mL/kg

Subgroup analyses according to the use of tidal volumes (TV) greater than or lower than 8 mL/kg in the studies. We observed a significantly lower alveolar-arterial oxygen pressure difference (A-aDO_2) with higher PEEP in studies using tidal volumes > 8 mL/kg vs. studies using tidal volumes < 8 mL/kg ($p < 0.01$) and a trend towards a reduction of hospital mortality ($p = 0.09$) and atelectasis ($p = 0.08$) with higher PEEP in studies using tidal volumes > 8 mL/kg and < 8 mL/kg, respectively.

Abbreviations: M-H, Mantel–Haenszel; CI, confidence interval; IV, inverse variance; ARDS, acute respiratory distress syndrome; ICU, intensive care unit.

Outcome or Subgroup	Studies	Participants	Statistical Method	Effect Estimate
3.1 Hospital mortality	7	1421	Risk Ratio (M-H, Random, 95% CI)	1.03 [0.90, 1.17]
3.1.1 VT>8	3	255	Risk Ratio (M-H, Random, 95% CI)	0.75 [0.51, 1.10]
3.1.2 VT<8	4	1166	Risk Ratio (M-H, Random, 95% CI)	1.07 [0.93, 1.24]
3.2 A-aDO_2	4	164	Std. Mean Difference (IV, Random, 95% CI)	-1.62 [-3.12, -0.11]
3.2.1 VT>8	2	54	Std. Mean Difference (IV, Random, 95% CI)	-3.21 [-4.06, -2.36]
3.2.2 VT<8	2	110	Std. Mean Difference (IV, Random, 95% CI)	-0.25 [-1.18, 0.68]
3.3 Compliance	3	189	Mean Difference (IV, Random, 95% CI)	8.46 [3.11, 13.82]
3.3.1 VT>8	1	79	Mean Difference (IV, Random, 95% CI)	3.00 [-14.88, 20.88]

3.3.2 VT<8	2	110	Mean Difference (IV, Random, 95% CI)	9.00 [3.39, 14.61]
3.4 Hypoxemia	4	1277	Risk Ratio (M-H, Random, 95% CI)	0.58 [0.37, 0.92]
3.4.1 VT>8	1	92	Risk Ratio (M-H, Random, 95% CI)	0.27 [0.03, 2.35]
3.4.2 VT<8	3	1185	Risk Ratio (M-H, Random, 95% CI)	0.60 [0.37, 0.96]
3.5 Atelectasis	4	1212	Risk Ratio (M-H, Random, 95% CI)	1.00 [0.76, 1.32]
3.5.1 VT>8	2	116	Risk Ratio (M-H, Random, 95% CI)	1.13 [0.90, 1.43]
3.5.2 VT<8	2	1096	Risk Ratio (M-H, Random, 95% CI)	0.71 [0.45, 1.13]
3.6 Barotrauma	5	1291	Risk Ratio (M-H, Random, 95% CI)	0.77 [0.54, 1.09]
3.6.1 VT>8	3	195	Risk Ratio (M-H, Random, 95% CI)	0.87 [0.58, 1.32]
3.6.2 VT<8	2	1096	Risk Ratio (M-H, Random, 95% CI)	0.54 [0.28, 1.07]
3.7 Ventilator-associated pneumonia	3	1188	Risk Ratio (M-H, Random, 95% CI)	0.62 [0.32, 1.23]
3.7.1 VT>8	1	92	Risk Ratio (M-H, Random, 95% CI)	0.73 [0.22, 2.41]
3.7.2 VT<8	2	1096	Risk Ratio (M-H, Random, 95% CI)	0.61 [0.21, 1.83]
3.8 ARDS	6	1315	Risk Ratio (M-H, Random, 95% CI)	0.50 [0.32, 0.78]
3.8.1 VT>8	2	171	Risk Ratio (M-H, Random, 95% CI)	0.59 [0.24, 1.41]
3.8.2 VT<8	4	1144	Risk Ratio (M-H, Random, 95% CI)	0.35 [0.16, 0.76]
3.9 Hypotension	5	283	Risk Ratio (M-H, Random, 95% CI)	1.15 [0.71, 1.84]
3.9.1 VT>8	4	220	Risk Ratio (M-H, Random, 95% CI)	2.73 [0.29, 25.73]

3.9.2 VT<8	1	63	Risk Ratio (M-H, Random, 95% CI)	1.10 [0.68, 1.79]
3.10 Cardiac index	3	127	Mean Difference (IV, Random, 95% CI)	0.04 [-0.21, 0.29]
3.10.1 VT>8	1	84	Mean Difference (IV, Random, 95% CI)	0.00 [-0.33, 0.33]
3.10.2 VT<8	2	43	Mean Difference (IV, Random, 95% CI)	0.10 [-0.30, 0.50]
3.11 Duration of ventilation	9	1472	Std. Mean Difference (IV, Random, 95% CI)	-0.07 [-0.32, 0.17]
3.11.1 VT>8	5	224	Std. Mean Difference (IV, Random, 95% CI)	0.25 [-0.44, 0.93]
3.11.2 VT<8	4	1248	Std. Mean Difference (IV, Random, 95% CI)	-0.16 [-0.34, 0.02]
3.12 ICU stay	4	1202	Mean Difference (IV, Random, 95% CI)	-1.00 [-2.51, 0.51]
3.12.1 VT>8	1	79	Mean Difference (IV, Random, 95% CI)	-9.30 [-21.28, 2.68]
3.12.2 VT<8	3	1123	Mean Difference (IV, Random, 95% CI)	-0.88 [-2.15, 0.38]
3.13 Hospital stay	4	1207	Mean Difference (IV, Random, 95% CI)	-0.04 [-0.84, 0.76]
3.13.1 VT>8	2	111	Mean Difference (IV, Random, 95% CI)	0.04 [-0.46, 0.54]
3.13.2 VT<8	2	1096	Mean Difference (IV, Random, 95% CI)	-2.93 [-8.42, 2.56]
3.14 ICU mortality	4	1035	Risk Ratio (M-H, Random, 95% CI)	1.09 [0.92, 1.29]
3.14.1 VT>8	1	25	Risk Ratio (M-H, Random, 95% CI)	2.17 [0.22, 20.94]
3.14.2 VT<8	3	1010	Risk Ratio (M-H, Random, 95% CI)	1.09 [0.92, 1.28]

Subgroup analyses: studies published before 2000 vs. studies published after 2000

Subgroup analyses according to year of publication (before 2000 vs. after 2000). A significantly lower alveolar-arterial oxygen pressure difference (A-aDO_2) with higher PEEP in studies published before 2000 ($p < 0.01$) was detected. Further, a trend towards lower atelectasis occurrence with higher PEEP in studies published after 2000 ($p = 0.08$) and towards lower hospital mortality with higher PEEP in studies published before 2000 ($p = 0.07$) was observed.

Abbreviations: IV, inverse variance; CI, confidence interval; M-H, Mantel–Haenszel; ARDS, acute respiratory distress syndrome; PRBC, packed red blood cell; ICU, intensive care unit.

Outcome or Subgroup	Studies	Participants	Statistical Method	Effect Estimate
4.1 Hospital mortality	9	1502	Risk Ratio (M-H, Random, 95% CI)	1.02 [0.89, 1.16]
4.1.1 >2000	5	1250	Risk Ratio (M-H, Random, 95% CI)	1.07 [0.93, 1.24]
4.1.2 <2000	4	252	Risk Ratio (M-H, Random, 95% CI)	0.74 [0.52, 1.07]
4.2 A-aDO2	4	164	Std. Mean Difference (IV, Random, 95% CI)	-1.62 [-3.12, -0.11]
4.2.1 >2000	2	110	Std. Mean Difference (IV, Random, 95% CI)	-0.25 [-1.18, 0.68]
4.2.2 <2000	2	54	Std. Mean Difference (IV, Random, 95% CI)	-3.21 [-4.06, -2.36]
4.3 Compliance	3	189	Mean Difference (IV, Random, 95% CI)	8.46 [3.11, 13.82]
4.3.1 >2000	2	110	Mean Difference (IV, Random, 95% CI)	9.00 [3.39, 14.61]
4.3.2 <2000	1	79	Mean Difference (IV, Random, 95% CI)	3.00 [-14.88, 20.88]
4.4 Hypoxemia	5	1320	Risk Ratio (M-H, Random, 95% CI)	0.60 [0.40, 0.92]

4.4.1 >2000	3	1185	Risk Ratio (M-H, Random, 95% CI)	0.60 [0.37, 0.96]
4.4.2 <2000	2	135	Risk Ratio (M-H, Random, 95% CI)	0.59 [0.14, 2.41]
4.5 Atelectasis	5	1255	Risk Ratio (M-H, Random, 95% CI)	1.02 [0.81, 1.28]
4.5.1 >2000	2	1096	Risk Ratio (M-H, Random, 95% CI)	0.71 [0.45, 1.13]
4.5.2 <2000	3	159	Risk Ratio (M-H, Random, 95% CI)	1.13 [0.90, 1.43]
4.6 Barotrauma	7	1372	Risk Ratio (M-H, Random, 95% CI)	0.78 [0.55, 1.11]
4.6.1 >2000	2	1096	Risk Ratio (M-H, Random, 95% CI)	0.54 [0.28, 1.07]
4.6.2 <2000	5	276	Risk Ratio (M-H, Random, 95% CI)	0.89 [0.59, 1.34]
4.7 Ventilator-associated pneumonia	3	1188	Risk Ratio (M-H, Random, 95% CI)	0.62 [0.32, 1.23]
4.7.1 >2000	2	1096	Risk Ratio (M-H, Random, 95% CI)	0.61 [0.21, 1.83]
4.7.2 <2000	1	92	Risk Ratio (M-H, Random, 95% CI)	0.73 [0.22, 2.41]
4.8 ARDS	6	1315	Risk Ratio (M-H, Random, 95% CI)	0.50 [0.32, 0.78]
4.8.1 >2000	4	1144	Risk Ratio (M-H, Random, 95% CI)	0.35 [0.16, 0.76]
4.8.2 <2000	2	171	Risk Ratio (M-H, Random, 95% CI)	0.59 [0.24, 1.41]
4.9 Hypotension	5	283	Risk Ratio (M-H, Random, 95% CI)	1.15 [0.71, 1.84]
4.9.1 >2000	1	63	Risk Ratio (M-H, Random, 95% CI)	1.10 [0.68, 1.79]
4.9.2 <2000	4	220	Risk Ratio (M-H, Random, 95% CI)	2.73 [0.29, 25.73]
4.10 Postoperative bleeding	2	601	Mean Difference (IV, Random, 95% CI)	26.47 [-99.95, 152.89]

4.10.1 >2000	1	84	Mean Difference (IV, Random, 95% CI)	116.00 [-52.52, 284.52]
4.10.2 <2000	1	517	Mean Difference (IV, Random, 95% CI)	-20.00 [-96.82, 56.82]
4.11 PRBC transfusion	3	1138	Mean Difference (IV, Random, 95% CI)	-0.38 [-0.77, 0.02]
4.11.1 >2000	2	1053	Mean Difference (IV, Random, 95% CI)	-0.30 [-0.94, 0.34]
4.11.2 <2000	1	85	Mean Difference (IV, Random, 95% CI)	-0.42 [-0.92, 0.08]
4.12 Duration of ventilation	10	1510	Std. Mean Difference (IV, Random, 95% CI)	-0.03 [-0.27, 0.21]
4.12.1 >2000	5	1332	Std. Mean Difference (IV, Random, 95% CI)	-0.13 [-0.26, -0.01]
4.12.2 <2000	5	178	Std. Mean Difference (IV, Random, 95% CI)	0.43 [-0.35, 1.22]
4.13 ICU stay	4	1202	Mean Difference (IV, Random, 95% CI)	-1.00 [-2.51, 0.51]
4.13.1 >2000	3	1123	Mean Difference (IV, Random, 95% CI)	-0.88 [-2.15, 0.38]
4.13.2 <2000	1	79	Mean Difference (IV, Random, 95% CI)	-9.30 [-21.28, 2.68]
4.14 Hospital stay	5	1245	Mean Difference (IV, Random, 95% CI)	-0.02 [-0.69, 0.66]
4.14.1 >2000	3	1180	Mean Difference (IV, Random, 95% CI)	-0.73 [-3.21, 1.74]
4.14.2 <2000	2	65	Mean Difference (IV, Random, 95% CI)	-0.10 [-0.45, 0.25]
4.15 ICU mortality	5	1073	Risk Ratio (M-H, Random, 95% CI)	1.09 [0.92, 1.28]
4.15.1 >2000	3	1010	Risk Ratio (M-H, Random, 95% CI)	1.09 [0.92, 1.28]
4.15.2 <2000	2	63	Risk Ratio (M-H, Random, 95% CI)	1.10 [0.37, 3.24]

Forest plots of subgroup analyses

Subgroup analysis (medical vs. surgical study population)

Hospital mortality

Study or Subgroup	Higher PEEP Events	Higher PEEP Total	Lower PEEP Events	Lower PEEP Total	Weight	Risk Ratio M-H, Random, 95% CI
**7.13.1 Medical**						
Lesur 2010	12	30	16	33	5.5%	0.82 [0.47, 1.45]
Manzano 2008	19	64	16	63	5.5%	1.17 [0.66, 2.06]
Nelson 1987	5	20	6	18	1.7%	0.75 [0.28, 2.04]
Pepe 1984	13	44	18	48	5.1%	0.79 [0.44, 1.41]
Relax 2020	208	489	185	472	75.4%	1.09 [0.93, 1.26]
Weigelt 1979	16	45	17	34	6.5%	0.71 [0.42, 1.19]
**Subtotal (95% CI)**		**692**		**668**	**99.8%**	**1.02 [0.89, 1.16]**
Total events	273		258			
Heterogeneity: Tau^2 = 0.00; Chi^2 = 4.38, df = 5 (P = 0.50); I^2 = 0%						
Test for overall effect: Z = 0.30 (P = 0.77)						
**7.13.2 Surgical**						
Collier 2002	1	40	1	44	0.2%	1.10 [0.07, 17.01]
Dyhr 2002	0	7	0	8		Not estimable
Michalopoulos 1998	0	21	0	22		Not estimable
**Subtotal (95% CI)**		**68**		**74**	**0.2%**	**1.10 [0.07, 17.01]**
Total events	1		1			
Heterogeneity: Not applicable						
Test for overall effect: Z = 0.07 (P = 0.95)						
**Total (95% CI)**		**760**		**742**	**100.0%**	**1.02 [0.89, 1.16]**
Total events	274		259			
Heterogeneity: Tau^2 = 0.00; Chi^2 = 4.39, df = 6 (P = 0.62); I^2 = 0%						
Test for overall effect: Z = 0.30 (P = 0.77)						
Test for subgroup differences: Chi^2 = 0.00, df = 1 (P = 0.96), I^2 = 0%						

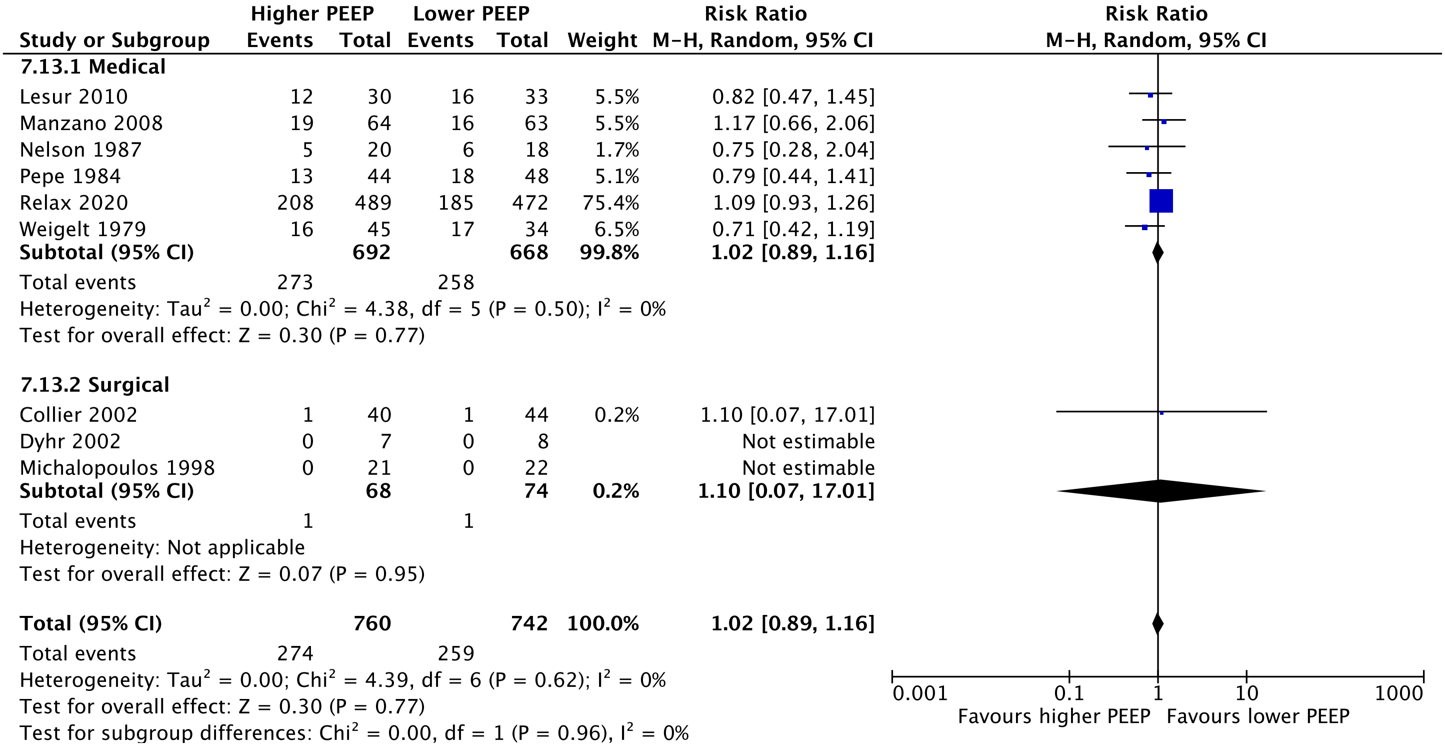

Subgroup analysis (medical vs. surgical study population)

PaO2/FiO2

Study or Subgroup	Higher PEEP Mean	Higher PEEP SD	Higher PEEP Total	Lower PEEP Mean	Lower PEEP SD	Lower PEEP Total	Weight	Mean Difference IV, Random, 95% CI
**7.1.1 Medical**								
Korovesi 2011	498	75	15	420	73	12	6.7%	78.00 [21.91, 134.09]
Koutsoukou 2006	409	65	11	437	74	10	6.0%	−28.00 [−87.83, 31.83]
Lesur 2010	293	135	30	228	67	33	7.2%	65.00 [11.56, 118.44]
Ma 2014	196	45	60	134	22	60	24.4%	62.00 [49.33, 74.67]
Manzano 2008	359	104	64	301	84	63	13.4%	58.00 [25.15, 90.85]
Relax 2020	248	112	493	190	84	476	24.5%	58.00 [45.56, 70.44]
**Subtotal (95% CI)**			**673**			**654**	**82.2%**	**56.58 [42.29, 70.87]**
Heterogeneity: $Tau^2 = 111.95$; $Chi^2 = 8.85$, df = 5 (P = 0.12); $I^2 = 44\%$								
Test for overall effect: Z = 7.76 (P < 0.00001)								
**7.1.2 Surgical**								
Holland 2007	307	82	14	337	82	14	5.9%	−30.00 [−90.75, 30.75]
Lago Borges 2013	328	85	45	270	90	44	12.0%	58.00 [21.61, 94.39]
**Subtotal (95% CI)**			**59**			**58**	**17.8%**	**17.50 [−68.47, 103.46]**
Heterogeneity: $Tau^2 = 3219.39$; $Chi^2 = 5.93$, df = 1 (P = 0.01); $I^2 = 83\%$								
Test for overall effect: Z = 0.40 (P = 0.69)								
**Total (95% CI)**			**732**			**712**	**100.0%**	**50.46 [33.93, 66.99]**
Heterogeneity: $Tau^2 = 249.71$; $Chi^2 = 16.89$, df = 7 (P = 0.02); $I^2 = 59\%$								
Test for overall effect: Z = 5.98 (P < 0.00001)								
Test for subgroup differences: $Chi^2 = 0.77$, df = 1 (P = 0.38), $I^2 = 0\%$								

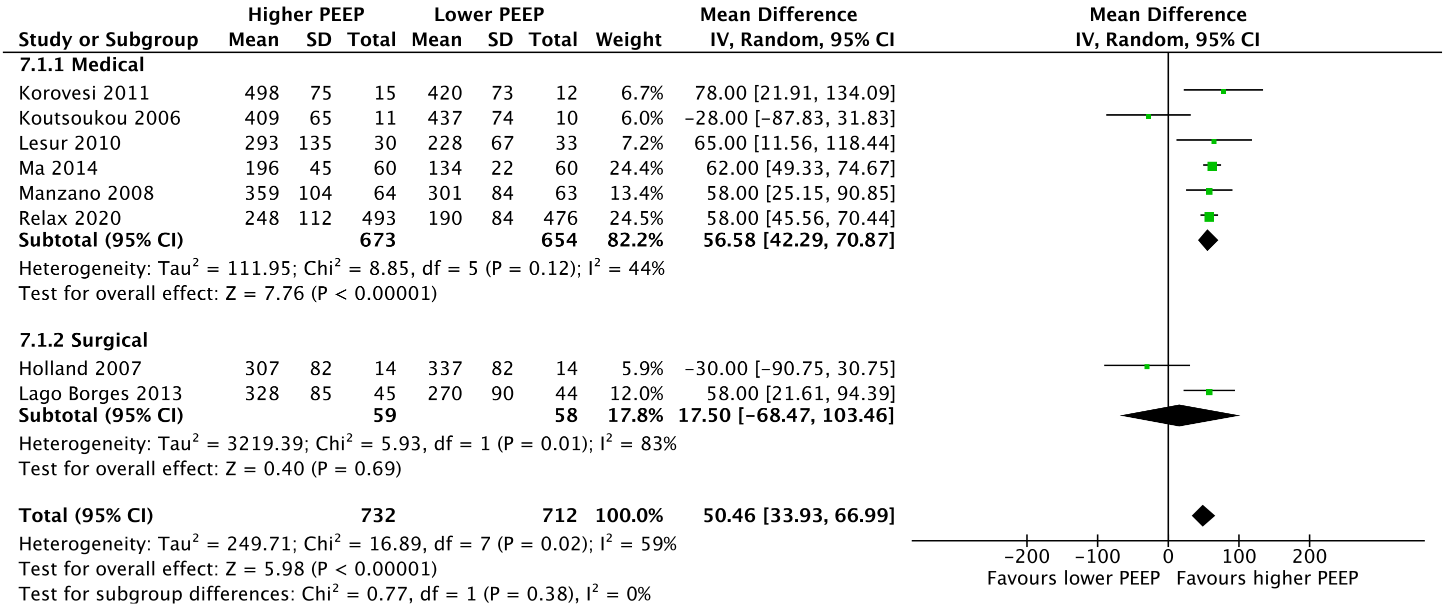

Subgroup analysis (medical vs. surgical study population)

A-aDO2

Study or Subgroup	Higher PEEP Mean	Higher PEEP SD	Higher PEEP Total	Lower PEEP Mean	Lower PEEP SD	Lower PEEP Total	Weight	Std. Mean Difference IV, Random, 95% CI
**7.2.1 Medical**								
Feeley 1975	10	22	12	102	35	13	23.6%	-3.02 [-4.22, -1.81]
Koutsoukou 2006	100	41	11	87	40	10	25.5%	0.31 [-0.55, 1.17]
**Subtotal (95% CI)**			**23**			**23**	**49.1%**	**-1.33 [-4.58, 1.93]**
Heterogeneity: $Tau^2 = 5.24$; $Chi^2 = 19.38$, df = 1 ($P < 0.0001$); $I^2 = 95\%$								
Test for overall effect: Z = 0.80 (P = 0.42)								
**7.2.2 Surgical**								
Lago Borges 2013	117	33	45	139	34	44	27.2%	-0.65 [-1.08, -0.22]
Marvel 1986	168	10	12	203	10	17	23.7%	-3.40 [-4.60, -2.20]
**Subtotal (95% CI)**			**57**			**61**	**50.9%**	**-1.97 [-4.66, 0.73]**
Heterogeneity: $Tau^2 = 3.57$; $Chi^2 = 17.99$, df = 1 ($P < 0.0001$); $I^2 = 94\%$								
Test for overall effect: Z = 1.43 (P = 0.15)								
**Total (95% CI)**			**80**			**84**	**100.0%**	**-1.62 [-3.12, -0.11]**
Heterogeneity: $Tau^2 = 2.13$; $Chi^2 = 37.49$, df = 3 ($P < 0.00001$); $I^2 = 92\%$								
Test for overall effect: Z = 2.10 (P = 0.04)								
Test for subgroup differences: $Chi^2 = 0.09$, df = 1 (P = 0.77), $I^2 = 0\%$								

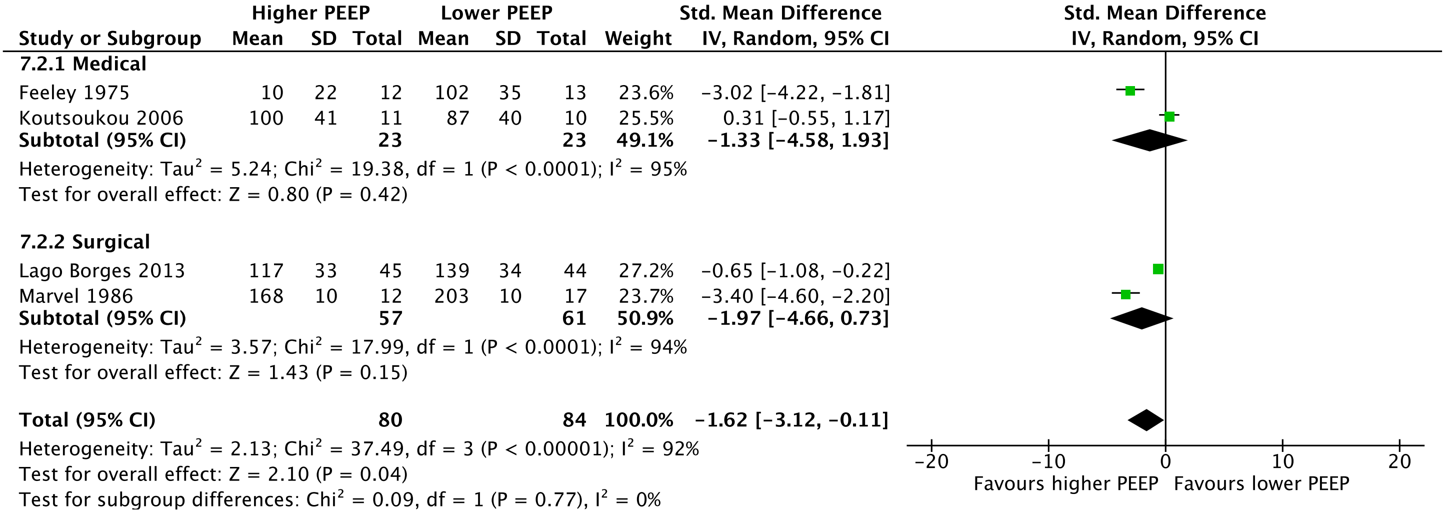

Subgroup analysis (medical vs. surgical study population)

Compliance

Study or Subgroup	Higher PEEP Mean	Higher PEEP SD	Higher PEEP Total	Lower PEEP Mean	Lower PEEP SD	Lower PEEP Total	Weight	Mean Difference IV, Random, 95% CI
**7.3.1 Medical**								
Koutsoukou 2006	62	14	11	53	11	10	24.9%	9.00 [-1.72, 19.72]
Weigelt 1979	42	36	45	39	43	34	9.0%	3.00 [-14.88, 20.88]
**Subtotal (95% CI)**			**56**			**44**	**33.9%**	**7.41 [-1.78, 16.61]**
Heterogeneity: $Tau^2 = 0.00$; $Chi^2 = 0.32$, df = 1 (P = 0.57); $I^2 = 0\%$								
Test for overall effect: Z = 1.58 (P = 0.11)								
**7.3.2 Surgical**								
Lago Borges 2013	56	19	45	47	12	44	66.1%	9.00 [2.41, 15.59]
**Subtotal (95% CI)**			**45**			**44**	**66.1%**	**9.00 [2.41, 15.59]**
Heterogeneity: Not applicable								
Test for overall effect: Z = 2.68 (P = 0.007)								
**Total (95% CI)**			**101**			**88**	**100.0%**	**8.46 [3.11, 13.82]**
Heterogeneity: $Tau^2 = 0.00$; $Chi^2 = 0.39$, df = 2 (P = 0.82); $I^2 = 0\%$								
Test for overall effect: Z = 3.10 (P = 0.002)								
Test for subgroup differences: $Chi^2 = 0.08$, df = 1 (P = 0.78), $I^2 = 0\%$								

Mean Difference
IV, Random, 95% CI

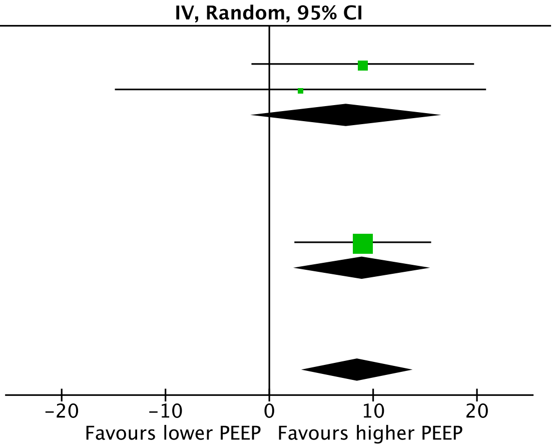

Subgroup analysis (medical vs. surgical study population)

Hypoxemia

Study or Subgroup	Higher PEEP Events	Higher PEEP Total	Lower PEEP Events	Lower PEEP Total	Weight	Risk Ratio M-H, Random, 95% CI
**7.4.1 Medical**						
Manzano 2008	12	64	34	63	24.3%	0.35 [0.20, 0.61]
Pepe 1984	1	44	4	48	3.4%	0.27 [0.03, 2.35]
Relax 2020	87	493	98	476	36.9%	0.86 [0.66, 1.11]
**Subtotal (95% CI)**		**601**		**587**	**64.6%**	**0.52 [0.24, 1.16]**
Total events	100		136			
Heterogeneity: $Tau^2 = 0.33$; $Chi^2 = 9.05$, df = 2 (P = 0.01); $I^2 = 78\%$						
Test for overall effect: Z = 1.59 (P = 0.11)						
**7.4.2 Surgical**						
Lago Borges 2013	19	45	30	44	30.9%	0.62 [0.42, 0.92]
Michalopoulos 1998	2	21	2	22	4.5%	1.05 [0.16, 6.77]
**Subtotal (95% CI)**		**66**		**66**	**35.4%**	**0.63 [0.43, 0.93]**
Total events	21		32			
Heterogeneity: $Tau^2 = 0.00$; $Chi^2 = 0.30$, df = 1 (P = 0.58); $I^2 = 0\%$						
Test for overall effect: Z = 2.30 (P = 0.02)						
**Total (95% CI)**		**667**		**653**	**100.0%**	**0.60 [0.40, 0.92]**
Total events	121		168			
Heterogeneity: $Tau^2 = 0.11$; $Chi^2 = 9.66$, df = 4 (P = 0.05); $I^2 = 59\%$						
Test for overall effect: Z = 2.37 (P = 0.02)						
Test for subgroup differences: $Chi^2 = 0.18$, df = 1 (P = 0.67), $I^2 = 0\%$						

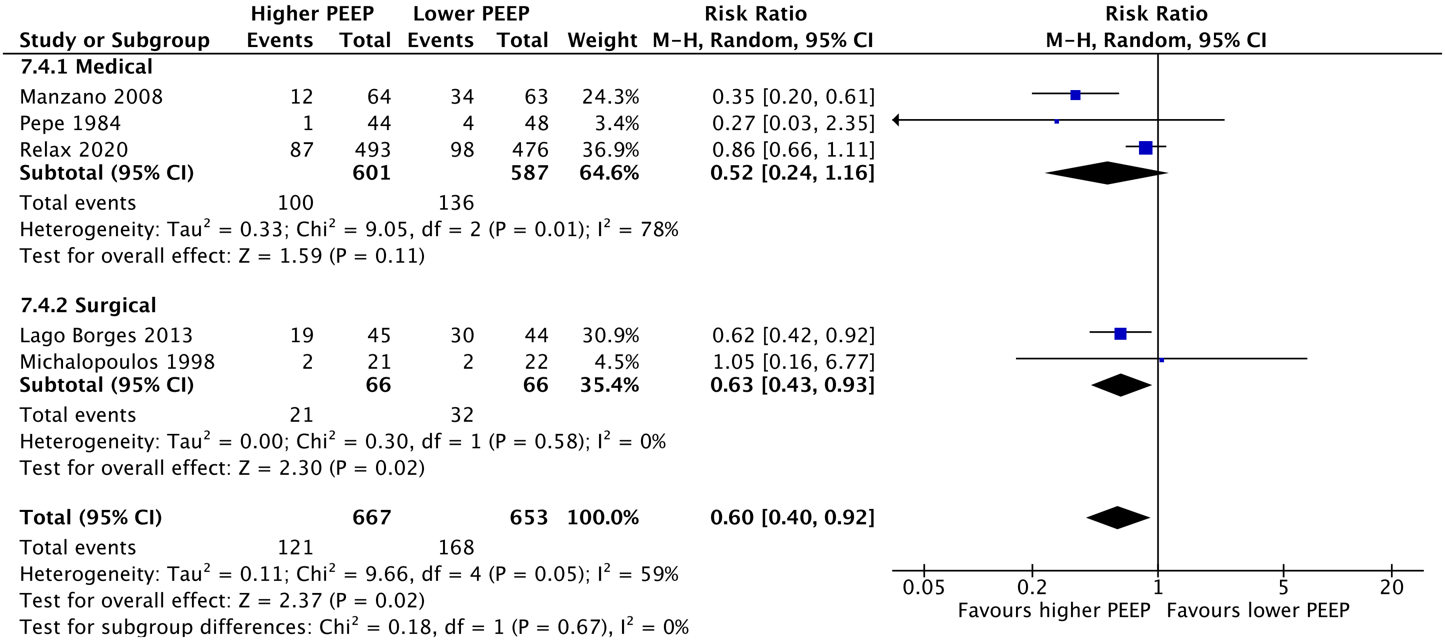

Subgroup analysis (medical vs. surgical study population)

Atelectasis

	Higher PEEP		Lower PEEP			Risk Ratio
**Study or Subgroup**	**Events**	**Total**	**Events**	**Total**	**Weight**	**M-H, Random, 95% CI**
**7.5.1 Medical**						
Manzano 2008	12	64	17	63	11.8%	0.69 [0.36, 1.33]
Pepe 1984	27	44	23	48	30.8%	1.28 [0.88, 1.87]
Relax 2020	15	493	20	476	11.6%	0.72 [0.38, 1.40]
**Subtotal (95% CI)**		**601**		**587**	**54.2%**	**0.92 [0.58, 1.46]**
Total events	54		60			
Heterogeneity: $Tau^2 = 0.09$; $Chi^2 = 4.17$, df = 2 (P = 0.12); $I^2 = 52\%$						
Test for overall effect: Z = 0.34 (P = 0.73)						
**7.5.2 Surgical**						
Good 1979	9	10	12	14	44.3%	1.05 [0.78, 1.41]
Michalopoulos 1998	2	21	2	22	1.5%	1.05 [0.16, 6.77]
**Subtotal (95% CI)**		**31**		**36**	**45.8%**	**1.05 [0.78, 1.41]**
Total events	11		14			
Heterogeneity: $Tau^2 = 0.00$; $Chi^2 = 0.00$, df = 1 (P = 1.00); $I^2 = 0\%$						
Test for overall effect: Z = 0.33 (P = 0.74)						
**Total (95% CI)**		**632**		**623**	**100.0%**	**1.02 [0.81, 1.28]**
Total events	65		74			
Heterogeneity: $Tau^2 = 0.01$; $Chi^2 = 4.51$, df = 4 (P = 0.34); $I^2 = 11\%$						
Test for overall effect: Z = 0.15 (P = 0.88)						
Test for subgroup differences: $Chi^2 = 0.22$, df = 1 (P = 0.64), $I^2 = 0\%$						

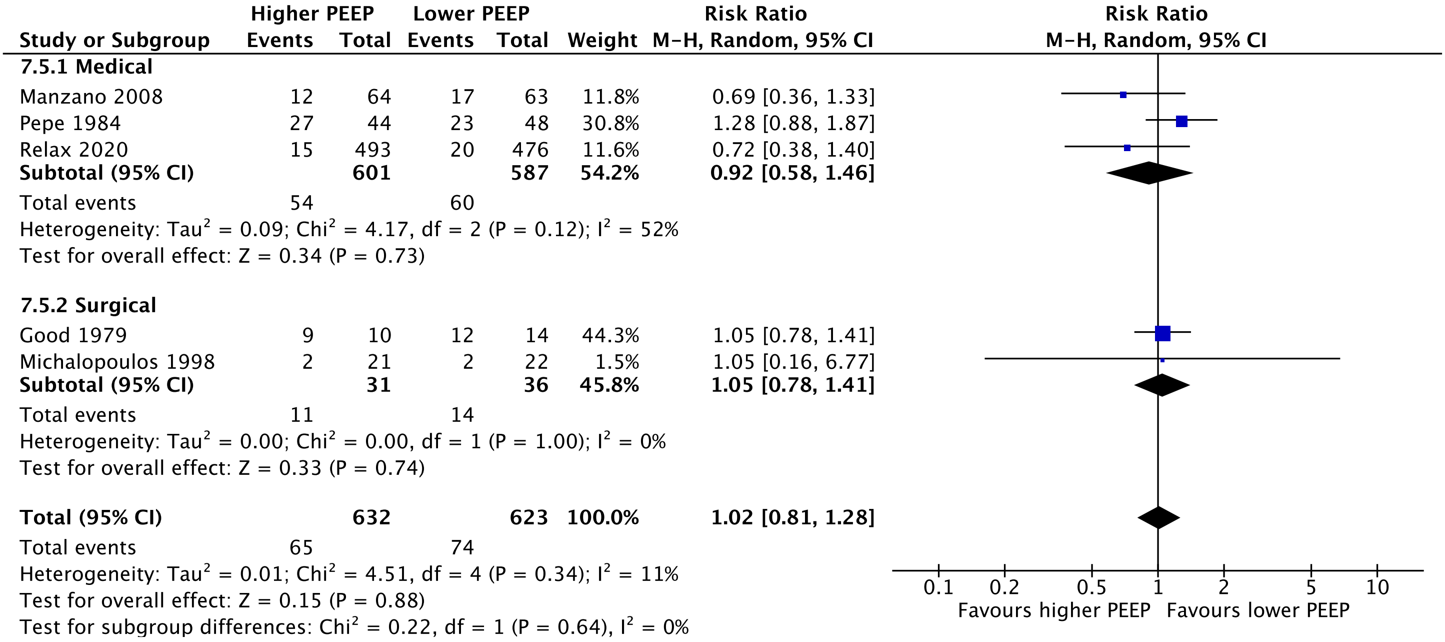

Subgroup analysis (medical vs. surgical study population)

Barotrauma

Study or Subgroup	Higher PEEP Events	Higher PEEP Total	Lower PEEP Events	Lower PEEP Total	Weight	Risk Ratio M-H, Random, 95% CI
**7.6.1 Medical**						
Manzano 2008	1	64	5	63	2.8%	0.20 [0.02, 1.64]
Nelson 1987	1	20	0	18	1.3%	2.71 [0.12, 62.70]
Pepe 1984	19	44	24	48	63.5%	0.86 [0.56, 1.34]
Relax 2020	12	493	19	476	24.4%	0.61 [0.30, 1.24]
Weigelt 1979	5	45	4	34	8.1%	0.94 [0.27, 3.25]
**Subtotal (95% CI)**		**666**		**639**	**100.0%**	**0.78 [0.55, 1.11]**
Total events	38		52			
Heterogeneity: $Tau^2 = 0.00$; $Chi^2 = 3.10$, df = 4 (P = 0.54); $I^2 = 0\%$						
Test for overall effect: Z = 1.40 (P = 0.16)						
**7.6.2 Surgical**						
Good 1979	0	10	0	14		Not estimable
Michalopoulos 1998	0	21	0	22		Not estimable
**Subtotal (95% CI)**		**31**		**36**		**Not estimable**
Total events	0		0			
Heterogeneity: Not applicable						
Test for overall effect: Not applicable						
**Total (95% CI)**		**697**		**675**	**100.0%**	**0.78 [0.55, 1.11]**
Total events	38		52			
Heterogeneity: $Tau^2 = 0.00$; $Chi^2 = 3.10$, df = 4 (P = 0.54); $I^2 = 0\%$						
Test for overall effect: Z = 1.40 (P = 0.16)						
Test for subgroup differences: Not applicable						

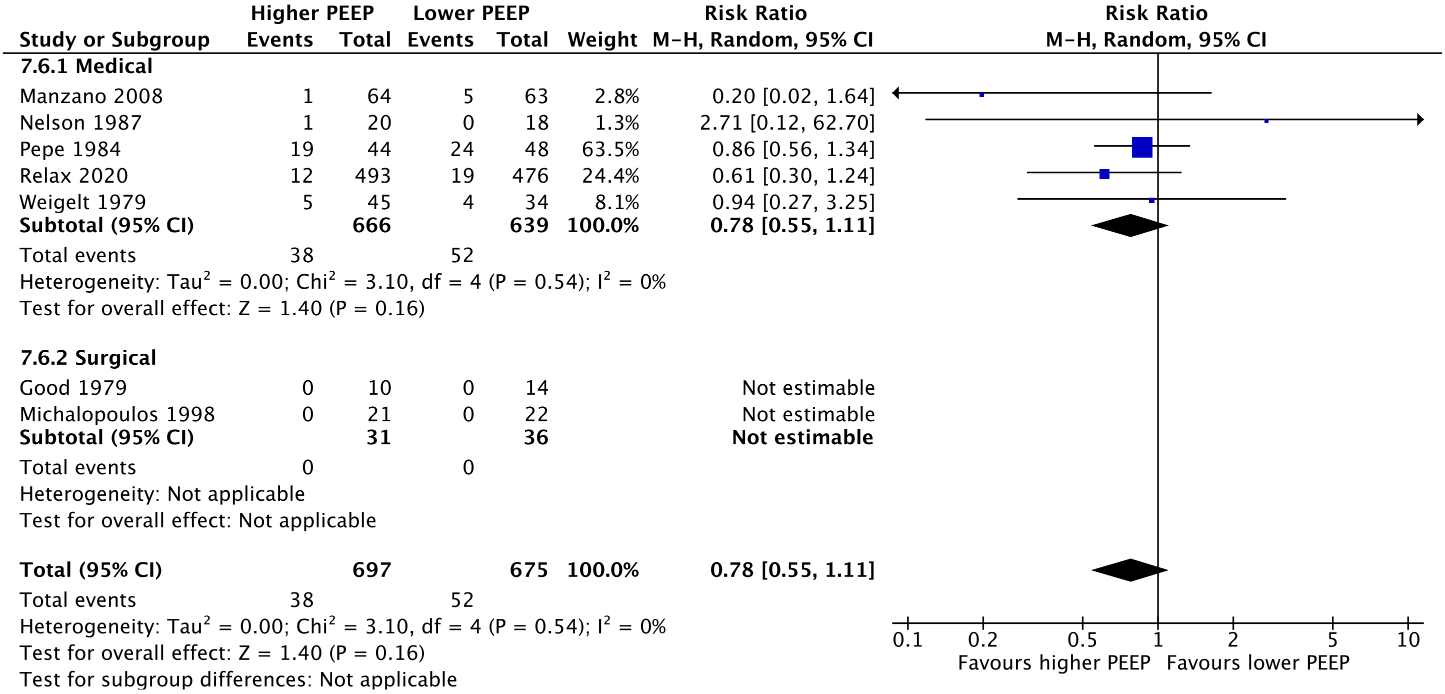

Subgroup analysis (medical vs. surgical study population)

Hypotension

Study or Subgroup	Higher PEEP Events	Higher PEEP Total	Lower PEEP Events	Lower PEEP Total	Weight	Risk Ratio M-H, Random, 95% CI
**7.7.1 Medical**						
Feeley 1975	0	12	0	13		Not estimable
Lesur 2010	16	30	16	33	95.5%	1.10 [0.68, 1.79]
Pepe 1984	1	44	0	48	2.2%	3.27 [0.14, 78.15]
Weigelt 1979	1	45	0	34	2.2%	2.28 [0.10, 54.36]
**Subtotal (95% CI)**		**131**		**128**	**100.0%**	**1.15 [0.71, 1.84]**
Total events	18		16			
Heterogeneity: $Tau^2 = 0.00; Chi^2 = 0.67, df = 2 (P = 0.72); I^2 = 0\%$						
Test for overall effect: Z = 0.56 (P = 0.57)						
**7.7.2 Surgical**						
Good 1979	0	10	0	14		Not estimable
**Subtotal (95% CI)**		**10**		**14**		**Not estimable**
Total events	0		0			
Heterogeneity: Not applicable						
Test for overall effect: Not applicable						
**Total (95% CI)**		**141**		**142**	**100.0%**	**1.15 [0.71, 1.84]**
Total events	18		16			
Heterogeneity: $Tau^2 = 0.00; Chi^2 = 0.67, df = 2 (P = 0.72); I^2 = 0\%$						
Test for overall effect: Z = 0.56 (P = 0.57)						
Test for subgroup differences: Not applicable						

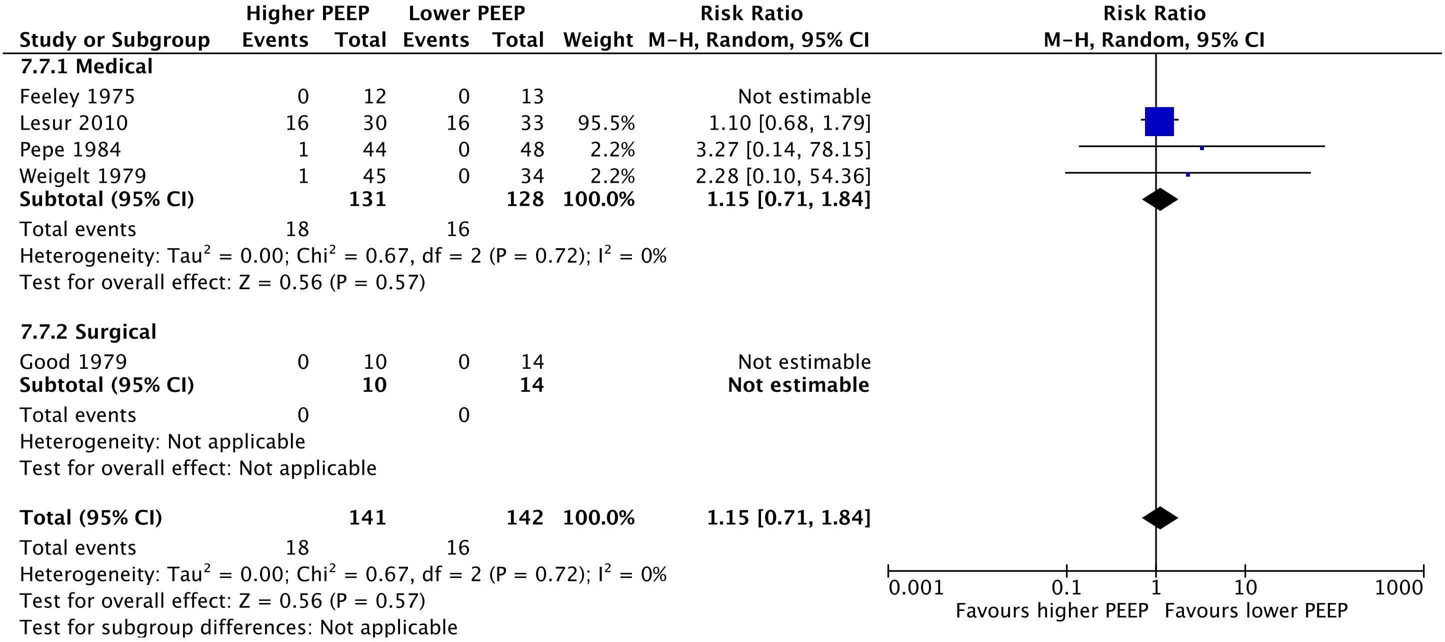

Subgroup analysis (medical vs. surgical study population)

Central venous pressure

Study or Subgroup	Higher PEEP Mean	Higher PEEP SD	Higher PEEP Total	Lower PEEP Mean	Lower PEEP SD	Lower PEEP Total	Weight	Mean Difference IV, Random, 95% CI
**7.8.1 Medical**								
Lesur 2010	12.5	1.5	30	10.8	2.9	33	78.7%	1.70 [0.57, 2.83]
**Subtotal (95% CI)**			**30**			**33**	**78.7%**	**1.70 [0.57, 2.83]**
Heterogeneity: Not applicable								
Test for overall effect: Z = 2.96 (P = 0.003)								
**7.8.2 Surgical**								
Dyhr 2002	13	11	7	9.7	7.1	8	1.1%	3.30 [−6.22, 12.82]
Holland 2007	9	3	14	9	3	14	20.2%	0.00 [−2.22, 2.22]
**Subtotal (95% CI)**			**21**			**22**	**21.3%**	**0.17 [−1.99, 2.33]**
Heterogeneity: $Tau^2 = 0.00$; $Chi^2 = 0.44$, df = 1 (P = 0.51); $I^2 = 0\%$								
Test for overall effect: Z = 0.15 (P = 0.88)								
**Total (95% CI)**			**51**			**55**	**100.0%**	**1.37 [0.38, 2.37]**
Heterogeneity: $Tau^2 = 0.00$; $Chi^2 = 1.95$, df = 2 (P = 0.38); $I^2 = 0\%$								
Test for overall effect: Z = 2.70 (P = 0.007)								
Test for subgroup differences: $Chi^2 = 1.51$, df = 1 (P = 0.22), $I^2 = 33.8\%$								

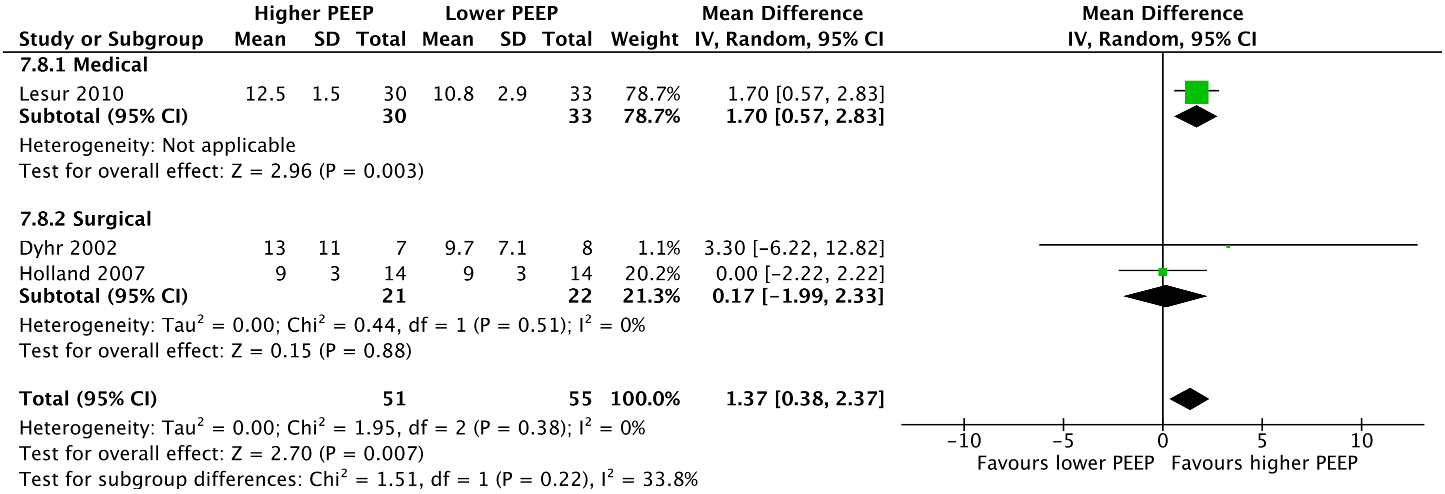

Subgroup analysis (medical vs. surgical study population)

Packed red blood cell transfusion

	Higher PEEP			Lower PEEP				Mean Difference
Study or Subgroup	Mean	SD	Total	Mean	SD	Total	Weight	IV, Random, 95% CI
**7.9.1 Medical**								
Relax 2020	1.7	0.7	493	1	0	476		Not estimable
**Subtotal (95% CI)**			**493**			**476**		**Not estimable**
Heterogeneity: Not applicable								
Test for overall effect: Not applicable								
**7.9.2 Surgical**								
Collier 2002	0.8	1.4	40	1.1	1.6	44	37.5%	−0.30 [−0.94, 0.34]
Zurich 1982	0.33	0.87	41	0.75	1.42	44	62.5%	−0.42 [−0.92, 0.08]
**Subtotal (95% CI)**			**81**			**88**	**100.0%**	**−0.38 [−0.77, 0.02]**
Heterogeneity: Tau² = 0.00; Chi² = 0.08, df = 1 (P = 0.77); I² = 0%								
Test for overall effect: Z = 1.87 (P = 0.06)								
**Total (95% CI)**			**574**			**564**	**100.0%**	**−0.38 [−0.77, 0.02]**
Heterogeneity: Tau² = 0.00; Chi² = 0.08, df = 1 (P = 0.77); I² = 0%								
Test for overall effect: Z = 1.87 (P = 0.06)								
Test for subgroup differences: Not applicable								

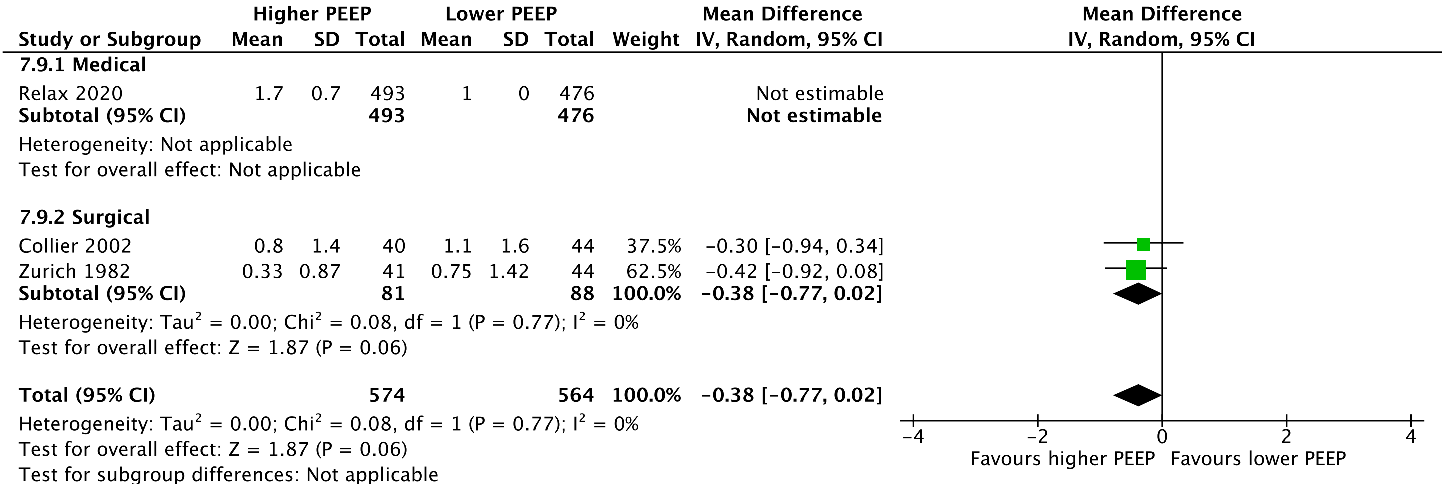

Subgroup analysis (medical vs. surgical study population)

Duration of ventilation

	Higher PEEP			Lower PEEP				Std. Mean Difference
**Study or Subgroup**	**Mean**	**SD**	**Total**	**Mean**	**SD**	**Total**	**Weight**	**IV, Random, 95% CI**
**7.10.1 Medical**								
Feeley 1975	258	217	12	259	149	13	6.4%	−0.01 [−0.79, 0.78]
Lesur 2010	9.2	8.8	30	9.2	8.5	33	10.9%	0.00 [−0.49, 0.49]
Manzano 2008	5.8	6.8	64	6.5	6.2	63	14.2%	−0.11 [−0.45, 0.24]
Nelson 1987	5.3	5	20	3.4	3	18	8.2%	0.45 [−0.20, 1.09]
Relax 2020	4.8	6.6	493	5.5	7.4	476	19.2%	−0.10 [−0.23, 0.03]
Weigelt 1979	9.3	13	45	14	21.7	34	11.9%	−0.27 [−0.72, 0.18]
**Subtotal (95% CI)**			**664**			**637**	**70.8%**	**−0.09 [−0.20, 0.02]**
Heterogeneity: $Tau^2 = 0.00$; $Chi^2 = 3.46$, df = 5 (P = 0.63); $I^2 = 0\%$								
Test for overall effect: Z = 1.59 (P = 0.11)								
**7.10.2 Surgical**								
Collier 2002	409.1	208.6	40	439.3	277.7	44	12.3%	−0.12 [−0.55, 0.31]
Good 1979	15.7	0.4	10	14.8	0.5	14	4.5%	1.88 [0.88, 2.88]
Lago Borges 2014	5.1	2.9	45	6.8	3.2	44	12.4%	−0.55 [−0.98, −0.13]
Marvel 1986	9.3	0.6	12	0	0	0		Not estimable
**Subtotal (95% CI)**			**107**			**102**	**29.2%**	**0.28 [−0.71, 1.27]**
Heterogeneity: $Tau^2 = 0.66$; $Chi^2 = 19.38$, df = 2 (P < 0.0001); $I^2 = 90\%$								
Test for overall effect: Z = 0.55 (P = 0.58)								
**Total (95% CI)**			**771**			**739**	**100.0%**	**−0.03 [−0.27, 0.21]**
Heterogeneity: $Tau^2 = 0.07$; $Chi^2 = 23.01$, df = 8 (P = 0.003); $I^2 = 65\%$								
Test for overall effect: Z = 0.24 (P = 0.81)								
Test for subgroup differences: $Chi^2 = 0.52$, df = 1 (P = 0.47), $I^2 = 0\%$								

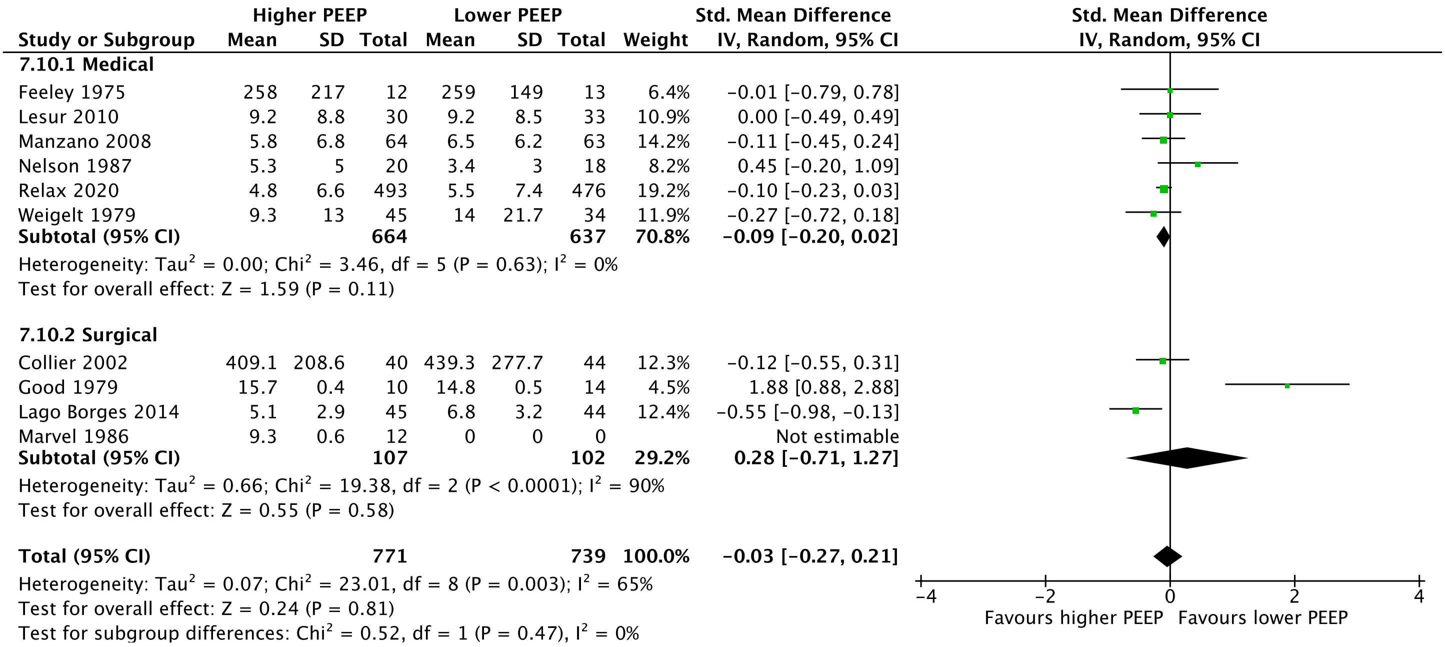

Subgroup analysis (medical vs. surgical study population)

Hospital stay

Study or Subgroup	Higher PEEP Mean	Higher PEEP SD	Higher PEEP Total	Lower PEEP Mean	Lower PEEP SD	Lower PEEP Total	Weight	Mean Difference IV, Random, 95% CI
**7.11.1 Medical**								
Manzano 2008	19.5	18.2	64	26.3	22	63	0.9%	−6.80 [−13.83, 0.23]
Nelson 1987	28	24	20	26	24	18	0.2%	2.00 [−13.28, 17.28]
Relax 2020	19	21.4	493	19.9	22.1	476	5.6%	−0.90 [−3.64, 1.84]
**Subtotal (95% CI)**			**577**			**557**	**6.7%**	**−2.11 [−5.95, 1.72]**
Heterogeneity: Tau^2 = 3.47; Chi^2 = 2.57, df = 2 (P = 0.28); I^2 = 22%								
Test for overall effect: Z = 1.08 (P = 0.28)								
**7.11.2 Surgical**								
Collier 2002	5.7	2.5	40	5.2	1.7	44	31.1%	0.50 [−0.42, 1.42]
Marvel 1986	8.8	0.5	12	8.9	0.4	15	62.2%	−0.10 [−0.45, 0.25]
**Subtotal (95% CI)**			**52**			**59**	**93.3%**	**0.04 [−0.46, 0.54]**
Heterogeneity: Tau^2 = 0.05; Chi^2 = 1.42, df = 1 (P = 0.23); I^2 = 30%								
Test for overall effect: Z = 0.16 (P = 0.87)								
**Total (95% CI)**			**629**			**616**	**100.0%**	**−0.02 [−0.69, 0.66]**
Heterogeneity: Tau^2 = 0.16; Chi^2 = 5.42, df = 4 (P = 0.25); I^2 = 26%								
Test for overall effect: Z = 0.04 (P = 0.96)								
Test for subgroup differences: Chi^2 = 1.19, df = 1 (P = 0.28), I^2 = 15.9%								

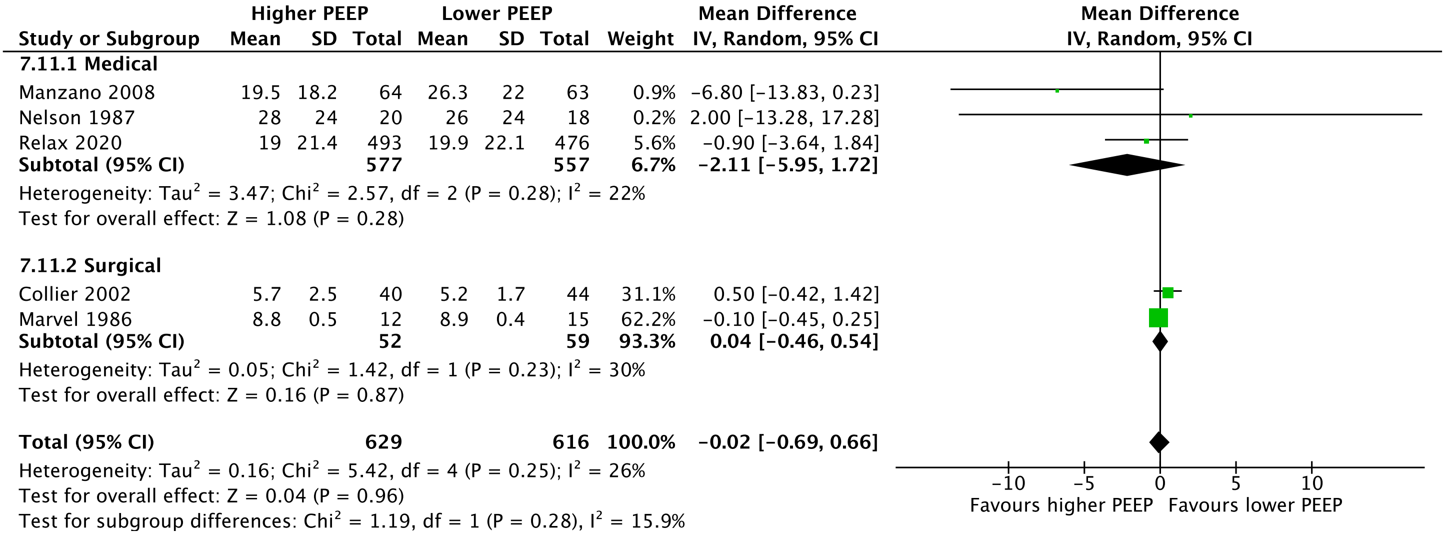

Subgroup analysis (medical vs. surgical study population)

ICU mortality

Study or Subgroup	Higher PEEP Events	Higher PEEP Total	Lower PEEP Events	Lower PEEP Total	Weight	Risk Ratio M-H, Random, 95% CI
**7.12.1 Medical**						
Feeley 1975	2	12	1	13	0.5%	2.17 [0.22, 20.94]
Korovesi 2011	3	15	4	12	1.6%	0.60 [0.17, 2.18]
Nelson 1987	4	20	4	18	1.8%	0.90 [0.26, 3.08]
Relax 2020	185	492	163	476	96.0%	1.10 [0.93, 1.30]
**Subtotal (95% CI)**		**539**		**519**	**100.0%**	**1.09 [0.92, 1.28]**
Total events	194		172			
Heterogeneity: Tau² = 0.00; Chi² = 1.27, df = 3 (P = 0.74); I² = 0%						
Test for overall effect: Z = 0.99 (P = 0.32)						
**7.12.2 Surgical**						
Dyhr 2002	0	7	0	8		Not estimable
**Subtotal (95% CI)**		**7**		**8**		**Not estimable**
Total events	0		0			
Heterogeneity: Not applicable						
Test for overall effect: Not applicable						
**Total (95% CI)**		**546**		**527**	**100.0%**	**1.09 [0.92, 1.28]**
Total events	194		172			
Heterogeneity: Tau² = 0.00; Chi² = 1.27, df = 3 (P = 0.74); I² = 0%						
Test for overall effect: Z = 0.99 (P = 0.32)						
Test for subgroup differences: Not applicable						

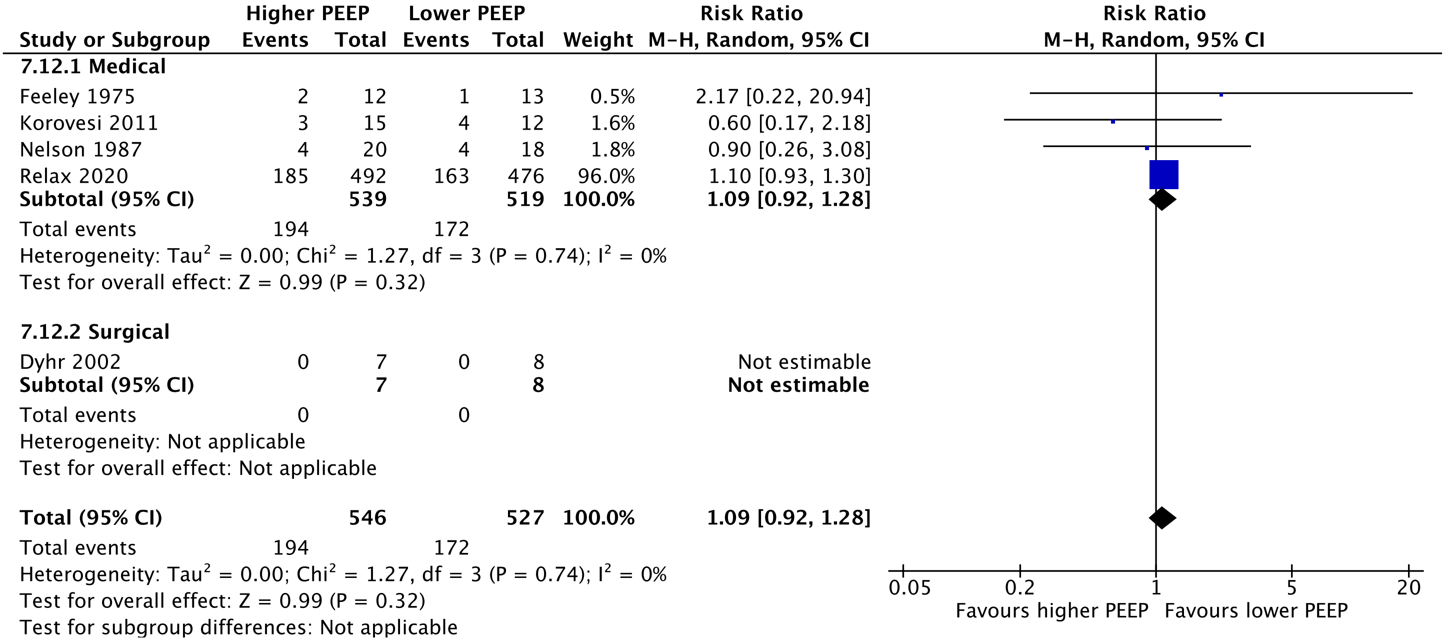

Subgroup analysis (ZEEP vs. lower PEEP)

Hospital mortality

Study or Subgroup	Higher PEEP Events	Higher PEEP Total	Lower PEEP Events	Lower PEEP Total	Weight	Risk Ratio M-H, Random, 95% CI
**8.17.1 ZEEP**						
Dyhr 2002	0	7	0	8		Not estimable
Lesur 2010	12	30	16	33	5.5%	0.82 [0.47, 1.45]
Manzano 2008	19	64	16	63	5.5%	1.17 [0.66, 2.06]
Michalopoulos 1998	0	21	0	22		Not estimable
Pepe 1984	13	44	18	48	5.1%	0.79 [0.44, 1.41]
Weigelt 1979	16	45	17	34	6.5%	0.71 [0.42, 1.19]
**Subtotal (95% CI)**		**211**		**208**	**22.7%**	**0.85 [0.64, 1.12]**
Total events	60		67			
Heterogeneity: $Tau^2 = 0.00$; $Chi^2 = 1.76$, df = 3 (P = 0.62); $I^2 = 0\%$						
Test for overall effect: Z = 1.14 (P = 0.25)						
**8.17.2 Lower PEEP**						
Collier 2002	1	40	1	44	0.2%	1.10 [0.07, 17.01]
Nelson 1987	5	20	6	18	1.7%	0.75 [0.28, 2.04]
Relax 2020	208	489	185	472	75.4%	1.09 [0.93, 1.26]
**Subtotal (95% CI)**		**549**		**534**	**77.3%**	**1.08 [0.93, 1.25]**
Total events	214		192			
Heterogeneity: $Tau^2 = 0.00$; $Chi^2 = 0.51$, df = 2 (P = 0.77); $I^2 = 0\%$						
Test for overall effect: Z = 0.96 (P = 0.34)						
**Total (95% CI)**		**760**		**742**	**100.0%**	**1.02 [0.89, 1.16]**
Total events	274		259			
Heterogeneity: $Tau^2 = 0.00$; $Chi^2 = 4.39$, df = 6 (P = 0.62); $I^2 = 0\%$						
Test for overall effect: Z = 0.30 (P = 0.77)						
Test for subgroup differences: $Chi^2 = 2.13$, df = 1 (P = 0.14), $I^2 = 53.0\%$						

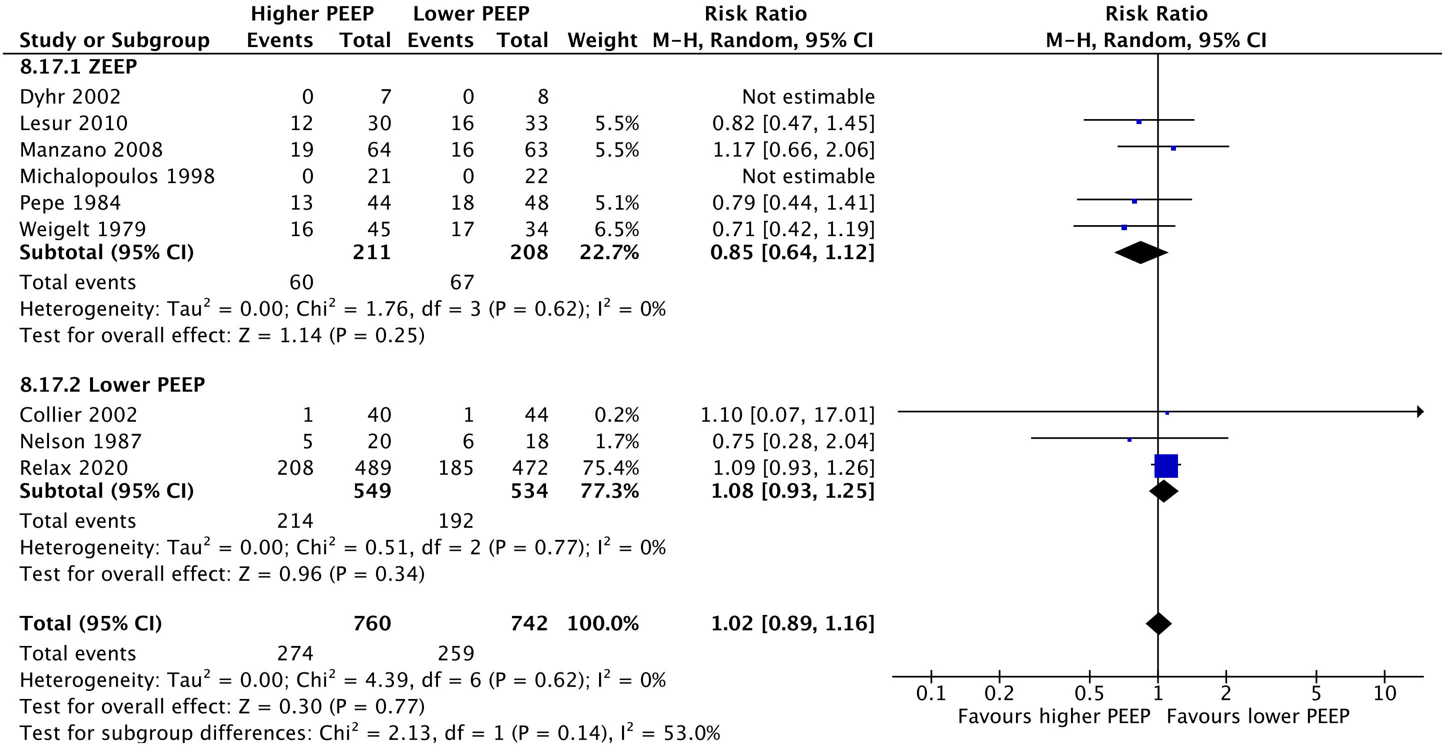

Subgroup analysis (ZEEP vs. lower PEEP)

PaO2/FiO2

Study or Subgroup	Higher PEEP Mean	Higher PEEP SD	Higher PEEP Total	Lower PEEP Mean	Lower PEEP SD	Lower PEEP Total	Weight	Mean Difference IV, Random, 95% CI
**8.1.1 ZEEP**								
Korovesi 2011	498	75	15	420	73	12	6.7%	78.00 [21.91, 134.09]
Koutsoukou 2006	409	65	11	437	74	10	6.0%	−28.00 [−87.83, 31.83]
Lesur 2010	293	135	30	228	67	33	7.2%	65.00 [11.56, 118.44]
Manzano 2008	359	104	64	301	84	63	13.4%	58.00 [25.15, 90.85]
**Subtotal (95% CI)**			**120**			**118**	**33.2%**	**45.75 [5.42, 86.09]**
Heterogeneity: $Tau^2 = 1040.99$; $Chi^2 = 8.02$, df = 3 (P = 0.05); $I^2 = 63\%$								
Test for overall effect: Z = 2.22 (P = 0.03)								
**8.1.2 Lower PEEP**								
Holland 2007	307	82	14	337	82	14	5.9%	−30.00 [−90.75, 30.75]
Lago Borges 2013	328	85	45	270	90	44	12.0%	58.00 [21.61, 94.39]
Ma 2014	196	45	60	134	22	60	24.4%	62.00 [49.33, 74.67]
Relax 2020	248	112	493	190	84	476	24.5%	58.00 [45.56, 70.44]
**Subtotal (95% CI)**			**612**			**594**	**66.8%**	**52.97 [34.89, 71.05]**
Heterogeneity: $Tau^2 = 181.76$; $Chi^2 = 8.44$, df = 3 (P = 0.04); $I^2 = 64\%$								
Test for overall effect: Z = 5.74 (P < 0.00001)								
**Total (95% CI)**			**732**			**712**	**100.0%**	**50.46 [33.93, 66.99]**
Heterogeneity: $Tau^2 = 249.71$; $Chi^2 = 16.89$, df = 7 (P = 0.02); $I^2 = 59\%$								
Test for overall effect: Z = 5.98 (P < 0.00001)								
Test for subgroup differences: $Chi^2 = 0.10$, df = 1 (P = 0.75), $I^2 = 0\%$								

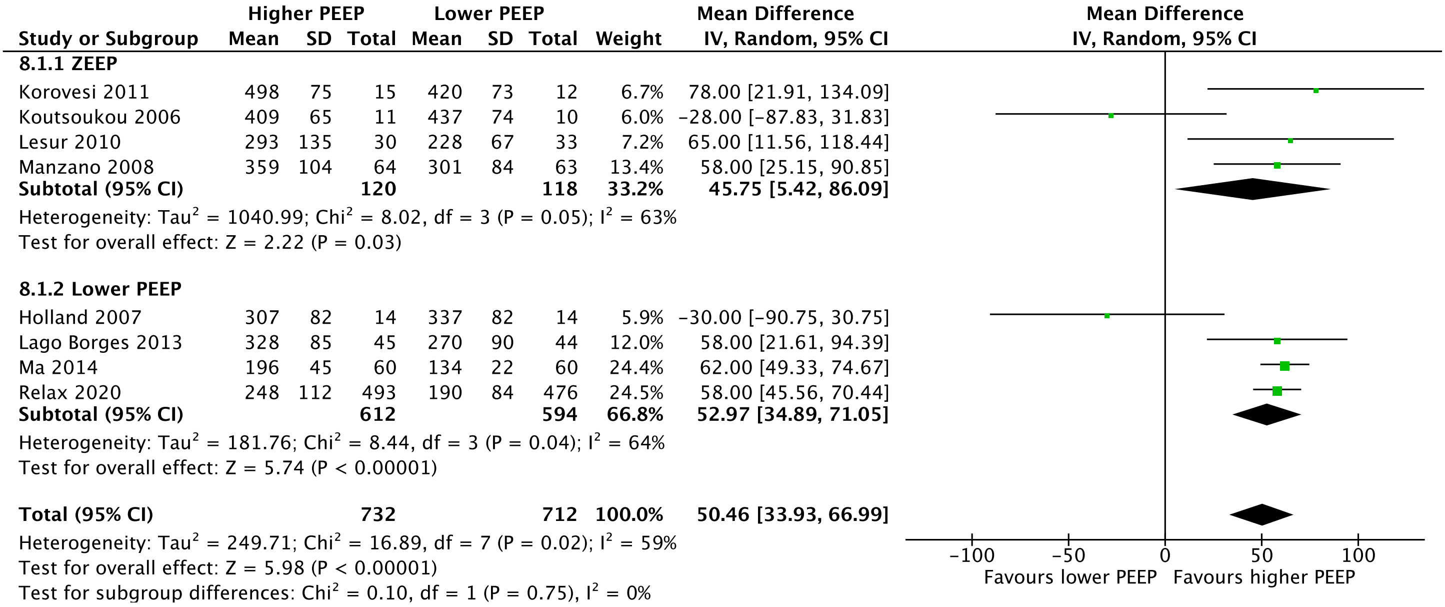

Subgroup analysis (ZEEP vs. lower PEEP)

A-aDO2

Study or Subgroup	Higher PEEP Mean	Higher PEEP SD	Higher PEEP Total	Lower PEEP Mean	Lower PEEP SD	Lower PEEP Total	Weight	Std. Mean Difference IV, Random, 95% CI
**8.2.1 ZEEP**								
Feeley 1975	10	22	12	102	35	13	23.6%	−3.02 [−4.22, −1.81]
Koutsoukou 2006	100	41	11	87	40	10	25.5%	0.31 [−0.55, 1.17]
**Subtotal (95% CI)**			**23**			**23**	**49.1%**	**−1.33 [−4.58, 1.93]**
Heterogeneity: $Tau^2 = 5.24$; $Chi^2 = 19.38$, df = 1 ($P < 0.0001$); $I^2 = 95\%$								
Test for overall effect: Z = 0.80 (P = 0.42)								
**8.2.2 Lower PEEP**								
Lago Borges 2013	117	33	45	139	34	44	27.2%	−0.65 [−1.08, −0.22]
Marvel 1986	168	10	12	203	10	17	23.7%	−3.40 [−4.60, −2.20]
**Subtotal (95% CI)**			**57**			**61**	**50.9%**	**−1.97 [−4.66, 0.73]**
Heterogeneity: $Tau^2 = 3.57$; $Chi^2 = 17.99$, df = 1 ($P < 0.0001$); $I^2 = 94\%$								
Test for overall effect: Z = 1.43 (P = 0.15)								
**Total (95% CI)**			**80**			**84**	**100.0%**	**−1.62 [−3.12, −0.11]**
Heterogeneity: $Tau^2 = 2.13$; $Chi^2 = 37.49$, df = 3 ($P < 0.00001$); $I^2 = 92\%$								
Test for overall effect: Z = 2.10 (P = 0.04)								
Test for subgroup differences: $Chi^2 = 0.09$, df = 1 ($P = 0.77$), $I^2 = 0\%$								

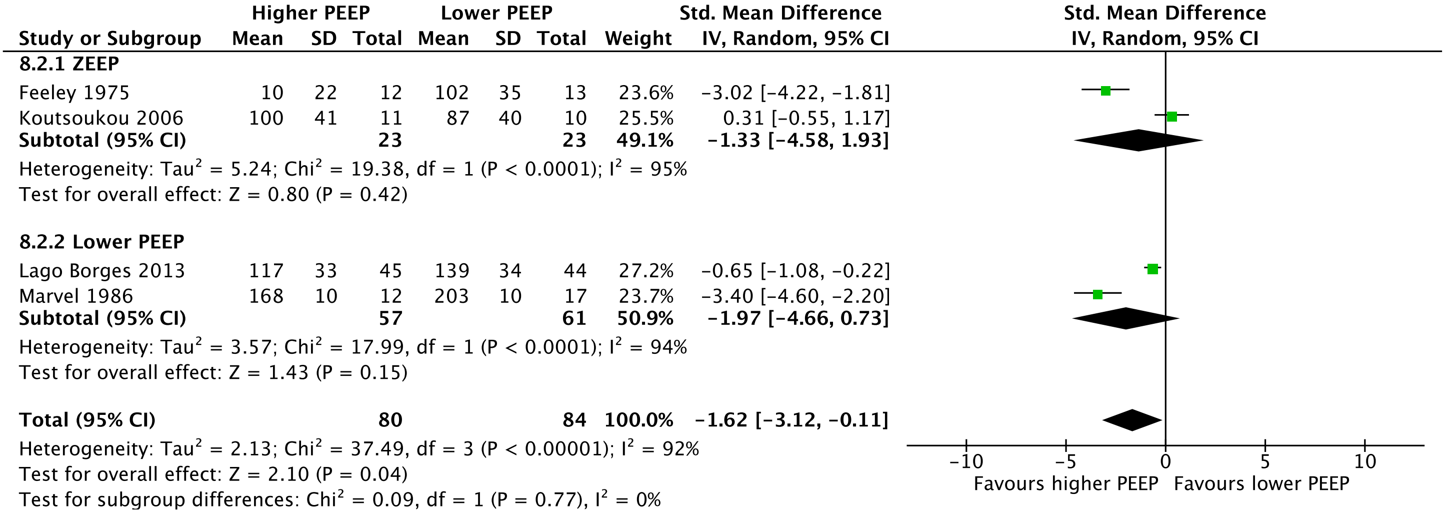

Subgroup analysis (ZEEP vs. lower PEEP)

Compliance

Study or Subgroup	Higher PEEP Mean	SD	Total	Lower PEEP Mean	SD	Total	Weight	Mean Difference IV, Random, 95% CI
**8.3.1 ZEEP**								
Koutsoukou 2006	62	14	11	53	11	10	24.9%	9.00 [−1.72, 19.72]
Weigelt 1979	42	36	45	39	43	34	9.0%	3.00 [−14.88, 20.88]
**Subtotal (95% CI)**			**56**			**44**	**33.9%**	**7.41 [−1.78, 16.61]**
Heterogeneity: Tau² = 0.00; Chi² = 0.32, df = 1 (P = 0.57); I² = 0%								
Test for overall effect: Z = 1.58 (P = 0.11)								
**8.3.2 Lower PEEP**								
Lago Borges 2013	56	19	45	47	12	44	66.1%	9.00 [2.41, 15.59]
**Subtotal (95% CI)**			**45**			**44**	**66.1%**	**9.00 [2.41, 15.59]**
Heterogeneity: Not applicable								
Test for overall effect: Z = 2.68 (P = 0.007)								
**Total (95% CI)**			**101**			**88**	**100.0%**	**8.46 [3.11, 13.82]**

Heterogeneity: $Tau^2 = 0.00$; $Chi^2 = 0.39$, df = 2 (P = 0.82); $I^2 = 0\%$
Test for overall effect: Z = 3.10 (P = 0.002)
Test for subgroup differences: $Chi^2 = 0.08$, df = 1 (P = 0.78), $I^2 = 0\%$

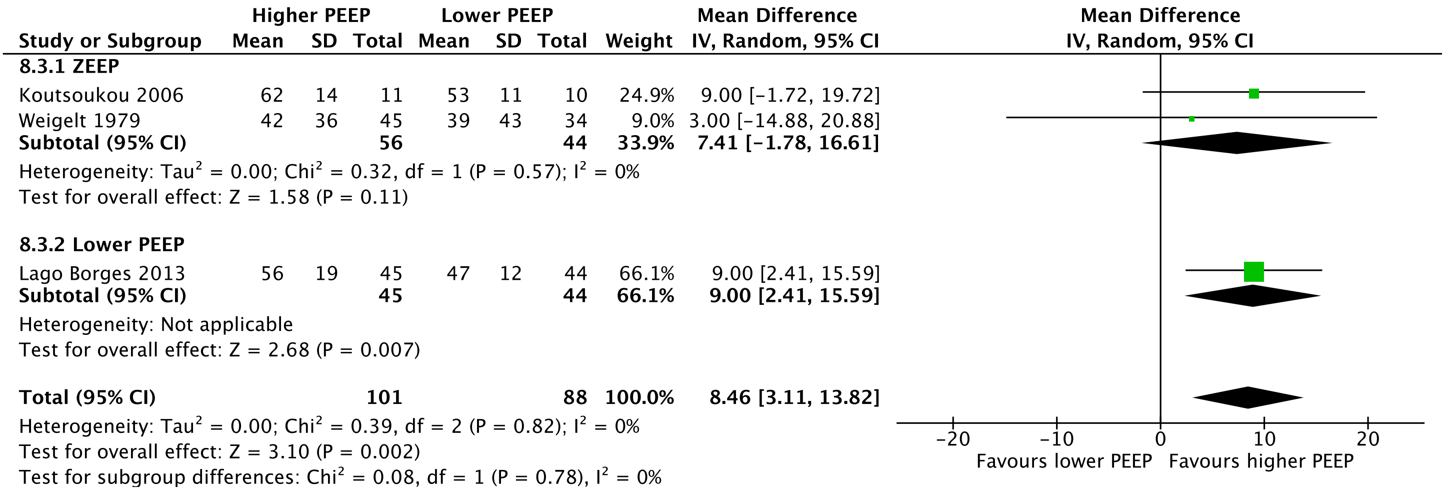

Subgroup analysis (ZEEP vs. lower PEEP)

Hypoxemia

Study or Subgroup	Higher PEEP Events	Higher PEEP Total	Lower PEEP Events	Lower PEEP Total	Weight	Risk Ratio M-H, Random, 95% CI
**8.4.1 ZEEP**						
Manzano 2008	12	64	34	63	24.3%	0.35 [0.20, 0.61]
Michalopoulos 1998	2	21	2	22	4.5%	1.05 [0.16, 6.77]
Pepe 1984	1	44	4	48	3.4%	0.27 [0.03, 2.35]
**Subtotal (95% CI)**		**129**		**133**	**32.2%**	**0.37 [0.22, 0.63]**
Total events	15		40			
Heterogeneity: $Tau^2 = 0.00$; $Chi^2 = 1.32$, df = 2 (P = 0.52); $I^2 = 0\%$						
Test for overall effect: Z = 3.72 (P = 0.0002)						
**8.4.2 Lower PEEP**						
Lago Borges 2013	19	45	30	44	30.9%	0.62 [0.42, 0.92]
Relax 2020	87	493	98	476	36.9%	0.86 [0.66, 1.11]
**Subtotal (95% CI)**		**538**		**520**	**67.8%**	**0.75 [0.55, 1.04]**
Total events	106		128			
Heterogeneity: $Tau^2 = 0.03$; $Chi^2 = 1.88$, df = 1 (P = 0.17); $I^2 = 47\%$						
Test for overall effect: Z = 1.74 (P = 0.08)						
**Total (95% CI)**		**667**		**653**	**100.0%**	**0.60 [0.40, 0.92]**
Total events	121		168			
Heterogeneity: $Tau^2 = 0.11$; $Chi^2 = 9.66$, df = 4 (P = 0.05); $I^2 = 59\%$						
Test for overall effect: Z = 2.37 (P = 0.02)						
Test for subgroup differences: $Chi^2 = 5.13$, df = 1 (P = 0.02), $I^2 = 80.5\%$						

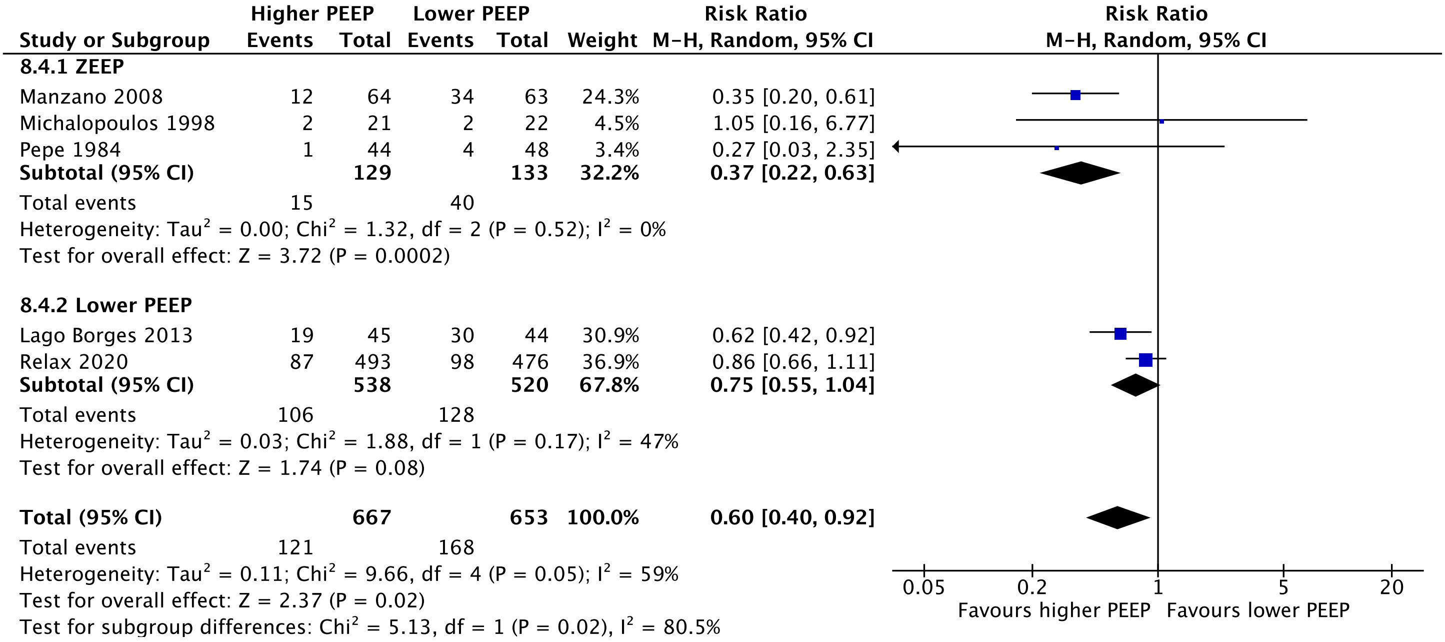

Subgroup analysis (ZEEP vs. lower PEEP)

Atelectasis

Study or Subgroup	Higher PEEP Events	Higher PEEP Total	Lower PEEP Events	Lower PEEP Total	Weight	Risk Ratio M-H, Random, 95% CI
**8.5.1 ZEEP**						
Good 1979	9	10	12	14	44.3%	1.05 [0.78, 1.41]
Manzano 2008	12	64	17	63	11.8%	0.69 [0.36, 1.33]
Michalopoulos 1998	2	21	2	22	1.5%	1.05 [0.16, 6.77]
Pepe 1984	27	44	23	48	30.8%	1.28 [0.88, 1.87]
**Subtotal (95% CI)**		**139**		**147**	**88.4%**	**1.07 [0.86, 1.33]**
Total events	50		54			
Heterogeneity: Tau² = 0.00; Chi² = 2.70, df = 3 (P = 0.44); I² = 0%						
Test for overall effect: Z = 0.62 (P = 0.53)						
**8.5.2 Lower PEEP**						
Relax 2020	15	493	20	476	11.6%	0.72 [0.38, 1.40]
**Subtotal (95% CI)**		**493**		**476**	**11.6%**	**0.72 [0.38, 1.40]**
Total events	15		20			
Heterogeneity: Not applicable						
Test for overall effect: Z = 0.96 (P = 0.34)						
**Total (95% CI)**		**632**		**623**	**100.0%**	**1.02 [0.81, 1.28]**
Total events	65		74			
Heterogeneity: Tau² = 0.01; Chi² = 4.51, df = 4 (P = 0.34); I² = 11%						
Test for overall effect: Z = 0.15 (P = 0.88)						
Test for subgroup differences: Chi² = 1.23, df = 1 (P = 0.27), I² = 18.7%						

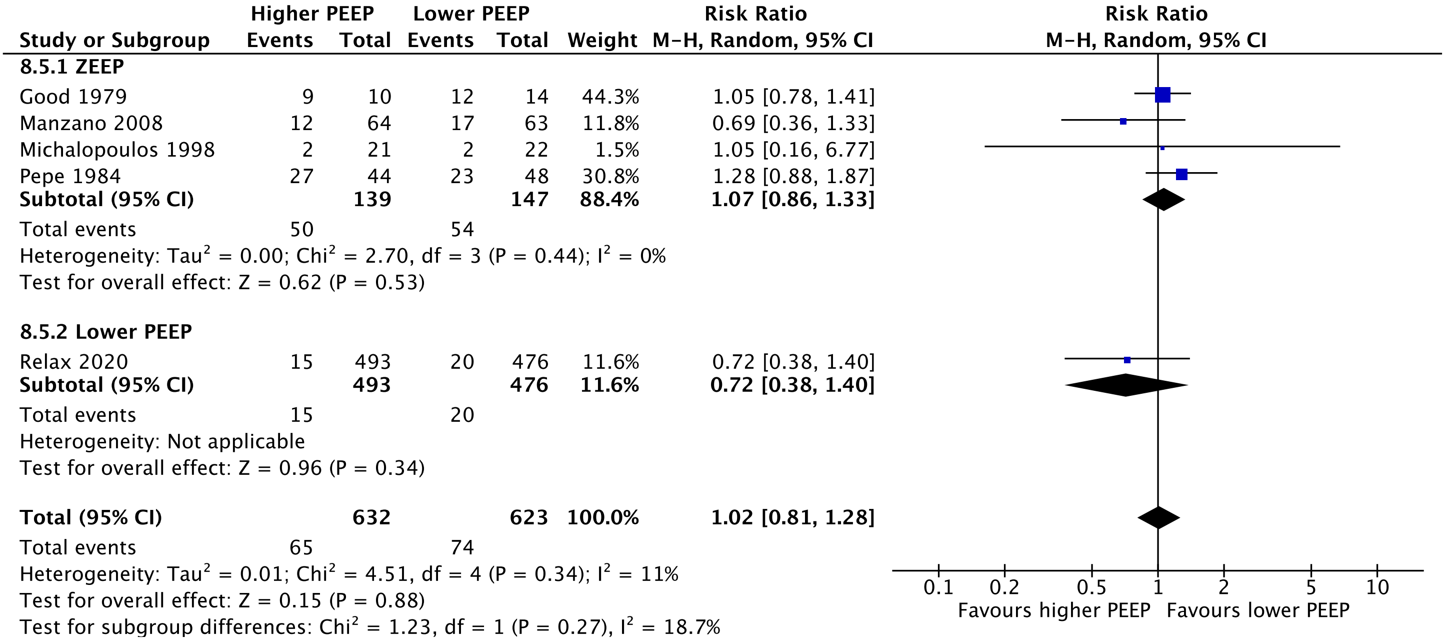

Subgroup analysis (ZEEP vs. lower PEEP)

Barotrauma

Study or Subgroup	Higher PEEP Events	Higher PEEP Total	Lower PEEP Events	Lower PEEP Total	Weight	Risk Ratio M-H, Random, 95% CI
**8.6.1 ZEEP**						
Good 1979	0	10	0	14		Not estimable
Manzano 2008	1	64	5	63	2.8%	0.20 [0.02, 1.64]
Michalopoulos 1998	0	21	0	22		Not estimable
Pepe 1984	19	44	24	48	63.5%	0.86 [0.56, 1.34]
Weigelt 1979	5	45	4	34	8.1%	0.94 [0.27, 3.25]
**Subtotal (95% CI)**		**184**		**181**	**74.3%**	**0.83 [0.55, 1.24]**
Total events	25		33			
Heterogeneity: Tau² = 0.00; Chi² = 1.95, df = 2 (P = 0.38); I² = 0%						
Test for overall effect: Z = 0.92 (P = 0.36)						
**8.6.2 Lower PEEP**						
Nelson 1987	1	20	0	18	1.3%	2.71 [0.12, 62.70]
Relax 2020	12	493	19	476	24.4%	0.61 [0.30, 1.24]
**Subtotal (95% CI)**		**513**		**494**	**25.7%**	**0.66 [0.33, 1.31]**
Total events	13		19			
Heterogeneity: Tau² = 0.00; Chi² = 0.83, df = 1 (P = 0.36); I² = 0%						
Test for overall effect: Z = 1.19 (P = 0.23)						
**Total (95% CI)**		**697**		**675**	**100.0%**	**0.78 [0.55, 1.11]**
Total events	38		52			
Heterogeneity: Tau² = 0.00; Chi² = 3.10, df = 4 (P = 0.54); I² = 0%						
Test for overall effect: Z = 1.40 (P = 0.16)						
Test for subgroup differences: Chi² = 0.31, df = 1 (P = 0.58), I² = 0%						

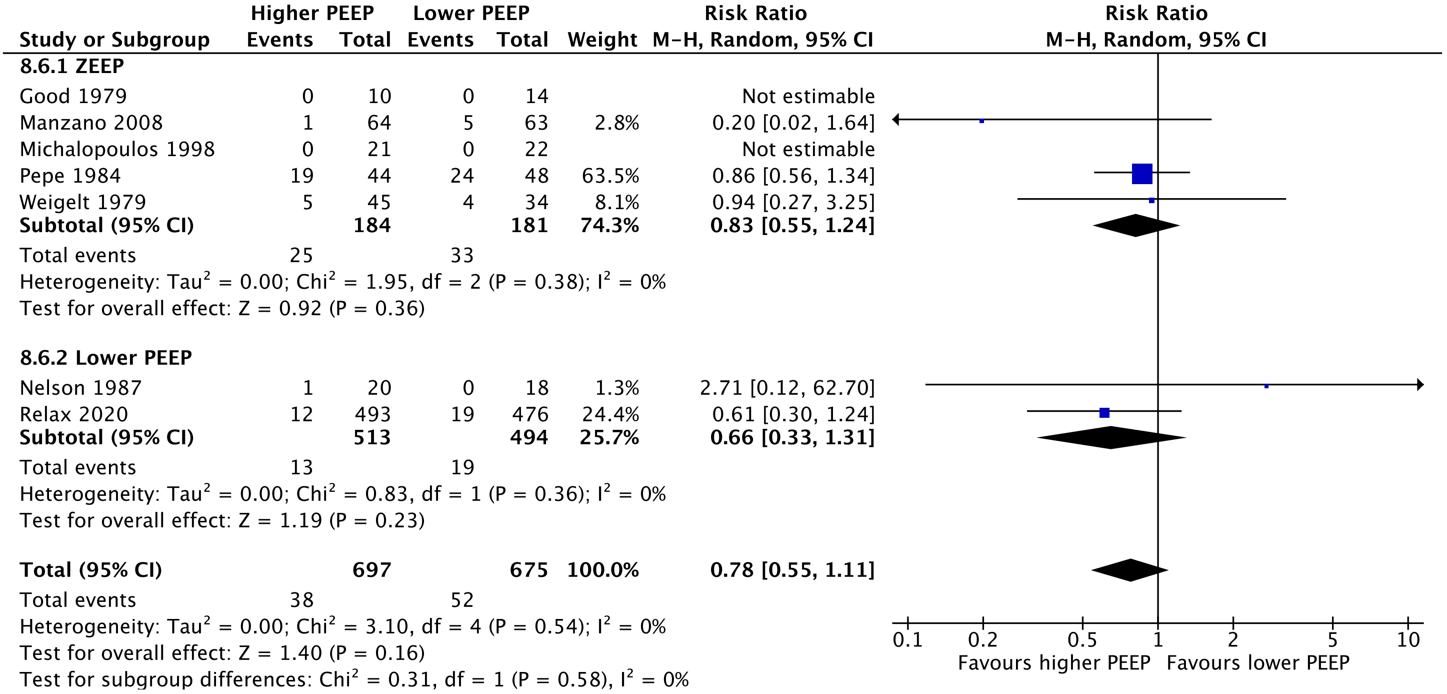

Subgroup analysis (ZEEP vs. lower PEEP)

Ventilator-associated pneumonia

Study or Subgroup	Higher PEEP Events	Higher PEEP Total	Lower PEEP Events	Lower PEEP Total	Weight	Risk Ratio M-H, Random, 95% CI
**8.7.1 ZEEP**						
Manzano 2008	6	64	16	63	42.8%	0.37 [0.15, 0.88]
Pepe 1984	4	44	6	48	26.3%	0.73 [0.22, 2.41]
**Subtotal (95% CI)**		**108**		**111**	**69.1%**	**0.47 [0.23, 0.94]**
Total events	10		22			
Heterogeneity: $Tau^2 = 0.00$; $Chi^2 = 0.81$, df = 1 (P = 0.37); $I^2 = 0\%$						
Test for overall effect: Z = 2.12 (P = 0.03)						
**8.7.2 Lower PEEP**						
Relax 2020	7	493	6	476	30.9%	1.13 [0.38, 3.33]
**Subtotal (95% CI)**		**493**		**476**	**30.9%**	**1.13 [0.38, 3.33]**
Total events	7		6			
Heterogeneity: Not applicable						
Test for overall effect: Z = 0.22 (P = 0.83)						
**Total (95% CI)**		**601**		**587**	**100.0%**	**0.62 [0.32, 1.23]**
Total events	17		28			
Heterogeneity: $Tau^2 = 0.08$; $Chi^2 = 2.59$, df = 2 (P = 0.27); $I^2 = 23\%$						
Test for overall effect: Z = 1.36 (P = 0.17)						
Test for subgroup differences: $Chi^2 = 1.79$, df = 1 (P = 0.18), $I^2 = 44.0\%$						

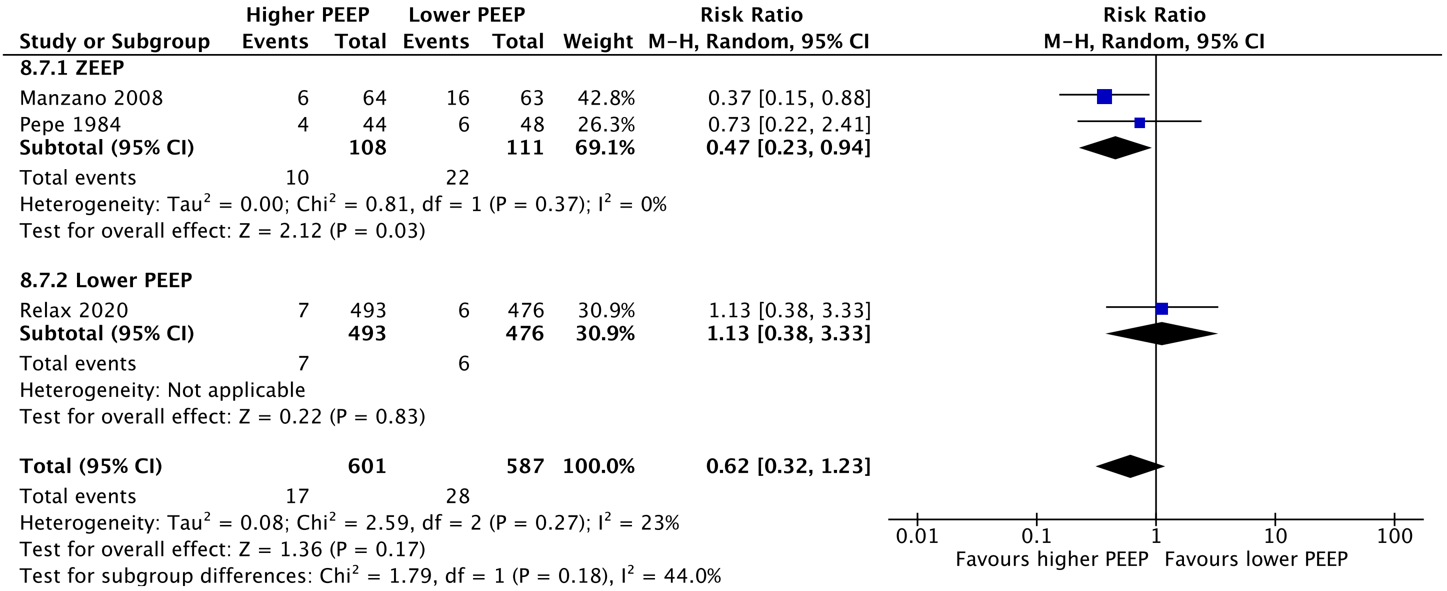

Subgroup analysis (ZEEP vs. lower PEEP)

ARDS

Study or Subgroup	Higher PEEP Events	Higher PEEP Total	Lower PEEP Events	Lower PEEP Total	Weight	Risk Ratio M-H, Random, 95% CI
**8.8.1 ZEEP**						
Korovesi 2016	0	15	0	12		Not estimable
Koutsoukou 2006	0	11	1	10	2.1%	0.31 [0.01, 6.74]
Manzano 2008	3	64	9	63	11.9%	0.33 [0.09, 1.16]
Pepe 1984	11	44	13	48	33.3%	0.92 [0.46, 1.84]
Weigelt 1979	9	45	18	34	35.4%	0.38 [0.19, 0.73]
**Subtotal (95% CI)**		**179**		**167**	**82.7%**	**0.52 [0.29, 0.92]**
Total events	23		41			
Heterogeneity: $Tau^2 = 0.10$; $Chi^2 = 4.16$, df = 3 (P = 0.24); $I^2 = 28\%$						
Test for overall effect: Z = 2.23 (P = 0.03)						
**8.8.2 Lower PEEP**						
Relax 2020	5	493	13	476	17.3%	0.37 [0.13, 1.03]
**Subtotal (95% CI)**		**493**		**476**	**17.3%**	**0.37 [0.13, 1.03]**
Total events	5		13			
Heterogeneity: Not applicable						
Test for overall effect: Z = 1.90 (P = 0.06)						
**Total (95% CI)**		**672**		**643**	**100.0%**	**0.50 [0.32, 0.78]**
Total events	28		54			
Heterogeneity: $Tau^2 = 0.04$; $Chi^2 = 4.59$, df = 4 (P = 0.33); $I^2 = 13\%$						
Test for overall effect: Z = 3.03 (P = 0.002)						
Test for subgroup differences: $Chi^2 = 0.31$, df = 1 (P = 0.58), $I^2 = 0\%$						

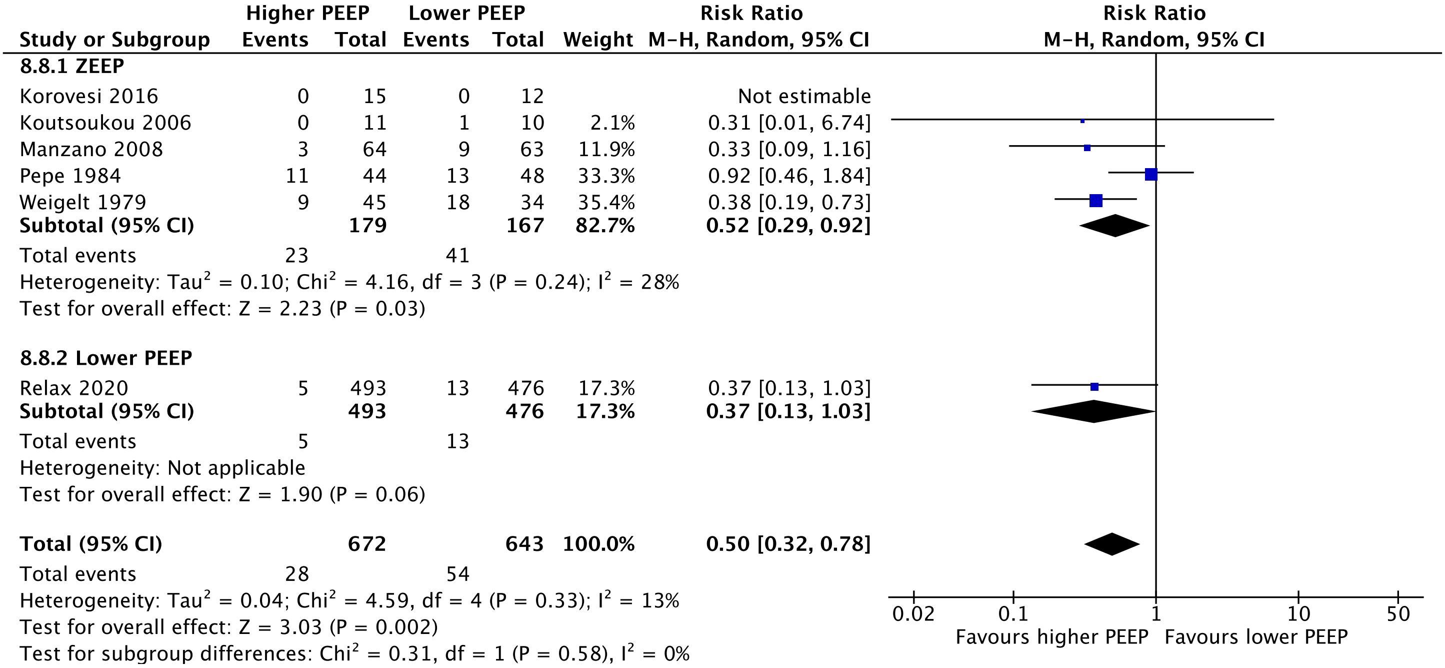

Subgroup analysis (ZEEP vs. lower PEEP)

Cardiac index

	Higher PEEP			Lower PEEP				Mean Difference
**Study or Subgroup**	**Mean**	**SD**	**Total**	**Mean**	**SD**	**Total**	**Weight**	**IV, Random, 95% CI**
**8.9.1 ZEEP**								
Dyhr 2002	2.2	0.6	7	2.1	1.1	8	8.2%	0.10 [−0.78, 0.98]
**Subtotal (95% CI)**			**7**			**8**	**8.2%**	**0.10 [−0.78, 0.98]**
Heterogeneity: Not applicable								
Test for overall effect: Z = 0.22 (P = 0.82)								
**8.9.2 Lower PEEP**								
Collier 2002	3.1	0.86	40	3.1	0.64	44	59.6%	0.00 [−0.33, 0.33]
Holland 2007	3	0.6	14	2.9	0.6	14	32.2%	0.10 [−0.34, 0.54]
**Subtotal (95% CI)**			**54**			**58**	**91.8%**	**0.04 [−0.23, 0.30]**
Heterogeneity: $Tau^2 = 0.00$; $Chi^2 = 0.13$, df = 1 (P = 0.72); $I^2 = 0\%$								
Test for overall effect: Z = 0.26 (P = 0.79)								
**Total (95% CI)**			**61**			**66**	**100.0%**	**0.04 [−0.21, 0.29]**
Heterogeneity: $Tau^2 = 0.00$; $Chi^2 = 0.15$, df = 2 (P = 0.93); $I^2 = 0\%$								
Test for overall effect: Z = 0.31 (P = 0.75)								
Test for subgroup differences: $Chi^2 = 0.02$, df = 1 (P = 0.89), $I^2 = 0\%$								

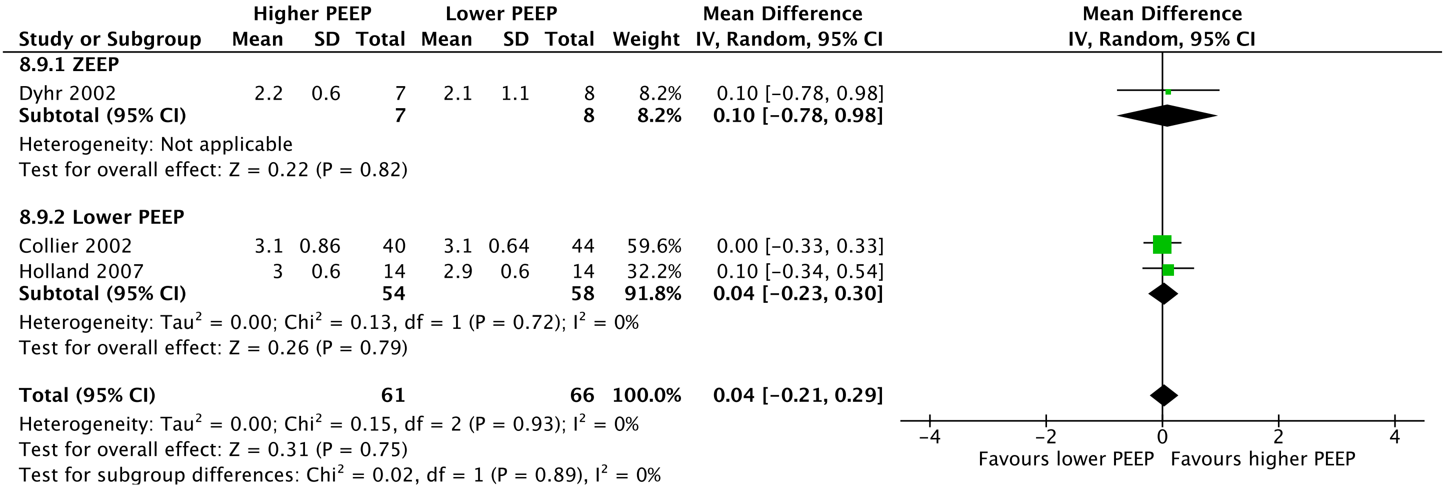

Subgroup analysis (ZEEP vs. lower PEEP)

Central venous pressure

	Higher PEEP			Lower PEEP				Mean Difference
**Study or Subgroup**	**Mean**	**SD**	**Total**	**Mean**	**SD**	**Total**	**Weight**	**IV, Random, 95% CI**
**8.10.1 ZEEP**								
Dyhr 2002	13	11	7	9.7	7.1	8	1.1%	3.30 [−6.22, 12.82]
Lesur 2010	12.5	1.5	30	10.8	2.9	33	78.7%	1.70 [0.57, 2.83]
**Subtotal (95% CI)**			**37**			**41**	**79.8%**	**1.72 [0.60, 2.84]**
Heterogeneity: $Tau^2 = 0.00$; $Chi^2 = 0.11$, df = 1 (P = 0.74); $I^2 = 0\%$								
Test for overall effect: Z = 3.02 (P = 0.003)								
**8.10.2 Lower PEEP**								
Holland 2007	9	3	14	9	3	14	20.2%	0.00 [−2.22, 2.22]
**Subtotal (95% CI)**			**14**			**14**	**20.2%**	**0.00 [−2.22, 2.22]**
Heterogeneity: Not applicable								
Test for overall effect: Z = 0.00 (P = 1.00)								
**Total (95% CI)**			**51**			**55**	**100.0%**	**1.37 [0.38, 2.37]**
Heterogeneity: $Tau^2 = 0.00$; $Chi^2 = 1.95$, df = 2 (P = 0.38); $I^2 = 0\%$								
Test for overall effect: Z = 2.70 (P = 0.007)								
Test for subgroup differences: $Chi^2 = 1.84$, df = 1 (P = 0.17), $I^2 = 45.7\%$								

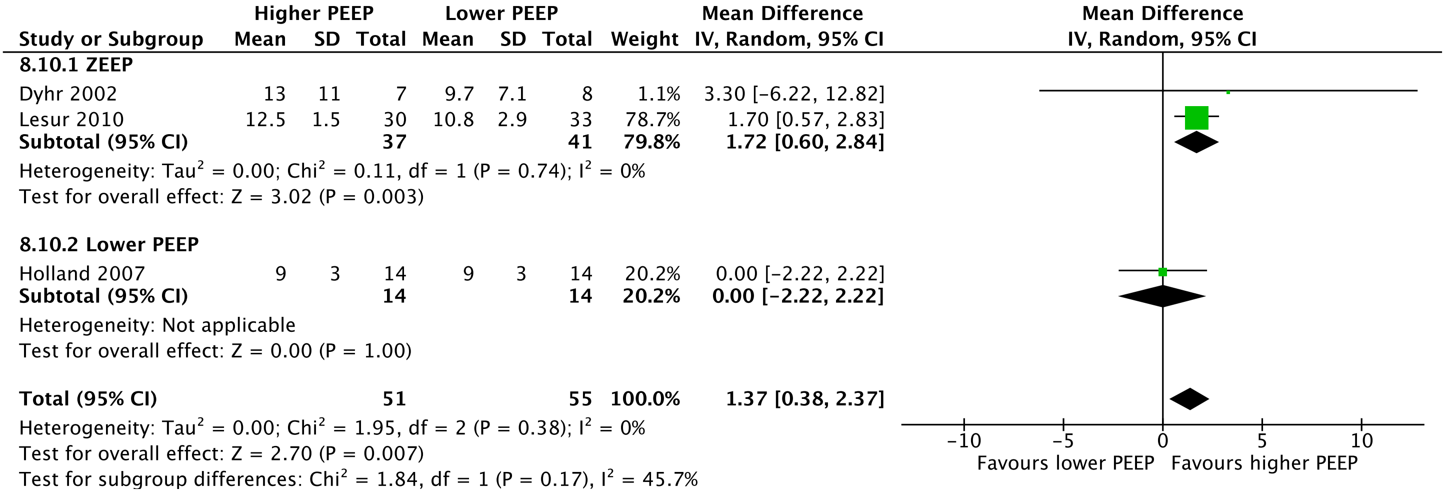

Subgroup analysis (ZEEP vs. lower PEEP)

Postoperative bleeding

	Higher PEEP			Lower PEEP				Mean Difference
**Study or Subgroup**	**Mean**	**SD**	**Total**	**Mean**	**SD**	**Total**	**Weight**	**IV, Random, 95% CI**
**8.11.1 ZEEP**								
Zurich 1982	542	239	41	562	261	476	65.8%	−20.00 [−96.82, 56.82]
**Subtotal (95% CI)**			**41**			**476**	**65.8%**	**−20.00 [−96.82, 56.82]**
Heterogeneity: Not applicable								
Test for overall effect: Z = 0.51 (P = 0.61)								
**8.11.2 Lower PEEP**								
Collier 2002	703	395	40	587	392	44	34.2%	116.00 [−52.52, 284.52]
**Subtotal (95% CI)**			**40**			**44**	**34.2%**	**116.00 [−52.52, 284.52]**
Heterogeneity: Not applicable								
Test for overall effect: Z = 1.35 (P = 0.18)								
**Total (95% CI)**			**81**			**520**	**100.0%**	**26.47 [−99.95, 152.89]**

Heterogeneity: $Tau^2 = 4783.35$; $Chi^2 = 2.07$, df = 1 (P = 0.15); $I^2 = 52\%$
Test for overall effect: Z = 0.41 (P = 0.68)
Test for subgroup differences: $Chi^2 = 2.07$, df = 1 (P = 0.15), $I^2 = 51.7\%$

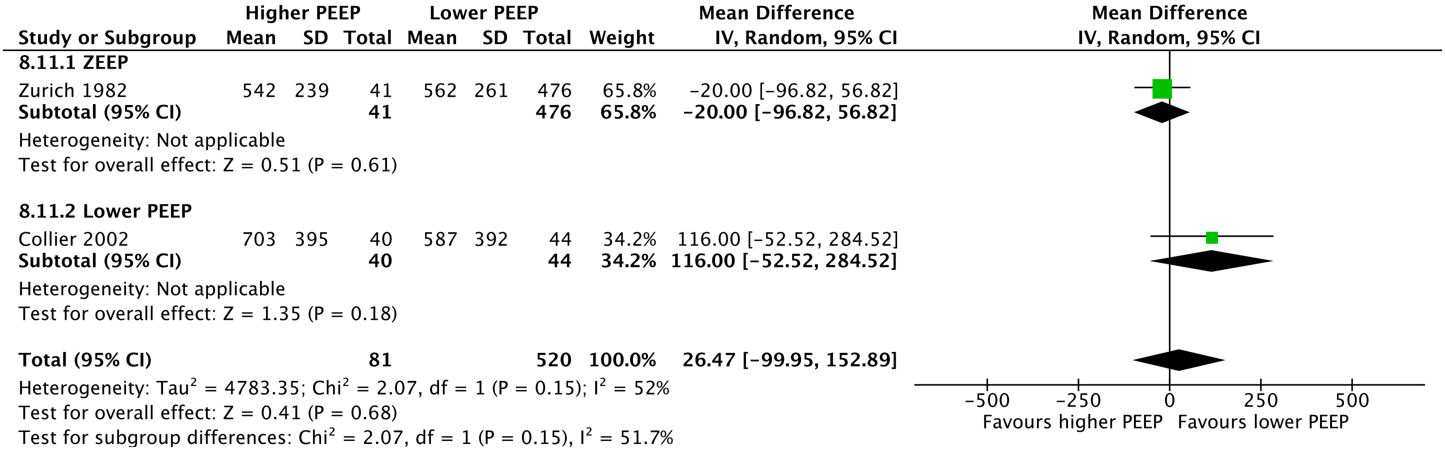

Subgroup analysis (ZEEP vs. lower PEEP)

Packed red blood cell transfusion

Study or Subgroup	Higher PEEP Mean	Higher PEEP SD	Higher PEEP Total	Lower PEEP Mean	Lower PEEP SD	Lower PEEP Total	Weight	Mean Difference IV, Random, 95% CI
**8.12.1 ZEEP**								
Zurich 1982	0.33	0.87	41	0.75	1.42	44	62.5%	−0.42 [−0.92, 0.08]
**Subtotal (95% CI)**			**41**			**44**	**62.5%**	**−0.42 [−0.92, 0.08]**
Heterogeneity: Not applicable								
Test for overall effect: Z = 1.66 (P = 0.10)								
**8.12.2 Lower PEEP**								
Collier 2002	0.8	1.4	40	1.1	1.6	44	37.5%	−0.30 [−0.94, 0.34]
Relax 2020	1.7	0.7	493	1	0	476		Not estimable
**Subtotal (95% CI)**			**533**			**520**	**37.5%**	**−0.30 [−0.94, 0.34]**
Heterogeneity: Not applicable								
Test for overall effect: Z = 0.92 (P = 0.36)								
**Total (95% CI)**			**574**			**564**	**100.0%**	**−0.38 [−0.77, 0.02]**

Heterogeneity: $Tau^2 = 0.00$; $Chi^2 = 0.08$, df = 1 (P = 0.77); $I^2 = 0\%$
Test for overall effect: Z = 1.87 (P = 0.06)
Test for subgroup differences: $Chi^2 = 0.08$, df = 1 (P = 0.77), $I^2 = 0\%$

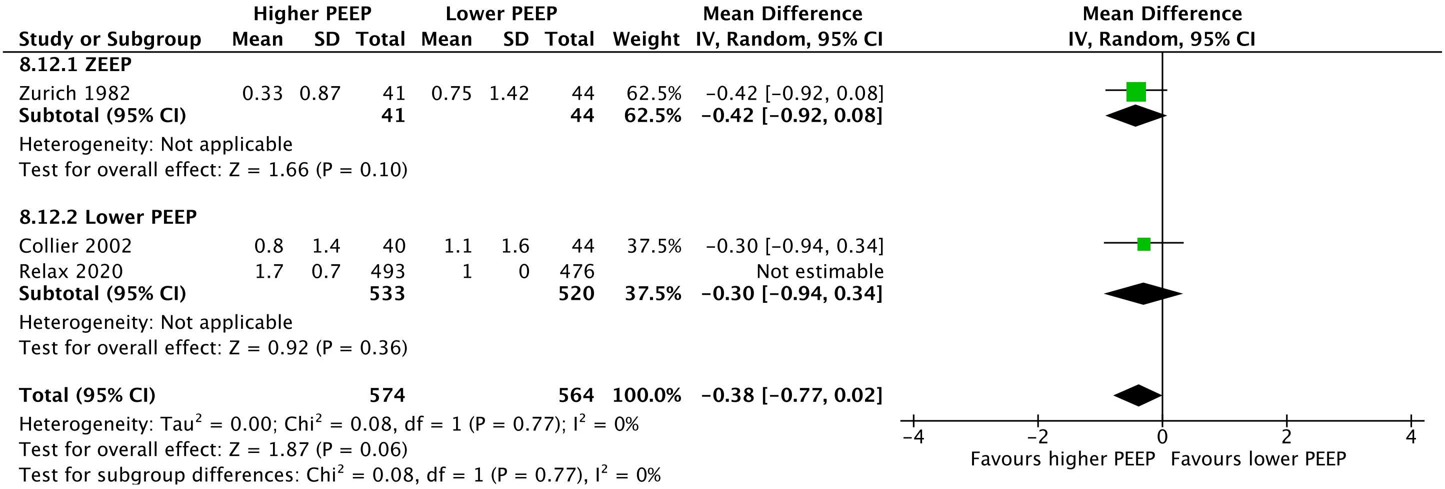

Subgroup analysis (ZEEP vs. lower PEEP)

Duration of ventilation

	Higher PEEP			Lower PEEP				Std. Mean Difference
**Study or Subgroup**	**Mean**	**SD**	**Total**	**Mean**	**SD**	**Total**	**Weight**	**IV, Random, 95% CI**
**8.13.1 ZEEP**								
Feeley 1975	258	217	12	259	149	13	6.4%	−0.01 [−0.79, 0.78]
Good 1979	15.7	0.4	10	14.8	0.5	14	4.5%	1.88 [0.88, 2.88]
Lesur 2010	9.2	8.8	30	9.2	8.5	33	10.9%	0.00 [−0.49, 0.49]
Manzano 2008	5.8	6.8	64	6.5	6.2	63	14.2%	−0.11 [−0.45, 0.24]
Weigelt 1979	9.3	13	45	14	21.7	34	11.9%	−0.27 [−0.72, 0.18]
**Subtotal (95% CI)**			**161**			**157**	**47.8%**	**0.15 [−0.33, 0.63]**
Heterogeneity: $Tau^2 = 0.21$; $Chi^2 = 15.37$, df = 4 (P = 0.004); $I^2 = 74\%$								
Test for overall effect: Z = 0.62 (P = 0.53)								
**8.13.2 Lower PEEP**								
Collier 2002	409.1	208.6	40	439.3	277.7	44	12.3%	−0.12 [−0.55, 0.31]
Lago Borges 2014	5.1	2.9	45	6.8	3.2	44	12.4%	−0.55 [−0.98, −0.13]
Marvel 1986	9.3	0.6	12	0	0	0		Not estimable
Nelson 1987	5.3	5	20	3.4	3	18	8.2%	0.45 [−0.20, 1.09]
Relax 2020	4.8	6.6	493	5.5	7.4	476	19.2%	−0.10 [−0.23, 0.03]
**Subtotal (95% CI)**			**610**			**582**	**52.2%**	**−0.13 [−0.41, 0.15]**
Heterogeneity: $Tau^2 = 0.04$; $Chi^2 = 7.04$, df = 3 (P = 0.07); $I^2 = 57\%$								
Test for overall effect: Z = 0.93 (P = 0.35)								
**Total (95% CI)**			**771**			**739**	**100.0%**	**−0.03 [−0.27, 0.21]**
Heterogeneity: $Tau^2 = 0.07$; $Chi^2 = 23.01$, df = 8 (P = 0.003); $I^2 = 65\%$								
Test for overall effect: Z = 0.24 (P = 0.81)								
Test for subgroup differences: $Chi^2 = 1.01$, df = 1 (P = 0.32), $I^2 = 0.9\%$								

Std. Mean Difference
IV, Random, 95% CI

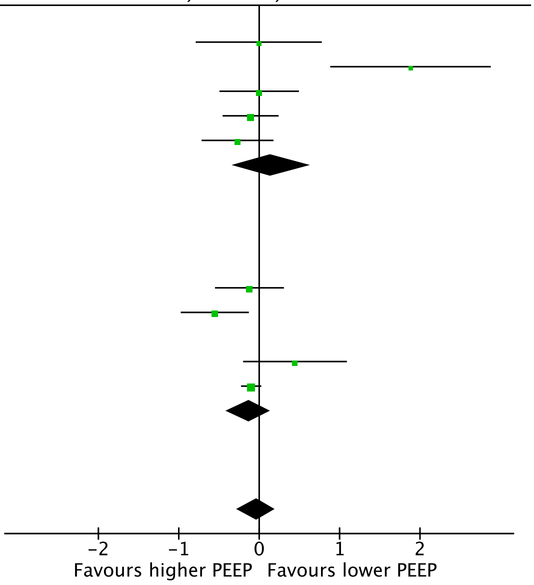

Subgroup analysis (ZEEP vs. lower PEEP)

ICU stay

	Higher PEEP			Lower PEEP				Mean Difference
**Study or Subgroup**	**Mean**	**SD**	**Total**	**Mean**	**SD**	**Total**	**Weight**	**IV, Random, 95% CI**
**8.14.1 ZEEP**								
Korovesi 2011	17.2	10.1	15	14.4	8.44	12	4.6%	2.80 [−4.19, 9.79]
Manzano 2008	10.5	9.8	64	12.3	11.4	63	15.5%	−1.80 [−5.50, 1.90]
Weigelt 1979	11.7	16.8	45	21	32.5	34	1.6%	−9.30 [−21.28, 2.68]
**Subtotal (95% CI)**			**124**			**109**	**21.6%**	**−1.41 [−6.15, 3.32]**
Heterogeneity: $Tau^2 = 6.71; Chi^2 = 3.10, df = 2 (P = 0.21); I^2 = 36\%$								
Test for overall effect: Z = 0.59 (P = 0.56)								
**8.14.2 Lower PEEP**								
Relax 2020	7.2	10.3	493	8.1	11.5	476	78.4%	−0.90 [−2.28, 0.48]
**Subtotal (95% CI)**			**493**			**476**	**78.4%**	**−0.90 [−2.28, 0.48]**
Heterogeneity: Not applicable								
Test for overall effect: Z = 1.28 (P = 0.20)								
**Total (95% CI)**			**617**			**585**	**100.0%**	**−1.00 [−2.51, 0.51]**
Heterogeneity: $Tau^2 = 0.26; Chi^2 = 3.18, df = 3 (P = 0.37); I^2 = 6\%$								
Test for overall effect: Z = 1.30 (P = 0.19)								
Test for subgroup differences: $Chi^2 = 0.04, df = 1 (P = 0.84), I^2 = 0\%$								

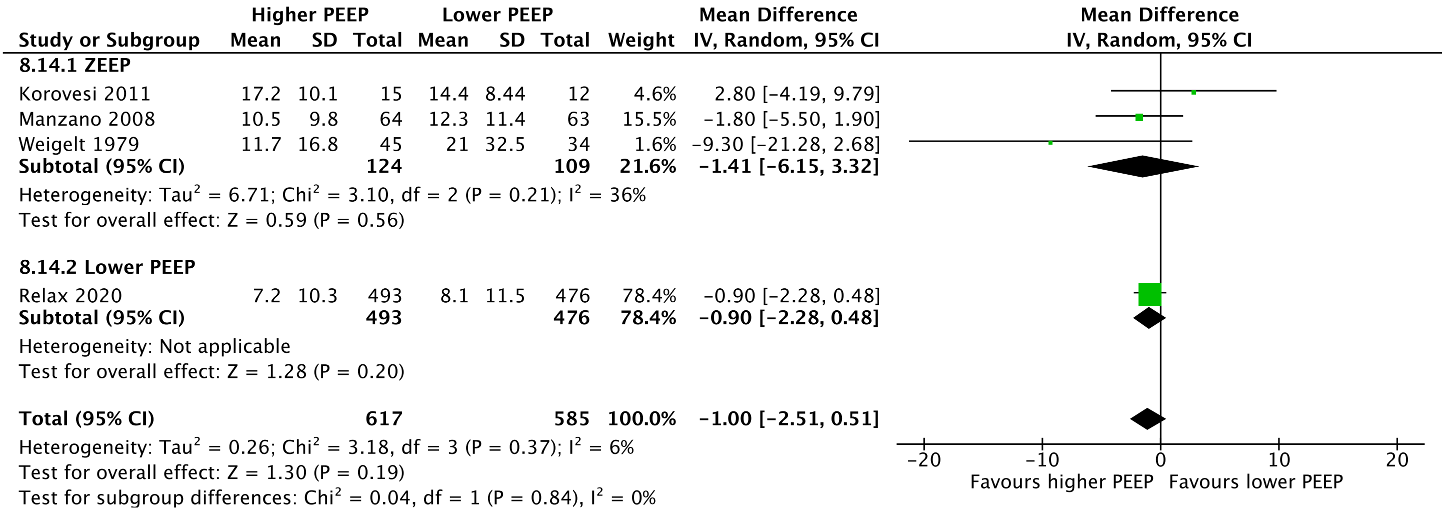

Subgroup analysis (ZEEP vs. lower PEEP)

Hospital stay

	Higher PEEP			Lower PEEP				Mean Difference
Study or Subgroup	Mean	SD	Total	Mean	SD	Total	Weight	IV, Random, 95% CI
**8.15.1 ZEEP**								
Manzano 2008	19.5	18.2	64	26.3	22	63	0.9%	-6.80 [-13.83, 0.23]
**Subtotal (95% CI)**			**64**			**63**	**0.9%**	**-6.80 [-13.83, 0.23]**
Heterogeneity: Not applicable								
Test for overall effect: Z = 1.90 (P = 0.06)								
**8.15.2 Lower PEEP**								
Collier 2002	5.7	2.5	40	5.2	1.7	44	31.1%	0.50 [-0.42, 1.42]
Marvel 1986	8.8	0.5	12	8.9	0.4	15	62.2%	-0.10 [-0.45, 0.25]
Nelson 1987	28	24	20	26	24	18	0.2%	2.00 [-13.28, 17.28]
Relax 2020	19	21.4	493	19.9	22.1	476	5.6%	-0.90 [-3.64, 1.84]
**Subtotal (95% CI)**			**565**			**553**	**99.1%**	**-0.04 [-0.36, 0.29]**
Heterogeneity: $Tau^2 = 0.00$; $Chi^2 = 1.87$, df = 3 (P = 0.60); $I^2 = 0\%$								
Test for overall effect: Z = 0.22 (P = 0.82)								
**Total (95% CI)**			**629**			**616**	**100.0%**	**-0.02 [-0.69, 0.66]**
Heterogeneity: $Tau^2 = 0.16$; $Chi^2 = 5.42$, df = 4 (P = 0.25); $I^2 = 26\%$								
Test for overall effect: Z = 0.04 (P = 0.96)								
Test for subgroup differences: $Chi^2 = 3.55$, df = 1 (P = 0.06), $I^2 = 71.8\%$								

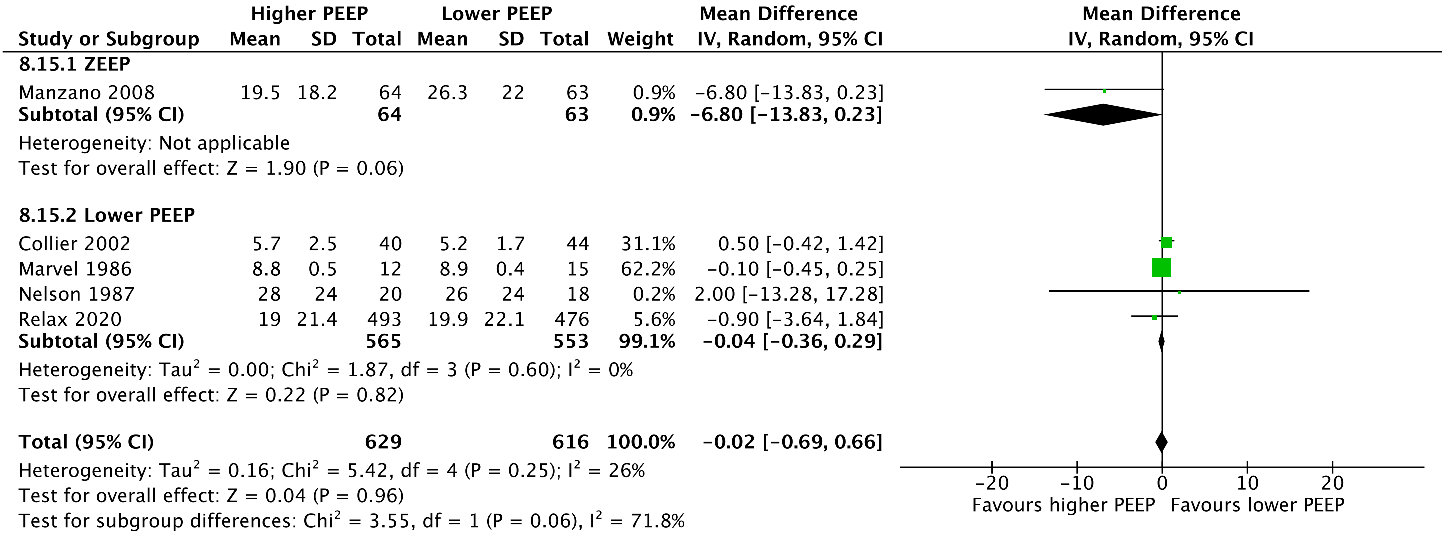

Subgroup analysis (ZEEP vs. lower PEEP)

ICU mortality

Study or Subgroup	Higher PEEP Events	Higher PEEP Total	Lower PEEP Events	Lower PEEP Total	Weight	Risk Ratio M-H, Random, 95% CI	Risk Ratio M-H, Random, 95% CI
**8.16.1 ZEEP**							
Dyhr 2002	0	7	0	8		Not estimable	
Feeley 1975	2	12	1	13	0.5%	2.17 [0.22, 20.94]	
Korovesi 2011	3	15	4	12	1.6%	0.60 [0.17, 2.18]	
**Subtotal (95% CI)**		**34**		**33**	**2.2%**	**0.82 [0.27, 2.52]**	
Total events	5		5				
Heterogeneity: $Tau^2 = 0.00$; $Chi^2 = 0.94$, df = 1 (P = 0.33); $I^2 = 0\%$							
Test for overall effect: Z = 0.34 (P = 0.73)							
**8.16.2 Lower PEEP**							
Nelson 1987	4	20	4	18	1.8%	0.90 [0.26, 3.08]	
Relax 2020	185	492	163	476	96.0%	1.10 [0.93, 1.30]	
**Subtotal (95% CI)**		**512**		**494**	**97.8%**	**1.09 [0.93, 1.29]**	
Total events	189		167				
Heterogeneity: $Tau^2 = 0.00$; $Chi^2 = 0.10$, df = 1 (P = 0.75); $I^2 = 0\%$							
Test for overall effect: Z = 1.05 (P = 0.29)							
**Total (95% CI)**		**546**		**527**	**100.0%**	**1.09 [0.92, 1.28]**	
Total events	194		172				
Heterogeneity: $Tau^2 = 0.00$; $Chi^2 = 1.27$, df = 3 (P = 0.74); $I^2 = 0\%$							
Test for overall effect: Z = 0.99 (P = 0.32)							
Test for subgroup differences: $Chi^2 = 0.25$, df = 1 (P = 0.62), $I^2 = 0\%$							

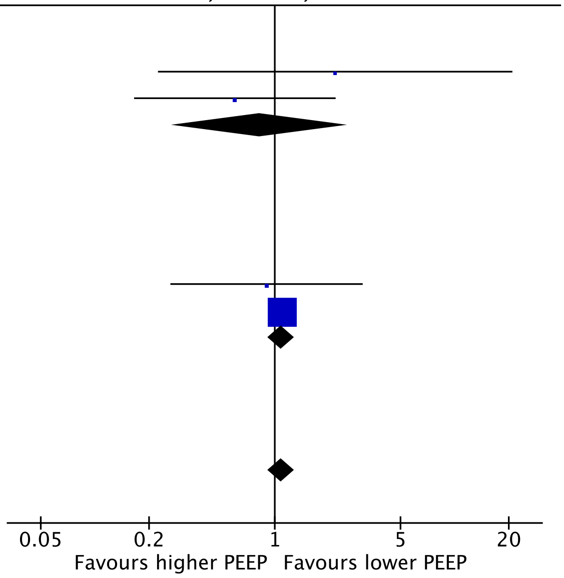

Subgroup analysis (ZEEP vs. lower PEEP)

28-day mortality

	Higher PEEP		Lower PEEP			Risk Ratio
**Study or Subgroup**	**Events**	**Total**	**Events**	**Total**	**Weight**	**M-H, Random, 95% CI**
**8.18.1 ZEEP**						
Lesur 2010	9	30	14	33	28.8%	0.71 [0.36, 1.39]
**Subtotal (95% CI)**		**30**		**33**	**28.8%**	**0.71 [0.36, 1.39]**
Total events	9		14			
Heterogeneity: Not applicable						
Test for overall effect: Z = 1.00 (P = 0.31)						
**8.18.2 Lower PEEP**						
Ma 2014	15	60	39	60	33.1%	0.38 [0.24, 0.62]
Relax 2020	207	493	183	476	38.1%	1.09 [0.94, 1.27]
**Subtotal (95% CI)**		**553**		**536**	**71.2%**	**0.66 [0.24, 1.85]**
Total events	222		222			
Heterogeneity: $Tau^2 = 0.51$; $Chi^2 = 16.79$, df = 1 (P < 0.0001); $I^2 = 94\%$						
Test for overall effect: Z = 0.78 (P = 0.43)						
**Total (95% CI)**		**583**		**569**	**100.0%**	**0.68 [0.33, 1.40]**
Total events	231		236			
Heterogeneity: $Tau^2 = 0.35$; $Chi^2 = 17.71$, df = 2 (P = 0.0001); $I^2 = 89\%$						
Test for overall effect: Z = 1.05 (P = 0.30)						
Test for subgroup differences: $Chi^2 = 0.01$, df = 1 (P = 0.92), $I^2 = 0\%$						

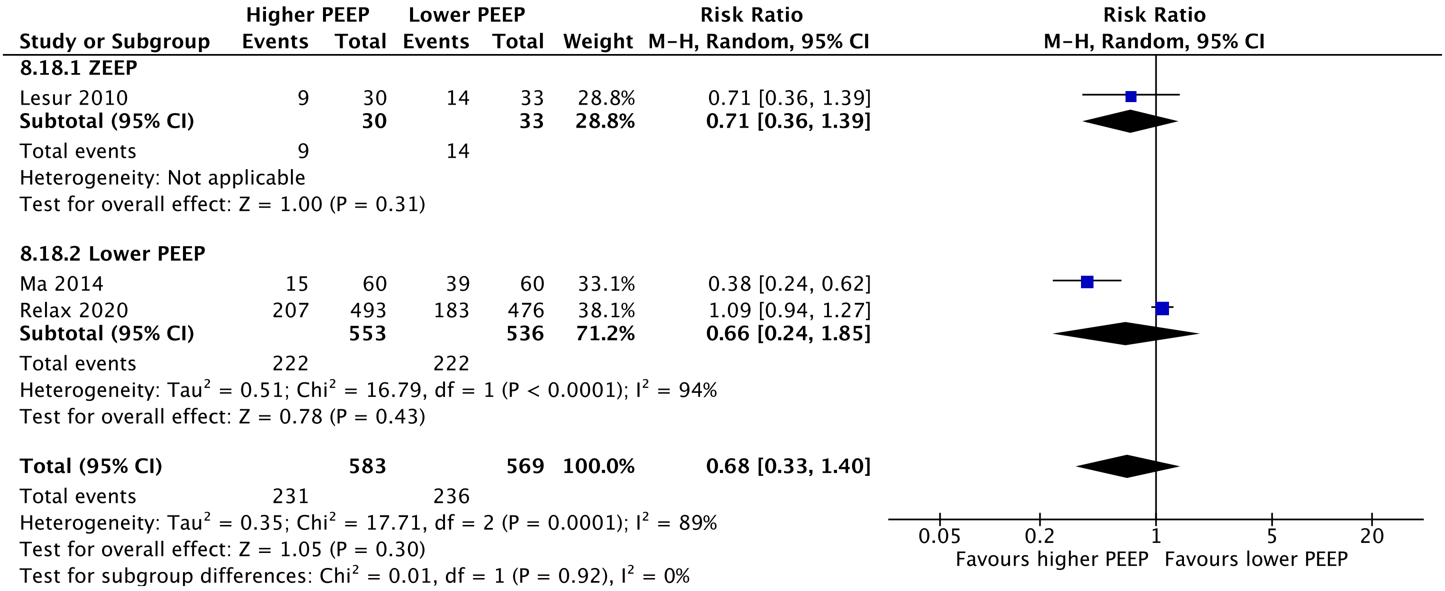

Subgroup analysis (TV > 8 mL/kg vs. TV < 8 mL/kg)

Hospital mortality

Study or Subgroup	Higher PEEP Events	Higher PEEP Total	Lower PEEP Events	Lower PEEP Total	Weight	Risk Ratio M-H, Random, 95% CI
**9.15.1 VT>8**						
Collier 2002	1	40	1	44	0.2%	1.10 [0.07, 17.01]
Pepe 1984	13	44	18	48	5.2%	0.79 [0.44, 1.41]
Weigelt 1979	16	45	17	34	6.7%	0.71 [0.42, 1.19]
**Subtotal (95% CI)**		**129**		**126**	**12.1%**	**0.75 [0.51, 1.10]**
Total events	30		36			
Heterogeneity: $Tau^2 = 0.00$; $Chi^2 = 0.15$, df = 2 (P = 0.93); $I^2 = 0\%$						
Test for overall effect: Z = 1.47 (P = 0.14)						
**9.15.2 VT<8**						
Dyhr 2002	0	7	0	8		Not estimable
Lesur 2010	12	30	16	33	5.6%	0.82 [0.47, 1.45]
Manzano 2008	19	64	16	63	5.5%	1.17 [0.66, 2.06]
Relax 2020	208	489	185	472	76.7%	1.09 [0.93, 1.26]
**Subtotal (95% CI)**		**590**		**576**	**87.9%**	**1.07 [0.93, 1.24]**
Total events	239		217			
Heterogeneity: $Tau^2 = 0.00$; $Chi^2 = 0.95$, df = 2 (P = 0.62); $I^2 = 0\%$						
Test for overall effect: Z = 0.95 (P = 0.34)						
**Total (95% CI)**		**719**		**702**	**100.0%**	**1.03 [0.90, 1.17]**
Total events	269		253			
Heterogeneity: $Tau^2 = 0.00$; $Chi^2 = 4.02$, df = 5 (P = 0.55); $I^2 = 0\%$						
Test for overall effect: Z = 0.38 (P = 0.71)						
Test for subgroup differences: $Chi^2 = 2.93$, df = 1 (P = 0.09), $I^2 = 65.8\%$						

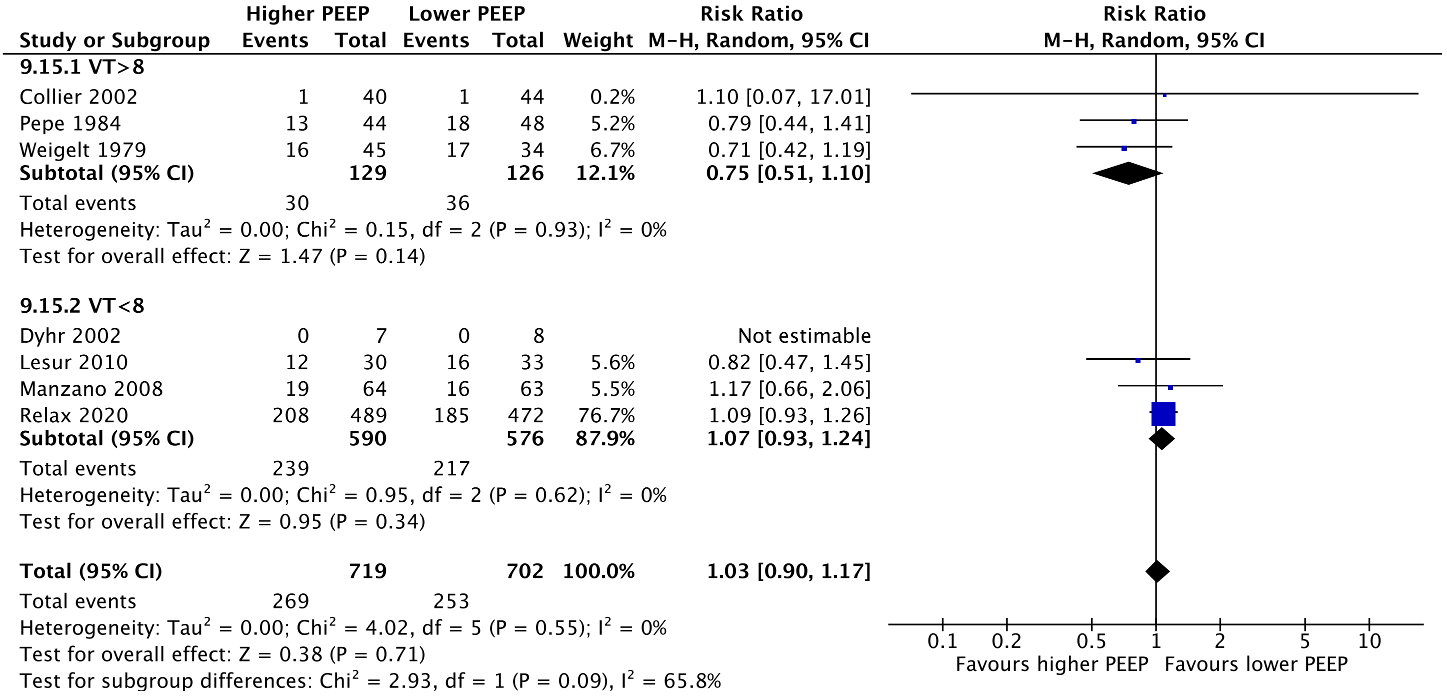

Subgroup analysis (TV > 8 mL/kg vs. TV < 8 mL/kg)

A-aDO2

Study or Subgroup	Higher PEEP Mean	Higher PEEP SD	Higher PEEP Total	Lower PEEP Mean	Lower PEEP SD	Lower PEEP Total	Weight	Std. Mean Difference IV, Random, 95% CI
**9.1.1 VT>8**								
Feeley 1975	10	22	12	102	35	13	23.6%	−3.02 [−4.22, −1.81]
Marvel 1986	168	10	12	203	10	17	23.7%	−3.40 [−4.60, −2.20]
**Subtotal (95% CI)**			**24**			**30**	**47.3%**	**−3.21 [−4.06, −2.36]**
Heterogeneity: Tau² = 0.00; Chi² = 0.20, df = 1 (P = 0.66); I² = 0%								
Test for overall effect: Z = 7.42 (P < 0.00001)								
**9.1.2 VT<8**								
Koutsoukou 2006	100	41	11	87	40	10	25.5%	0.31 [−0.55, 1.17]
Lago Borges 2013	117	33	45	139	34	44	27.2%	−0.65 [−1.08, −0.22]
**Subtotal (95% CI)**			**56**			**54**	**52.7%**	**−0.25 [−1.18, 0.68]**
Heterogeneity: Tau² = 0.34; Chi² = 3.81, df = 1 (P = 0.05); I² = 74%								
Test for overall effect: Z = 0.52 (P = 0.60)								
**Total (95% CI)**			**80**			**84**	**100.0%**	**−1.62 [−3.12, −0.11]**
Heterogeneity: Tau² = 2.13; Chi² = 37.49, df = 3 (P < 0.00001); I² = 92%								
Test for overall effect: Z = 2.10 (P = 0.04)								
Test for subgroup differences: Chi² = 21.32, df = 1 (P < 0.00001), I² = 95.3%								

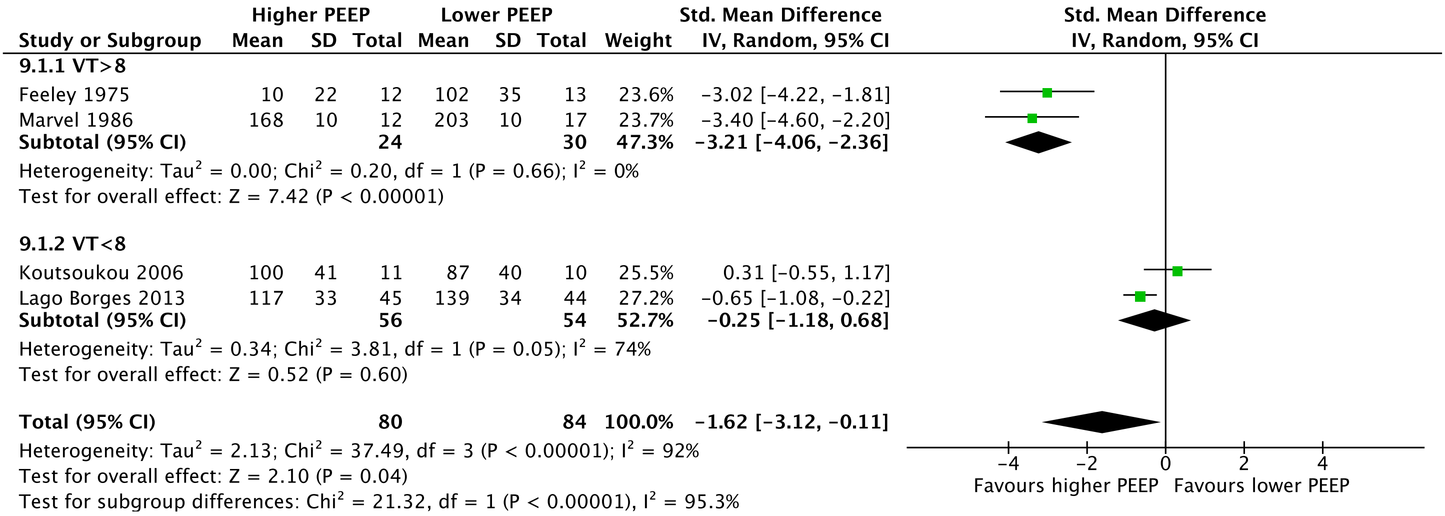

Subgroup analysis (TV > 8 mL/kg vs. TV < 8 mL/kg)

Compliance

	Higher PEEP			Lower PEEP				Mean Difference
**Study or Subgroup**	**Mean**	**SD**	**Total**	**Mean**	**SD**	**Total**	**Weight**	**IV, Random, 95% CI**
**9.2.1 VT>8**								
Weigelt 1979	42	36	45	39	43	34	9.0%	3.00 [−14.88, 20.88]
**Subtotal (95% CI)**			**45**			**34**	**9.0%**	**3.00 [−14.88, 20.88]**
Heterogeneity: Not applicable								
Test for overall effect: Z = 0.33 (P = 0.74)								
**9.2.2 VT<8**								
Koutsoukou 2006	62	14	11	53	11	10	24.9%	9.00 [−1.72, 19.72]
Lago Borges 2013	56	19	45	47	12	44	66.1%	9.00 [2.41, 15.59]
**Subtotal (95% CI)**			**56**			**54**	**91.0%**	**9.00 [3.39, 14.61]**
Heterogeneity: $Tau^2 = 0.00$; $Chi^2 = 0.00$, df = 1 (P = 1.00); $I^2 = 0\%$								
Test for overall effect: Z = 3.14 (P = 0.002)								
**Total (95% CI)**			**101**			**88**	**100.0%**	**8.46 [3.11, 13.82]**
Heterogeneity: $Tau^2 = 0.00$; $Chi^2 = 0.39$, df = 2 (P = 0.82); $I^2 = 0\%$								
Test for overall effect: Z = 3.10 (P = 0.002)								
Test for subgroup differences: $Chi^2 = 0.39$, df = 1 (P = 0.53), $I^2 = 0\%$								

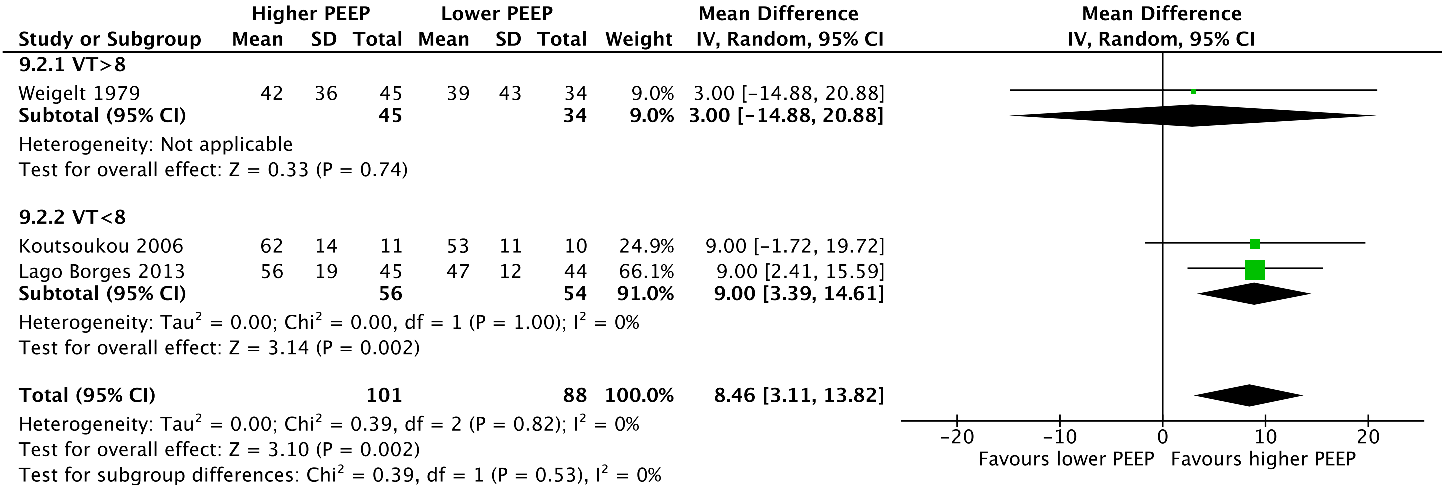

Subgroup analysis (TV > 8 mL/kg vs. TV < 8 mL/kg)

Hypoxemia

Study or Subgroup	Higher PEEP Events	Higher PEEP Total	Lower PEEP Events	Lower PEEP Total	Weight	Risk Ratio M-H, Random, 95% CI
**9.3.1 VT>8**						
Pepe 1984	1	44	4	48	4.0%	0.27 [0.03, 2.35]
**Subtotal (95% CI)**		**44**		**48**	**4.0%**	**0.27 [0.03, 2.35]**
Total events	1		4			
Heterogeneity: Not applicable						
Test for overall effect: Z = 1.18 (P = 0.24)						
**9.3.2 VT<8**						
Lago Borges 2013	19	45	30	44	32.3%	0.62 [0.42, 0.92]
Manzano 2008	12	64	34	63	26.0%	0.35 [0.20, 0.61]
Relax 2020	87	493	98	476	37.7%	0.86 [0.66, 1.11]
**Subtotal (95% CI)**		**602**		**583**	**96.0%**	**0.60 [0.37, 0.96]**
Total events	118		162			
Heterogeneity: $Tau^2 = 0.13$; $Chi^2 = 8.75$, df = 2 (P = 0.01); $I^2 = 77\%$						
Test for overall effect: Z = 2.11 (P = 0.03)						
**Total (95% CI)**		**646**		**631**	**100.0%**	**0.58 [0.37, 0.92]**
Total events	119		166			
Heterogeneity: $Tau^2 = 0.12$; $Chi^2 = 9.47$, df = 3 (P = 0.02); $I^2 = 68\%$						
Test for overall effect: Z = 2.34 (P = 0.02)						
Test for subgroup differences: $Chi^2 = 0.49$, df = 1 (P = 0.48), $I^2 = 0\%$						

Risk Ratio
M-H, Random, 95% CI

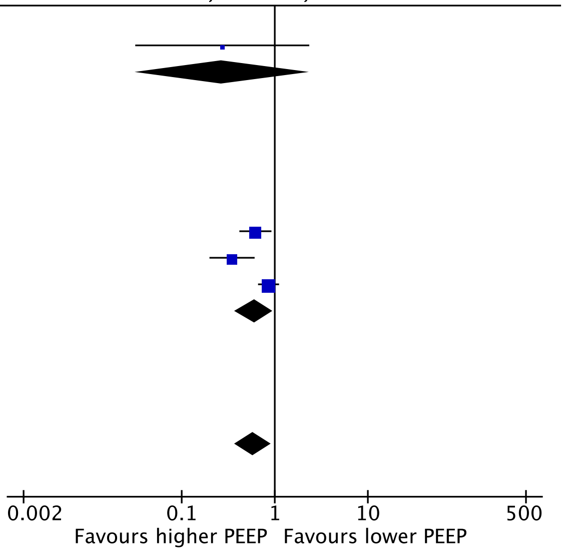

Subgroup analysis (TV > 8 mL/kg vs. TV < 8 mL/kg)

Atelectasis

Study or Subgroup	Higher PEEP Events	Higher PEEP Total	Lower PEEP Events	Lower PEEP Total	Weight	Risk Ratio M-H, Random, 95% CI	Risk Ratio M-H, Random, 95% CI
**9.4.1 VT>8**							
Good 1979	9	10	12	14	39.9%	1.05 [0.78, 1.41]	
Pepe 1984	27	44	23	48	31.2%	1.28 [0.88, 1.87]	
**Subtotal (95% CI)**		**54**		**62**	**71.1%**	**1.13 [0.90, 1.43]**	
Total events	36		35				
Heterogeneity: $Tau^2 = 0.00; Chi^2 = 0.95, df = 1 (P = 0.33); I^2 = 0\%$							
Test for overall effect: Z = 1.05 (P = 0.29)							
**9.4.2 VT<8**							
Manzano 2008	12	64	17	63	14.5%	0.69 [0.36, 1.33]	
Relax 2020	15	493	20	476	14.3%	0.72 [0.38, 1.40]	
**Subtotal (95% CI)**		**557**		**539**	**28.9%**	**0.71 [0.45, 1.13]**	
Total events	27		37				
Heterogeneity: $Tau^2 = 0.00; Chi^2 = 0.01, df = 1 (P = 0.93); I^2 = 0\%$							
Test for overall effect: Z = 1.45 (P = 0.15)							
**Total (95% CI)**		**611**		**601**	**100.0%**	**1.00 [0.76, 1.32]**	
Total events	63		72				
Heterogeneity: $Tau^2 = 0.03; Chi^2 = 4.55, df = 3 (P = 0.21); I^2 = 34\%$							
Test for overall effect: Z = 0.02 (P = 0.99)							
Test for subgroup differences: $Chi^2 = 3.14, df = 1 (P = 0.08), I^2 = 68.1\%$							

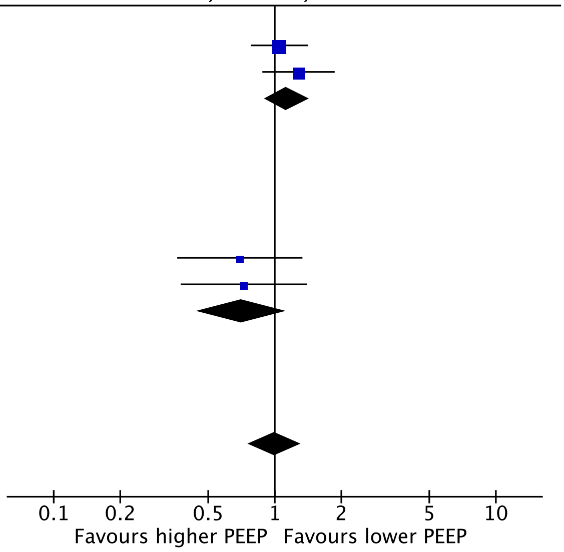

Subgroup analysis (TV > 8 mL/kg vs. TV < 8 mL/kg)

Barotrauma

Study or Subgroup	Higher PEEP Events	Higher PEEP Total	Lower PEEP Events	Lower PEEP Total	Weight	Risk Ratio M-H, Random, 95% CI
**9.5.1 VT>8**						
Good 1979	0	10	0	14		Not estimable
Pepe 1984	19	44	24	48	64.3%	0.86 [0.56, 1.34]
Weigelt 1979	5	45	4	34	8.2%	0.94 [0.27, 3.25]
**Subtotal (95% CI)**		**99**		**96**	**72.5%**	**0.87 [0.58, 1.32]**
Total events	24		28			
Heterogeneity: Tau² = 0.00; Chi² = 0.02, df = 1 (P = 0.89); I² = 0%						
Test for overall effect: Z = 0.64 (P = 0.52)						
**9.5.2 VT<8**						
Manzano 2008	1	64	5	63	2.8%	0.20 [0.02, 1.64]
Relax 2020	12	493	19	476	24.7%	0.61 [0.30, 1.24]
**Subtotal (95% CI)**		**557**		**539**	**27.5%**	**0.54 [0.28, 1.07]**
Total events	13		24			
Heterogeneity: Tau² = 0.00; Chi² = 0.99, df = 1 (P = 0.32); I² = 0%						
Test for overall effect: Z = 1.77 (P = 0.08)						
**Total (95% CI)**		**656**		**635**	**100.0%**	**0.77 [0.54, 1.09]**
Total events	37		52			
Heterogeneity: Tau² = 0.00; Chi² = 2.54, df = 3 (P = 0.47); I² = 0%						
Test for overall effect: Z = 1.48 (P = 0.14)						
Test for subgroup differences: Chi² = 1.37, df = 1 (P = 0.24), I² = 26.9%						

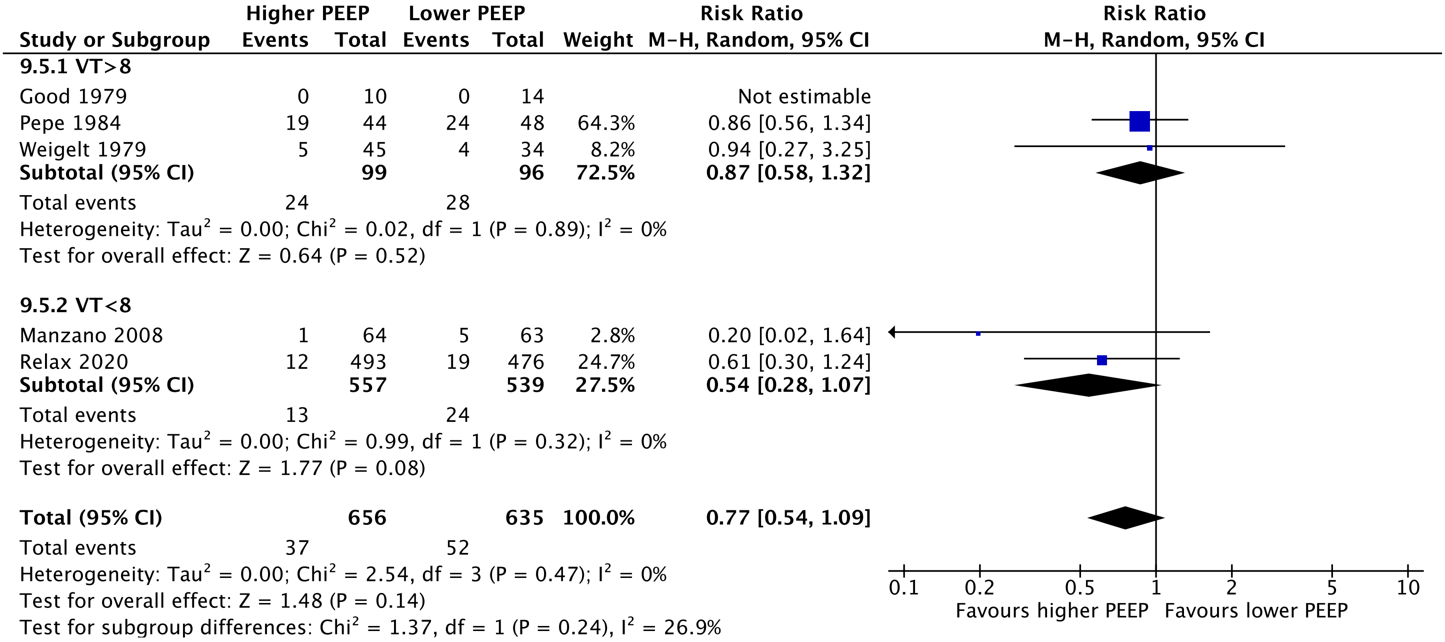

Subgroup analysis (TV > 8 mL/kg vs. TV < 8 mL/kg)

Ventilator-associated pneumonia

	Higher PEEP		Lower PEEP			Risk Ratio
**Study or Subgroup**	**Events**	**Total**	**Events**	**Total**	**Weight**	**M-H, Random, 95% CI**
**9.6.1 VT>8**						
Pepe 1984	4	44	6	48	26.3%	0.73 [0.22, 2.41]
**Subtotal (95% CI)**		**44**		**48**	**26.3%**	**0.73 [0.22, 2.41]**
Total events	4		6			
Heterogeneity: Not applicable						
Test for overall effect: Z = 0.52 (P = 0.60)						
**9.6.2 VT<8**						
Manzano 2008	6	64	16	63	42.8%	0.37 [0.15, 0.88]
Relax 2020	7	493	6	476	30.9%	1.13 [0.38, 3.33]
**Subtotal (95% CI)**		**557**		**539**	**73.7%**	**0.61 [0.21, 1.83]**
Total events	13		22			
Heterogeneity: $Tau^2 = 0.37$; $Chi^2 = 2.47$, df = 1 (P = 0.12); $I^2 = 60\%$						
Test for overall effect: Z = 0.88 (P = 0.38)						
**Total (95% CI)**		**601**		**587**	**100.0%**	**0.62 [0.32, 1.23]**
Total events	17		28			
Heterogeneity: $Tau^2 = 0.08$; $Chi^2 = 2.59$, df = 2 (P = 0.27); $I^2 = 23\%$						
Test for overall effect: Z = 1.36 (P = 0.17)						
Test for subgroup differences: $Chi^2 = 0.04$, df = 1 (P = 0.84), $I^2 = 0\%$						

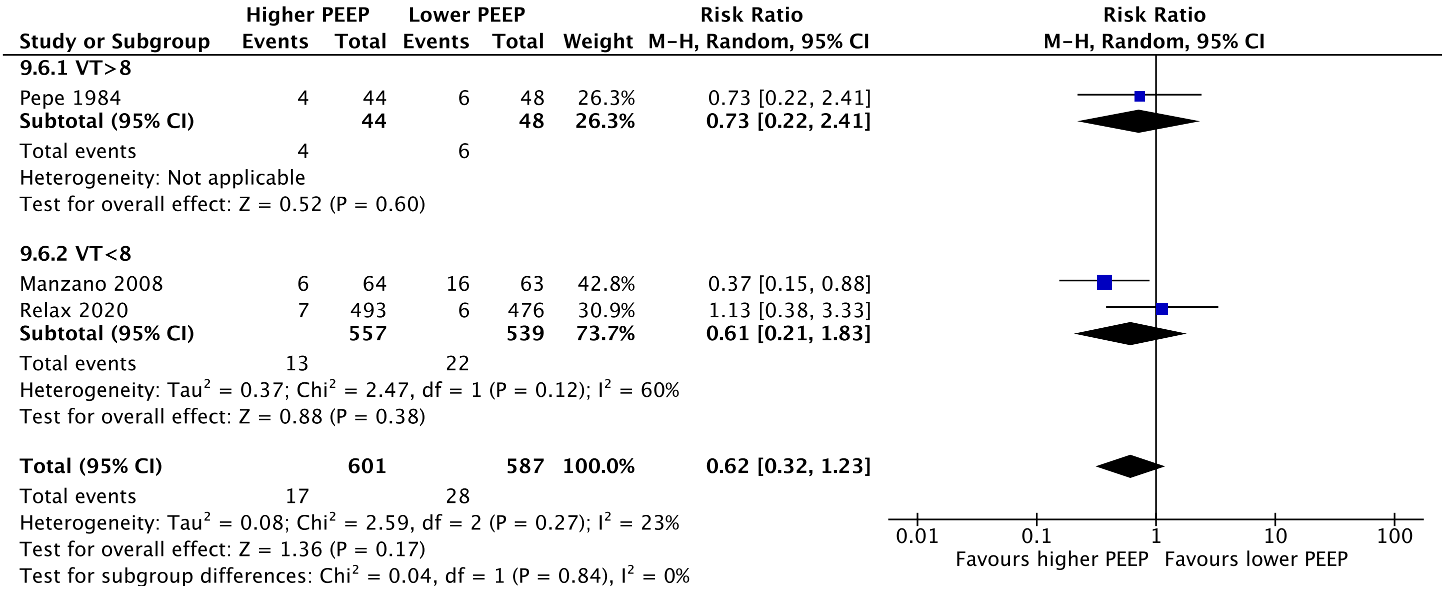

Subgroup analysis (TV > 8 mL/kg vs. TV < 8 mL/kg)

ARDS

Study or Subgroup	Higher PEEP Events	Higher PEEP Total	Lower PEEP Events	Lower PEEP Total	Weight	Risk Ratio M-H, Random, 95% CI
**9.7.1 VT>8**						
Pepe 1984	11	44	13	48	33.3%	0.92 [0.46, 1.84]
Weigelt 1979	9	45	18	34	35.4%	0.38 [0.19, 0.73]
**Subtotal (95% CI)**		**89**		**82**	**68.7%**	**0.59 [0.24, 1.41]**
Total events	20		31			
Heterogeneity: Tau² = 0.28; Chi² = 3.34, df = 1 (P = 0.07); I² = 70%						
Test for overall effect: Z = 1.19 (P = 0.23)						
**9.7.2 VT<8**						
Korovesi 2016	0	15	0	12		Not estimable
Koutsoukou 2006	0	11	1	10	2.1%	0.31 [0.01, 6.74]
Manzano 2008	3	64	9	63	11.9%	0.33 [0.09, 1.16]
Relax 2020	5	493	13	476	17.3%	0.37 [0.13, 1.03]
**Subtotal (95% CI)**		**583**		**561**	**31.3%**	**0.35 [0.16, 0.76]**
Total events	8		23			
Heterogeneity: Tau² = 0.00; Chi² = 0.03, df = 2 (P = 0.98); I² = 0%						
Test for overall effect: Z = 2.67 (P = 0.008)						
**Total (95% CI)**		**672**		**643**	**100.0%**	**0.50 [0.32, 0.78]**
Total events	28		54			
Heterogeneity: Tau² = 0.04; Chi² = 4.59, df = 4 (P = 0.33); I² = 13%						
Test for overall effect: Z = 3.03 (P = 0.002)						
Test for subgroup differences: Chi² = 0.76, df = 1 (P = 0.38), I² = 0%						

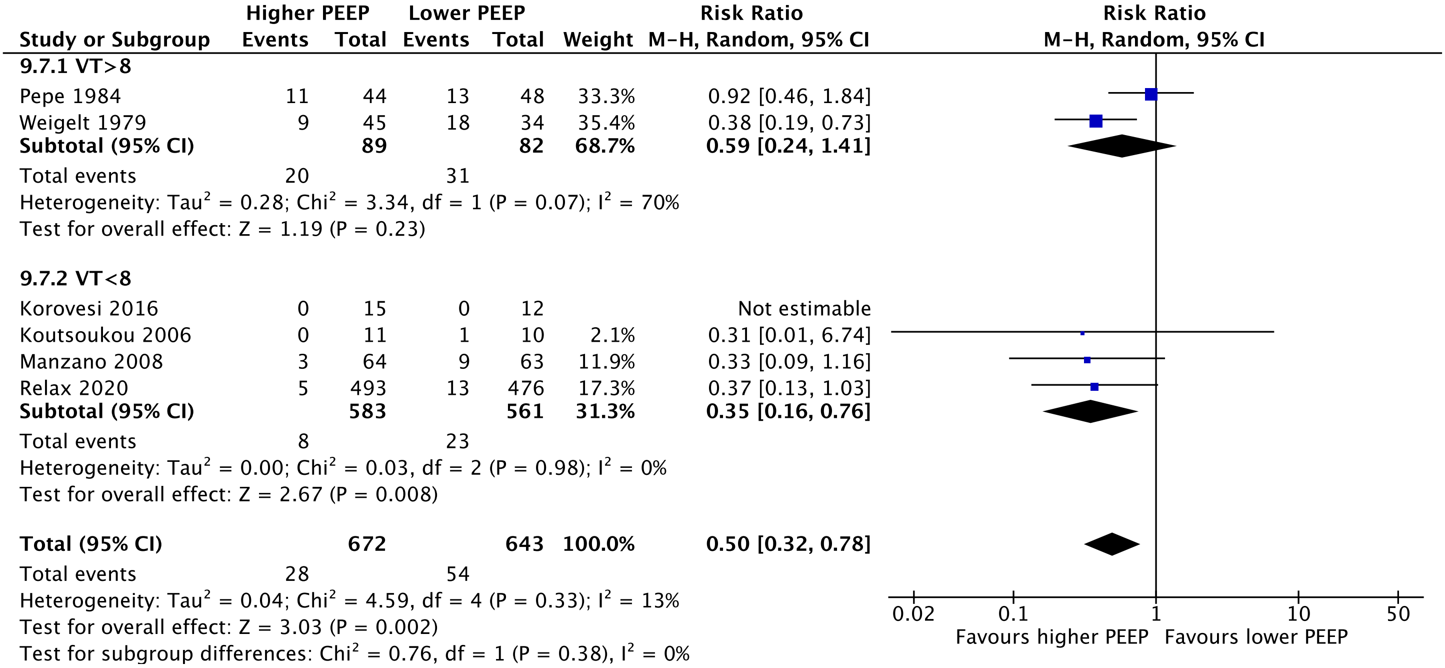

Subgroup analysis (TV > 8 mL/kg vs. TV < 8 mL/kg)

Hypotension

Study or Subgroup	Higher PEEP Events	Higher PEEP Total	Lower PEEP Events	Lower PEEP Total	Weight	Risk Ratio M-H, Random, 95% CI
**9.8.1 VT>8**						
Feeley 1975	0	12	0	13		Not estimable
Good 1979	0	10	0	14		Not estimable
Pepe 1984	1	44	0	48	2.2%	3.27 [0.14, 78.15]
Weigelt 1979	1	45	0	34	2.2%	2.28 [0.10, 54.36]
**Subtotal (95% CI)**		**111**		**109**	**4.5%**	**2.73 [0.29, 25.73]**
Total events	2		0			
Heterogeneity: $Tau^2 = 0.00$; $Chi^2 = 0.02$, df = 1 (P = 0.88); $I^2 = 0\%$						
Test for overall effect: Z = 0.88 (P = 0.38)						
**9.8.2 VT<8**						
Lesur 2010	16	30	16	33	95.5%	1.10 [0.68, 1.79]
**Subtotal (95% CI)**		**30**		**33**	**95.5%**	**1.10 [0.68, 1.79]**
Total events	16		16			
Heterogeneity: Not applicable						
Test for overall effect: Z = 0.38 (P = 0.70)						
**Total (95% CI)**		**141**		**142**	**100.0%**	**1.15 [0.71, 1.84]**
Total events	18		16			
Heterogeneity: $Tau^2 = 0.00$; $Chi^2 = 0.67$, df = 2 (P = 0.72); $I^2 = 0\%$						
Test for overall effect: Z = 0.56 (P = 0.57)						
Test for subgroup differences: $Chi^2 = 0.60$, df = 1 (P = 0.44), $I^2 = 0\%$						

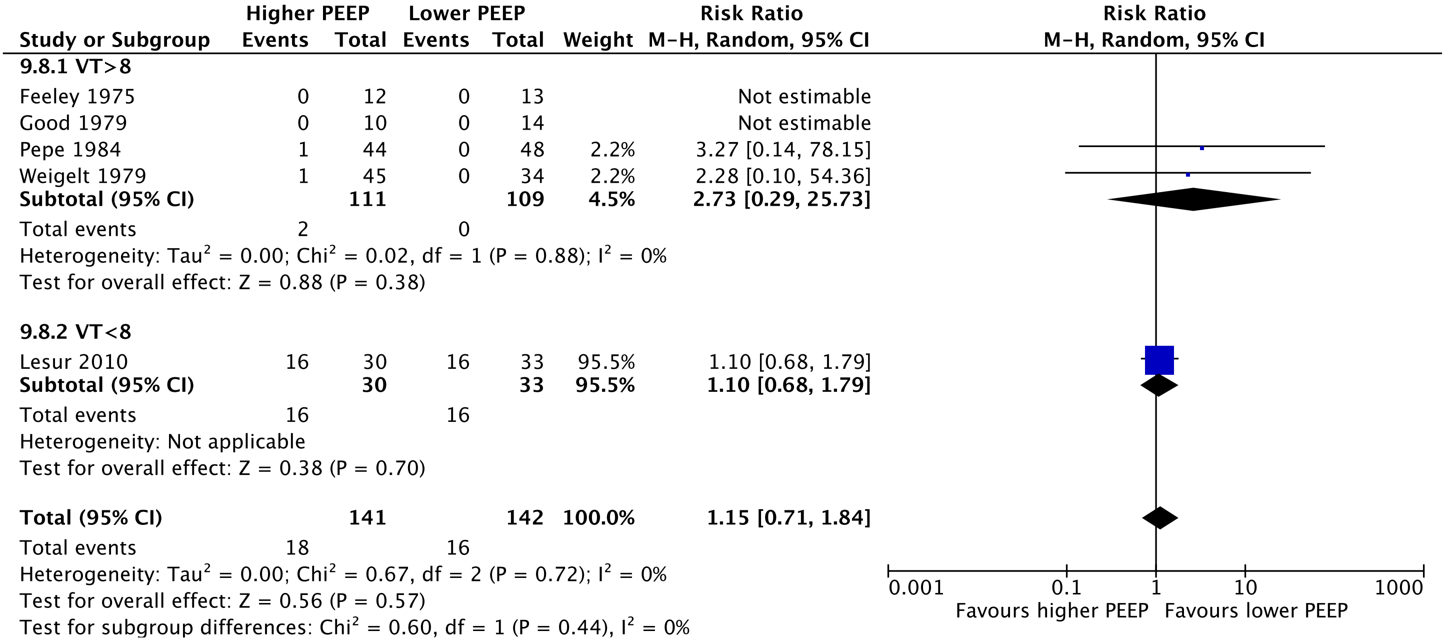

Subgroup analysis (TV > 8 mL/kg vs. TV < 8 mL/kg)

Cardiac index

Study or Subgroup	Higher PEEP Mean	Higher PEEP SD	Higher PEEP Total	Lower PEEP Mean	Lower PEEP SD	Lower PEEP Total	Weight	Mean Difference IV, Random, 95% CI
**9.9.1 VT>8**								
Collier 2002	3.1	0.86	40	3.1	0.64	44	59.6%	0.00 [−0.33, 0.33]
**Subtotal (95% CI)**			**40**			**44**	**59.6%**	**0.00 [−0.33, 0.33]**
Heterogeneity: Not applicable								
Test for overall effect: Z = 0.00 (P = 1.00)								
**9.9.2 VT<8**								
Dyhr 2002	2.2	0.6	7	2.1	1.1	8	8.2%	0.10 [−0.78, 0.98]
Holland 2007	3	0.6	14	2.9	0.6	14	32.2%	0.10 [−0.34, 0.54]
**Subtotal (95% CI)**			**21**			**22**	**40.4%**	**0.10 [−0.30, 0.50]**
Heterogeneity: $Tau^2 = 0.00$; $Chi^2 = 0.00$, df = 1 (P = 1.00); $I^2 = 0\%$								
Test for overall effect: Z = 0.49 (P = 0.62)								
**Total (95% CI)**			**61**			**66**	**100.0%**	**0.04 [−0.21, 0.29]**
Heterogeneity: $Tau^2 = 0.00$; $Chi^2 = 0.15$, df = 2 (P = 0.93); $I^2 = 0\%$								
Test for overall effect: Z = 0.31 (P = 0.75)								
Test for subgroup differences: $Chi^2 = 0.15$, df = 1 (P = 0.70), $I^2 = 0\%$								

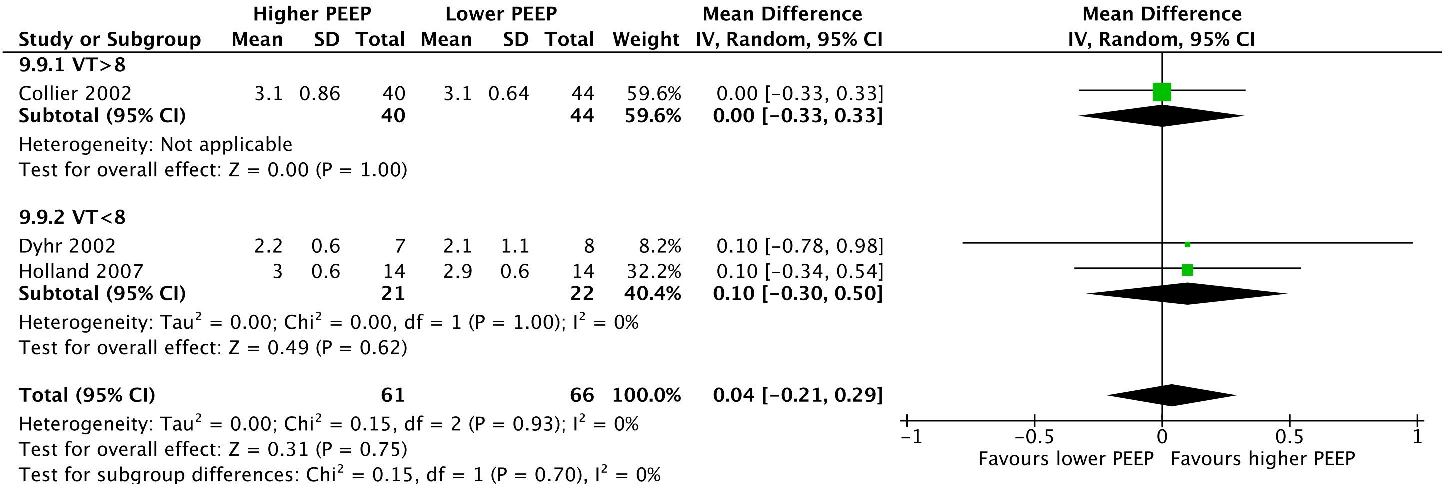

Subgroup analysis (TV > 8 mL/kg vs. TV < 8 mL/kg)

Duration of ventilation

	Higher PEEP			Lower PEEP				Std. Mean Difference
Study or Subgroup	Mean	SD	Total	Mean	SD	Total	Weight	IV, Random, 95% CI
**9.11.1 VT>8**								
Collier 2002	409.1	208.6	40	439.3	277.7	44	13.4%	−0.12 [−0.55, 0.31]
Feeley 1975	258	217	12	259	149	13	6.8%	−0.01 [−0.79, 0.78]
Good 1979	15.7	0.4	10	14.8	0.5	14	4.8%	1.88 [0.88, 2.88]
Marvel 1986	9.3	0.6	12	0	0	0		Not estimable
Weigelt 1979	9.3	13	45	14	21.7	34	12.9%	−0.27 [−0.72, 0.18]
**Subtotal (95% CI)**			**119**			**105**	**37.8%**	**0.25 [−0.44, 0.93]**
Heterogeneity: $Tau^2 = 0.37$; $Chi^2 = 15.33$, df = 3 (P = 0.002); $I^2 = 80\%$								
Test for overall effect: Z = 0.71 (P = 0.48)								
**9.11.2 VT<8**								
Lago Borges 2014	5.1	2.9	45	6.8	3.2	44	13.5%	−0.55 [−0.98, −0.13]
Lesur 2010	9.2	8.8	30	9.2	8.5	33	11.8%	0.00 [−0.49, 0.49]
Manzano 2008	5.8	6.8	64	6.5	6.2	63	15.5%	−0.11 [−0.45, 0.24]
Relax 2020	4.8	6.6	493	5.5	7.4	476	21.4%	−0.10 [−0.23, 0.03]
**Subtotal (95% CI)**			**632**			**616**	**62.2%**	**−0.16 [−0.34, 0.02]**
Heterogeneity: $Tau^2 = 0.01$; $Chi^2 = 4.31$, df = 3 (P = 0.23); $I^2 = 30\%$								
Test for overall effect: Z = 1.70 (P = 0.09)								
**Total (95% CI)**			**751**			**721**	**100.0%**	**−0.07 [−0.32, 0.17]**
Heterogeneity: $Tau^2 = 0.07$; $Chi^2 = 20.24$, df = 7 (P = 0.005); $I^2 = 65\%$								
Test for overall effect: Z = 0.59 (P = 0.55)								
Test for subgroup differences: $Chi^2 = 1.26$, df = 1 (P = 0.26), $I^2 = 20.5\%$								

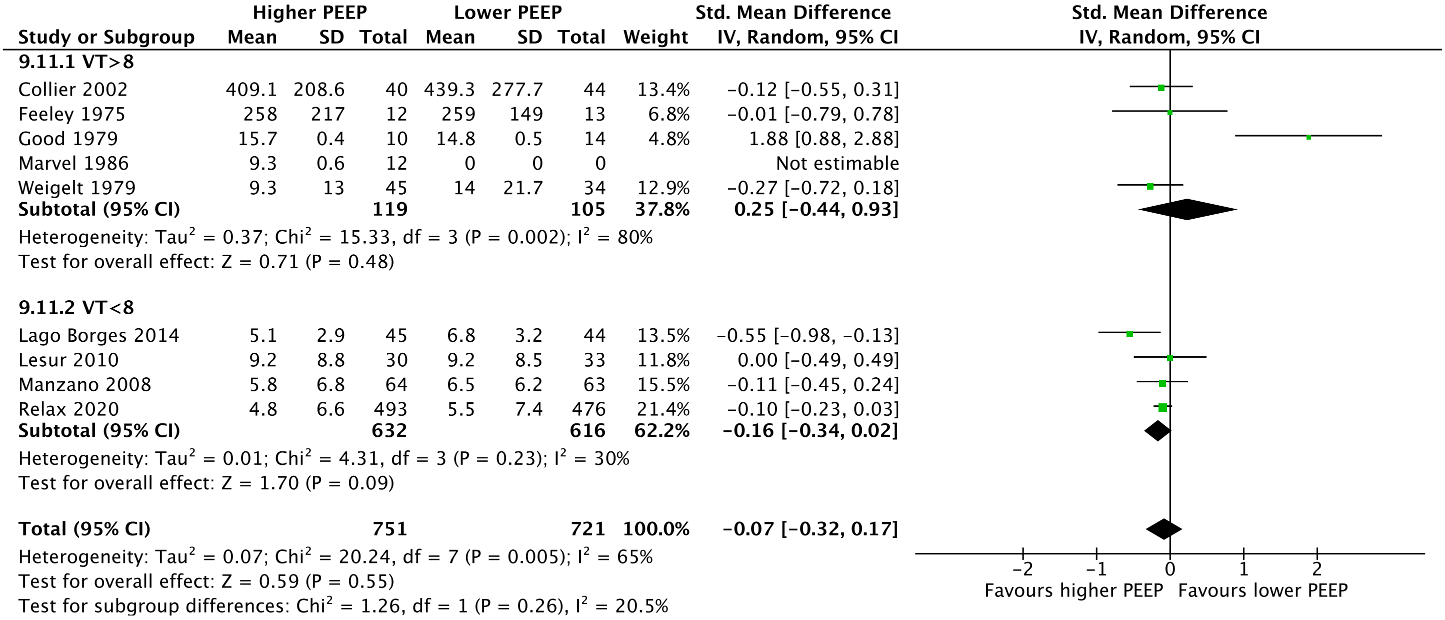

Subgroup analysis (TV > 8 mL/kg vs. TV < 8 mL/kg)

ICU stay

Study or Subgroup	Higher PEEP Mean	Higher PEEP SD	Higher PEEP Total	Lower PEEP Mean	Lower PEEP SD	Lower PEEP Total	Weight	Mean Difference IV, Random, 95% CI
**9.12.1 VT>8**								
Weigelt 1979	11.7	16.8	45	21	32.5	34	1.6%	−9.30 [−21.28, 2.68]
**Subtotal (95% CI)**			**45**			**34**	**1.6%**	**−9.30 [−21.28, 2.68]**
Heterogeneity: Not applicable								
Test for overall effect: Z = 1.52 (P = 0.13)								
**9.12.2 VT<8**								
Korovesi 2011	17.2	10.1	15	14.4	8.44	12	4.6%	2.80 [−4.19, 9.79]
Manzano 2008	10.5	9.8	64	12.3	11.4	63	15.5%	−1.80 [−5.50, 1.90]
Relax 2020	7.2	10.3	493	8.1	11.5	476	78.4%	−0.90 [−2.28, 0.48]
**Subtotal (95% CI)**			**572**			**551**	**98.4%**	**−0.88 [−2.15, 0.38]**
Heterogeneity: $Tau^2 = 0.00$; $Chi^2 = 1.30$, df = 2 (P = 0.52); $I^2 = 0\%$								
Test for overall effect: Z = 1.37 (P = 0.17)								
**Total (95% CI)**			**617**			**585**	**100.0%**	**−1.00 [−2.51, 0.51]**
Heterogeneity: $Tau^2 = 0.26$; $Chi^2 = 3.18$, df = 3 (P = 0.37); $I^2 = 6\%$								
Test for overall effect: Z = 1.30 (P = 0.19)								
Test for subgroup differences: $Chi^2 = 1.88$, df = 1 (P = 0.17), $I^2 = 46.7\%$								

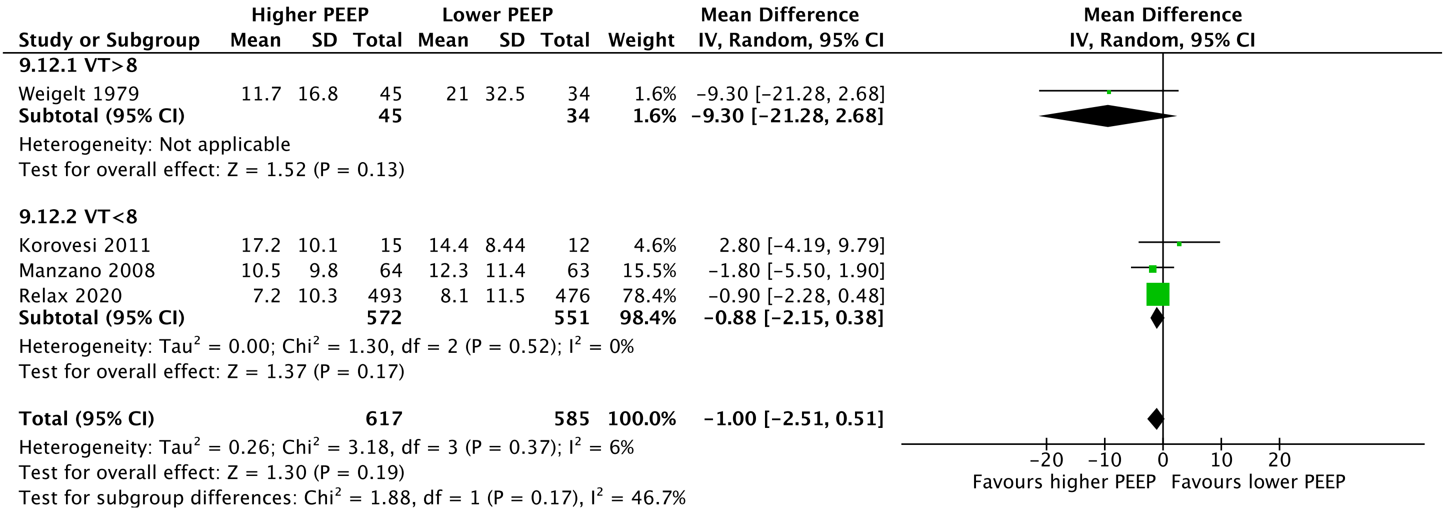

Subgroup analysis (TV > 8 mL/kg vs. TV < 8 mL/kg)

Hospital stay

Study or Subgroup	Higher PEEP Mean	Higher PEEP SD	Higher PEEP Total	Lower PEEP Mean	Lower PEEP SD	Lower PEEP Total	Weight	Mean Difference IV, Random, 95% CI
**9.13.1 VT>8**								
Collier 2002	5.7	2.5	40	5.2	1.7	44	34.5%	0.50 [−0.42, 1.42]
Marvel 1986	8.8	0.5	12	8.9	0.4	15	56.7%	−0.10 [−0.45, 0.25]
**Subtotal (95% CI)**			**52**			**59**	**91.2%**	**0.04 [−0.46, 0.54]**
Heterogeneity: Tau² = 0.05; Chi² = 1.42, df = 1 (P = 0.23); I² = 30%								
Test for overall effect: Z = 0.16 (P = 0.87)								
**9.13.2 VT<8**								
Manzano 2008	19.5	18.2	64	26.3	22	63	1.3%	−6.80 [−13.83, 0.23]
Relax 2020	19	21.4	493	19.9	22.1	476	7.5%	−0.90 [−3.64, 1.84]
**Subtotal (95% CI)**			**557**			**539**	**8.8%**	**−2.93 [−8.42, 2.56]**
Heterogeneity: Tau² = 10.00; Chi² = 2.35, df = 1 (P = 0.13); I² = 57%								
Test for overall effect: Z = 1.04 (P = 0.30)								
**Total (95% CI)**			**609**			**598**	**100.0%**	**−0.04 [−0.84, 0.76]**
Heterogeneity: Tau² = 0.26; Chi² = 5.36, df = 3 (P = 0.15); I² = 44%								
Test for overall effect: Z = 0.10 (P = 0.92)								
Test for subgroup differences: Chi² = 1.11, df = 1 (P = 0.29), I² = 10.1%								

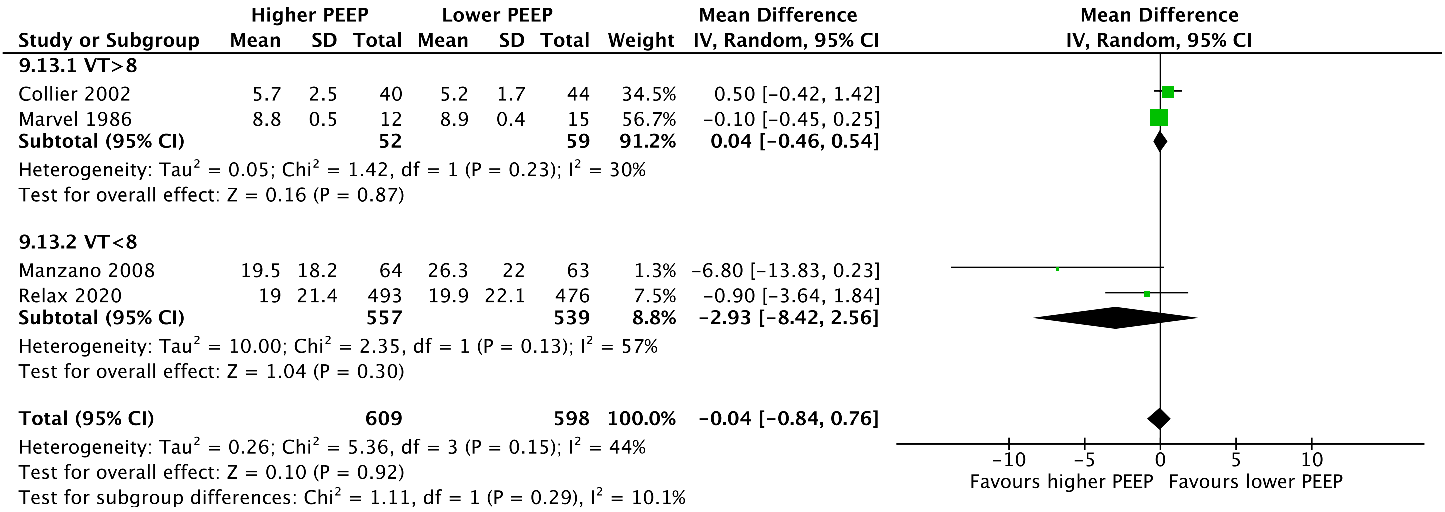

Subgroup analysis (TV > 8 mL/kg vs. TV < 8 mL/kg)

ICU mortality

Study or Subgroup	Higher PEEP Events	Higher PEEP Total	Lower PEEP Events	Lower PEEP Total	Weight	Risk Ratio M-H, Random, 95% CI	Risk Ratio M-H, Random, 95% CI
**9.14.1 VT>8**							
Feeley 1975	2	12	1	13	0.5%	2.17 [0.22, 20.94]	
**Subtotal (95% CI)**		**12**		**13**	**0.5%**	**2.17 [0.22, 20.94]**	
Total events	2		1				
Heterogeneity: Not applicable							
Test for overall effect: Z = 0.67 (P = 0.50)							
**9.14.2 VT<8**							
Dyhr 2002	0	7	0	8		Not estimable	
Korovesi 2011	3	15	4	12	1.7%	0.60 [0.17, 2.18]	
Relax 2020	185	492	163	476	97.8%	1.10 [0.93, 1.30]	
**Subtotal (95% CI)**		**514**		**496**	**99.5%**	**1.09 [0.92, 1.28]**	
Total events	188		167				
Heterogeneity: $Tau^2 = 0.00$; $Chi^2 = 0.83$, df = 1 (P = 0.36); $I^2 = 0\%$							
Test for overall effect: Z = 0.98 (P = 0.33)							
**Total (95% CI)**		**526**		**509**	**100.0%**	**1.09 [0.92, 1.29]**	
Total events	190		168				
Heterogeneity: $Tau^2 = 0.00$; $Chi^2 = 1.18$, df = 2 (P = 0.55); $I^2 = 0\%$							
Test for overall effect: Z = 1.02 (P = 0.31)							
Test for subgroup differences: $Chi^2 = 0.35$, df = 1 (P = 0.55), $I^2 = 0\%$							

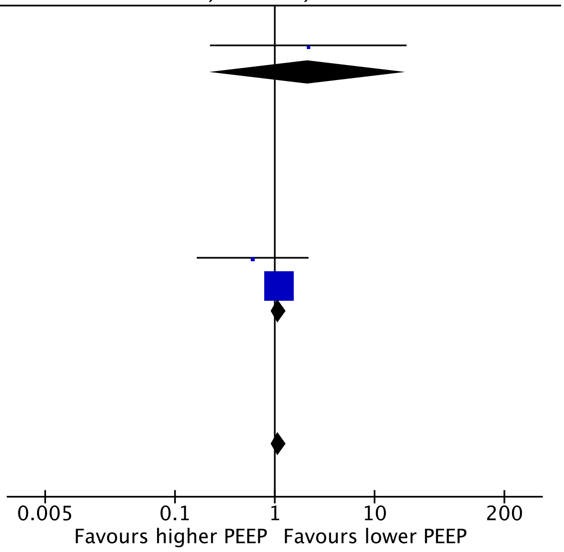

Subgroup analysis (year of publication > 2000 vs. < 2000)

Hospital mortality

Study or Subgroup	Higher PEEP Events	Higher PEEP Total	Lower PEEP Events	Lower PEEP Total	Weight	Risk Ratio M-H, Random, 95% CI
**10.15.1 >2000**						
Collier 2002	1	40	1	44	0.2%	1.10 [0.07, 17.01]
Dyhr 2002	0	7	0	8		Not estimable
Lesur 2010	12	30	16	33	5.5%	0.82 [0.47, 1.45]
Manzano 2008	19	64	16	63	5.5%	1.17 [0.66, 2.06]
Relax 2020	208	489	185	472	75.4%	1.09 [0.93, 1.26]
**Subtotal (95% CI)**		**630**		**620**	**86.6%**	**1.07 [0.93, 1.24]**
Total events	240		218			
Heterogeneity: $Tau^2 = 0.00$; $Chi^2 = 0.95$, df = 3 (P = 0.81); $I^2 = 0\%$						
Test for overall effect: Z = 0.95 (P = 0.34)						
**10.15.2 <2000**						
Michalopoulos 1998	0	21	0	22		Not estimable
Nelson 1987	5	20	6	18	1.7%	0.75 [0.28, 2.04]
Pepe 1984	13	44	18	48	5.1%	0.79 [0.44, 1.41]
Weigelt 1979	16	45	17	34	6.5%	0.71 [0.42, 1.19]
**Subtotal (95% CI)**		**130**		**122**	**13.4%**	**0.74 [0.52, 1.07]**
Total events	34		41			
Heterogeneity: $Tau^2 = 0.00$; $Chi^2 = 0.07$, df = 2 (P = 0.97); $I^2 = 0\%$						
Test for overall effect: Z = 1.60 (P = 0.11)						
**Total (95% CI)**		**760**		**742**	**100.0%**	**1.02 [0.89, 1.16]**
Total events	274		259			
Heterogeneity: $Tau^2 = 0.00$; $Chi^2 = 4.39$, df = 6 (P = 0.62); $I^2 = 0\%$						
Test for overall effect: Z = 0.30 (P = 0.77)						
Test for subgroup differences: $Chi^2 = 3.37$, df = 1 (P = 0.07), $I^2 = 70.3\%$						

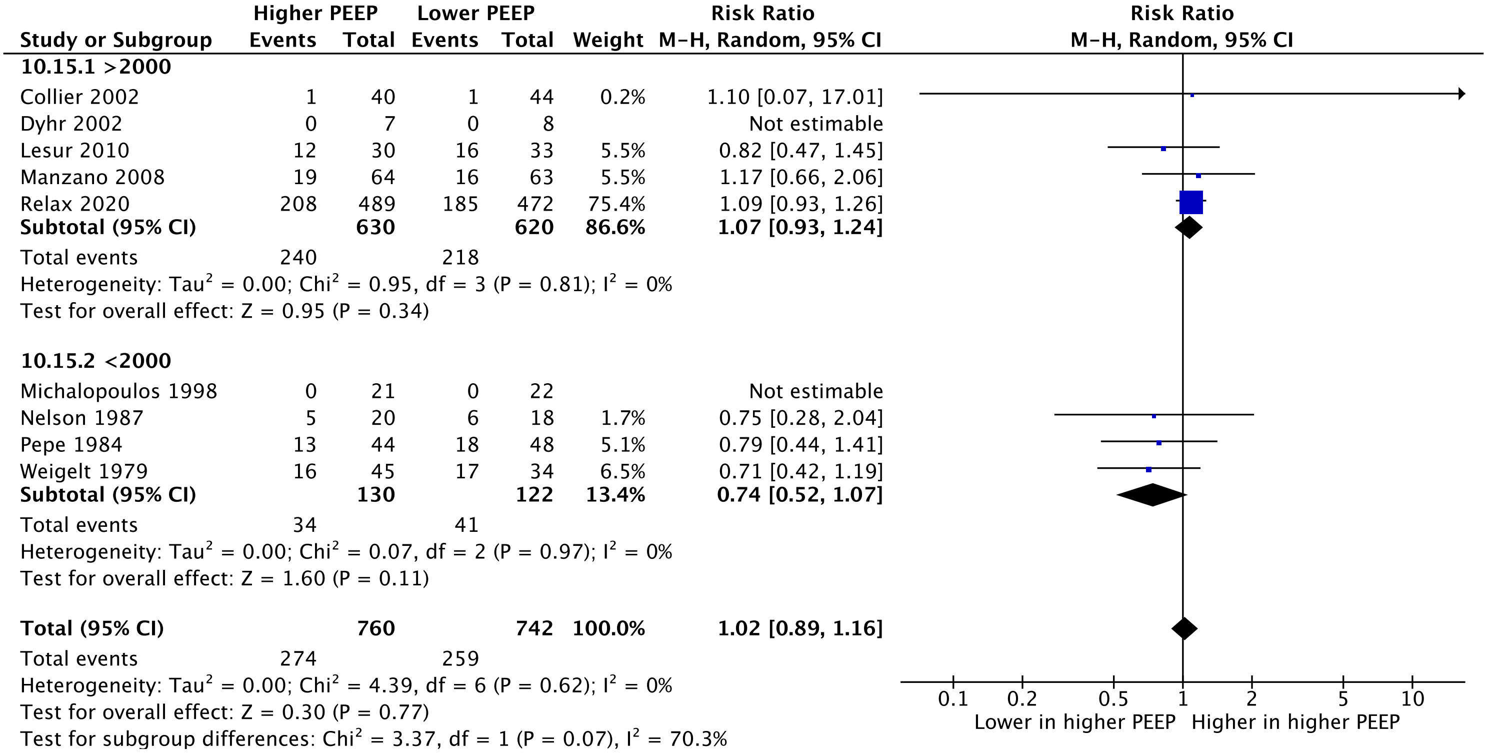

Subgroup analysis (year of publication > 2000 vs. < 2000)

A-aDO2

Study or Subgroup	Higher PEEP Mean	Higher PEEP SD	Higher PEEP Total	Lower PEEP Mean	Lower PEEP SD	Lower PEEP Total	Weight	Std. Mean Difference IV, Random, 95% CI
**10.1.1 >2000**								
Koutsoukou 2006	100	41	11	87	40	10	25.5%	0.31 [−0.55, 1.17]
Lago Borges 2013	117	33	45	139	34	44	27.2%	−0.65 [−1.08, −0.22]
**Subtotal (95% CI)**			**56**			**54**	**52.7%**	**−0.25 [−1.18, 0.68]**
Heterogeneity: $Tau^2 = 0.34$; $Chi^2 = 3.81$, df = 1 (P = 0.05); $I^2 = 74\%$								
Test for overall effect: Z = 0.52 (P = 0.60)								
**10.1.2 <2000**								
Feeley 1975	10	22	12	102	35	13	23.6%	−3.02 [−4.22, −1.81]
Marvel 1986	168	10	12	203	10	17	23.7%	−3.40 [−4.60, −2.20]
**Subtotal (95% CI)**			**24**			**30**	**47.3%**	**−3.21 [−4.06, −2.36]**
Heterogeneity: $Tau^2 = 0.00$; $Chi^2 = 0.20$, df = 1 (P = 0.66); $I^2 = 0\%$								
Test for overall effect: Z = 7.42 (P < 0.00001)								
**Total (95% CI)**			**80**			**84**	**100.0%**	**−1.62 [−3.12, −0.11]**
Heterogeneity: $Tau^2 = 2.13$; $Chi^2 = 37.49$, df = 3 (P < 0.00001); $I^2 = 92\%$								
Test for overall effect: Z = 2.10 (P = 0.04)								
Test for subgroup differences: $Chi^2 = 21.32$, df = 1 (P < 0.00001), $I^2 = 95.3\%$								

Std. Mean Difference
IV, Random, 95% CI

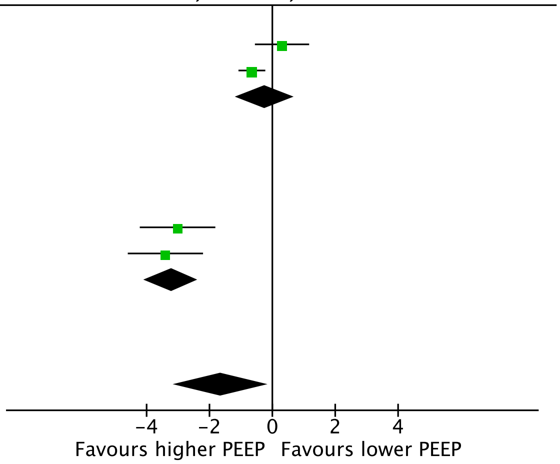

Subgroup analysis (year of publication > 2000 vs. < 2000)

Compliance

Study or Subgroup	Higher PEEP Mean	Higher PEEP SD	Higher PEEP Total	Lower PEEP Mean	Lower PEEP SD	Lower PEEP Total	Weight	Mean Difference IV, Random, 95% CI
**10.2.1 >2000**								
Koutsoukou 2006	62	14	11	53	11	10	24.9%	9.00 [−1.72, 19.72]
Lago Borges 2013	56	19	45	47	12	44	66.1%	9.00 [2.41, 15.59]
**Subtotal (95% CI)**			**56**			**54**	**91.0%**	**9.00 [3.39, 14.61]**
Heterogeneity: $Tau^2 = 0.00$; $Chi^2 = 0.00$, df = 1 (P = 1.00); $I^2 = 0\%$								
Test for overall effect: Z = 3.14 (P = 0.002)								
**10.2.2 <2000**								
Weigelt 1979	42	36	45	39	43	34	9.0%	3.00 [−14.88, 20.88]
**Subtotal (95% CI)**			**45**			**34**	**9.0%**	**3.00 [−14.88, 20.88]**
Heterogeneity: Not applicable								
Test for overall effect: Z = 0.33 (P = 0.74)								
**Total (95% CI)**			**101**			**88**	**100.0%**	**8.46 [3.11, 13.82]**
Heterogeneity: $Tau^2 = 0.00$; $Chi^2 = 0.39$, df = 2 (P = 0.82); $I^2 = 0\%$								
Test for overall effect: Z = 3.10 (P = 0.002)								
Test for subgroup differences: $Chi^2 = 0.39$, df = 1 (P = 0.53), $I^2 = 0\%$								

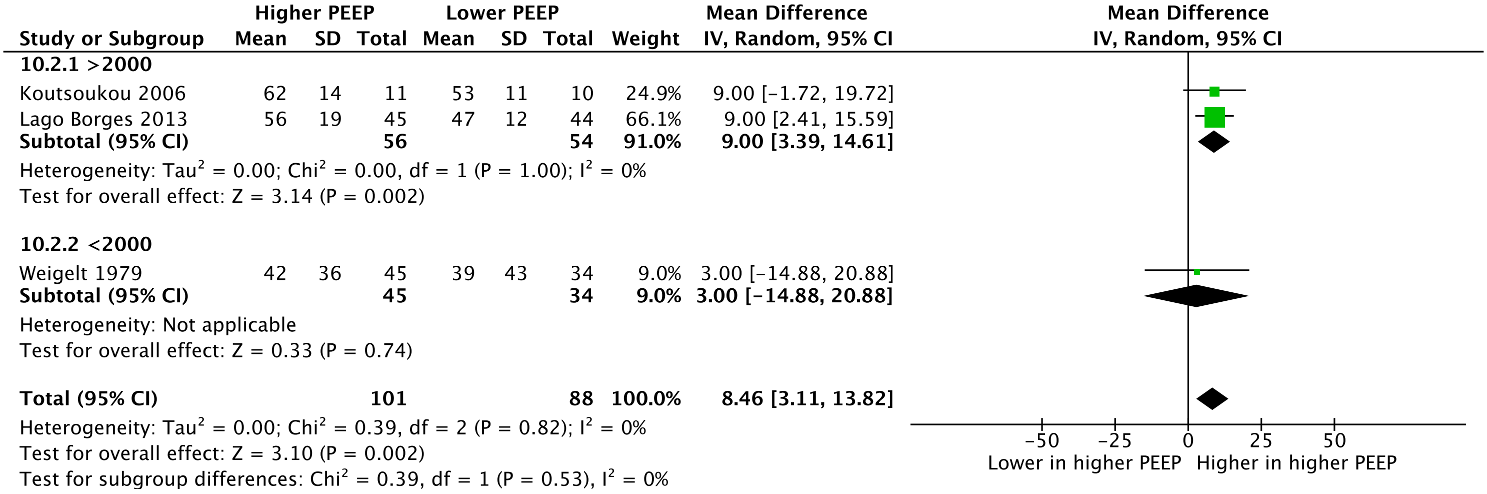

Subgroup analysis (year of publication > 2000 vs. < 2000)

Hypoxemia

Study or Subgroup	Higher PEEP Events	Higher PEEP Total	Lower PEEP Events	Lower PEEP Total	Weight	Risk Ratio M–H, Random, 95% CI
**10.3.1 >2000**						
Lago Borges 2013	19	45	30	44	30.9%	0.62 [0.42, 0.92]
Manzano 2008	12	64	34	63	24.3%	0.35 [0.20, 0.61]
Relax 2020	87	493	98	476	36.9%	0.86 [0.66, 1.11]
**Subtotal (95% CI)**		**602**		**583**	**92.1%**	**0.60 [0.37, 0.96]**
Total events	118		162			
Heterogeneity: $Tau^2 = 0.13$; $Chi^2 = 8.75$, df = 2 (P = 0.01); $I^2 = 77\%$						
Test for overall effect: Z = 2.11 (P = 0.03)						
**10.3.2 <2000**						
Michalopoulos 1998	2	21	2	22	4.5%	1.05 [0.16, 6.77]
Pepe 1984	1	44	4	48	3.4%	0.27 [0.03, 2.35]
**Subtotal (95% CI)**		**65**		**70**	**7.9%**	**0.59 [0.14, 2.41]**
Total events	3		6			
Heterogeneity: $Tau^2 = 0.00$; $Chi^2 = 0.87$, df = 1 (P = 0.35); $I^2 = 0\%$						
Test for overall effect: Z = 0.74 (P = 0.46)						
**Total (95% CI)**		**667**		**653**	**100.0%**	**0.60 [0.40, 0.92]**
Total events	121		168			
Heterogeneity: $Tau^2 = 0.11$; $Chi^2 = 9.66$, df = 4 (P = 0.05); $I^2 = 59\%$						
Test for overall effect: Z = 2.37 (P = 0.02)						
Test for subgroup differences: $Chi^2 = 0.00$, df = 1 (P = 0.98), $I^2 = 0\%$						

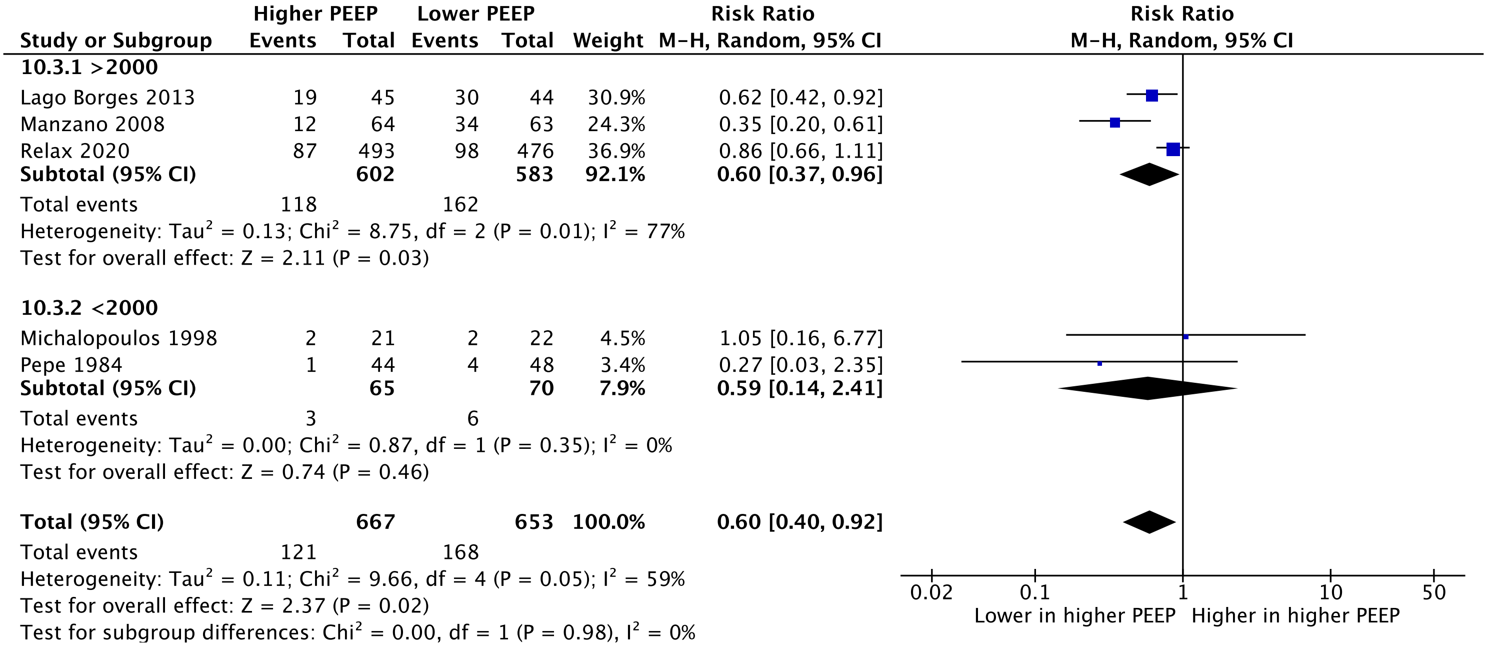

Subgroup analysis (year of publication > 2000 vs. < 2000)

Atelectasis

Study or Subgroup	Higher PEEP Events	Higher PEEP Total	Lower PEEP Events	Lower PEEP Total	Weight	Risk Ratio M-H, Random, 95% CI
**10.4.1 >2000**						
Manzano 2008	12	64	17	63	11.8%	0.69 [0.36, 1.33]
Relax 2020	15	493	20	476	11.6%	0.72 [0.38, 1.40]
**Subtotal (95% CI)**		**557**		**539**	**23.4%**	**0.71 [0.45, 1.13]**
Total events	27		37			
Heterogeneity: Tau² = 0.00; Chi² = 0.01, df = 1 (P = 0.93); I² = 0%						
Test for overall effect: Z = 1.45 (P = 0.15)						
**10.4.2 <2000**						
Good 1979	9	10	12	14	44.3%	1.05 [0.78, 1.41]
Michalopoulos 1998	2	21	2	22	1.5%	1.05 [0.16, 6.77]
Pepe 1984	27	44	23	48	30.8%	1.28 [0.88, 1.87]
**Subtotal (95% CI)**		**75**		**84**	**76.6%**	**1.13 [0.90, 1.43]**
Total events	38		37			
Heterogeneity: Tau² = 0.00; Chi² = 0.90, df = 2 (P = 0.64); I² = 0%						
Test for overall effect: Z = 1.05 (P = 0.29)						
**Total (95% CI)**		**632**		**623**	**100.0%**	**1.02 [0.81, 1.28]**
Total events	65		74			
Heterogeneity: Tau² = 0.01; Chi² = 4.51, df = 4 (P = 0.34); I² = 11%						
Test for overall effect: Z = 0.15 (P = 0.88)						
Test for subgroup differences: Chi² = 3.13, df = 1 (P = 0.08), I² = 68.1%						

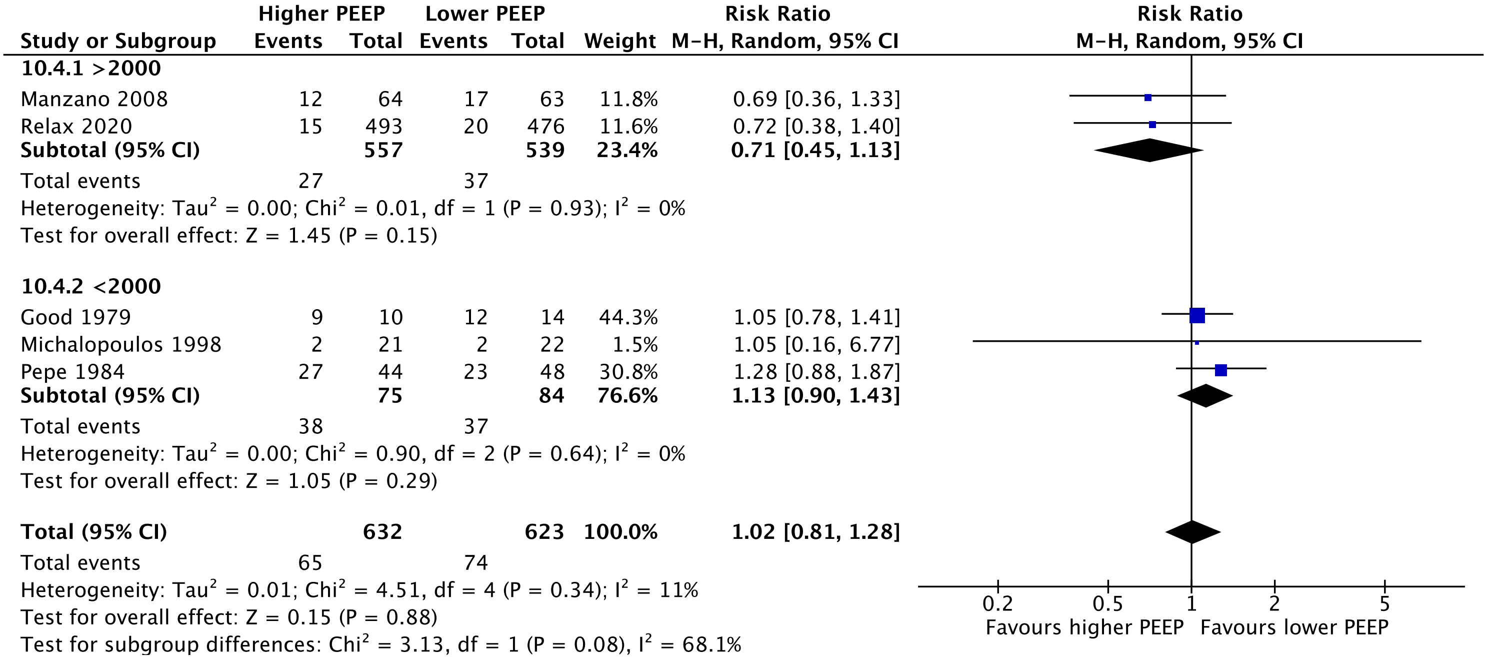

Subgroup analysis (year of publication > 2000 vs. < 2000)

Barotrauma

Study or Subgroup	Higher PEEP Events	Higher PEEP Total	Lower PEEP Events	Lower PEEP Total	Weight	Risk Ratio M-H, Random, 95% CI
**10.5.1 >2000**						
Manzano 2008	1	64	5	63	2.8%	0.20 [0.02, 1.64]
Relax 2020	12	493	19	476	24.4%	0.61 [0.30, 1.24]
**Subtotal (95% CI)**		**557**		**539**	**27.2%**	**0.54 [0.28, 1.07]**
Total events	13		24			
Heterogeneity: $Tau^2 = 0.00; Chi^2 = 0.99, df = 1 (P = 0.32); I^2 = 0\%$						
Test for overall effect: Z = 1.77 (P = 0.08)						
**10.5.2 <2000**						
Good 1979	0	10	0	14		Not estimable
Michalopoulos 1998	0	21	0	22		Not estimable
Nelson 1987	1	20	0	18	1.3%	2.71 [0.12, 62.70]
Pepe 1984	19	44	24	48	63.5%	0.86 [0.56, 1.34]
Weigelt 1979	5	45	4	34	8.1%	0.94 [0.27, 3.25]
**Subtotal (95% CI)**		**140**		**136**	**72.8%**	**0.89 [0.59, 1.34]**
Total events	25		28			
Heterogeneity: $Tau^2 = 0.00; Chi^2 = 0.52, df = 2 (P = 0.77); I^2 = 0\%$						
Test for overall effect: Z = 0.56 (P = 0.58)						
**Total (95% CI)**		**697**		**675**	**100.0%**	**0.78 [0.55, 1.11]**
Total events	38		52			
Heterogeneity: $Tau^2 = 0.00; Chi^2 = 3.10, df = 4 (P = 0.54); I^2 = 0\%$						
Test for overall effect: Z = 1.40 (P = 0.16)						
Test for subgroup differences: $Chi^2 = 1.49, df = 1 (P = 0.22), I^2 = 32.9\%$						

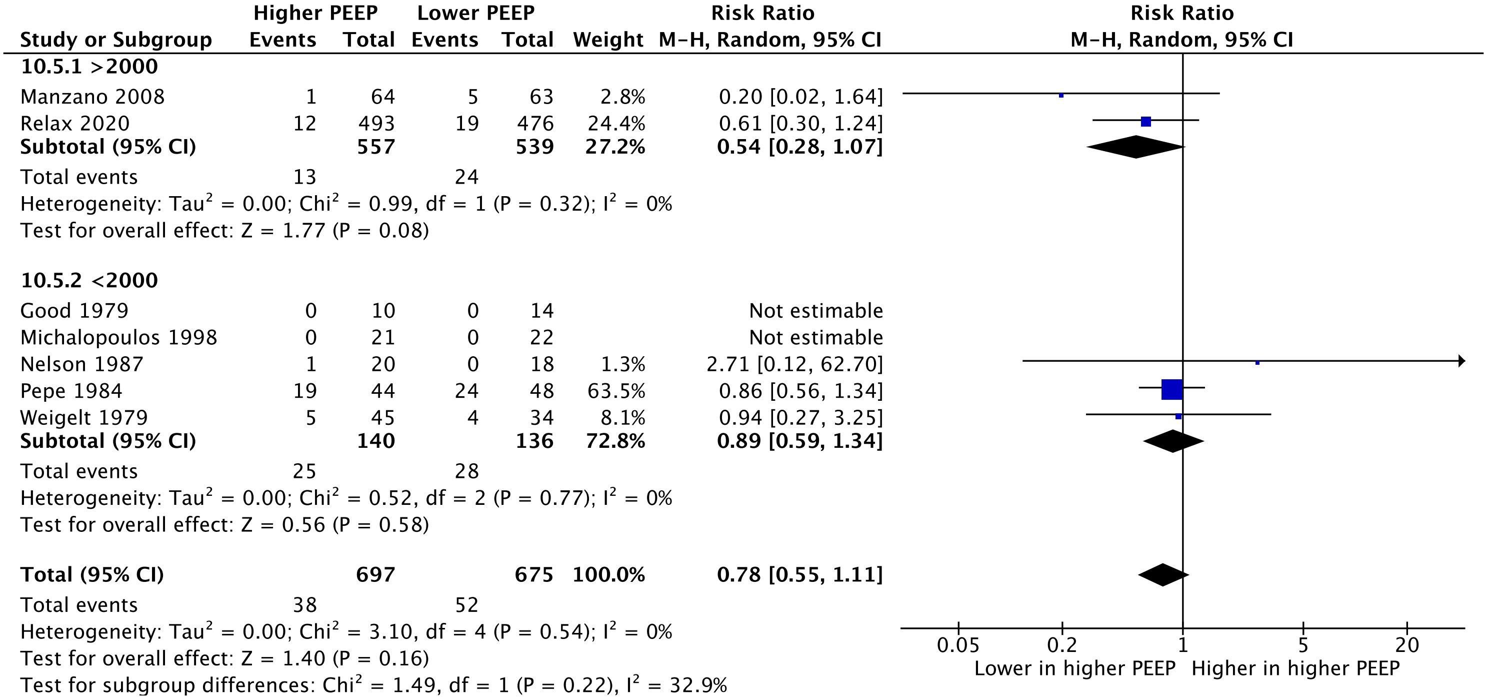

Subgroup analysis (year of publication > 2000 vs. < 2000)

Ventilator-associated pneumonia

Study or Subgroup	Higher PEEP Events	Higher PEEP Total	Lower PEEP Events	Lower PEEP Total	Weight	Risk Ratio M-H, Random, 95% CI
**10.6.1 >2000**						
Manzano 2008	6	64	16	63	42.8%	0.37 [0.15, 0.88]
Relax 2020	7	493	6	476	30.9%	1.13 [0.38, 3.33]
**Subtotal (95% CI)**		**557**		**539**	**73.7%**	**0.61 [0.21, 1.83]**
Total events	13		22			
Heterogeneity: $Tau^2 = 0.37$; $Chi^2 = 2.47$, df = 1 (P = 0.12); $I^2 = 60\%$						
Test for overall effect: Z = 0.88 (P = 0.38)						
**10.6.2 <2000**						
Pepe 1984	4	44	6	48	26.3%	0.73 [0.22, 2.41]
**Subtotal (95% CI)**		**44**		**48**	**26.3%**	**0.73 [0.22, 2.41]**
Total events	4		6			
Heterogeneity: Not applicable						
Test for overall effect: Z = 0.52 (P = 0.60)						
**Total (95% CI)**		**601**		**587**	**100.0%**	**0.62 [0.32, 1.23]**
Total events	17		28			
Heterogeneity: $Tau^2 = 0.08$; $Chi^2 = 2.59$, df = 2 (P = 0.27); $I^2 = 23\%$						
Test for overall effect: Z = 1.36 (P = 0.17)						
Test for subgroup differences: $Chi^2 = 0.04$, df = 1 (P = 0.84), $I^2 = 0\%$						

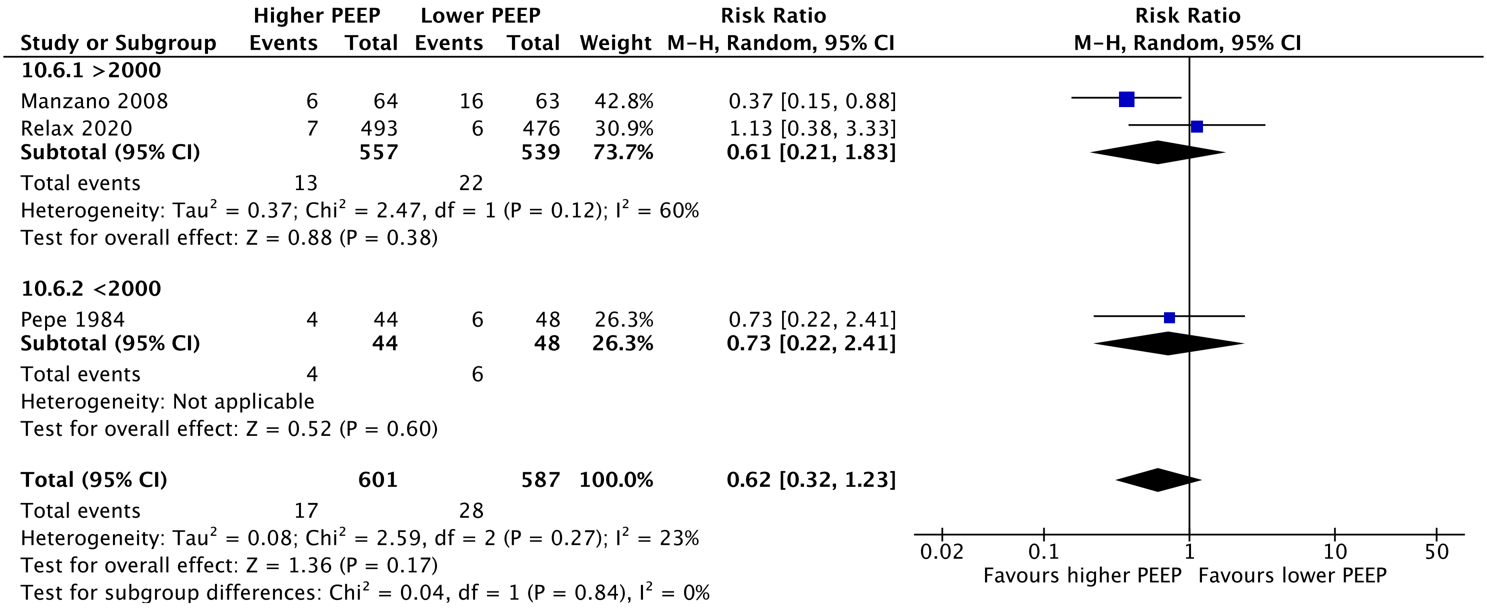

Subgroup analysis (year of publication > 2000 vs. < 2000)

ARDS

	Higher PEEP		Lower PEEP			Risk Ratio
**Study or Subgroup**	**Events**	**Total**	**Events**	**Total**	**Weight**	**M-H, Random, 95% CI**
**10.7.1 >2000**						
Korovesi 2016	0	15	0	12		Not estimable
Koutsoukou 2006	0	11	1	10	2.1%	0.31 [0.01, 6.74]
Manzano 2008	3	64	9	63	11.9%	0.33 [0.09, 1.16]
Relax 2020	5	493	13	476	17.3%	0.37 [0.13, 1.03]
**Subtotal (95% CI)**		**583**		**561**	**31.3%**	**0.35 [0.16, 0.76]**
Total events	8		23			
Heterogeneity: Tau² = 0.00; Chi² = 0.03, df = 2 (P = 0.98); I² = 0%						
Test for overall effect: Z = 2.67 (P = 0.008)						
**10.7.2 <2000**						
Pepe 1984	11	44	13	48	33.3%	0.92 [0.46, 1.84]
Weigelt 1979	9	45	18	34	35.4%	0.38 [0.19, 0.73]
**Subtotal (95% CI)**		**89**		**82**	**68.7%**	**0.59 [0.24, 1.41]**
Total events	20		31			
Heterogeneity: Tau² = 0.28; Chi² = 3.34, df = 1 (P = 0.07); I² = 70%						
Test for overall effect: Z = 1.19 (P = 0.23)						
**Total (95% CI)**		**672**		**643**	**100.0%**	**0.50 [0.32, 0.78]**
Total events	28		54			
Heterogeneity: Tau² = 0.04; Chi² = 4.59, df = 4 (P = 0.33); I² = 13%						
Test for overall effect: Z = 3.03 (P = 0.002)						
Test for subgroup differences: Chi² = 0.76, df = 1 (P = 0.38), I² = 0%						

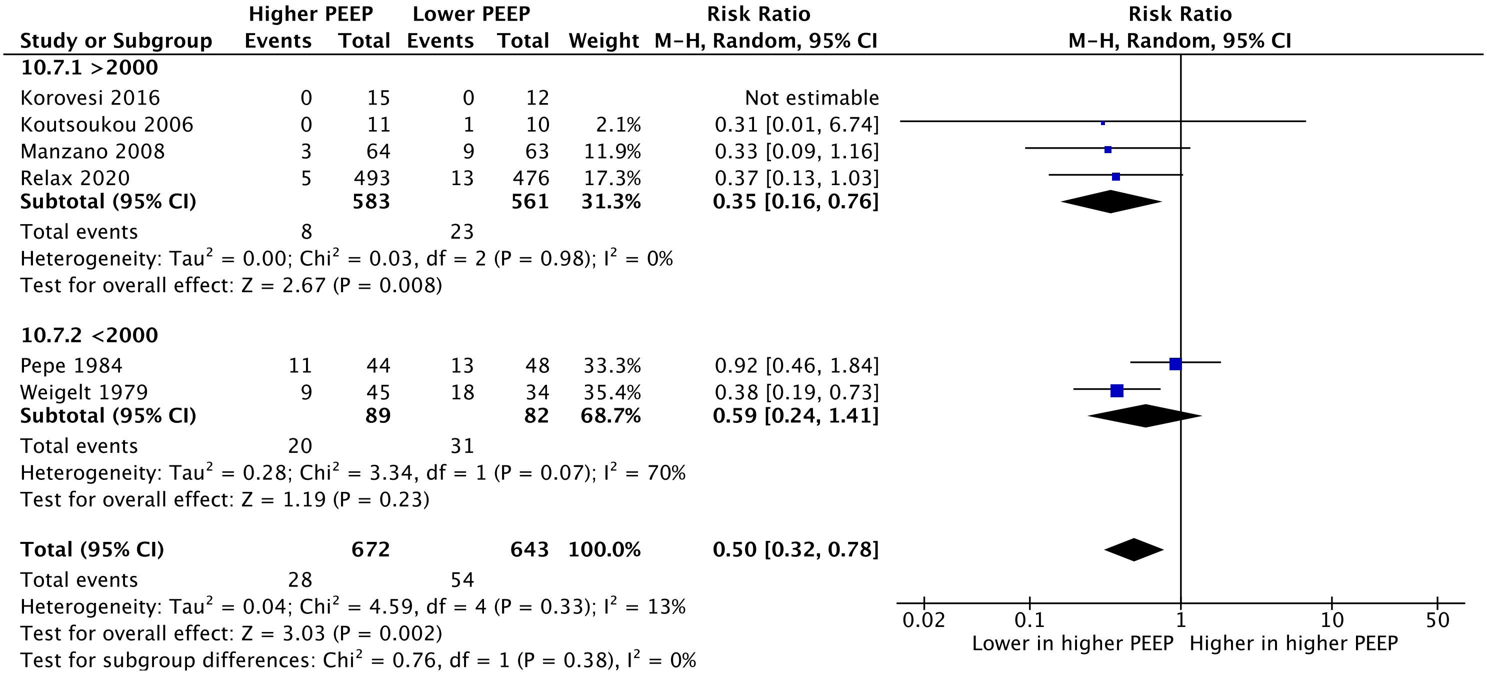

Subgroup analysis (year of publication > 2000 vs. < 2000)

Hypotension

Study or Subgroup	Higher PEEP Events	Higher PEEP Total	Lower PEEP Events	Lower PEEP Total	Weight	Risk Ratio M-H, Random, 95% CI
**10.8.1 >2000**						
Lesur 2010	16	30	16	33	95.5%	1.10 [0.68, 1.79]
**Subtotal (95% CI)**		**30**		**33**	**95.5%**	**1.10 [0.68, 1.79]**
Total events	16		16			
Heterogeneity: Not applicable						
Test for overall effect: Z = 0.38 (P = 0.70)						
**10.8.2 <2000**						
Feeley 1975	0	12	0	13		Not estimable
Good 1979	0	10	0	14		Not estimable
Pepe 1984	1	44	0	48	2.2%	3.27 [0.14, 78.15]
Weigelt 1979	1	45	0	34	2.2%	2.28 [0.10, 54.36]
**Subtotal (95% CI)**		**111**		**109**	**4.5%**	**2.73 [0.29, 25.73]**
Total events	2		0			
Heterogeneity: $Tau^2 = 0.00$; $Chi^2 = 0.02$, df = 1 (P = 0.88); $I^2 = 0\%$						
Test for overall effect: Z = 0.88 (P = 0.38)						
**Total (95% CI)**		**141**		**142**	**100.0%**	**1.15 [0.71, 1.84]**
Total events	18		16			
Heterogeneity: $Tau^2 = 0.00$; $Chi^2 = 0.67$, df = 2 (P = 0.72); $I^2 = 0\%$						
Test for overall effect: Z = 0.56 (P = 0.57)						
Test for subgroup differences: $Chi^2 = 0.60$, df = 1 (P = 0.44), $I^2 = 0\%$						

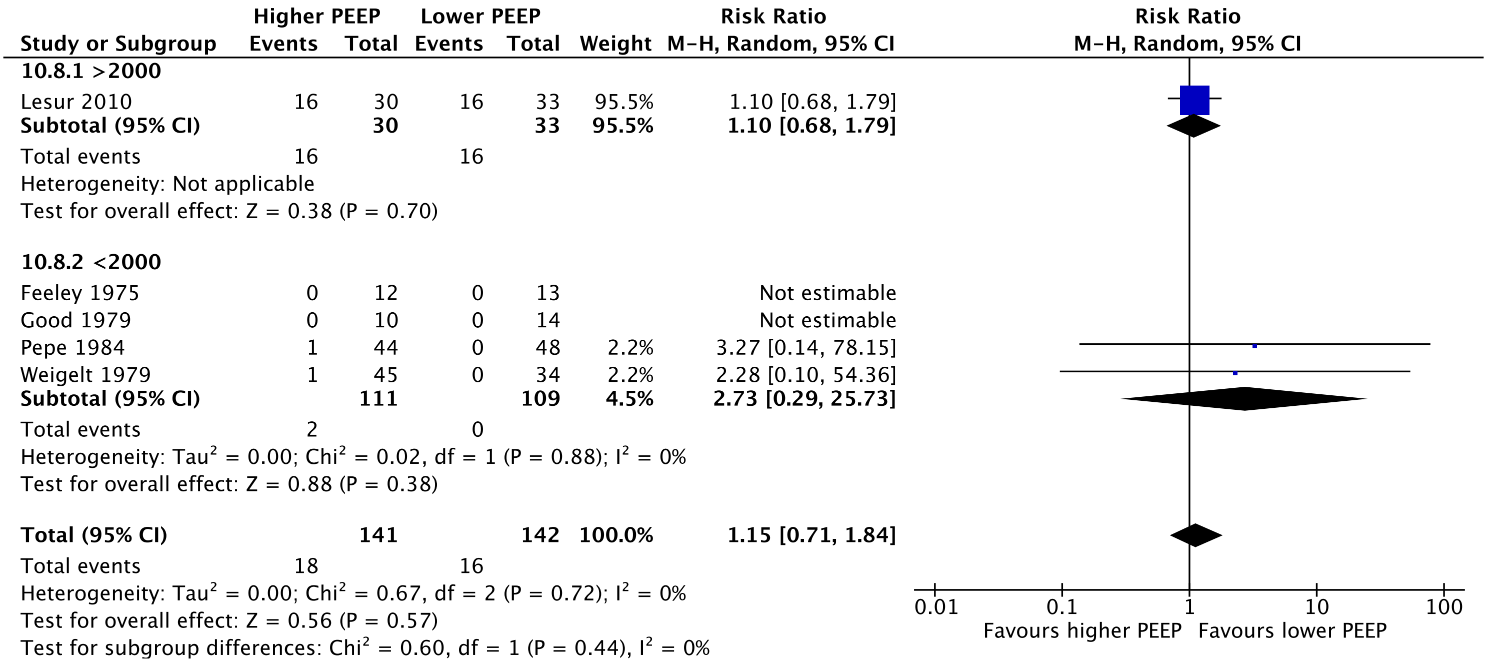

Subgroup analysis (year of publication > 2000 vs. < 2000)

Postoperative bleeding

	Higher PEEP			Lower PEEP				Mean Difference
**Study or Subgroup**	**Mean**	**SD**	**Total**	**Mean**	**SD**	**Total**	**Weight**	**IV, Random, 95% CI**
**10.9.1 >2000**								
Collier 2002	703	395	40	587	392	44	34.2%	116.00 [-52.52, 284.52]
**Subtotal (95% CI)**			**40**			**44**	**34.2%**	**116.00 [-52.52, 284.52]**
Heterogeneity: Not applicable								
Test for overall effect: Z = 1.35 (P = 0.18)								
**10.9.2 <2000**								
Zurich 1982	542	239	41	562	261	476	65.8%	-20.00 [-96.82, 56.82]
**Subtotal (95% CI)**			**41**			**476**	**65.8%**	**-20.00 [-96.82, 56.82]**
Heterogeneity: Not applicable								
Test for overall effect: Z = 0.51 (P = 0.61)								
**Total (95% CI)**			**81**			**520**	**100.0%**	**26.47 [-99.95, 152.89]**

Heterogeneity: $Tau^2 = 4783.35$; $Chi^2 = 2.07$, df = 1 (P = 0.15); $I^2 = 52\%$
Test for overall effect: Z = 0.41 (P = 0.68)
Test for subgroup differences: $Chi^2 = 2.07$, df = 1 (P = 0.15), $I^2 = 51.7\%$

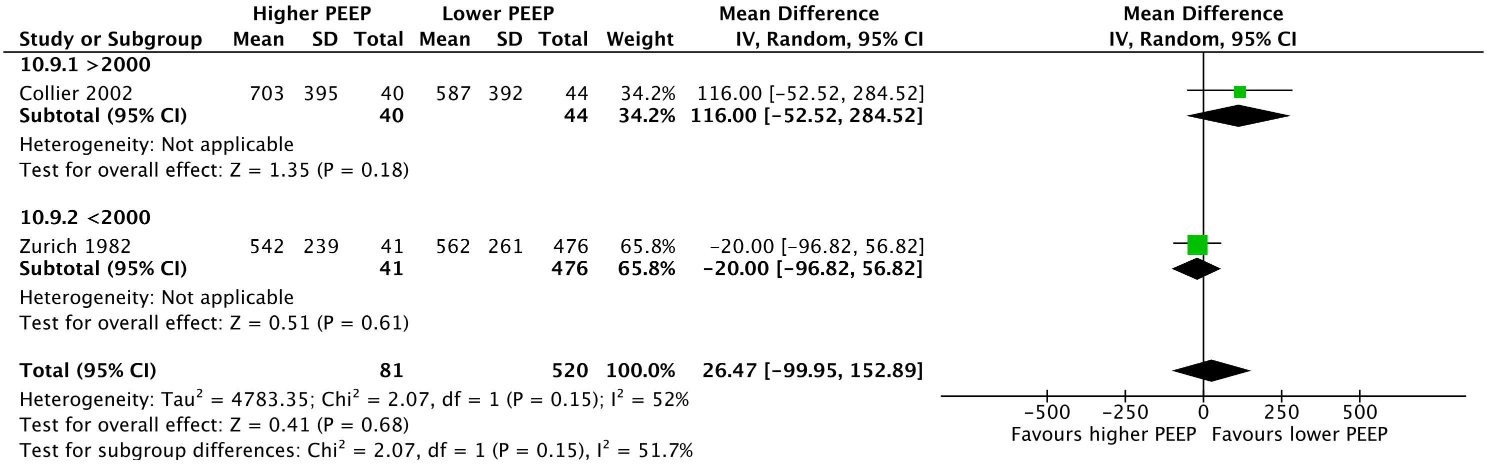

Subgroup analysis (year of publication > 2000 vs. < 2000)

Packed red blood cell transfusion

Study or Subgroup	Higher PEEP Mean	Higher PEEP SD	Higher PEEP Total	Lower PEEP Mean	Lower PEEP SD	Lower PEEP Total	Weight	Mean Difference IV, Random, 95% CI
**10.10.1 >2000**								
Collier 2002	0.8	1.4	40	1.1	1.6	44	37.5%	−0.30 [−0.94, 0.34]
Relax 2020	1.7	0.7	493	1	0	476		Not estimable
**Subtotal (95% CI)**			**533**			**520**	**37.5%**	**−0.30 [−0.94, 0.34]**
Heterogeneity: Not applicable								
Test for overall effect: Z = 0.92 (P = 0.36)								
**10.10.2 <2000**								
Zurich 1982	0.33	0.87	41	0.75	1.42	44	62.5%	−0.42 [−0.92, 0.08]
**Subtotal (95% CI)**			**41**			**44**	**62.5%**	**−0.42 [−0.92, 0.08]**
Heterogeneity: Not applicable								
Test for overall effect: Z = 1.66 (P = 0.10)								
**Total (95% CI)**			**574**			**564**	**100.0%**	**−0.38 [−0.77, 0.02]**

Heterogeneity: $Tau^2 = 0.00$; $Chi^2 = 0.08$, df = 1 (P = 0.77); $I^2 = 0\%$
Test for overall effect: Z = 1.87 (P = 0.06)
Test for subgroup differences: $Chi^2 = 0.08$, df = 1 (P = 0.77), $I^2 = 0\%$

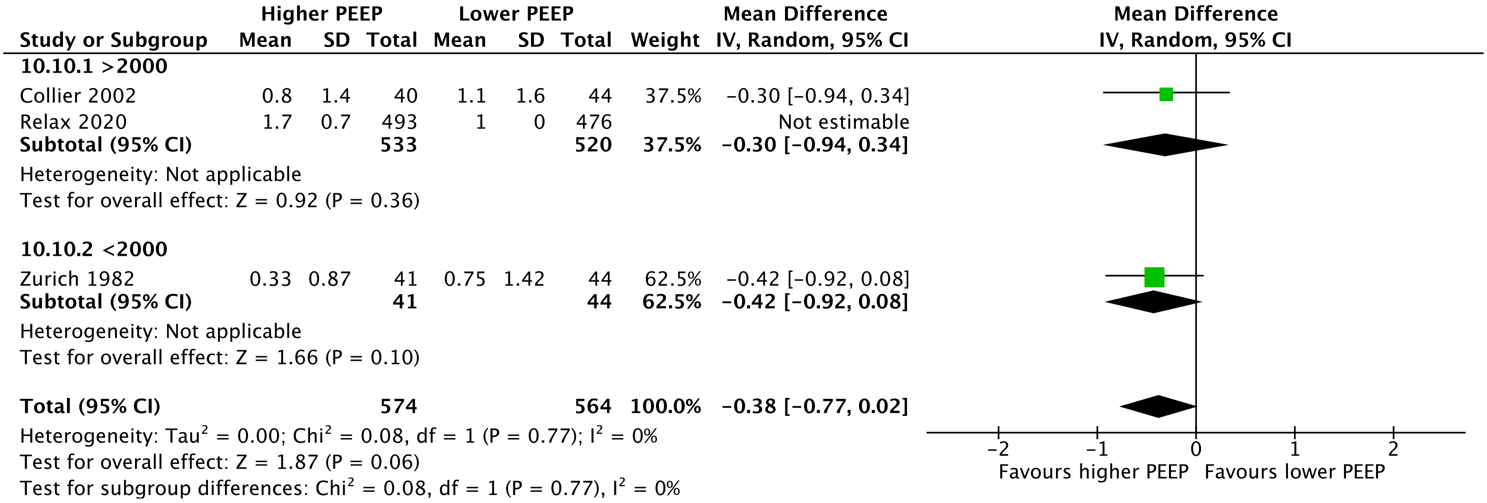

Subgroup analysis (year of publication > 2000 vs. < 2000)

Duration of ventilation

Study or Subgroup	Higher PEEP Mean	Higher PEEP SD	Higher PEEP Total	Lower PEEP Mean	Lower PEEP SD	Lower PEEP Total	Weight	Std. Mean Difference IV, Random, 95% CI
**10.11.1 >2000**								
Collier 2002	409.1	208.6	40	439.3	277.7	44	12.3%	−0.12 [−0.55, 0.31]
Lago Borges 2014	5.1	2.9	45	6.8	3.2	44	12.4%	−0.55 [−0.98, −0.13]
Lesur 2010	9.2	8.8	30	9.2	8.5	33	10.9%	0.00 [−0.49, 0.49]
Manzano 2008	5.8	6.8	64	6.5	6.2	63	14.2%	−0.11 [−0.45, 0.24]
Relax 2020	4.8	6.6	493	5.5	7.4	476	19.2%	−0.10 [−0.23, 0.03]
**Subtotal (95% CI)**			**672**			**660**	**69.1%**	**−0.13 [−0.26, −0.01]**
Heterogeneity: $Tau^2 = 0.00$; $Chi^2 = 4.31$, df = 4 (P = 0.37); $I^2 = 7\%$								
Test for overall effect: Z = 2.10 (P = 0.04)								
**10.11.2 <2000**								
Feeley 1975	258	217	12	259	149	13	6.4%	−0.01 [−0.79, 0.78]
Good 1979	15.7	0.4	10	14.8	0.5	14	4.5%	1.88 [0.88, 2.88]
Marvel 1986	9.3	0.6	12	0	0	0		Not estimable
Nelson 1987	5.3	5	20	3.4	3	18	8.2%	0.45 [−0.20, 1.09]
Weigelt 1979	9.3	13	45	14	21.7	34	11.9%	−0.27 [−0.72, 0.18]
**Subtotal (95% CI)**			**99**			**79**	**30.9%**	**0.43 [−0.35, 1.22]**
Heterogeneity: $Tau^2 = 0.51$; $Chi^2 = 15.88$, df = 3 (P = 0.001); $I^2 = 81\%$								
Test for overall effect: Z = 1.08 (P = 0.28)								
**Total (95% CI)**			**771**			**739**	**100.0%**	**−0.03 [−0.27, 0.21]**
Heterogeneity: $Tau^2 = 0.07$; $Chi^2 = 23.01$, df = 8 (P = 0.003); $I^2 = 65\%$								
Test for overall effect: Z = 0.24 (P = 0.81)								
Test for subgroup differences: $Chi^2 = 1.94$, df = 1 (P = 0.16), $I^2 = 48.5\%$								

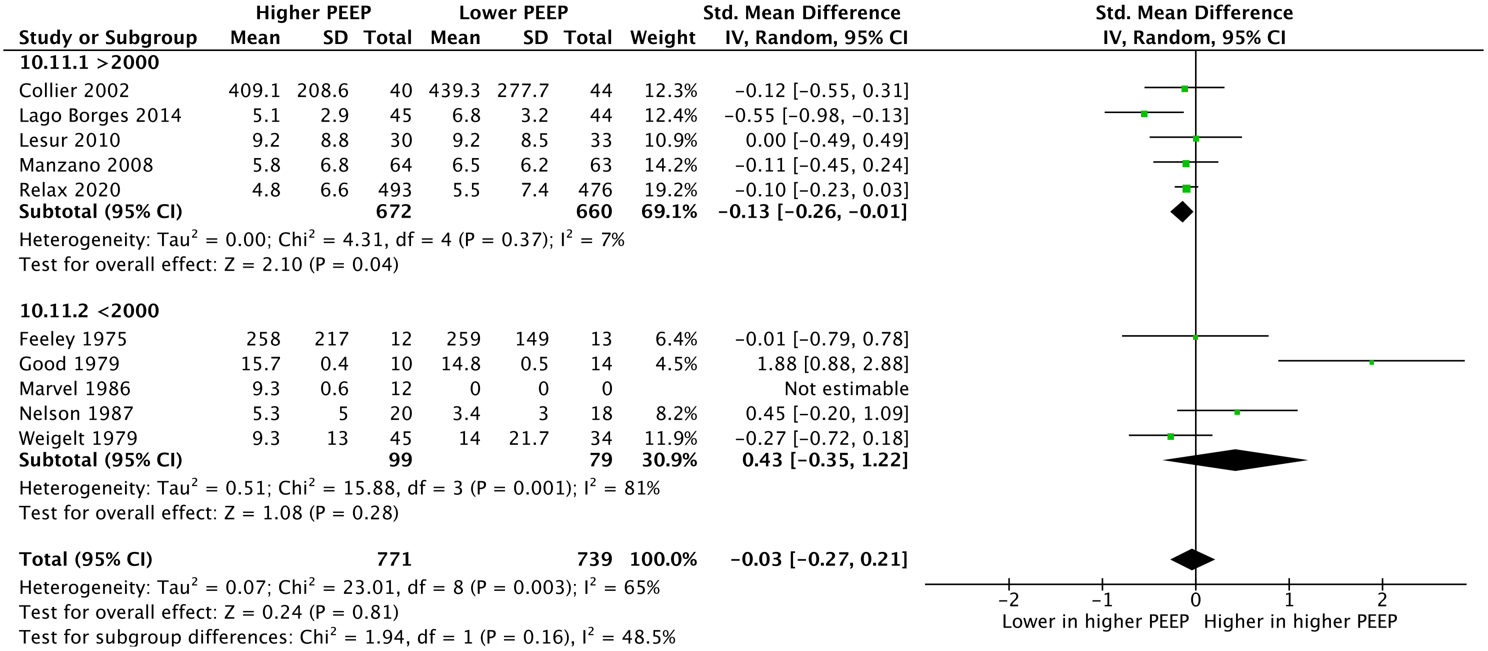

Subgroup analysis (year of publication > 2000 vs. < 2000)

ICU stay

Study or Subgroup	Higher PEEP Mean	Higher PEEP SD	Higher PEEP Total	Lower PEEP Mean	Lower PEEP SD	Lower PEEP Total	Weight	Mean Difference IV, Random, 95% CI
**10.12.1 >2000**								
Korovesi 2011	17.2	10.1	15	14.4	8.44	12	4.6%	2.80 [-4.19, 9.79]
Manzano 2008	10.5	9.8	64	12.3	11.4	63	15.5%	-1.80 [-5.50, 1.90]
Relax 2020	7.2	10.3	493	8.1	11.5	476	78.4%	-0.90 [-2.28, 0.48]
**Subtotal (95% CI)**			**572**			**551**	**98.4%**	**-0.88 [-2.15, 0.38]**
Heterogeneity: $Tau^2 = 0.00$; $Chi^2 = 1.30$, df = 2 (P = 0.52); $I^2 = 0\%$								
Test for overall effect: Z = 1.37 (P = 0.17)								
**10.12.2 <2000**								
Weigelt 1979	11.7	16.8	45	21	32.5	34	1.6%	-9.30 [-21.28, 2.68]
**Subtotal (95% CI)**			**45**			**34**	**1.6%**	**-9.30 [-21.28, 2.68]**
Heterogeneity: Not applicable								
Test for overall effect: Z = 1.52 (P = 0.13)								
**Total (95% CI)**			**617**			**585**	**100.0%**	**-1.00 [-2.51, 0.51]**
Heterogeneity: $Tau^2 = 0.26$; $Chi^2 = 3.18$, df = 3 (P = 0.37); $I^2 = 6\%$								
Test for overall effect: Z = 1.30 (P = 0.19)								
Test for subgroup differences: $Chi^2 = 1.88$, df = 1 (P = 0.17), $I^2 = 46.7\%$								

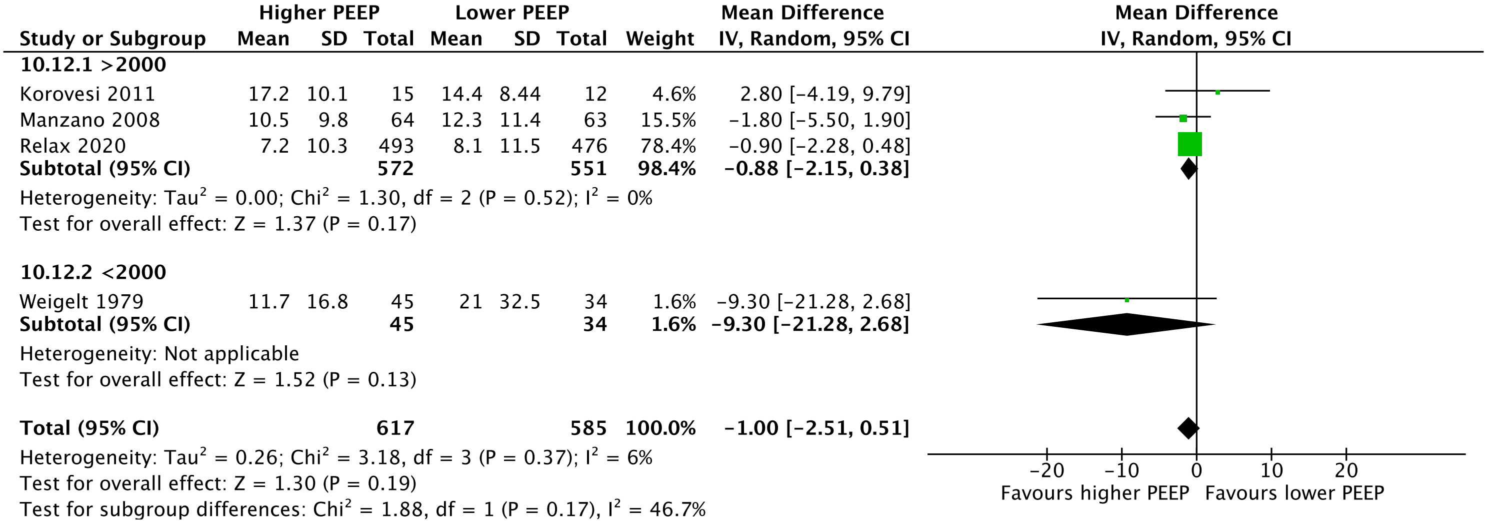

Subgroup analysis (year of publication > 2000 vs. < 2000)

Hospital stay

Study or Subgroup	Higher PEEP Mean	Higher PEEP SD	Higher PEEP Total	Lower PEEP Mean	Lower PEEP SD	Lower PEEP Total	Weight	Mean Difference IV, Random, 95% CI
**10.13.1 >2000**								
Collier 2002	5.7	2.5	40	5.2	1.7	44	31.1%	0.50 [−0.42, 1.42]
Manzano 2008	19.5	18.2	64	26.3	22	63	0.9%	−6.80 [−13.83, 0.23]
Relax 2020	19	21.4	493	19.9	22.1	476	5.6%	−0.90 [−3.64, 1.84]
**Subtotal (95% CI)**			**597**			**583**	**37.6%**	**−0.73 [−3.21, 1.74]**
Heterogeneity: $Tau^2 = 2.67$; $Chi^2 = 4.82$, df = 2 (P = 0.09); $I^2 = 59\%$								
Test for overall effect: Z = 0.58 (P = 0.56)								
**10.13.2 <2000**								
Marvel 1986	8.8	0.5	12	8.9	0.4	15	62.2%	−0.10 [−0.45, 0.25]
Nelson 1987	28	24	20	26	24	18	0.2%	2.00 [−13.28, 17.28]
**Subtotal (95% CI)**			**32**			**33**	**62.4%**	**−0.10 [−0.45, 0.25]**
Heterogeneity: $Tau^2 = 0.00$; $Chi^2 = 0.07$, df = 1 (P = 0.79); $I^2 = 0\%$								
Test for overall effect: Z = 0.56 (P = 0.58)								
**Total (95% CI)**			**629**			**616**	**100.0%**	**−0.02 [−0.69, 0.66]**
Heterogeneity: $Tau^2 = 0.16$; $Chi^2 = 5.42$, df = 4 (P = 0.25); $I^2 = 26\%$								
Test for overall effect: Z = 0.04 (P = 0.96)								
Test for subgroup differences: $Chi^2 = 0.25$, df = 1 (P = 0.62), $I^2 = 0\%$								

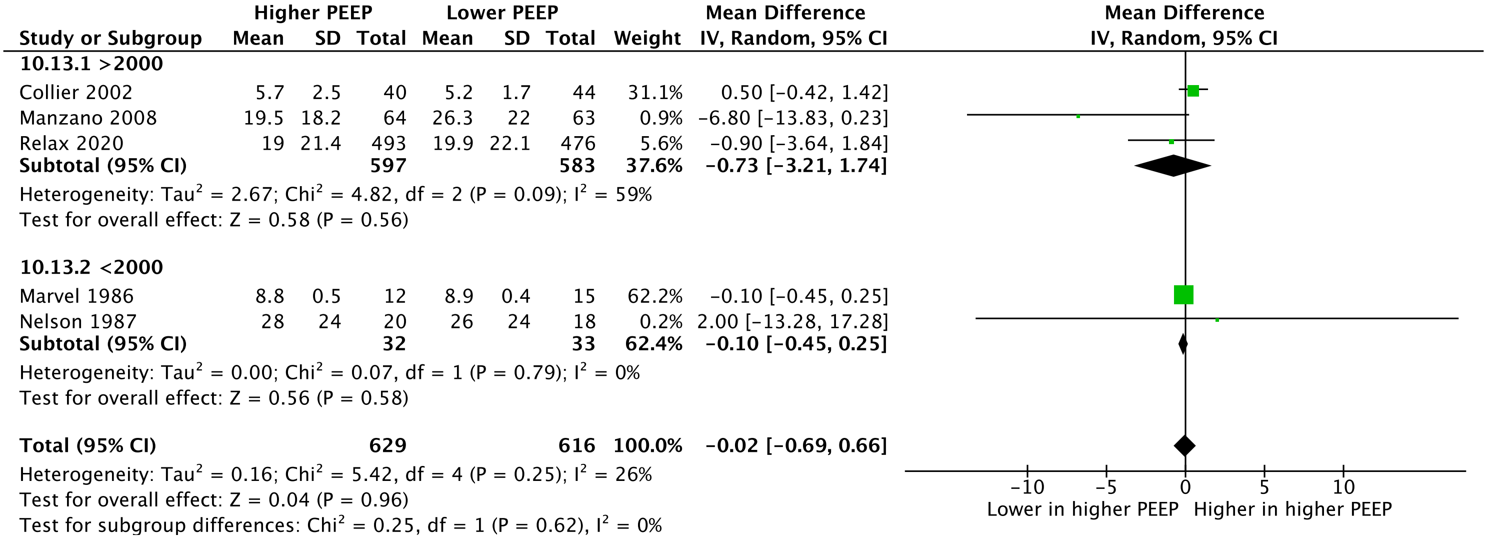

Subgroup analysis (year of publication > 2000 vs. < 2000)

ICU mortality

Study or Subgroup	Higher PEEP Events	Higher PEEP Total	Lower PEEP Events	Lower PEEP Total	Weight	Risk Ratio M-H, Random, 95% CI
**10.14.1 >2000**						
Dyhr 2002	0	7	0	8		Not estimable
Korovesi 2011	3	15	4	12	1.6%	0.60 [0.17, 2.18]
Relax 2020	185	492	163	476	96.0%	1.10 [0.93, 1.30]
**Subtotal (95% CI)**		**514**		**496**	**97.7%**	**1.09 [0.92, 1.28]**
Total events	188		167			
Heterogeneity: $Tau^2 = 0.00$; $Chi^2 = 0.83$, df = 1 (P = 0.36); $I^2 = 0\%$						
Test for overall effect: Z = 0.98 (P = 0.33)						
**10.14.2 <2000**						
Feeley 1975	2	12	1	13	0.5%	2.17 [0.22, 20.94]
Nelson 1987	4	20	4	18	1.8%	0.90 [0.26, 3.08]
**Subtotal (95% CI)**		**32**		**31**	**2.3%**	**1.10 [0.37, 3.24]**
Total events	6		5			
Heterogeneity: $Tau^2 = 0.00$; $Chi^2 = 0.45$, df = 1 (P = 0.50); $I^2 = 0\%$						
Test for overall effect: Z = 0.17 (P = 0.86)						
**Total (95% CI)**		**546**		**527**	**100.0%**	**1.09 [0.92, 1.28]**
Total events	194		172			
Heterogeneity: $Tau^2 = 0.00$; $Chi^2 = 1.27$, df = 3 (P = 0.74); $I^2 = 0\%$						
Test for overall effect: Z = 0.99 (P = 0.32)						
Test for subgroup differences: $Chi^2 = 0.00$, df = 1 (P = 0.98), $I^2 = 0\%$						

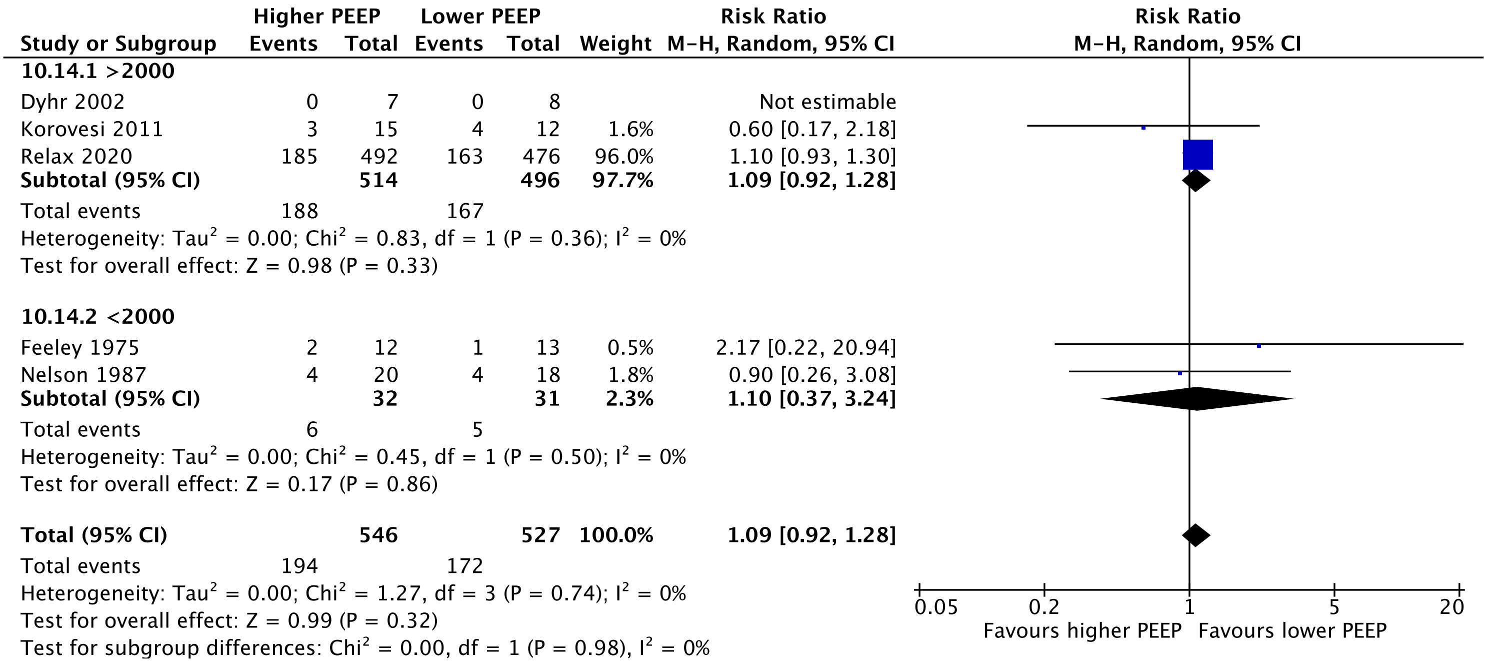

Online Resource 10. Meta-regression

Table S9. Meta-regression for the association between PEEP level and primary outcome and main secondary outcomes using tidal volume as covariate

Outcome	β (95% confidence interval)	P value
Hospital mortality	-0.056 (-0.116 – 0.003)	0.07
PaO_2/FiO_2	-8.20 (-32.8 – 16.4)	0.51
Risk of hypoxemia	-0.41 (-0.77 – -0.06)	0.02
Risk of barotrauma	0.08 (-0.06 – 0.22)	0.28
Risk of ARDS	0.01 (-0.12 – 0.15)	0.84
Duration of ventilation	0.01 (-0.05 – 0.06)	0.80
Hospital stay	-0.03 (-0.39 – 0.34)	0.89

Abbreviations: PEEP, positive end-expiratory pressure; PaO_2/FiO_2, arterial partial pressure of oxygen to fraction of inspired oxygen ratio; ARDS, acute respiratory distress syndrome.

Online Resource 11. Trial sequential analysis

Trial sequential analysis assessing the relationship between higher vs. lower positive end-expiratory pressure (PEEP) and hospital mortality. The required information sizes to demonstrate or reject an 11% (vertical red line) relative risk reduction (RRR) for higher PEEP with a control group proportion of 33%, an alpha of 5%, and a beta of 10% is 6845 patients. The blue line represents the cumulative Z-curve of the meta-analysis of 1502 patients. The oblique red lines represent the trial sequential monitoring boundaries and the futility boundaries for 11% RRR, respectively. The green horizontal lines are the conventional 5% significance thresholds (Z-value = 1.96). A constant continuity correction of 1 was applied.

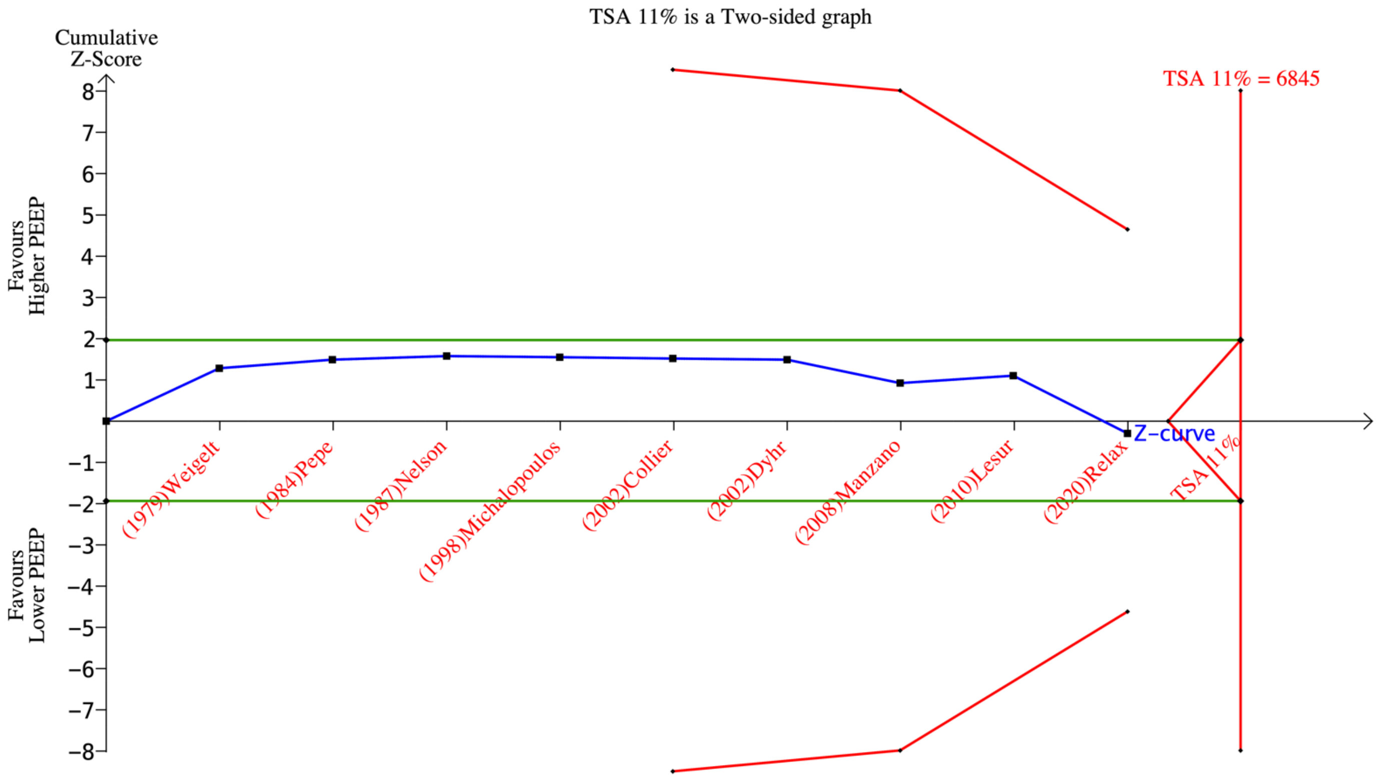

References

S1. Sterne JAC, Savović J, Page MJ, et al. RoB 2: a revised tool for assessing risk of bias in randomised trials. *BMJ*. 2019;366:l4898. doi:10.1136/bmj.l4898.
